# Ni-catalyzed asymmetric decarboxylation for the construction of carbocycles with contiguous quaternary carbon stereocenters†

**DOI:** 10.1039/d4sc06849a

**Published:** 2024-11-26

**Authors:** Yicheng He, Biwei Yan, Cheng Ma, Shaofei Ni, Wusheng Guo

**Affiliations:** a Frontier Institute of Science and Technology (FIST), Xi’an Jiaotong University Yanxiang Road 99 Xi’an 710045 China wusheng.guo@mail.xjtu.edu.cn gws314@126.com; b Department of Chemistry, Shantou University Shantou 515063 China

## Abstract

The first Ni-catalyzed asymmetric decarboxylative strategy for the construction of carbocycles with contiguous quaternary all-carbon stereocenters is reported. The key to the success of these reactions is the utilization of rationally designed allenylic methylene cyclic carbonates as substrates with Ni catalysis. The floppy allenylic group exerts unique electronic properties on the carbonate, which allows further asymmetric nucleophilic annulations with alkenes. These reactions can be performed at room temperature and feature wide functional group tolerance with excellent asymmetric induction that is typically >94% ee. The mechanistic insights imply that this conceptually new chemistry is completely different from previous reports on the catalytic transformation of cyclic carbonates, and thus, it offers an inventive novel methodology to create complex enantio-enriched molecules.

## Abstract 2

The first Ni-catalyzed decarboxylative construction of carbocycles with contiguous quaternary carbons is reported. The allenylic group imparts unique electronic properties on the carbonates enabling further asymmetric annulations with alkenes.

## Graphical abstract

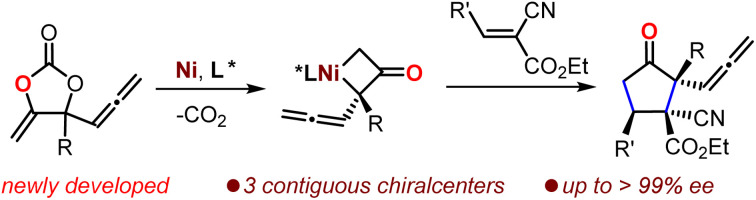

## Introduction

The contiguous quaternary carbon stereocenters (CQCSs) feature two connected sp^3^-carbon atoms with three additional and distinct stereo-defined carbon substituents on each. The creation of CQCSs is interesting because such skeletons are frequently encountered in natural products and bioactive compounds, and they also create the potential to build diverse and complex enantio-enriched organic molecules.^1^ It has been widely accepted in recent years that the growing molecular complexity that is measured by the fraction of sp^3^-hybridized carbons in drug candidates is associated largely with their clinical success.^2^ However, the establishment of CQCSs continues to be a daunting challenge, which derives from the fact that increasing steric repulsion increases difficulties for the two reaction fragments to approach each other for C–C bond formation. It is also an intimidating challenge to control such a process and proceed in a diastereo- and enantio-selective manner.

**Scheme 1:**
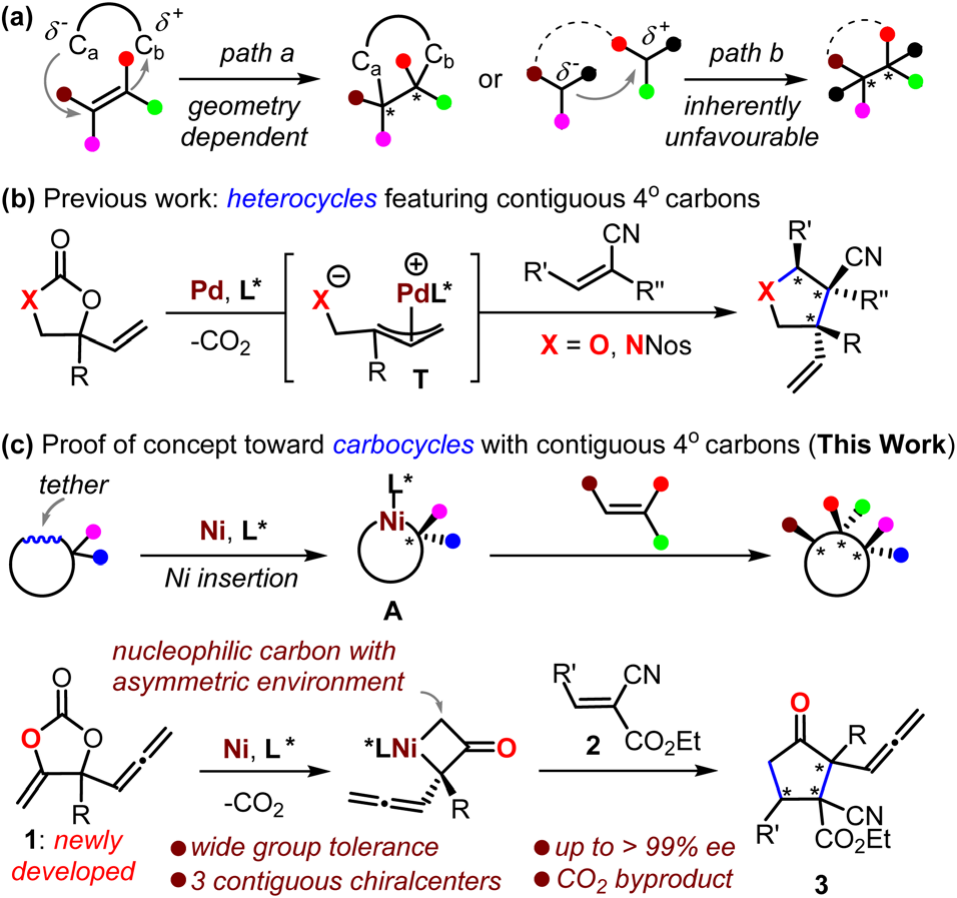
Strategies for the asymmetric construction of contiguous all-carbon quaternary stereocenters.

Difunctionalization of all-carbon substituted alkenes is feasible for the construction of CQCSs, although a limited number of successful substrates are reported.^3^ Such a protocol is circumscribed to a large extent by its requirement of a synthetically challenging stereo-defined alkene as a reaction partner (Scheme 1a, path a).^4^ Alternative methods include the sequential modification of vicinal-activated methines^5^ or desymmetrization of *meso* compounds,^6^ but such protocols are suitable only for a handful of privileged substrates. The inherently unfavorable asymmetric coupling of tertiary carbon nucleophiles and electrophiles can allow the building of CQCSs, provided that an elegant catalytic system is intriguingly designed (Scheme 1a, path b); thus far, different catalytic strategies have been devised for the synthesis of valuable molecules featuring CQCSs, where enantio-enriched cyclopropanes^7^ and heterocycles^8^ are the most investigated. To this end, research on the synthesizing CQCSs is still in its early stages,^1^ and the exploration of novel strategies is quite necessary and significantly important for the development of synthetic chemistry and related drug screening.

Decarboxylation of cyclic carbonates or their derivatives offers a user-friendly means of creating molecules. It has the advantages of mild conditions, redox neutrality, and the generation of only CO_2_ as the byproduct.^9–11^ In this context, the Ooi group, in 2014, pioneered an intriguing Pd-catalyzed decarboxylation of cyclic carbamate for the construction of enantio-enriched pyrrolidines that featured CQCSs by the intermediacy of zwitter-ionic Pd-allyl intermediate T (Scheme 1b, X = NNos).^12^ This approach was then further developed toward the formation of O-heterocycles featuring CQCSs (Scheme 1b, X = O).^10,11^ Enlightened by the elegant work from the groups of Krische^13^ and Kimura^14^ on the transformation of cyclic carbonates based on Ni-insertion chemistry, we envisaged that the asymmetric capture of a nickelacycle A that was formed *in situ* from a rationally designed cyclic carbonate with suitable acceptor would offer an unprecedented inventive methodology for the five-membered carbocycles featuring CQCSs that are otherwise quite challenging to synthesize (Scheme 1c).^3,15^ Herein, we communicate the realization of this novel concept and also the mechanistic considerations (Scheme 1c, lower).

## Results and discussion

To check our working hypothesis, we commenced the study with the use of methene cyclic carbonate 1 and cyanocrylate 2a as model substrates (Table 1). Considering that the substituent equipped on the carbonate exerts a significant effect on the nickel insertion step, a batch of methene carbonates 1a–1e was synthesized that featured electronically and sterically different groups; these were submitted for the reaction (Table 1 (a)). In the presence of Ni(cod)_2_ as a catalyst and diphosphine L1 as a ligand in toluene at room temperature, no reaction was observed, when carbonates 1a–1d were utilized; when the substituent was changed to an allenylic group (1e), the desired product could be isolated in 42% yield, although with only 44% ee and low diastereoselectivity. With this promising finding as a starting point, extensive screening data indicated that the target product 3 could be prepared with high efficiency with excellent diastero- and enantio-selectivity at room temperature (Table 1 (b), entry 1). The combination of the Ni(cod)_2_ catalyst and the L2 ligand in the NMP solvent with molecular sieves as an additive was crucial for the efficiency of the reaction (Table 1 (b), entry 1). Instead of the target product 3, a notable amount of cyclopent-2-en-1-one byproduct, which was derived from the intramolecular cyclization of the carbonate 1e, was observed with the use of palladium catalyst under otherwise identical conditions (Table 1 (b), entries 2–3). Changing the nickel catalyst or the ligand led to an inferior outcome (Table 1 (b), entries 4–9). The solvent proved to have a significant effect on either the reactivity or the diastereoselectivity (Table 1 (b), entries 10–13). The addition of molecular sieves improved the efficiency of the production (Table 1 (b), entry 14). The reaction performed at a lower temperature did not improve the desired conversion significantly (Table 1 (b), entry 15).

**Table 1:** Selected screening data for the enantio-enriched α-allenylic cyclopentanone 3 with cyclic carbonate 1 and cyanocrylate 2a as substrates

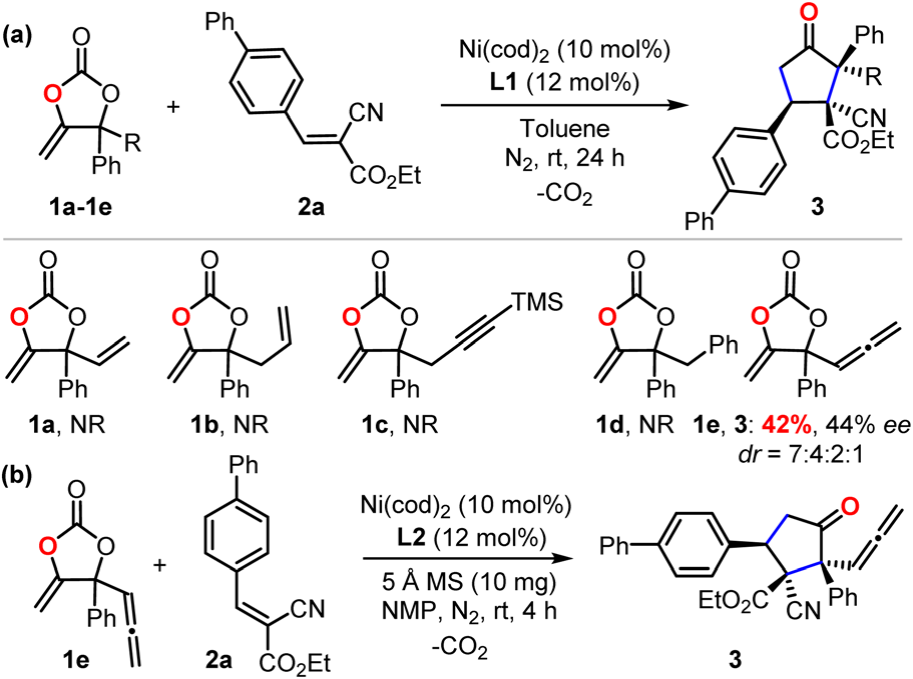				
Entry^a^	Condition variations	Yield^b^ (%)	dr^c^	ee^d^ (%)
1	None	86	10 : 1 : 1	95
2	Pd_2_(dba)_3_·CHCl_3_ catalyst	ND	—	—
3	Pd(OAc)_2_ catalyst	ND	—	—
4	Ni(glyme)Cl_2_ catalyst	ND	—	—
5	L3 as ligand	64	11 : 1 : 1	90
6	L4 as ligand	56	18 : 1 : 1	86
7	L1 as ligand	45	20 : 3 : 1 : 1	76
8	L5 as ligand	19	>20 : 1	68
9	L6–L8 as ligands	ND	—	—
10	Toluene as solvent	90	3 : 1 : 1	95
11	THF as solvent	96	5 : 1 : 1	98
12	DMF as solvent	46	16 : 1 : 1	91
13	DCM or MeOH as solvent	ND	—	—
14	Without 5 Å MS	73	10 : 1 : 1	95
15	At 0 °C	77	11 : 1 : 1	94
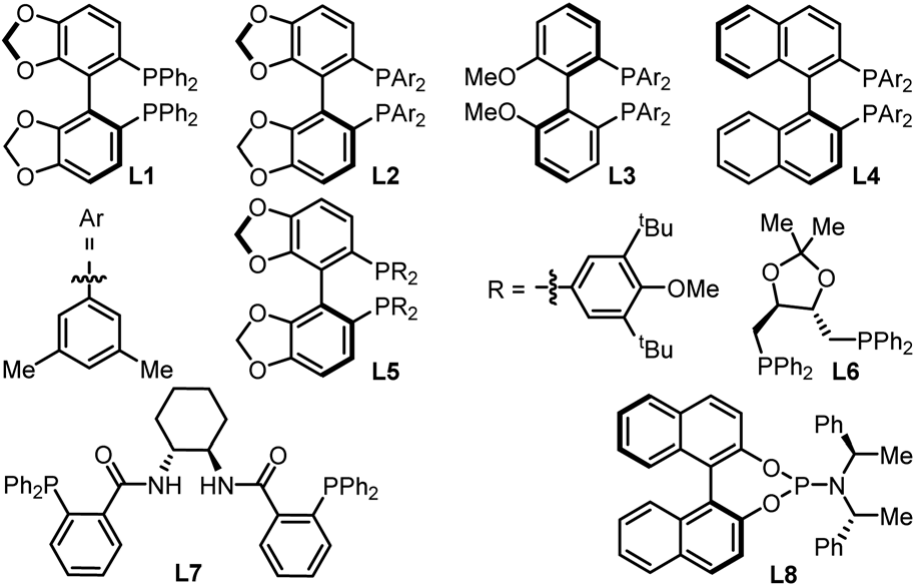				

a Reaction conditions unless otherwise noted: the cyclic carbonate 1 (0.2 mmol, 2.0 equiv.), cyanoacylate 2a (0.1 mmol), 5 Å MS (10 mg), solvent (0.3 mL), Ni(cod)_2_ catalyst (10 mol%), L (12 mol%), room temperature, 4 h.

b The yield of all the diastereoisomers was reported.

c The dr (diastereomeric ratio) was determined by ^1^H NMR spectroscopy.

d The ee (enantiomeric excess) was determined by HPLC equipped with a chiral column.

**Fig. 1:**
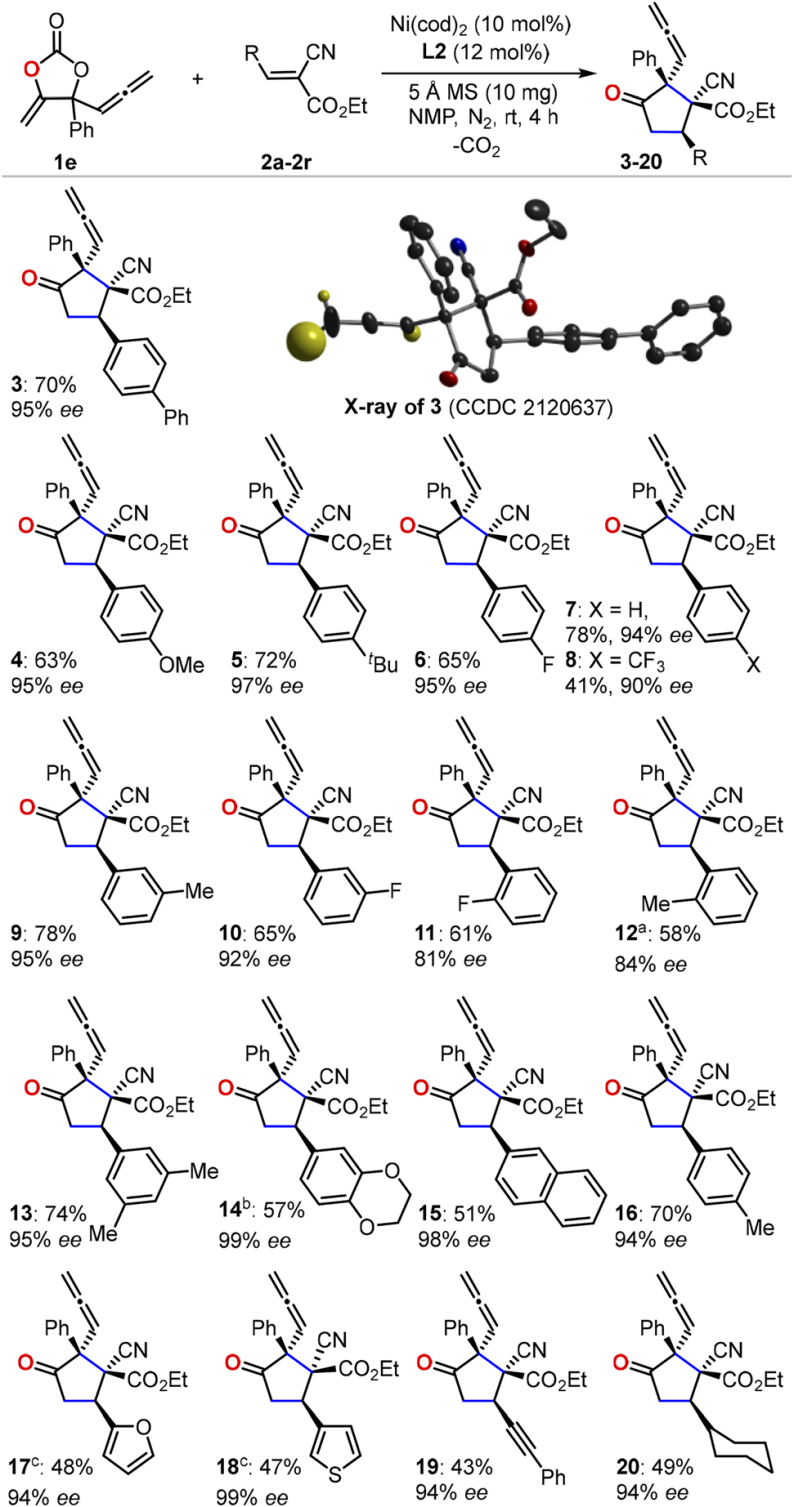
An investigation of cyanoacrylate scope. Isolated yield of a single diastereoisomer is reported and the enantioselectivity is determined by HPLC equipped with a chiral column. For clarity, only the hydrogen atoms on the allenylic group are shown in the crystal of the product 3. ^*a*^8 h. ^*b*^72 h. ^*c*^24 h.

**Fig. 2:**
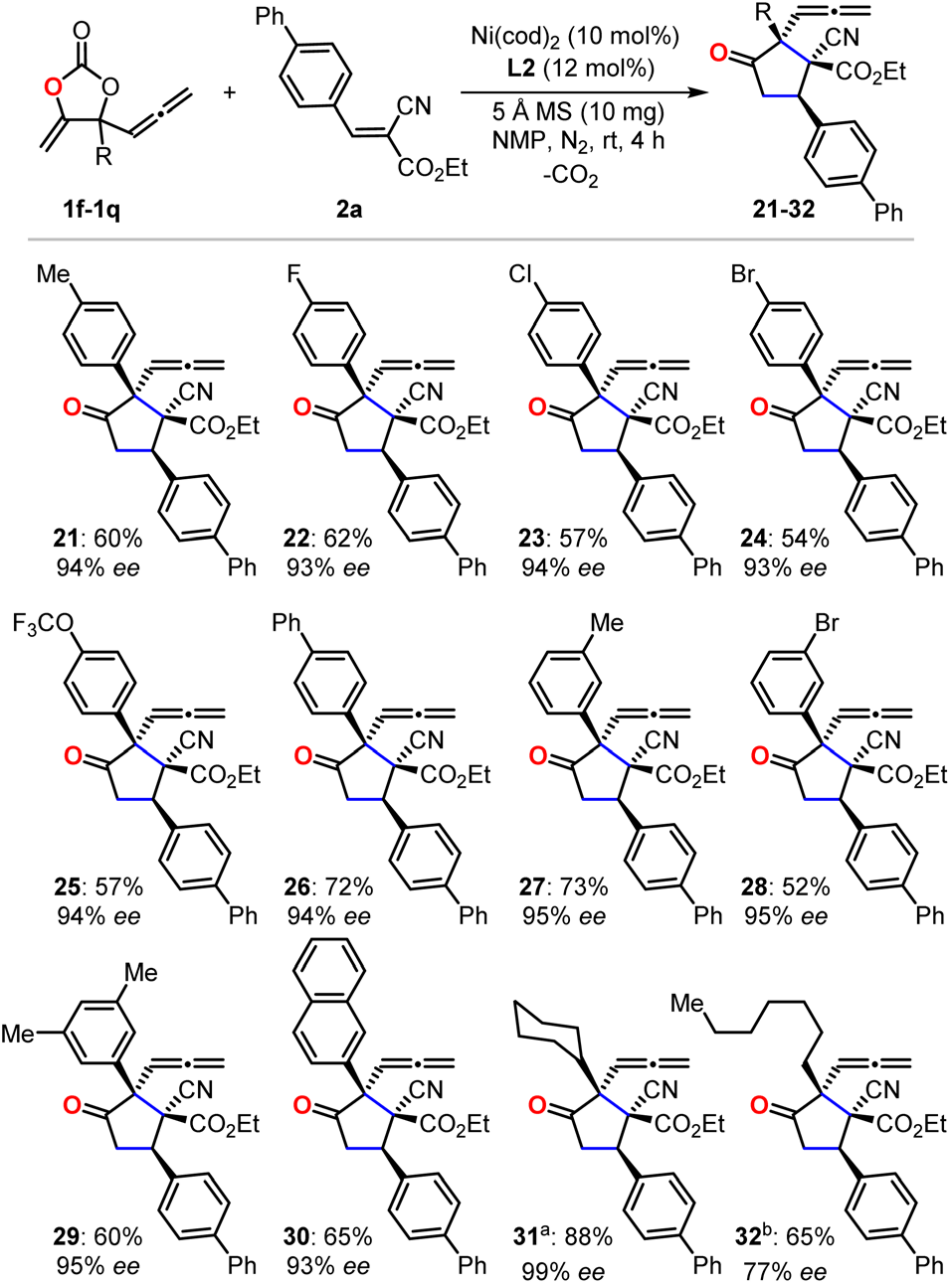
The investigation of allenylic carbonate scope. Isolated yield of the single diastereoisomer is reported, and the enantio-selectivity is determined by HPLC equipped with a chiral column. ^*a*^72 h. ^*b*^12 h.

With the optimized reaction conditions in hand, we first evaluated the scope of cyanoacrylates 2a–2r to react with the carbonate 1e as the model substrate for the formation of enantio-enriched α-allenylic cyclopentanones (Fig. 1). To our delight, this nickel-based catalytic system worked quite well with cyanocrylates containing different functional groups on the aryl substituent, resulting in the products 3–16 with excellent enantioselectivity and mostly >94% ee. Shifting the functional group on the aryl substituent of the cyanoacrylate 2 from *para*- to *meta*-position (6*vs.*10 and 9*vs.*16) did not affect the production or the enantio-selectivity of the reactions. In contrast, the cyanoacrylates that featured *ortho*-substituted aryl group showed relatively lower efficiency for the electron-donating (12) or -withdrawing (11) group; these results suggest that other than the electronic effect, the steric effect dominates during the asymmetric nucleophilic cyclization process. More promisingly, the introduction of heterocyclic functional groups into the products was feasible with excellent enantio-selectivity, such as furyl and thienyl functionalities (17 and 18). The alkynyl- (19) and alkyl-substituted (20) cyanoacrylates also showed satisfactory reactivities to yield the corresponding products in 94% ee. The absolute configuration of the products could be deduced from the unambiguous X-ray analysis of product 3 (inset in Fig. 1). It is worth noting that this approach enabled the retention of the rather reactive allenylic group which is quite useful for the synthesis of a number of natural products, pharmaceuticals, and chemical materials.^16,17^ Next, we investigated the scope of allenylic cyclic carbonates 1f–1q (Fig. 2). A large range of cyclic carbonates bearing functionalized aryl groups (21–30) were feasible for the effective reactions and led to the corresponding products with excellent enantio-selectivity. In contrast, the cyclic carbonate featuring the *ortho*-substituted aryl group was not productive, which further demonstrated the significant steric effect on the reactions. The installation of the bromo-group in the products (24 and 28) creates more potential for diversity upon treatment with Suzuki-coupling reactions. Notably, the alkyl-functionalized carbonates showed promising reactivity resulting in the desired products (31–32).

**Fig. 3:**
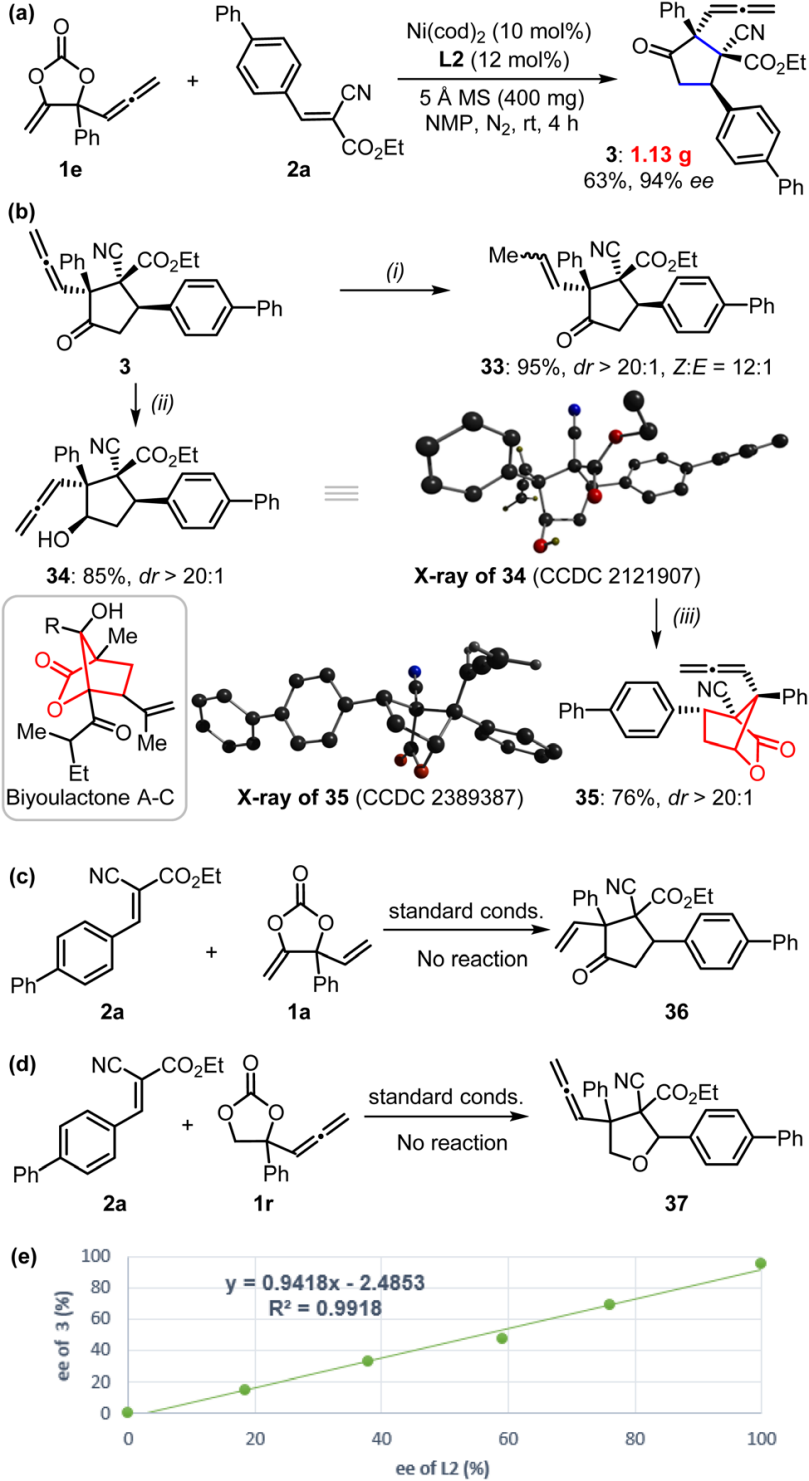
(a) Gram-scale synthesis of α-allenylic cyclopentanone 3. (b) Synthetic transformations of cyclopentanone 3: (i) Pd/C (10%), H_2_, MeOH, rt, 4 h; (ii) NaBH_4_, MeOH/THF (v/v: 2/1), 0 °C to rt, 72 h; (iii) 20 mol% Bi(OTf)_3_, DCE, 80 °C, 12 h. The inset is the X-ray of compounds 34 and 35; only the hydroxyl and allenylic hydrogens are shown for clarity. (c–e) Mechanistic considerations.

These reactions could be performed on a larger scale as demonstrated by the gram-scale synthesis of product 3 (Fig. 3a). In order to further showcase the synthetic value of this approach, we carried out late-stage transformations of the product (Fig. 3b). For example, the allenylic group could be easily converted into internal alkenyl functionality (33) in the presence of H_2_ with Pd/C catalysts. Selective reduction of the carbonyl of product 3 could be achieved using NaBH_4_ as a reducing agent to afford compound 34 with the reactive allenylic group intact; this product possesses four contiguous chiral carbon centers, which is quite challenging to synthesize. The X-ray analysis further confirmed its absolute configuration. Treatment of compound 34 in the presence of Lewis acid catalyst produced a bridged [2,2,1] bicyclic lactones 35, which is the derivative of biyoulactone **A–C**, a kind of compound with pharmaceutical interests.^18^

**Fig. 4:**
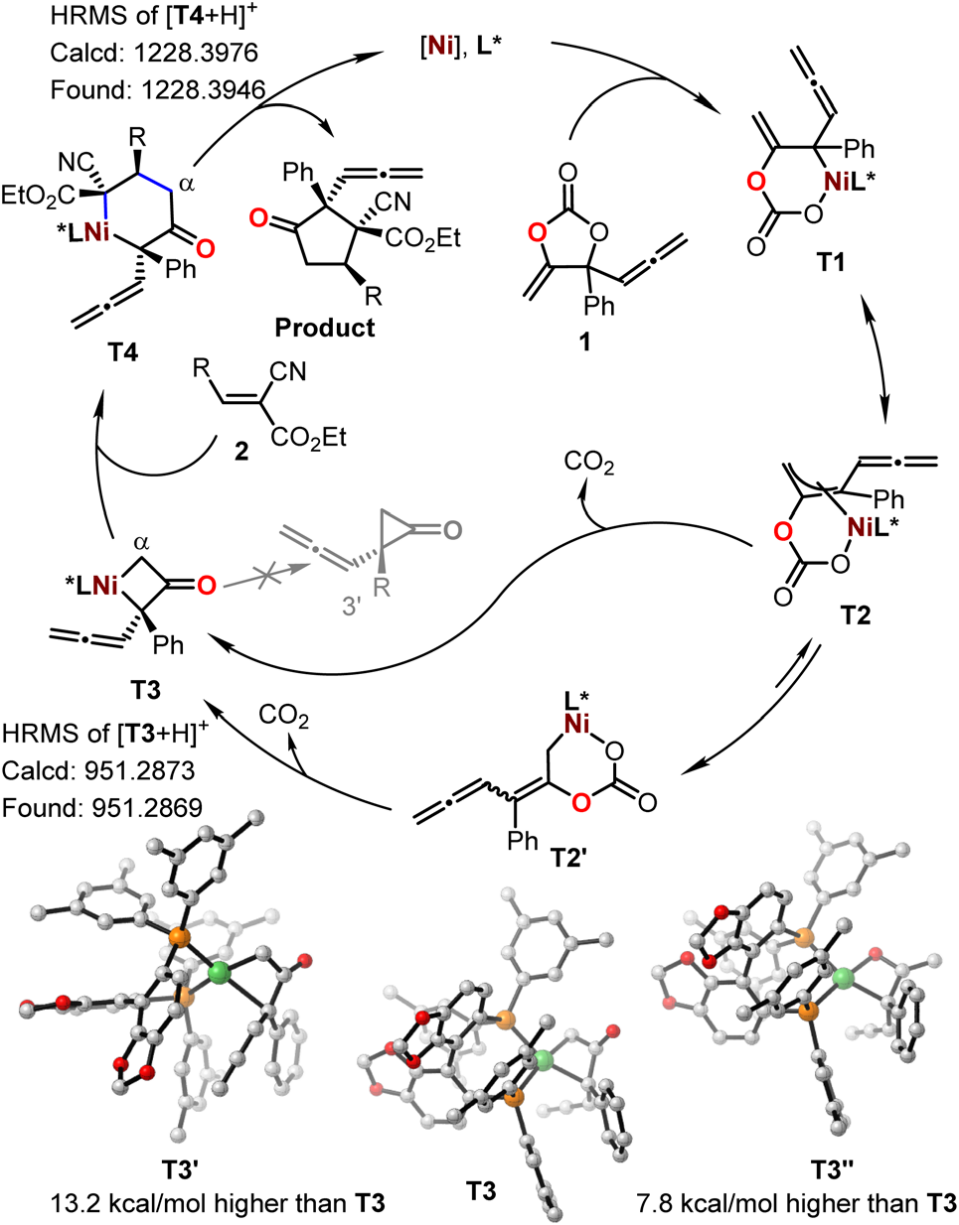
Mechanistic proposal.

To further gain insight into the reaction mechanism, we conducted control experiments and compared DFT calculations. No reaction was observed with the treatment of cyanocrylate 2a with either vinyl methene carbonate 1a or allenylic cyclic carbonate 1r,^19^ under the standard reaction conditions (Fig. 3c and d). In combination with the observations by others,^13,14,20,21^ these results implied that the Ni-catalyzed reactions showed in Fig. 1 and 2 probably did not proceed through a Ni-π-allyl species. A linear relationship between the enantio-purity of L2 and the corresponding enantio-selectivity of the resultant α-allenylic cyclopentanone 3 was observed (Fig. 3e), suggesting that one molecule of ligand L2 is involved in the enantio-discrimination step. Based on our experimental results and previous reports^13,14,20,21^ on nickel catalysis, we proposed the title reaction proceeded as depicted in Fig. 4. Firstly, an oxidative nickel insertion took place in the C–O bond of the carbonate 1 to afford the six-membered nickelacycle T1. The isomerization of T1 to T2, or the more stable form T2′,^14^ resulted in a dynamic equilibrium. In the presence of chiral ligand L2, the decarboxylation of T2 or T2′ produced an enantio-enriched and thermodynamically more favorable four-membered nickelacycle T3 which was also optimized by computational calculations (Fig. 4, inset). As the corresponding enantiomer of T3, T3′ was also calculated, whose energy was 13.2 kcal mol^−1^ higher than that of T3.^22^ As another isomer of T3, four-membered nickelacycle T3′′ featured a Ni–O bond that also showed energy that was 7.8 kcal mol^−1^ higher than that of T3. The α-carbon nucleophilic asymmetric cyclization between T3 and the alkene 2 would generate intermediate T4. Upon reductive elimination, the enantio-enriched tittle product was produced, and both the nickel catalyst and ligand were regenerated. Subsequently, we analyzed the frontier molecular orbital interactions between the LUMO of alkene 2a (R = 4-Ph-Ar) and the HOMO of an imaginary intermediate T3-vinyl that might be derived from the cyclic carbonate 1a (Table 1), in which the four-membered intermediate was functionalized with a vinyl moiety, instead of an allenylic group. Computational analysis suggested a larger HOMO orbital coefficient on the α-carbon of T3 (3.1%) than that of the T3-vinyl (1.8%).^22^ Similar results (3.1% *vs.* 3.0%) were also obtained from the calculations of relevant intermediate that may be derived from the cyclic carbonate 1c (Table 1).^22^ These results indicated that the allenylic group exerts unique electronic properties on the cyclic carbonates 1e–1q in the current Ni-based catalytic system. It is also logical to conclude that steric substituents on the cyclic carbonate may prevent the formation of T1*via* nickel insertion. The reductive elimination of T3 is thermodynamically unfavourable due to the high ring strain of the resultant cyclopropanone 3′, which is a key for the chemoselective formation of the target products. Observation of the signals of intermediates T1–T4 in high-resolution mass spectra further supports the proposed mechanism.^22^

## Conclusions

In summary, we developed the first Ni-catalyzed decarboxylative strategy for the construction of carbocycles with contiguous quaternary carbon stereocenters, which are otherwise quite challenging to synthesize. The key to the success of these reactions is the utilization of rationally designed allenylic methylene cyclic carbonates as substrates under nickel catalysis. The floppy allenylic group imparts unique electronic properties on the carbonate, enabling further asymmetric nucleophilic annulations with alkenes. The mechanistic insight gained here indicates that this conceptually new chemistry is completely different from previous reports on the catalytic transformation of cyclic carbonates, and thus, our findings offer an inventive and novel methodology for creating complex enantio-enriched molecules. These reactions could be performed at room temperature and feature wide functional group tolerance with excellent asymmetric induction that is typically >94% ee.

## Data availability

The ESI† includes experimental details and data from HPLC, NMR, and HRMS analyses.

## Author contributions

Y. H. and W. G. designed the project. Y. H. and B. Y. performed the experiments. C. M. and S. N. performed the computational calculations, and S. N. supervised the DFT calculations. Y. H and W. G. wrote the manuscript, and W. G. supervised the research.

## Conflicts of interest

There are no conflicts to declare.

## Supplementary Material

SC-OLF-D4SC06849A-s001

SC-OLF-D4SC06849A-s002
