# Supplementary material for: Ni-catalyzed asymmetric decarboxylation for the construction of carbocycles with contiguous quaternary carbon stereocenters
Source: Chem Sci. 2024 Nov 26;16(2):834–9. doi: 10.1039/d4sc06849a (PMC11621946; doi:10.1039/d4sc06849a)

Supplementary Information (SI) for Chemical Science.
This journal is © The Royal Society of Chemistry 2024

Supporting Information for:

Ni-Catalyzed Asymmetric Decarboxylation for the Construction of Carbocycles with Contiguous Quaternary Carbon Stereocenters

Yicheng He,[†] Biwei Yan,[†] Cheng Ma,[§] Shaofei Ni,[§] and Wusheng Guo[†,*]

[†]*Frontier Institute of Science and Technology (FIST), Xi'an Jiaotong University, Yanxiang Road 99, Xi'an 710045, China*

[§]*Department of Chemistry, Shantou University, Shantou 515063, China*

E-mail: wusheng.guo@mail.xjtu.edu.cn

Contents:

Page S2: General comments
Page S2: Typical procedure for the preparation of cyclic carbonates
Page S5: Typical procedure for the synthesis of α-allenylic cyclopentanones
Page S5: Table S1: Selective screening data toward **3**
Page S6: Gram-scale reaction
Page S6: Synthetic transformations
Page S7: ESI-HRMS analysis of the key intermediates
Page S9: Characterization data of all the new compounds
Page S50: X-ray crystallographic information of product **3**
Page S67: X-ray crystallographic information of product **34**
Page S77: X-ray crystallographic information of product **35**
Page S87: Computational details
Page S88: Cartesian coordinates of the optimized structures
Page S102: References
Page S103: NMR spectra

General comments

Commercially available reagents and solvents were purchased from Energy, J&K, TCI, aladdin or Daicel, and used without further purification. ^{1}H NMR, ^{13}C NMR and ^{19}F NMR spectra were recorded at room temperature on a Bruker AV-400 spectrometer and referenced to the residual deuterated solvent signals ($CDCl_3$ ^{1}H NMR, δ = 7.26; ^{13}C NMR, δ = 77.16). All reported NMR values are given in parts per million (ppm). FT-IR measurements were carried out on a Bruker ALPHA II. High resolution mass spectra (HRMS) were obtained on a WATERS I-Class VION IMS Qtof Spectrometer. The X-ray analysis of **3** and **33** was collected at 100 K on a Rigaku Oxford Diffraction Supernova Dual Source, Cu at Zero equipped with an AtlasS2 CCD using Cu Kα radiation. Optical rotations were recorded on a polarimeter with a sodium lamp of wavelength 589 nm. Enantiomeric excesses were determined by chiral High Performance Liquid Chromatography (HPLC) analysis. HPLC samples were dissolved in HPLC grade isopropanol (IPA) unless otherwise stated. All the arylidenecyanoacetates **2**[1] and alkynyl ketones[2] were prepared according to reported procedures.

Typical procedure for the preparation of cyclic carbonates

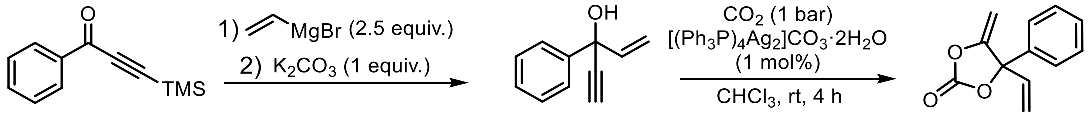

SI-1 **SI-2** **1a**

The synthesis of cyclic carbonate **1a** was prepared according to our previously reported method.[1]

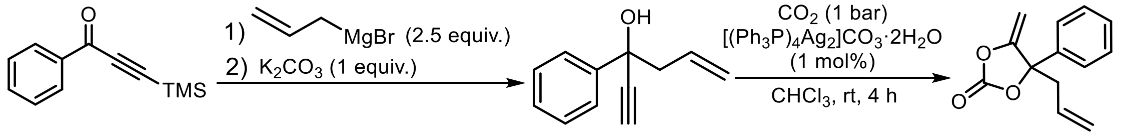

SI-1 **SI-3** **1b**

The synthesis of cyclic carbonate **1b** was prepared according to our previously reported method[1], in which the allyl magnesium bromide was used instead of vinyl magnesium bromide.

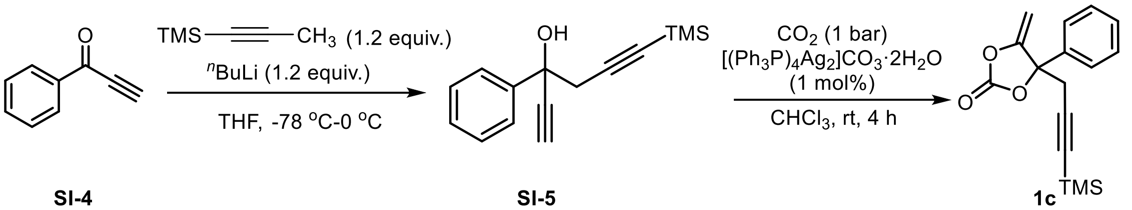

SI-4 **SI-5** **1c**

Preparation of **SI-5**: The 1-(trimethylsilyl)-1-propyne (1.2 equiv.) and anhydrous THF 20 mL were charged into a flame-dried two-necked round-bottom flask equipped with a stirring bar and dropping funnel; to which n-butyllithium reagent (1.2 equiv.) was added

dropwise under N_2 atmosphere at -78 ^oC. After that, the reaction mixture was stirred at 0 ^oC for 1 h. Then, the reaction mixture was cooled down to -78 ^oC and was added a solution of the ketone **SI-4** (5 mmol, 1.0 equiv.) in anhydrous THF 10 mL. After stirring at -78 ^oC for 6 h, the reaction mixture was quenched by saturated NH_4Cl and extracted with EtOAc (3 × 20 mL). The combined organic layers were dried over Na_2SO_4 and concentrated under reduced pressure. The mixture was purified by flash column chromatography to give pure alcohol product **SI-5.**

Preparation of cyclic carbonate **1c**: The cyclic carbonate **1c** was prepared according to our previously reported method.[1]

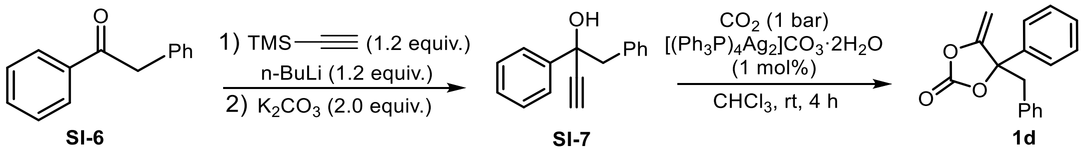

Preparation of **SI-7**: The trimethylsilylacetylene (1.2 equiv.) and anhydrous THF 20 mL were charged into a flame-dried two-necked round-bottom flask equipped with a stirring bar and dropping funnel; to which n-butyllithium reagent (1.2 equiv.) was added dropwise under N_2 atmosphere at -78 ^oC. After stirring for 1 h at -78 ^oC, to the reaction mixture was added a solution of ketone **SI-6** (5 mmol, 1.0 equiv.) in anhydrous THF 10 mL. After stirring for 6 h at -78 ^oC, the reaction mixture was quenched by saturated NH_4Cl and extracted with EtOAc (3 × 20 mL). The combined organic layers were dried over Na_2SO_4 and concentrated under reduced pressure. The mixture was purified by flash column chromatography to give pure alcohol product **SI-7**.

Preparation of cyclic carbonate **1d**: The cyclic carbonate **1d** was prepared according to our previously reported method.[1]

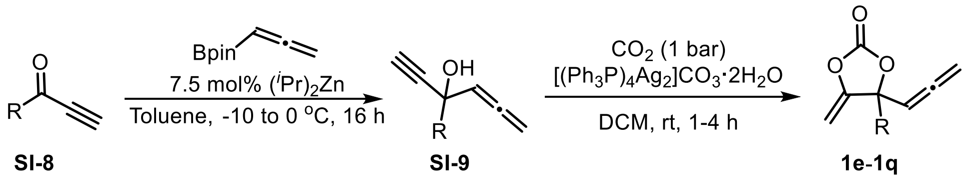

The synthesis of compound **SI-9**:[3] An oven-dried round-bottomed flask equipped with a magnetic stir bar was charged with a solution of functionalized alkynyl ketones **SI-8** (9.0 mmol, 1.0 equiv.) in dry toluene (30 mL) under nitrogen atmosphere at -10 ^oC. Then, the allenylboronic acid pinacol ester (CAS: 865350-17-0) (9.9 mmol, 1.1 equiv.) and diisopropylzinc (0.675 mmol, 0.075 equiv.) (1.0 M in toluene) was added, respectively. The reaction mixture was stirred at -10 ^oC for 1 h, then at 0 ^oC for 16 h more. After the reaction was finished, the reaction mixture was added diethanollamine (1.9 g, 18.0 mmol) and stirred at room temprature for 1 h. Next, the resulting mixture was extracted with ethyl acetate. The combined organic layers were washed with sat. NaCl aq., then dried over Na_2SO_4. Upon concentration in vacuo, the resulting residue was purified by column chromatography to give the corresponding α-allenylic propargylic alcohols **SI-9**.

The synthesis of cyclic carbonates **1e-1q**: A 10 mL of Schlenk tube equipped with a stirring bar was charged with $[(PPh_3)_2Ag]_2CO_3 \cdot 2H_2O$[4] (27.2 mg, 0.01 equiv.). Subsequently, the Schlenk tube was subjected to three cycles of pressurization/depressurization using CO_2 (99.999%). Then the α-allenylic propynols **SI-9** (2.0 mmol, 1.0 equiv.) and dry DCM (0.5 mL) were added and the resultant reaction mixture was pressured with CO_2 (1 bar). Then the reaction mixture was stirred at room temperature for 1-4 h. The excessive CO_2 was released carefully after the reaction was completed. The allenylic carbonates **1e-1q** could be obtained upon purification by flash column chromatography on silica gel.

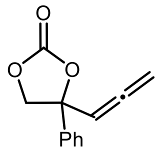

1r

The cyclic carbonate **1r** was prepared according to a previously reported procedure.[5]

Typical procedure for the synthesis of α-allenylic cyclopentanones

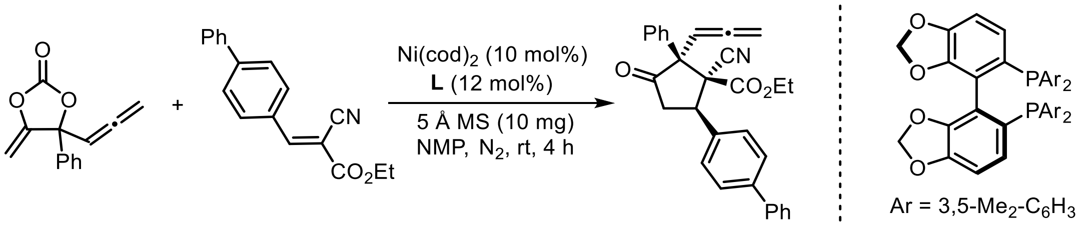

In a N_2-filled glovebox, a screw-capped vial was charged with Ni(cod)$_2$ (2.8 mg, 0.01 mmol, 10 mol%), **L** (8.7 mg, 0.012 mmol, 12 mol%), 5Å molecular sieve (10 mg) and NMP (0.2 mL). The resulting solution was stirred for 1 h at room temperature. Then, allenylic carbonate **1e** (42.8 mg, 0.2 mmol, 2.0 equiv.), arylidenecyanoacetate **2a** (27.7 mg, 0.1 mmol, 1.0 equiv.) and NMP (0.1 mL) were added to the reaction mixture. The resultant mixture was stirred at room temperature for 4 h. The crude product was purified by column chromatography (PE:EA = 50:1 to 20:1) to afford the desired product **3** as a white solid (31.3 mg, 70%, 95% *ee*).

Table S1: Selective screening data toward 3

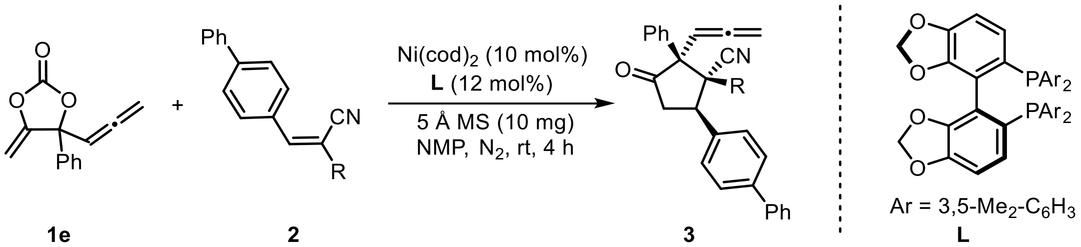

Product	**3**	**3-1**	**3-2**	**3-3**	**3-4**	**3-5**	**3-6**
R	CO_2Et	CO_2Me	$CO_2{}^n$Pr	$CO_2{}^i$Pr	$CO_2{}^t$Bu	CO_2Ph	SO_2Ph
Yield/%	86	83	82	84	90	<5	0
dr	10:1:1	12:1:1	10:1:1	9:2:1	5:2.5:1	-	-
ee/%	95	93	95	97	98	-	-

Gram-scale reaction

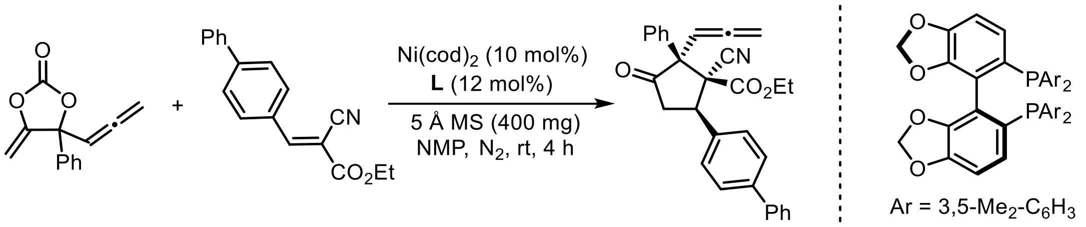

1e **2a** **3** **L**

In a N_2-filled glovebox, an oven-dried round-bottomed flask equipped with a magnetic stir bar was charged with $Ni(cod)_2$ (110 mg, 0.4 mmol, 10 mol%), **L** (347 mg, 0.48 mmol, 12 mol%), 5Å molecular sieve (400 mg) and NMP (8 mL). The resulting solution was stirred for 1 h at room temperature. Then, allenylic carbonate **1e** (1.71 g, 8 mmol, 2.0 equiv.), arylidenecyanoacetates **2a** (1.11 g, 4 mmol, 1.0 equiv.) and NMP (4 mL) were added to the mixture. The resultant mixture was stirred at room temperature for 4 h and then was extracted with ethyl acetate. The combined organic layers were dried over Na_2SO_4, filtered and concentrated in vacuo. The resultant crude product was purified by column chromatography (PE:EA = 50:1 to 20:1) to afford the desired product **3** as a white solid (1.13g, 63%, 94% *ee*).

Synthetic transformations

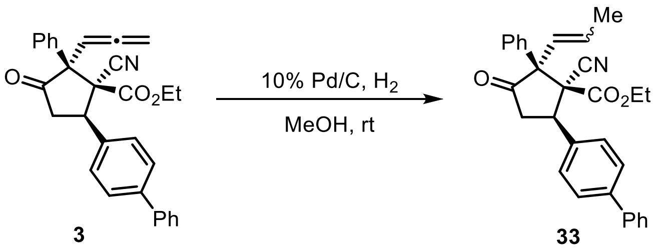

3 **33**

To a solution of **3** (44.7 mg, 0.1 mmol, 1.0 equiv.) in MeOH (2 mL). The mixture was degassed and purged with argon atomsphere. After that, 10% Pd/C (1.1 mg, 10 mol%) was carefully added. The resulting reaction mixture was degassed and purged with hydrogen. The reaction is allowed to stir for 4 h at room temperature. After the completion of the reaction, the mixture was filtered through a celite pad and concentrated under reduced pressure and purified by flash column chromatography (PE:EA = 20:1) to aford the desired product **33** (42.7 mg, 95%) as a white solid (42.7 mg, *dr* >20:1, Z/E = 12:1). The *dr* and Z/E ration were determined by ^{1}H NMR spectrum.

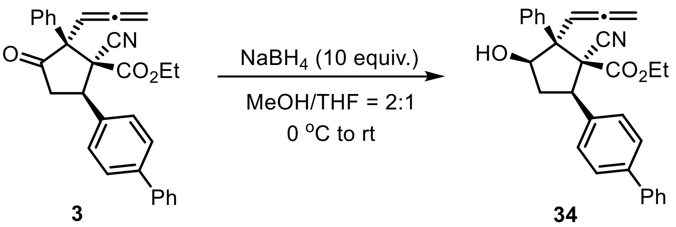

3 **34**

A 10 mL Schlenk tube equipped with a stirring bar was charged with **3** (44.7 mg, 0.1 mmol, 1.0 equiv.) in a mixed solvent (MeOH/THF, 2/1, v/v, 1.5 mL) at 0 °C was slowly added $NaBH_4$ (1.0 mmol, 37.8 mg, 10 equiv.). The reaction mixture was stirred for 72 h at room temperature. After the completion of the reaction (monitored by TLC), the mixture was filtered through a celite pad and concentrated under reduced pressure. The resultant crude product was purified by column chromatography (PE:EA = 10:1) to afford the desired product **34** as a white solid (38.2 mg, 85% yield, *dr* >20:1). The *dr* of product **34** was determined by ^{1}H NMR spectrum.

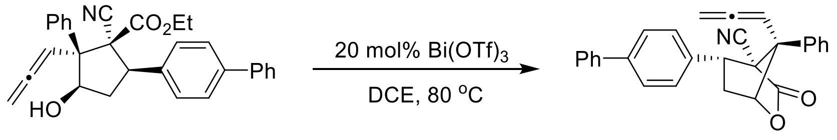

34 **35**

A 10 mL Schlenk tube equipped with a stirring bar was charged with **34** (44.7 mg, 0.1 mmol, 1.0 equiv.) and $Bi(OTf)_3$ (0.02 mmol, 13.1 mg, 0.02 equiv.) in DCE (0.2 mL) at room temperature. The reaction mixture was stirred for 12 h at 80 °C. After the completion of the reaction (monitored by TLC), the mixture was filtered through a celite pad and concentrated under reduced pressure. The resultant crude product was purified by column chromatography (PE:EA = 10:1) to afford the desired product **35** as a white solid (30.6 mg, 76% yield, *dr* >20:1). The *dr* of product **35** was determined by ^{1}H NMR spectrum.

ESI-HRMS analysis of the key intermediates

In a N_2-filled glovebox, a GC vial was charged with $Ni(cod)_2$ (2.8 mg, 0.01 mmol, 10 mol%), **L2** (8.7 mg, 0.012 mmol, 12 mol%), 5Å molecular sieve (10 mg) and NMP (0.2 mL). The resulting solution was stirred for 1 h at room temperature. Then, allenylic carbonate **1e** (42.8 mg, 0.2 mmol, 2.0 equiv.), arylidenecyanoacetate **2a** (27.7 mg, 0.1 mmol, 1.0 equiv.) and NMP (0.1 mL) were added to the reaction mixture. The resultant mixture was stirred at room temperature for 1 h more. Then the reaction mixture was taken out of the glove box, which is ready for the HRMS analysis.

HRMS spetrum of [**T1/T2/T2′**+H]$^+$

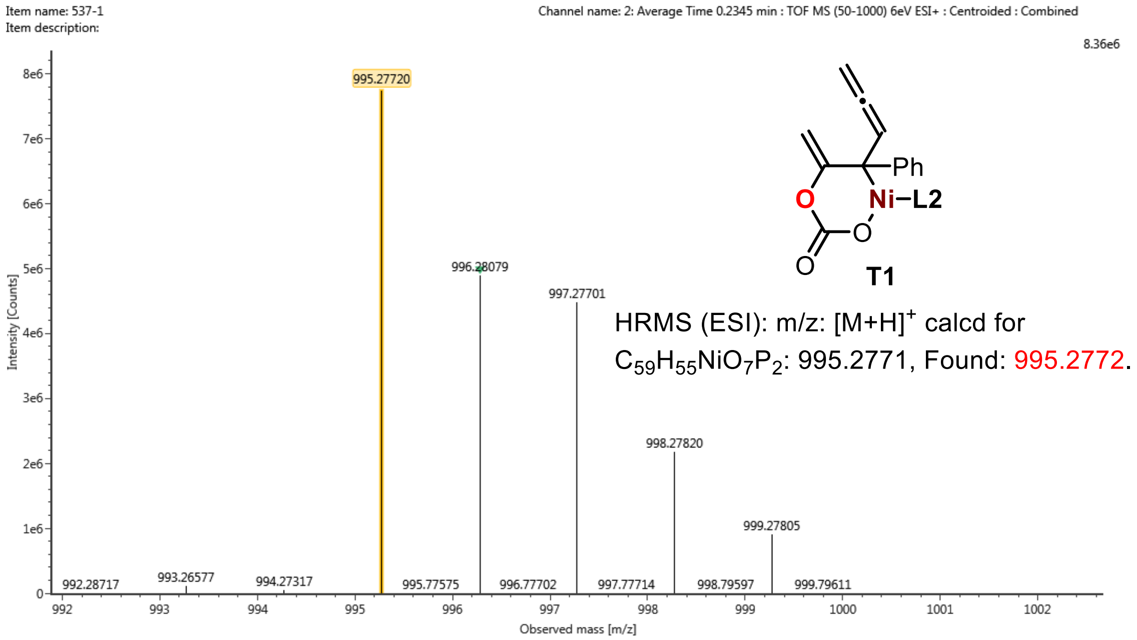

HRMS spetrum of [**T3**+H]$^+$

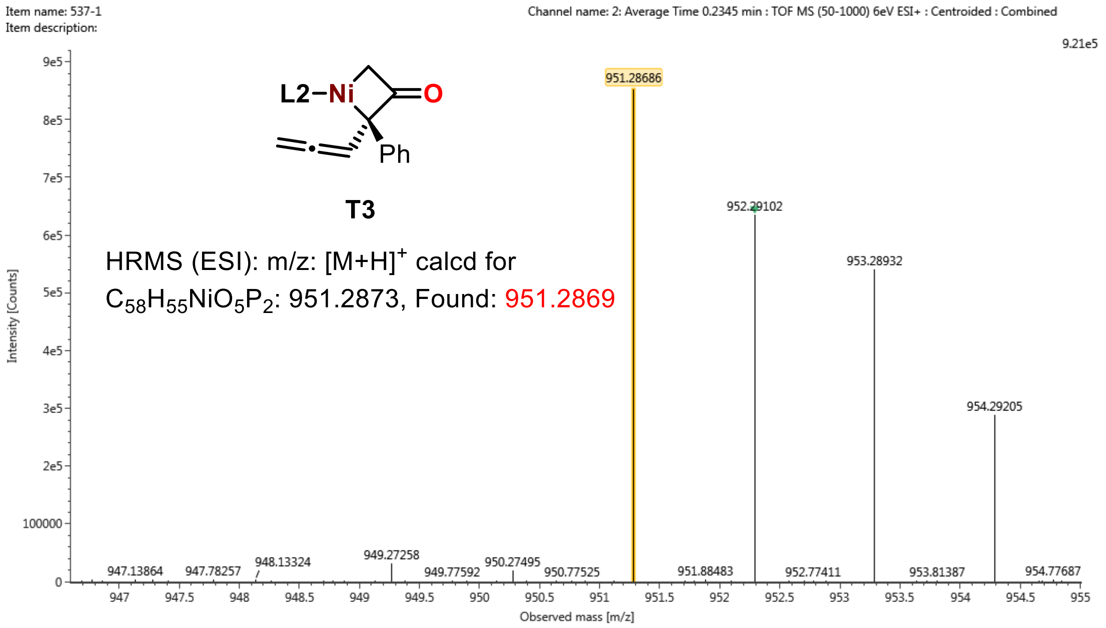

HRMS spetrum of [**T4**+H]$^+$

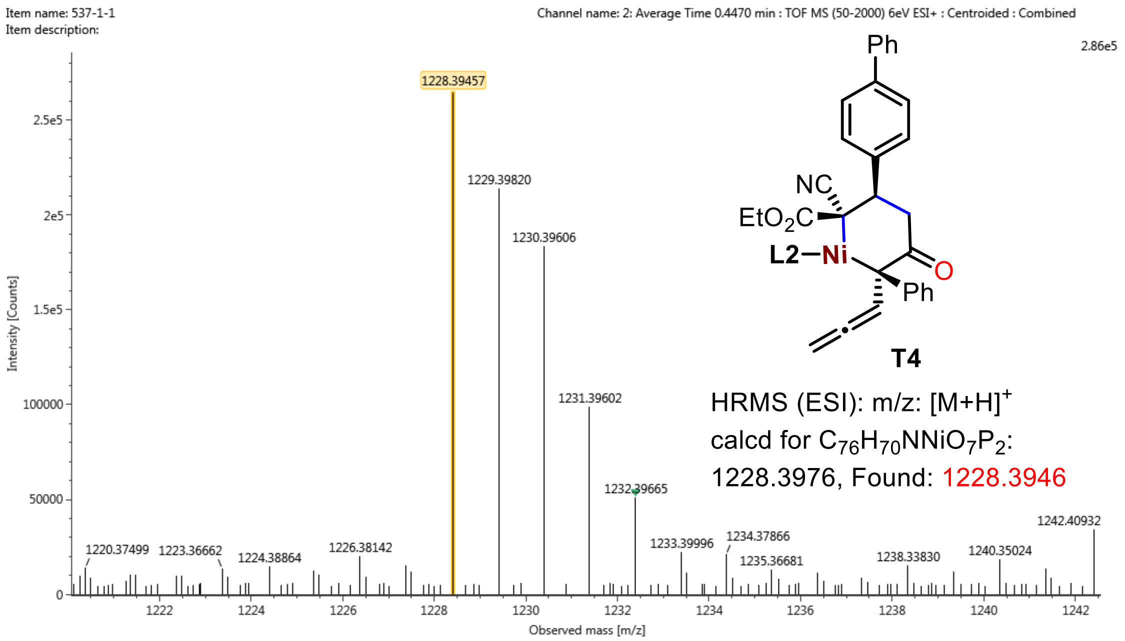

Characterization data of all the new compounds

Compounds **1a** and **2** were previously reported.[1]

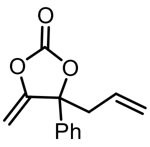

1b

4-allyl-5-methylene-4-phenyl-1,3-dioxolan-2-one (1b): ^{1}H NMR (400 MHz, $CDCl_3$) δ 7.53-7.35 (m, 5H), 5.80-5.64 (m, 1H), 5.27 (d, J = 4.3 Hz, 1H), 5.24 (s, 1H), 4.99 (d, J = 4.1 Hz, 1H), 4.51 (d, J = 4.1 Hz, 1H), 2.99 (dd, J = 14.6, 7.5 Hz, 1H), 2.89 (dd, J = 14.6, 6.7 Hz, 1H); ^{13}C NMR (100 MHz, $CDCl_3$) δ 155.5, 151.2, 138.4, 129.4, 129.2, 129.0, 124.9, 122.2, 88.84, 88.78, 44.5; IR (neat, cm^{-1}) 1821, 1680, 1447, 1265, 1181, 1101, 1068, 1023, 1000, 928, 854, 757, 696, 660; HRMS (ESI): *m/z*: [M-CO_2+H]$^+$ calcd for $C_{12}H_{13}O$: 173.0966, found: 173.0970.

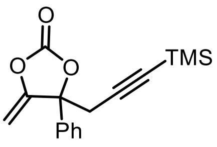

1c

5-methylene-4-phenyl-4-(3-(trimethylsilyl)prop-2-yn-1-yl)-1,3-dioxolan-2-one (1c): ^{1}H NMR (400 MHz, $CDCl_3$) δ 7.53-7.38 (m, 5H), 5.05 (d, J = 4.1 Hz, 1H), 4.60 (d, J = 4.1 Hz, 1H), 3.11 (q, J = 17.0 Hz, 2H), 0.14 (s, 9H); ^{13}C NMR (100 MHz, $CDCl_3$) δ 155.1, 151.0, 137.2, 129.6, 129.0, 125.1, 98.4, 90.2, 89.2, 87.4, 33.1, 0.3; IR (neat, cm^{-1}) 3788, 3470, 2960, 2183, 1828, 1677, 1598, 1252, 1162, 1068, 1024, 948, 839, 755, 695; HRMS (ESI): *m/z*: $[M-CO_2+H]^+$ calcd for $C_{15}H_{19}OSi$: 243.1205, found: 243.1208.

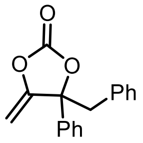

1d

4-benzyl-5-methylene-4-phenyl-1,3-dioxolan-2-one (1d): ^{1}H NMR (400 MHz, $CDCl_3$) δ 7.62-7.47 (m, 2H), 7.46-7.34 (m, 3H), 7.33-7.24 (m, 3H), 7.23-7.09 (m, 2H), 4.96 (d, J = 4.0 Hz, 1H), 4.57 (d, J = 4.0 Hz, 1H), 3.52 (d, J = 14.2 Hz, 1H), 3.37 (d, J = 14.2 Hz, 1H); ^{13}C NMR (100 MHz, $CDCl_3$) δ 155.2, 150.9, 138.7, 132.9, 131.0, 129.2, 129.0, 128.5, 127.9, 125.0, 89.4, 89.3, 46.4; IR (neat, cm^{-1}) 3902, 3749, 3012, 2929, 1811, 1683, 1497, 1436, 1284, 1150, 1028, 872, 773, 697, 655, 525; HRMS (ESI): *m/z*: $[M+H]^+$ calcd for $C_{17}H_{15}O_3$: 267.1021, found: 267.1017.

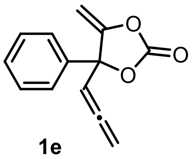

1e

5-methylene-4-phenyl-4-(propa-1,2-dien-1-yl)-1,3-dioxolan-2-one (1e): ^{1}H NMR (400 MHz, $CDCl_3$) δ 7.54-7.47 (m, 2H), 7.47-7.39 (m, 3H), 5.67 (t, J = 6.6 Hz, 1H), 5.07 (d, J = 6.6 Hz, 2H), 5.03 (d, J = 3.9 Hz, 1H), 4.42 (d, J = 3.9 Hz, 1H); ^{13}C NMR (100 MHz, $CDCl_3$) δ 208.5, 154.7, 151.1, 137.7, 129.6, 128.9, 125.9, 93.4, 90.5, 88.0, 81.1; IR (neat, cm^{-1}) 2072, 2023, 1963, 1820, 1680, 1450, 1284, 1185, 1124, 1016, 949, 853, 758, 699; HRMS (ESI): *m/z*: $[M-CO_2+H]^+$ calcd for $C_{12}H_{11}O$: 171.0810, found: 171.0814.

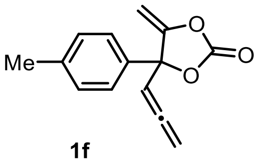

1f

5-methylene-4-(propa-1,2-dien-1-yl)-4-(p-tolyl)-1,3-dioxolan-2-one (1f): ^{1}H NMR (400 MHz, $CDCl_3$) δ 7.38 (d, J = 8.3 Hz, 2H), 7.22 (d, J = 8.1 Hz, 2H), 5.66 (t, J = 6.6

Hz, 1H), 5.06 (d, *J* = 6.6 Hz, 2H), 5.01 (d, *J* = 3.9 Hz, 1H), 4.38 (d, *J* = 3.9 Hz, 1H), 2.37 (s, 3H); ^{13}C NMR (100 MHz, $CDCl_3$) δ 208.4, 154.9, 151.2, 139.6, 134.8, 129.5, 125.9, 93.4, 90.3, 88.0, 81.0, 21.3; IR (neat, cm^{-1}) 3782, 2922, 1956, 1818, 1688, 1284, 1209, 1118, 1049, 1017, 854, 818, 761; HRMS (ESI): *m/z*: $[M-CO_2+H]^+$ calcd for $C_{13}H_{13}O$: 185.0966, found: 185.0965.

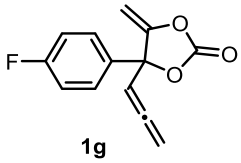

1g

4-(4-fluorophenyl)-5-methylene-4-(propa-1,2-dien-1-yl)-1,3-dioxolan-2-one (1g): ^{1}H NMR (400 MHz, $CDCl_3$) δ 7.56-7.43 (m, 2H), 7.10 (t, *J* = 8.6 Hz, 2H), 5.64 (t, *J* = 6.6 Hz, 1H), 5.05 (dd, *J* = 5.2, 3.3 Hz, 3H), 4.42 (d, *J* = 4.0 Hz, 1H); ^{13}C NMR (100 MHz, $CDCl_3$) δ 208.5, 163.3 (d, *J* = 248.0 Hz), 154.6, 150.8, 133.5 (d, *J* = 3.2 Hz), 128.2 (d, *J* = 8.6 Hz), 115.8 (d, *J* = 22.0 Hz), 93.4, 90.7, 87.6, 81.2; ^{19}F NMR (376 MHz, $CDCl_3$) δ -111.7 (s); IR (neat, cm^{-1}) 3783, 1956, 1817, 1685, 1601, 1281, 1229, 1162, 1123, 1102, 1047, 835, 760, 727; HRMS (ESI): *m/z*: $[M-CO_2+H]^+$ calcd for $C_{12}H_{10}FO$: 189.0716, found: 189.0723.

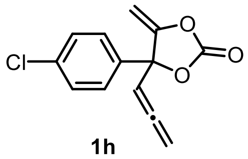

1h

4-(4-chlorophenyl)-5-methylene-4-(propa-1,2-dien-1-yl)-1,3-dioxolan-2-one (1h): ^{1}H NMR (400 MHz, $CDCl_3$) δ 7.41 (dd, *J* = 20.6, 8.7 Hz, 4H), 5.63 (t, *J* = 6.5 Hz, 1H), 5.06 (t, *J* = 5.3 Hz, 3H), 4.43 (d, *J* = 4.0 Hz, 1H); ^{13}C NMR (100 MHz, $CDCl_3$) δ 208.6, 154.4, 150.8, 136.2, 135.7, 129.1, 127.4, 93.2, 90.8, 87.5, 81.3; IR (neat, cm^{-1}) 3471, 1977, 1956, 1824, 1676, 1286, 1189, 1093, 1048, 1011, 947, 853, 827, 760, 724; HRMS (ESI): *m/z*: $[M-CO_2+H]^+$ calcd for $C_{12}H_{10}ClO$: 205.0420, found: 205.0428.

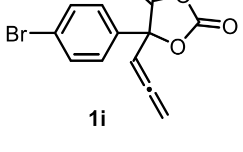

1i

4-(4-bromophenyl)-5-methylene-4-(propa-1,2-dien-1-yl)-1,3-dioxolan-2-one (1i): ^{1}H NMR (400 MHz, $CDCl_3$) δ 7.54 (d, *J* = 8.7 Hz, 2H), 7.37 (d, *J* = 8.7 Hz, 2H), 5.62 (t, *J* = 6.6 Hz, 1H), 5.06 (t, *J* = 5.6 Hz, 3H), 4.43 (d, *J* = 4.1 Hz, 1H); ^{13}C NMR (100 MHz, $CDCl_3$) δ 208.6, 154.3, 150.8, 136.7, 132.0, 127.7, 123.9, 93.1, 90.9, 87.5, 81.3; IR

(neat, cm^{-1}) 3471, 1977, 1955, 1823, 1675, 1283, 1189, 1073, 1048, 853, 823, 759; HRMS (ESI): *m/z*: $[M-CO_2+H]^+$ calcd for $C_{12}H_{10}BrO$: 248.9915, found: 248.9916.

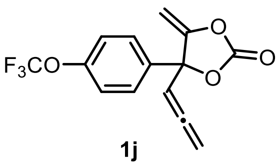

1j

5-methylene-4-(propa-1,2-dien-1-yl)-4-(4-(trifluoromethoxy)phenyl)-1,3-dioxolan-2-one (1j): ^{1}H NMR (400 MHz, $CDCl_3$) δ 7.54 (d, *J* = 8.8 Hz, 2H), 7.32-7.23 (m, 2H), 5.64 (t, *J* = 6.5 Hz, 1H), 5.12-5.02 (m, 3H), 4.45 (d, *J* = 4.1 Hz, 1H); ^{13}C NMR (100 MHz, $CDCl_3$) (one carbon signal was overlapped) δ 208.6, 154.3, 150.0 (d, *J* = 1.8 Hz), 136.2, 127.8, 121.1, 120.5 (q, *J* = 256.0 Hz), 93.2, 90.9, 87.4, 81.3; ^{19}F NMR (376 MHz, $CDCl_3$) δ -57.9 (s); IR (neat, cm^{-1}) 1977, 1956, 1832, 1681, 1253, 1208, 1159, 1049, 1014, 850, 760; HRMS (ESI): *m/z*: $[M-CO_2+H]^+$ calcd for $C_{13}H_{10}F_3O_2$: 255.0633, found: 255.0643.

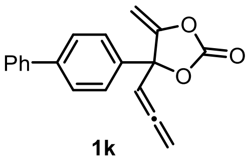

1k

4-([1,1'-biphenyl]-4-yl)-5-methylene-4-(propa-1,2-dien-1-yl)-1,3-dioxolan-2-one (1k): ^{1}H NMR (400 MHz, $CDCl_3$) δ 7.66 (d, *J* = 8.4 Hz, 2H), 7.60 (t, *J* = 7.9 Hz, 4H), 7.47 (t, *J* = 7.6 Hz, 2H), 7.40 (d, *J* = 7.1 Hz, 1H), 5.72 (t, *J* = 6.6 Hz, 1H), 5.11 (d, *J* = 6.6 Hz, 2H), 5.07 (d, *J* = 4.0 Hz, 1H), 4.47 (d, *J* = 4.0 Hz, 1H); ^{13}C NMR (100 MHz, $CDCl_3$) δ 208.4, 154.7, 151.1, 142.5, 140.1, 136.6, 129.0, 128.0, 127.5, 127.3, 126.4, 93.4, 90.6, 87.9, 81.1; IR (neat, cm^{-1}) 3784, 3062, 3028, 1973, 1947, 1830, 1681, 1483, 1316, 1279, 1211, 1109, 1053, 1015, 937, 856, 830, 760, 715; HRMS (ESI): *m/z*: $[M-CO_2+H]^+$ calcd for $C_{18}H_{15}O$: 247.1123, found: 247.1114.

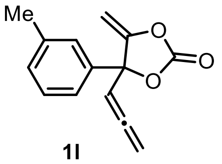

1l

5-methylene-4-(propa-1,2-dien-1-yl)-4-(m-tolyl)-1,3-dioxolan-2-one (1l): ^{1}H NMR (400 MHz, $CDCl_3$) δ 7.37-7.27 (m, 3H), 7.25-7.16 (m, 1H), 5.66 (t, *J* = 6.6 Hz, 1H), 5.08 (d, *J* = 6.6 Hz, 2H), 5.02 (d, *J* = 3.9 Hz, 1H), 4.41 (d, *J* = 3.9 Hz, 1H), 2.39 (s, 3H); ^{13}C NMR (100 MHz, $CDCl_3$) δ 208.3, 154.7, 151.1, 138.7, 137.7, 130.2, 128.7, 126.3, 122.9, 93.3, 90.4, 87.9, 81.0, 21.6; IR (neat, cm^{-1}) 3785, 1977, 1955, 1813, 1682, 1597,

1282, 1191, 1122, 1052, 1023, 851, 787, 760, 700; HRMS (ESI): *m/z*: $[M\text{-}CO_2\text{+}H]^+$ calcd for $C_{13}H_{13}O$: 185.0966, found: 185.0964.

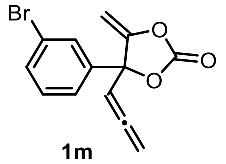

1m

4-(3-bromophenyl)-5-methylene-4-(propa-1,2-dien-1-yl)-1,3-dioxolan-2-one (1m): ^1H NMR (400 MHz, $CDCl_3$) δ 7.63 (s, 1H), 7.53 (d, *J* = 7.9 Hz, 1H), 7.43 (d, *J* = 7.9 Hz, 1H), 7.29 (t, *J* = 7.9 Hz, 1H), 5.62 (t, *J* = 6.5 Hz, 1H), 5.13-5.02 (m, 3H), 4.45 (d, *J* = 4.1 Hz, 1H); ^{13}C NMR (100 MHz, $CDCl_3$) δ 208.6, 154.1, 150.7, 139.9, 132.7, 130.4, 129.0, 124.6, 122.9, 93.1, 91.1, 87.2, 81.4; IR (neat, cm^{-1}) 1977, 1955, 1822, 1679, 1418, 1283, 1191, 1124, 1049, 1018, 851, 756, 692; HRMS (ESI): *m/z*: $[M\text{-}CO_2\text{+}H]^+$ calcd for $C_{12}H_{10}BrO$: 248.9915, found: 248.9909.

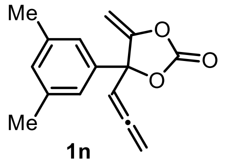

1n

4-(3,5-dimethylphenyl)-5-methylene-4-(propa-1,2-dien-1-yl)-1,3-dioxolan-2-one (1n): ^1H NMR (400 MHz, $CDCl_3$) δ 7.09 (s, 2H), 7.03 (s, 1H), 5.66 (t, *J* = 6.6 Hz, 1H), 5.09 (d, *J* = 6.6 Hz, 2H), 5.00 (d, *J* = 3.9 Hz, 1H), 4.39 (d, *J* = 3.9 Hz, 1H), 2.35 (s, 6H); ^{13}C NMR (100 MHz, $CDCl_3$) δ 208.3, 154.8, 151.2, 138.6, 137.8, 131.1, 123.5, 93.4, 90.2, 88.0, 80.9, 21.5; IR (neat, cm^{-1}) 3006, 2916, 1981, 1956, 1808, 1685, 1604, 1441, 1287, 1209, 1174, 1122, 1051, 1019, 849, 767, 720, 698; HRMS (ESI): *m/z*: $[M\text{-}CO_2\text{+}H]^+$ calcd for $C_{14}H_{15}O$: 199.1123, found: 199.1126.

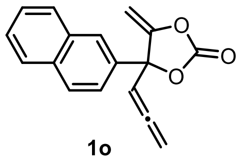

1o

5-methylene-4-(naphthalen-2-yl)-4-(propa-1,2-dien-1-yl)-1,3-dioxolan-2-one (1o): ^1H NMR (400 MHz, $CDCl_3$) δ 8.02 (d, *J* = 1.2 Hz, 1H), 7.97-7.83 (m, 3H), 7.64-7.50 (m, 3H), 5.78 (t, *J* = 6.6 Hz, 1H), 5.10 (dd, *J* = 5.1, 3.0 Hz, 3H), 4.50 (d, *J* = 4.0 Hz, 1H); ^{13}C NMR (100 MHz, $CDCl_3$) δ 208.5, 154.5, 151.1, 134.8, 133.5, 132.6, 129.0, 128.6, 127.7, 127.3, 126.9, 125.2, 123.3, 93.3, 90.7, 88.2, 81.1; IR (neat, cm^{-1}) 3059, 3020, 1976, 1955, 1815, 1682, 1357, 1281, 1188, 1116, 1050, 1018, 852, 816, 747; HRMS (ESI): *m/z*: $[M\text{-}CO_2\text{+}H]^+$ calcd for $C_{16}H_{13}O$: 221.0966, found: 221.0969.

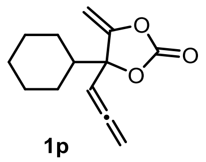

1p

4-cyclohexyl-5-methylene-4-(propa-1,2-dien-1-yl)-1,3-dioxolan-2-one (1p): ^{1}H NMR (400 MHz, $CDCl_3$) δ 5.44-5.27 (m, 1H), 5.09-4.95 (m, 2H), 4.92-4.81 (m, 1H), 4.28 (dd, *J* = 3.8, 1.2 Hz, 1H), 2.01-1.53 (m, 6H), 1.37-0.95 (m, 5H); ^{13}C NMR (100 MHz, $CDCl_3$) δ 207.3, 154.1, 151.5, 91.8, 89.2, 88.1, 80.3, 46.2, 26.1, 26.0, 25.94, 25.92, 25.91; IR (neat, cm^{-1}) 2932, 2857, 1958, 1817, 1680, 1451, 1285, 1205, 1178, 1033, 1017, 847, 763; HRMS (ESI): *m/z*: $[M\text{-}CO_2+H]^+$ calcd for $C_{12}H_{17}O$: 177.1279, found: 177.1276.

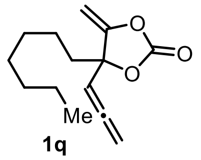

1q

4-heptyl-5-methylene-4-(propa-1,2-dien-1-yl)-1,3-dioxolan-2-one (1q): ^{1}H NMR (400 MHz, $CDCl_3$) δ 5.38 (t, *J* = 6.6 Hz, 1H), 5.06 (d, *J* = 6.4 Hz, 2H), 4.87 (d, *J* = 3.8 Hz, 1H), 4.31 (d, *J* = 3.8 Hz, 1H), 2.10-1.90 (m, 1H), 1.87-1.71 (m, 1H), 1.54-1.08 (m, 10H), 0.87 (t, *J* = 6.4 Hz, 3H); ^{13}C NMR (100 MHz, $CDCl_3$) δ 207.6, 155.4, 151.4, 93.1, 87.5, 87.1, 80.6, 39.1, 31.8, 29.3, 29.1, 22.9, 22.7, 14.2; IR (neat, cm^{-1}) 3781, 2927, 2857, 1956, 1822, 1682, 1284, 1107, 1021, 849, 763; HRMS (ESI): *m/z*: $[M\text{-}CO_2+H]^+$ calcd for $C_{13}H_{21}O$: 193.1592, found: 193.1601.

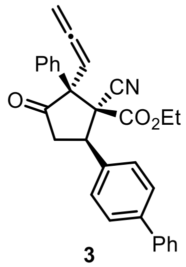

3

ethyl (1S,2S,5R)-5-([1,1'-biphenyl]-4-yl)-1-cyano-3-oxo-2-phenyl-2-(propa-1,2-dien-1-yl)cyclopentane-1-carboxylate (3): white solid; 31.3 mg, 70% yield; 95% *ee*; $[\alpha]_D^{20}$ = 70.4 (*c* = 0.115, $CHCl_3$); m.p. 128-130 °C; ^{1}H NMR (400 MHz, $CDCl_3$) δ 7.68-7.56 (m, 6H), 7.52 (d, *J* = 8.3 Hz, 2H), 7.46 (t, *J* = 7.5 Hz, 2H), 7.41-7.27 (m, 4H), 5.89 (t, *J* = 6.7 Hz, 1H), 5.21-5.03 (m, 2H), 4.30 (t, *J* = 11.5, 9.8 Hz, 1H), 3.80-3.58 (m, 2H), 3.43 (dd, *J* = 18.9, 11.7 Hz, 1H), 2.95 (dd, *J* = 18.9, 9.6 Hz, 1H), 0.71 (t, *J* = 7.1 Hz, 3H); ^{13}C NMR (100 MHz, $CDCl_3$) δ 207.9, 205.8, 165.7, 141.6, 140.3, 136.1, 133.3, 129.0, 128.7, 128.4, 128.23, 128.16, 127.8, 127.6, 127.1, 117.4, 91.4, 80.5, 64.7, 63.2, 62.9, 45.9,

38.5, 13.4; IR (neat, cm^{-1}) 2921, 2851, 1955, 1748, 1730, 1485, 1446, 1235, 1138, 848, 763, 698; HRMS (ESI): *m/z*: $[M+Na]^+$ calcd for $C_{30}H_{25}NO_3Na$: 470.1732, found: 470.1729.

The *ee* was determined by HPLC analysis: CHIRALPAK ODH (4.6 mm i.d. × 250 mm); hexane/2-propanol = 90/10; flow rate 0.5 mL/min; 35 °C; 220 nm; retention time: 28.9 min (major) and 33.6 min (minor).

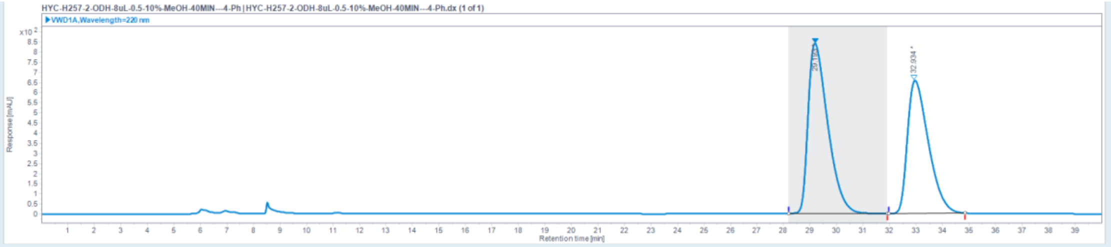

Injection Results

Peaks Summary

#	RT (min)	Area%	Start time (min)	End time (min)	Symmetry
1	29.193	54.573	28.182	31.918	0.53763
2	32.934	45.427	31.962	34.836	0.50977

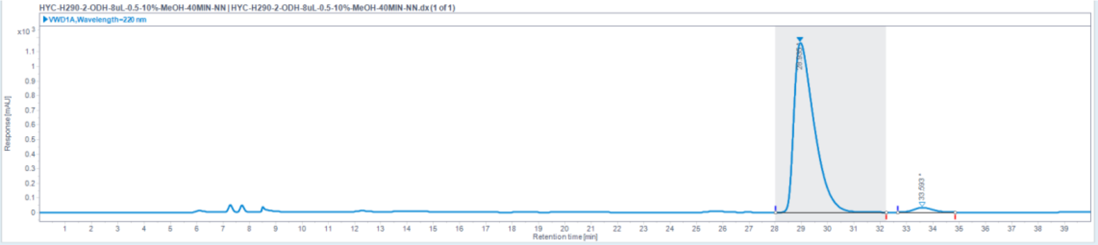

Injection Results

Peaks Summary

#	RT (min)	Area%	Start time (min)	End time (min)	Symmetry
1	28.935	97.451	28.016	32.213	0.42512
2	33.593	2.549	32.650	34.833	0.83600

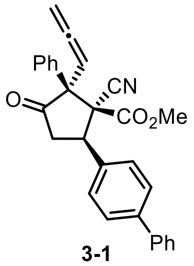

3-1

methyl (1S,2S,5R)-5-([1,1'-biphenyl]-4-yl)-1-cyano-3-oxo-2-phenyl-2-(propa-1,2-dien-1-yl)cyclopentane-1-carboxylate (3-1): white solid; 30.8 mg, 71% yield; 93% *ee*; $[\alpha]_D^{20}$ = 32.5 (c = 0.120, $CHCl_3$); m.p. 132-133 °C; ^1H NMR (400 MHz, $CDCl_3$) δ 7.66-7.56 (m, 6H), 7.51-7.42 (m, 4H), 7.40-7.27 (m, 4H), 5.88 (t, *J* = 6.7 Hz, 1H), 5.12 (qd, *J* = 12.1, 6.7 Hz, 2H), 4.29 (dd, *J* = 11.5, 9.8 Hz, 1H), 3.41 (dd, *J* = 18.9, 11.7 Hz, 1H), 3.22 (s, 3H), 2.95 (dd, *J* = 18.9, 9.6 Hz, 1H); ^{13}C NMR (100 MHz, CDCl3) δ 207.9, 205.7, 166.2, 141.7, 140.2, 136.0, 133.3, 129.0, 128.6, 128.5, 128.3, 128.1, 127.8, 127.7, 127.1, 117.2, 91.3, 80.7, 64.8, 63.3, 53.3, 46.0, 38.6; IR (neat, cm-1) 3059, 2953, 1951, 1748, 1488, 1320, 1243, 1107, 844, 763, 731, 697, 576; HRMS (ESI): m/z: $[M+Na]^+$ calcd for $C_{29}H_{23}NO_3Na$: 456.1576, found: 456.1574.

The *ee* was determined by HPLC analysis: CHIRALPAK ODH (4.6 mm i.d. × 250 mm); hexane/2-propanol = 90/10; flow rate 0.5 mL/min; 35 °C; 220 nm; retention time: 35.5 min (major) and 39.7 min (minor).

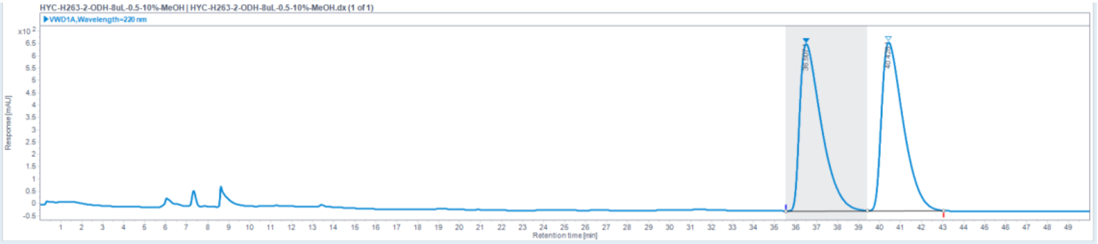

Injection Results

Peaks Summary

#	RT (min)	Area%	Start time (min)	End time (min)	Symmetry
1	36.507	49.823	35.540	39.402	0.41778
2	40.428	50.177	39.402	43.029	0.47162

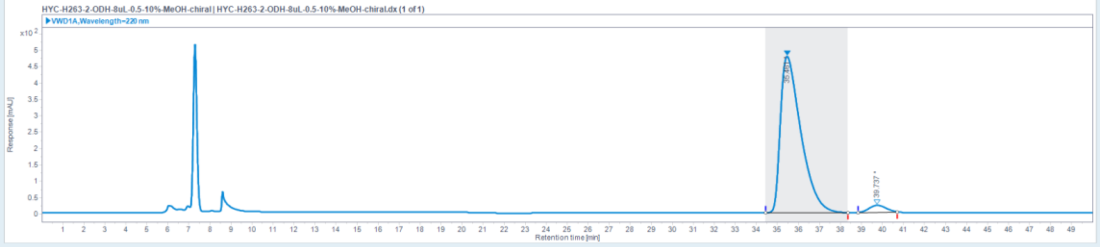

Injection Results

Peaks Summary

#	RT (min)	Area%	Start time (min)	End time (min)	Symmetry
1	35.461	96.532	34.424	38.341	0.47265
2	39.737	3.468	38.823	40.685	0.92598

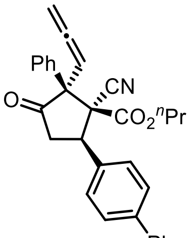

3-2

propyl (1S,2S,5R)-5-([1,1'-biphenyl]-4-yl)-1-cyano-3-oxo-2-phenyl-2-(propa-1,2-dien-1-yl)cyclopentane-1-carboxylate (3-2): white solid; 31.5 mg, 68% yield; 95% *ee*; $[\alpha]_D^{20}$ = 35.7 (*c* = 0.175, $CHCl_3$); m.p. 95-96 °C; ^{1}H NMR (400 MHz, $CDCl_3$) δ 7.66-7.55 (m, 6H), 7.51 (d, *J* = 8.3 Hz, 2H), 7.48-7.42 (m, 2H), 7.40-7.27 (m, 4H), 5.89 (t, *J* = 6.7 Hz, 1H), 5.12 (qd, *J* = 12.1, 6.7 Hz, 2H), 4.30 (dd, *J* = 11.5, 9.8 Hz, 1H), 3.66-3.49 (m, 2H), 3.43 (dd, *J* = 18.9, 11.6 Hz, 1H), 2.94 (dd, *J* = 18.9, 9.6 Hz, 1H), 1.19-1.02 (m, 2H), 0.46 (t, *J* = 7.4 Hz, 3H); ^{13}C NMR (100 MHz, $CDCl_3$) δ 207.9, 205.8, 165.8, 141.7, 140.3, 136.1, 133.3, 129.0, 128.7, 128.5, 128.3, 128.2, 127.8, 127.7, 127.1, 117.3, 91.4, 80.6, 68.4, 64.7, 63.3, 46.0, 38.5, 21.3, 9.9; IR (neat, cm^{-1}) 3060, 2969, 1952, 1752, 1735, 1489, 1319, 1229, 909, 846, 763, 731, 699; HRMS (ESI): *m/z*: $[M+Na]^+$ calcd for $C_{31}H_{27}NO_3Na$: 484.1889, found: 484.1893.

The *ee* was determined by HPLC analysis: CHIRALPAK ODH (4.6 mm i.d. × 250 mm); hexane/2-propanol = 90/10; flow rate 0.5 mL/min; 35 °C; 220 nm; retention time: 26.9 min (major) and 29.9 min (minor).

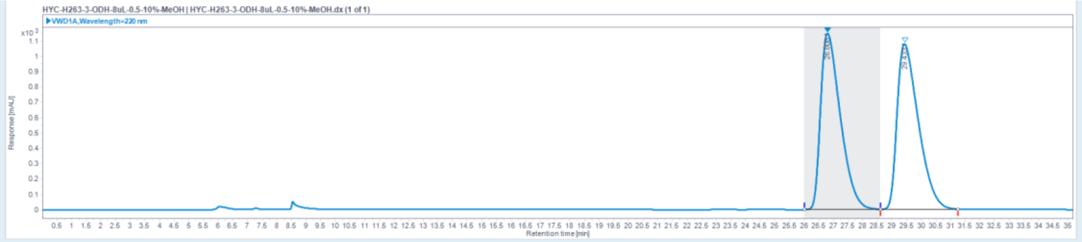

Injection Results

Peaks Summary

#	RT (min)	Area%	Start time (min)	End time (min)	Symmetry
1	26.808	49.718	25.997	28.594	0.56476
2	29.433	50.282	28.594	31.237	0.54700

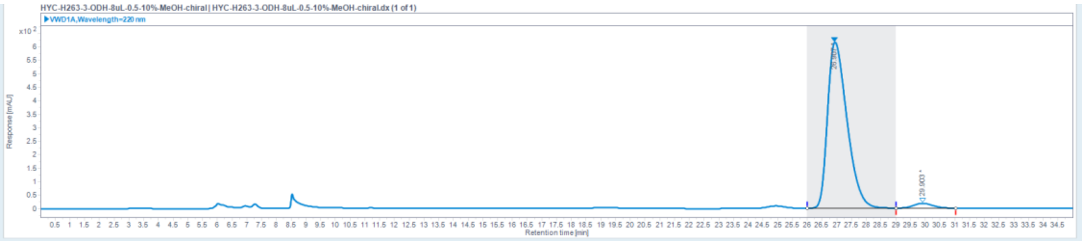

Injection Results

Peaks Summary

#	RT (min)	Area%	Start time (min)	End time (min)	Symmetry
1	26.907	97.229	25.985	28.997	0.53552
2	29.903	2.771	28.997	31.020	0.87005

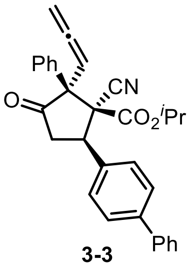

3-3

isopropyl (1S,2S,5R)-5-([1,1'-biphenyl]-4-yl)-1-cyano-3-oxo-2-phenyl-2-(propa-1,2-dien-1-yl)cyclopentane-1-carboxylate (3-3): white solid; 29.1 mg, 63% yield; 97% *ee*; $[\alpha]_D^{20}$ = 40.7 (*c* = 0.145, $CHCl_3$); m.p. 110-112 °C; ^{1}H NMR (400 MHz, $CDCl_3$) δ 7.66-7.56 (m, 6H), 7.52 (d, *J* = 8.2 Hz, 2H), 7.49-7.42 (m, 2H), 7.41-7.26 (m, 4H), 5.87 (t, *J* = 6.7 Hz, 1H), 5.11 (qd, *J* = 12.0, 6.7 Hz, 2H), 4.49 (dt, *J* = 12.5, 6.3 Hz, 1H), 4.29 (t, *J* = 10.6 Hz, 1H), 3.43 (dd, *J* = 18.9, 11.6 Hz, 1H), 2.94 (dd, *J* = 18.9, 9.6 Hz, 1H), 0.69 (t, *J* = 6.4 Hz, 6H); ^{13}C NMR (100 MHz, $CDCl_3$) δ 207.8, 205.9, 165.3, 141.6, 140.4, 136.3, 133.4, 129.0, 128.7, 128.4, 128.17, 128.16, 127.8, 127.6, 127.1, 117.5, 91.5, 80.5, 71.3, 64.5, 63.1, 45.8, 38.5, 20.91, 20.89; IR (neat, cm^{-1}) 2983, 1951, 1754, 1730, 1489, 1314, 1102, 910, 847, 699; HRMS (ESI): *m/z*: $[M+Na]^+$ calcd for $C_{31}H_{27}NO_3Na$: 484.1889, found: 484.1886.

The *ee* was determined by HPLC analysis: CHIRALPAK ODH (4.6 mm i.d. × 250 mm); hexane/2-propanol = 90/10; flow rate 0.5 mL/min; 35 °C; 220 nm; retention time: 23.2 min (major) and 27.8 min (minor).

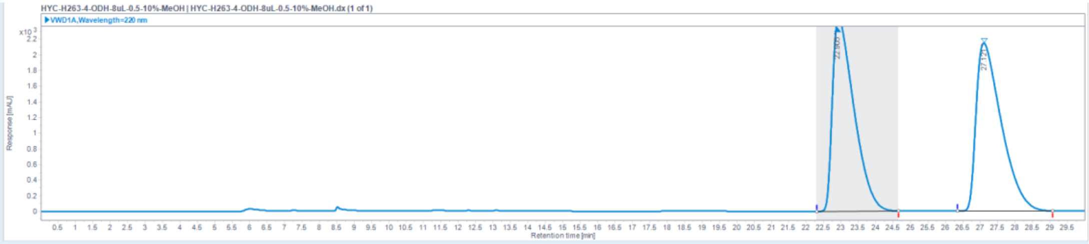

Injection Results

Peaks | Summary

#	RT (min)	Area%	Start time (min)	End time (min)	Symmetry
1	22.905	49.567	22.297	24.641	0.35574
2	27.121	50.433	26.336	29.072	0.46632

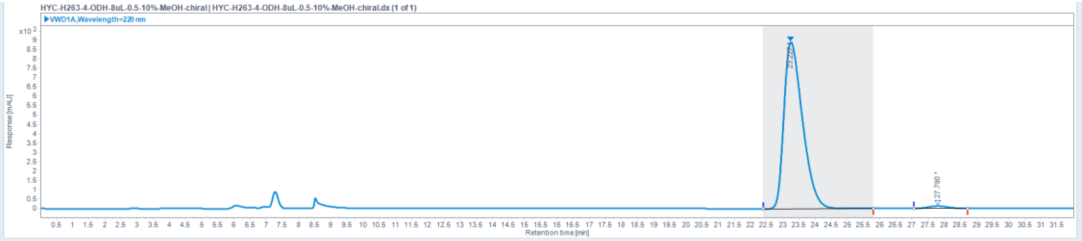

Injection Results

Peaks | Summary

#	RT (min)	Area%	Start time (min)	End time (min)	Symmetry
1	23.227	98.407	22.392	25.793	0.54420
2	27.790	1.593	27.046	28.711	0.87112

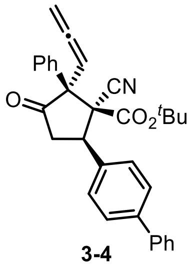

3-4

tert-butyl (1S,2S,5R)-5-([1,1'-biphenyl]-4-yl)-1-cyano-3-oxo-2-phenyl-2-(propa-1,2-dien-1-yl)cyclopentane-1-carboxylate (3-4): white solid; 25.2 mg, 53% yield; 98% *ee*; $[\alpha]_D^{20}$ = 54.3 (*c* = 0.110, $CHCl_3$); m.p. 116-118 °C; ^1H NMR (400 MHz, $CDCl_3$) δ 7.71-7.52 (m, 8H), 7.46 (t, *J* = 7.5 Hz, 2H), 7.41-7.27 (m, 4H), 5.89 (t, *J* = 6.7 Hz, 1H), 5.09 (qd, *J* = 12.0, 6.7 Hz, 2H), 4.26 (t, *J* = 10.6 Hz, 1H), 3.45 (dd, *J* = 18.8, 11.6 Hz, 1H), 2.92 (dd, *J* = 18.8, 9.7 Hz, 1H), 0.88 (s, 9H); ^{13}C NMR (100 MHz, $CDCl_3$) (one carbon signal was overlapped) δ 207.8, 206.3, 164.7, 141.4, 140.4, 136.3, 133.4, 129.0, 128.9, 128.4, 128.1, 127.7, 127.5, 127.1, 118.0, 91.6, 85.2, 80.4, 64.4, 63.3, 45.6, 38.5, 27.2; IR (neat, cm^{-1}) 2981, 1952, 1751, 1726, 1488, 1371, 1324, 1255, 1149, 842, 734, 697; HRMS (ESI): *m/z*: $[M+H]^+$ calcd for $C_{32}H_{30}NO_3$: 476.2226, found: 476.2226.

The *ee* was determined by HPLC analysis: CHIRALPAK ADH (4.6 mm i.d. × 250 mm); hexane/2-propanol = 90/10; flow rate 0.5 mL/min; 35 °C; 220 nm; retention time: 17.6 min (major) and 15.0 min (minor).

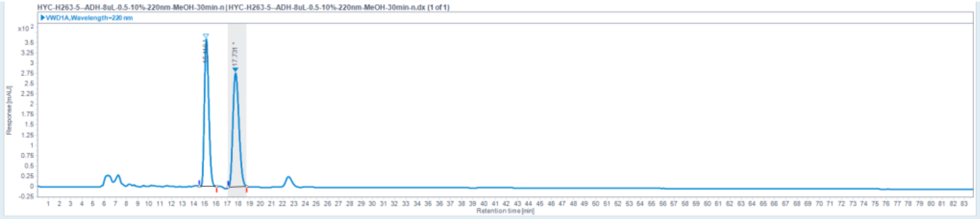

Injection Results

Peaks | Summary

#	RT (min)	Area%	Start time (min)	End time (min)	Symmetry
1	15.116	48.224	14.497	16.046	0.81240
2	17.731	51.776	17.107	18.770	0.79819

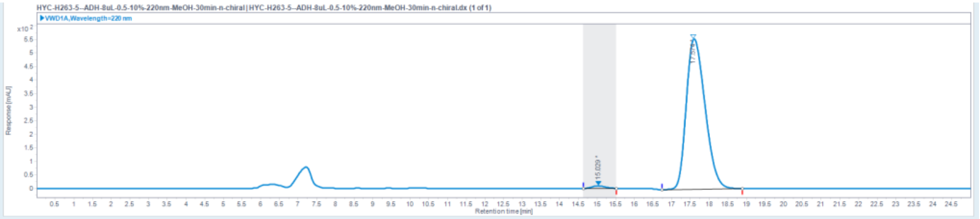

Injection Results

Peaks | Summary

#	RT (min)	Area%	Start time (min)	End time (min)	Symmetry
1	15.029	1.184	14.628	15.511	0.87406
2	17.574	98.816	16.746	18.882	0.61805

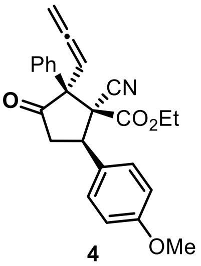

4

ethyl (1S,2S,5R)-1-cyano-5-(4-methoxyphenyl)-3-oxo-2-phenyl-2-(propa-1,2-dien-1-yl)cyclopentane-1-carboxylate (4): white solid; 25.3 mg, 63% yield; 95% *ee*; $[\alpha]_D^{20}$ = 24.2 (*c* = 0.065, $CHCl_3$); m.p. 86-87 ^oC; ^1H NMR (400 MHz, $CDCl_3$) δ 7.58 (d, *J* = 7.6 Hz, 2H), 7.41-7.27 (m, 5H), 6.90 (d, *J* = 8.6 Hz, 2H), 5.85 (t, *J* = 6.7 Hz, 1H), 5.20-4.95 (m, 2H), 4.26-4.10 (m, 1H), 3.80 (s, 3H), 3.77-3.57 (m, 2H), 3.33 (dd, *J* = 18.9, 11.7 Hz, 1H), 2.88 (dd, *J* = 18.9, 9.6 Hz, 1H), 0.72 (t, *J* = 7.1 Hz, 3H); ^{13}C NMR (100 MHz, $CDCl_3$) δ 207.8, 206.0, 165.8, 159.9, 136.2, 129.4, 128.4, 128.2, 128.1, 126.1, 117.5, 114.3, 91.4, 80.4, 64.6, 63.4, 62.8, 55.4, 45.6, 38.7, 13.5; IR (neat, cm^{-1}) 2962, 2933, 1952, 1750, 1736, 1611, 1514, 1300, 1257, 1233, 1031, 837, 733, 699; HRMS (ESI): *m/z*: $[M+Na]^+$ calcd for $C_{25}H_{23}NO_4Na$: 424.1525, found: 424.1524.

The *ee* was determined by HPLC analysis: CHIRALPAK ADH (4.6 mm i.d. × 250 mm); hexane/2-propanol = 90/10; flow rate 0.5 mL/min; 35 ^oC; 220 nm; retention time: 22.1 min (major) and 27.4 min (minor).

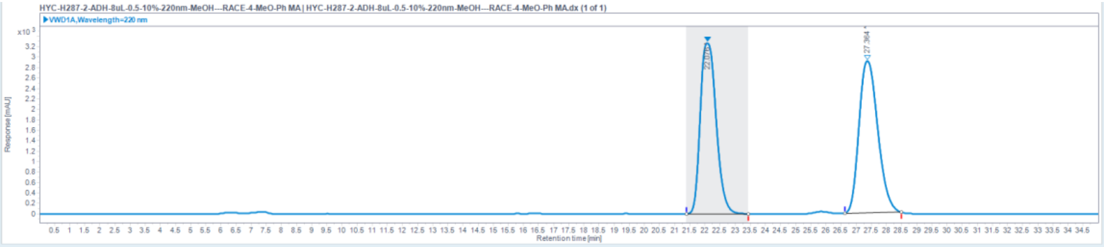

Injection Results

Peaks | Summary

#	RT (min)	Area%	Start time (min)	End time (min)	Symmetry
1	22.076	48.767	21.378	23.423	0.79097
2	27.364	51.233	26.615	28.480	0.78558

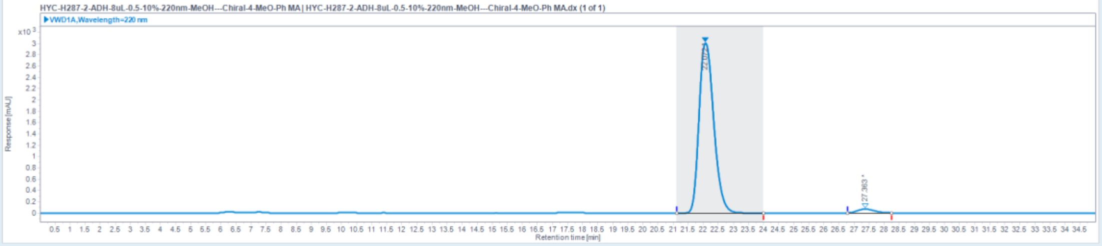

Injection Results

Peaks | Summary

#	RT (min)	Area%	Start time (min)	End time (min)	Symmetry
1	22.072	97.498	21.108	23.985	0.78830
2	27.363	2.502	26.772	28.233	0.79839

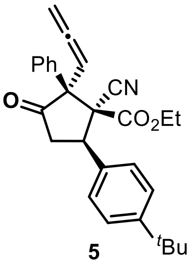

5

ethyl (1S,2S,5R)-5-(4-(tert-butyl)phenyl)-1-cyano-3-oxo-2-phenyl-2-(propa-1,2-dien-1-yl)cyclopentane-1-carboxylate (5): yellow solid; 30.8 mg, 72% yield; 97% *ee*; $[\alpha]_D^{20}$ = 19.7 (*c* = 0.175, $CHCl_3$); m.p. 89-91 ^oC; ^1H NMR (400 MHz, $CDCl_3$) δ 7.59 (d, *J* = 7.4 Hz, 2H), 7.43-7.26 (m, 7H), 5.85 (t, *J* = 6.7 Hz, 1H), 5.19-4.98 (m, 2H), 4.22 (dd, *J* = 11.5, 9.8 Hz, 1H), 3.77-3.56 (m, 2H), 3.36 (dd, *J* = 18.9, 11.7 Hz, 1H), 2.88 (dd, *J* = 18.9, 9.6 Hz, 1H), 1.30 (s, 9H), 0.67 (t, *J* = 7.1 Hz, 3H); ^{13}C NMR (100 MHz, $CDCl_3$) δ 207.9, 206.0, 165.7, 151.9, 136.3, 131.2, 128.4, 128.2, 128.1, 127.9, 125.9, 117.5, 91.5, 80.4, 64.6, 63.2, 62.7, 45.8, 38.5, 34.7, 31.4, 13.4; IR (neat, cm^{-1}) 2960, 2926, 2858, 1944, 1757, 1735, 1218, 1105, 985, 841, 761, 699; HRMS (ESI): *m/z*: $[M+Na]^+$ calcd for $C_{28}H_{29}NO_3Na$: 450.2045, found: 450.2044.

The *ee* was determined by HPLC analysis: CHIRALPAK ADH (4.6 mm i.d. × 250 mm); hexane/2-propanol = 90/10; flow rate 0.5 mL/min; 35 ^oC; 220 nm; retention time: 15.6 min (major) and 12.0 min (minor).

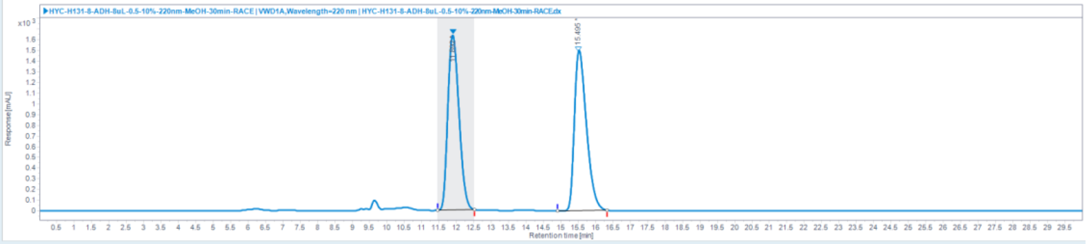

Injection Results

Peaks | Summary

#	RT (min)	Area%	Start time (min)	End time (min)	Symmetry
1	11.892	50.303	11.465	12.505	0.82391
2	15.495	49.697	14.910	16.319	0.49333

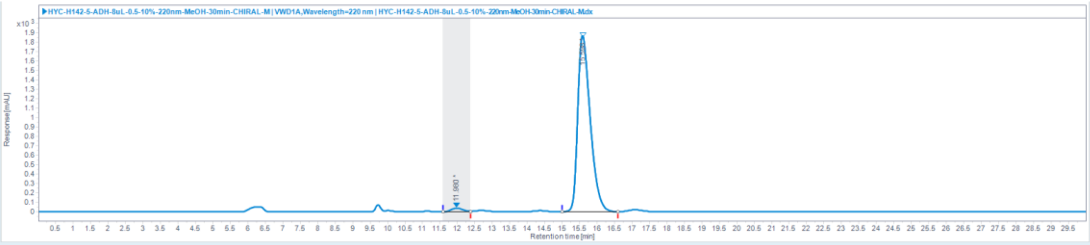

Injection Results

Peaks | Summary

#	RT (min)	Area%	Start time (min)	End time (min)	Symmetry
1	11.980	1.638	11.579	12.369	0.97830
2	15.598	98.362	14.989	16.604	0.71339

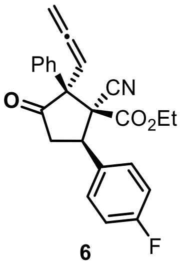

6

ethyl (1S,2S,5R)-1-cyano-5-(4-fluorophenyl)-3-oxo-2-phenyl-2-(propa-1,2-dien-1-yl)cyclopentane-1-carboxylate (6): red oil; 25.3 mg, 65% yield; 95% *ee*; $[\alpha]_D^{20}$ = 12.4 (*c* = 0.185, $CHCl_3$); ^{1}H NMR (400 MHz, $CDCl_3$) δ 7.51 (d, *J* = 7.7 Hz, 2H), 7.38-7.31 (m, 2H), 7.30-7.17 (m, 3H), 7.00 (t, *J* = 8.4 Hz, 2H), 5.78 (t, *J* = 6.7 Hz, 1H), 5.11-4.93 (m, 2H), 4.15 (t, *J* = 10.6 Hz, 1H), 3.69-3.49 (m, 2H), 3.26 (dd, *J* = 18.8, 11.7 Hz, 1H), 2.83 (dd, *J* = 18.9, 9.6 Hz, 1H), 0.64 (t, *J* = 7.1 Hz, 3H); ^{13}C NMR (100 MHz, $CDCl_3$) δ 207.8, 205.4, 165.6, 162.9 (d, *J* = 247.0 Hz), 136.0, 130.1 (d, *J* = 3.0 Hz), 130.0 (d, *J* = 8.0 Hz), 128.4, 128.2, 128.1, 117.2, 115.9 (d, *J* = 21.0 Hz), 91.3, 80.5, 64.6, 63.2, 62.9, 45.4, 38.6, 13.4; ^{19}F NMR (376 MHz, $CDCl_3$) δ -113.0 (s); IR (neat, cm^{-1}) 2986, 2937, 1951, 1751, 1732, 1604, 1511, 1227, 1100, 993, 841, 754, 698; HRMS (ESI): *m/z*: $[M+Na]^+$ calcd for $C_{24}H_{20}NO_3NaF$: 412.1325, found: 412.1318.

The *ee* was determined by HPLC analysis: CHIRALPAK ADH (4.6 mm i.d. 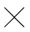 250 mm); hexane/2-propanol = 90/10; flow rate 0.5 mL/min; 35 ^oC; 220 nm; retention time: 15.6 min (major) and 22.2 min (minor).

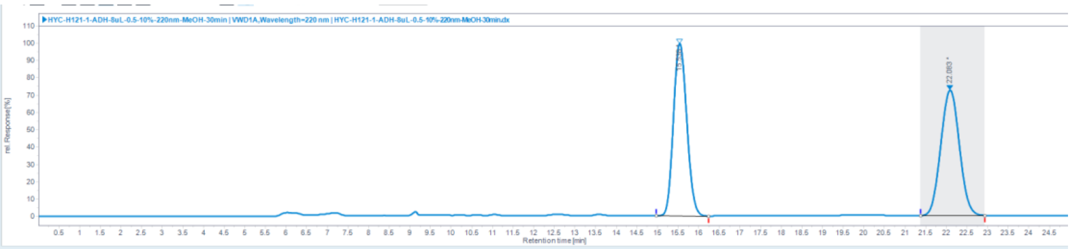

Injection Results

#	RT (min)	Area%	Start time (min)	End time (min)	Symmetry
1	15.538	49.600	14.979	16.247	0.89208
2	22.083	50.400	21.368	22.926	0.96085

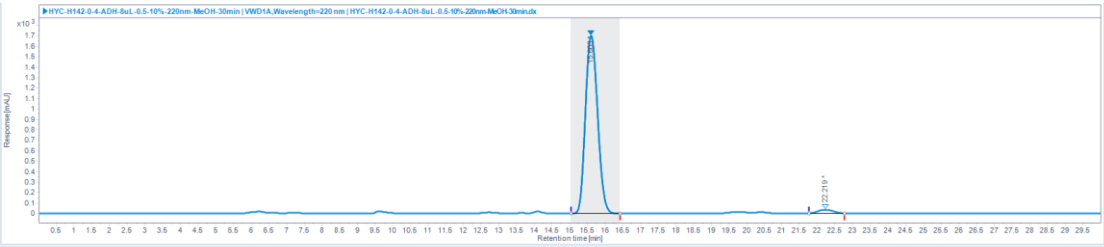

Injection Results

#	RT (min)	Area%	Start time (min)	End time (min)	Symmetry
1	15.603	97.507	15.028	16.434	0.88095
2	22.219	2.493	21.752	22.754	0.93226

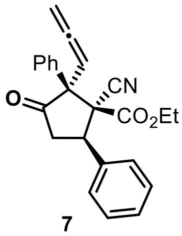

7

ethyl (1S,2S,5R)-1-cyano-3-oxo-2,5-diphenyl-2-(propa-1,2-dien-1-yl)cyclopentane-1-carboxylate (7): yellow oil; 28.9mg, 78% yield; 94% *ee*; $[\alpha]_D^{20}$ = 10.5 (c = 0.19, $CHCl_3$); ^{1}H NMR (400 MHz, $CDCl_3$) δ 7.52 (d, J = 7.5 Hz, 2H), 7.42-7.17 (m, 8H), 5.79 (t, J = 6.7 Hz, 1H), 5.14-4.91 (m, 2H), 4.17 (t, J = 12.0 Hz, 1H), 3.68-3.44 (m, 2H), 3.32 (dd, J = 18.9, 11.7 Hz, 1H), 2.83 (dd, J = 18.9, 9.6 Hz, 1H), 0.62 (t, J = 7.1 Hz, 3H); ^{13}C NMR (100 MHz, $CDCl_3$) (one carbon signal was overlapped) δ 207.9, 205.9, 165.7, 136.2, 134.3, 129.0, 128.8, 128.5, 128.24, 128.17, 117.4, 91.5, 80.5, 64.7, 63.2, 62.8, 46.2, 38.5, 13.4; IR (neat, cm^{-1}) 3062, 2927, 2854, 1952, 1749, 1734, 1498, 1449, 1234, 854, 754, 734, 697; HRMS (ESI): m/z: $[M+Na]^+$ calcd for $C_{24}H_{21}NO_3Na$: 394.1419, found: 394.1411.

The *ee* was determined by HPLC analysis: CHIRALPAK ADH (4.6 mm i.d. × 250 mm); hexane/2-propanol = 90/10; flow rate 0.5 mL/min; 35 °C; 220 nm; retention time: 14.2 min (major) and 18.1 min (minor).

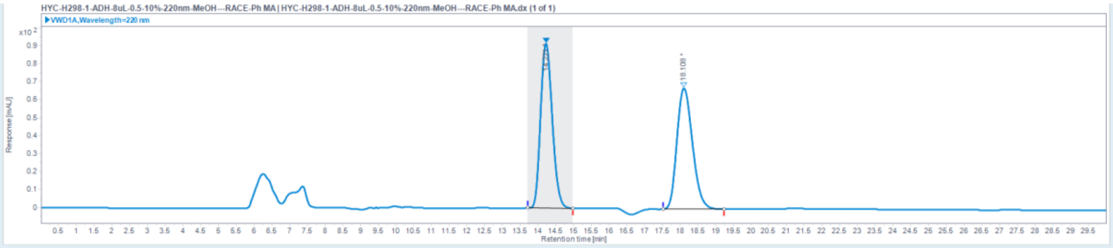

Injection Results

Peaks Summary

#	RT (min)	Area%	Start time (min)	End time (min)	Symmetry
1	14.221	50.508	13.699	14.971	0.85576
2	18.108	49.492	17.514	19.244	0.81485

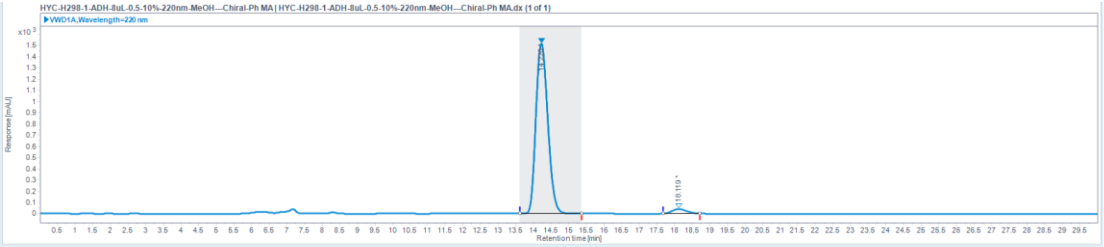

Injection Results

Peaks Summary

#	RT (min)	Area%	Start time (min)	End time (min)	Symmetry
1	14.220	96.895	13.602	15.357	0.85103
2	18.119	3.105	17.667	18.707	0.85389

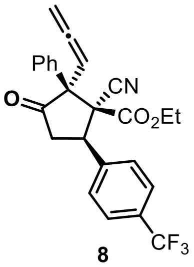

8

ethyl (1S,2S,5R)-1-cyano-3-oxo-2-phenyl-2-(propa-1,2-dien-1-yl)-5-(4-(trifluoromethyl)phenyl)cyclopentane-1-carboxylate (8): white solid; 18.0 mg, 41% yield; 90% *ee*; $[\alpha]_D^{20}$ = 26. 7 (*c* = 0.105, $CHCl_3$); m.p. 97-98 ^oC; ^1H NMR (400 MHz, $CDCl_3$) δ 7.65 (d, *J* = 8.2 Hz, 2H), 7.57 (d, *J* = 8.2 Hz, 4H), 7.38-7.28 (m, 3H), 5.87 (t, *J* = 6.7 Hz, 1H), 5.21-5.00 (m, 2H), 4.30 (t, *J* = 10.6 Hz, 1H), 3.76-3.55 (m, 2H), 3.40 (dd, *J* = 18.8, 11.6 Hz, 1H), 2.95 (dd, *J* = 18.8, 9.6 Hz, 1H), 0.68 (t, *J* = 7.1 Hz, 3H); ^{13}C NMR (100 MHz, $CDCl_3$) δ 207.8, 205.1, 165.5, 138.6, 135.8, 131.0 (q, *J* = 32.0 Hz), 128.8, 128.5, 128.4, 128.2, 125.9 (q, *J* = 4.0 Hz), 123.9 (q, *J* = 270.0 Hz), 117.0, 91.2, 80.7, 64.7, 63.1, 63.0, 45.7, 38.3, 13.3; ^{19}F NMR (376 MHz, $CDCl_3$) δ -62.7 (s); IR (neat, cm^{-1}) 2927, 2855, 1952, 1753, 1735, 1620, 1325, 1234, 1168, 1126, 1112, 1069, 849, 736, 699; HRMS (ESI): *m/z*: $[M+Na]^+$ calcd for $C_{25}H_{21}NO_3NaF_3$: 440.1474, found: 440.1475.

The *ee* was determined by HPLC analysis: CHIRALPAK ADH (4.6 mm i.d. × 250 mm); hexane/2-propanol = 90/10; flow rate 0.5 mL/min; 35 ^oC; 220 nm; retention time: 16.6 min (major) and 18.8 min (minor).

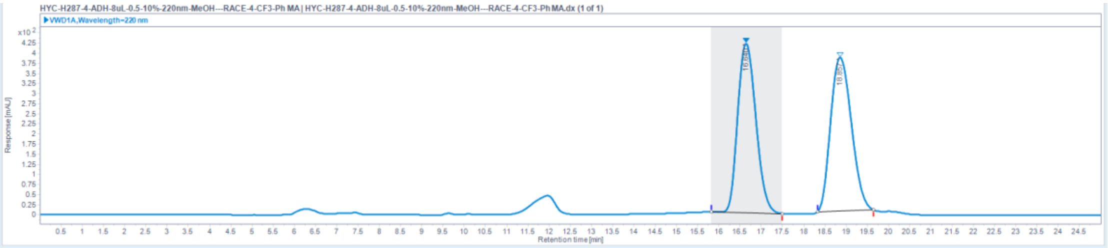

Injection Results

Peaks Summary

#	RT (min)	Area%	Start time (min)	End time (min)	Symmetry
1	16.640	49.125	15.816	17.485	0.83317
2	18.857	50.875	18.320	19.636	0.82815

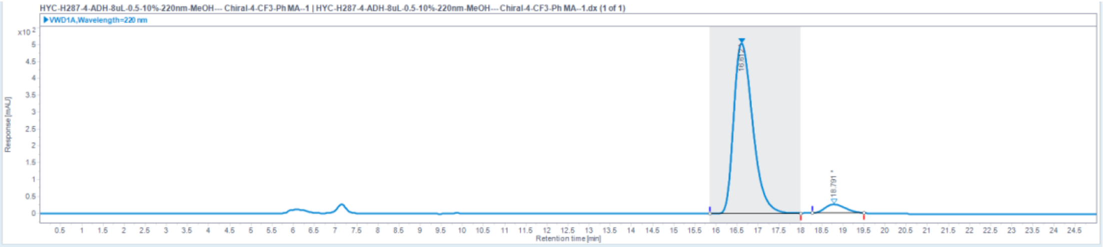

Injection Results

Peaks Summary

#	RT (min)	Area%	Start time (min)	End time (min)	Symmetry
1	16.612	94.852	15.848	18.000	0.71941
2	18.791	5.148	18.272	19.493	0.78559

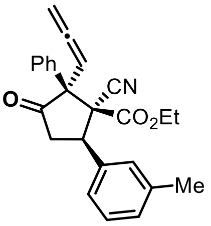

9

ethyl (1S,2S,5R)-1-cyano-3-oxo-2-phenyl-2-(propa-1,2-dien-1-yl)-5-(m-tolyl)cyclo-pentane-1-carboxylate (9): white solid; 30.0 mg, 78% yield; 95% *ee*; $[\alpha]_D^{20}$ = 16.2 (c = 0.105, $CHCl_3$); m.p. 76-78 ^oC; ^1H NMR (400 MHz, $CDCl_3$) δ 7.59 (d, J = 7.5 Hz, 2H), 7.33 (t, J = 7.4 Hz, 2H), 7.28 (d, J = 7.0 Hz, 1H), 7.22 (t, J = 8.5 Hz, 3H), 7.15 (d, J = 7.3 Hz, 1H), 5.85 (t, J = 6.7 Hz, 1H), 5.16-4.99 (m, 2H), 4.20 (dd, J = 11.4, 9.9 Hz, 1H), 3.77-3.57 (m, 2H), 3.36 (dd, J = 18.9, 11.7 Hz, 1H), 2.89 (dd, J = 18.9, 9.6 Hz, 1H), 2.36 (s, 3H), 0.72 (t, J = 7.1 Hz, 3H); ^{13}C NMR (100 MHz, $CDCl_3$) δ 207.8, 206.0, 165.7, 138.7, 136.2, 134.2, 129.5, 129.0, 128.9, 128.4, 128.20, 128.15, 125.2, 117.3, 91.5, 80.5, 64.6, 63.1, 62.8, 46.1, 38.6, 21.6, 13.4; IR (neat, cm^{-1}) 2962, 2925, 2854, 1952, 1750, 1735, 1606, 1447, 1228, 1096, 1032, 1014, 854, 794, 698; HRMS (ESI): m/z: $[M+Na]^+$ calcd for $C_{25}H_{23}NO_3Na$: 408.1576, found: 408.1571.

The *ee* was determined by HPLC analysis: CHIRALPAK ADH (4.6 mm i.d. × 250 mm); hexane/2-propanol = 90/10; flow rate 0.5 mL/min; 35 ^oC; 220 nm; retention time: 12.4 min (major) and 15.3 min (minor).

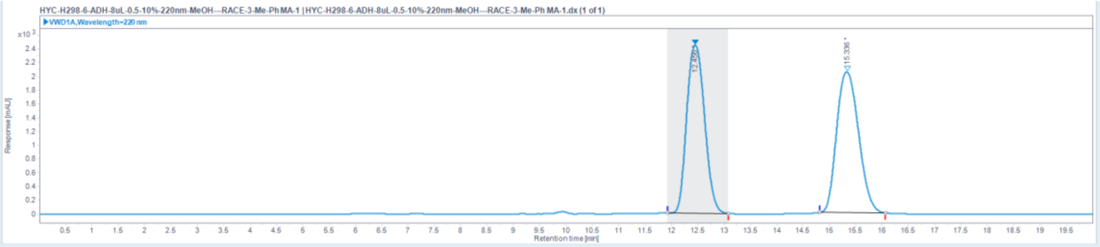

Injection Results

Peaks Summary

#	RT (min)	Area%	Start time (min)	End time (min)	Symmetry
1	12.456	49.320	11.919	13.075	0.88421
2	15.336	50.680	14.809	16.055	0.84455

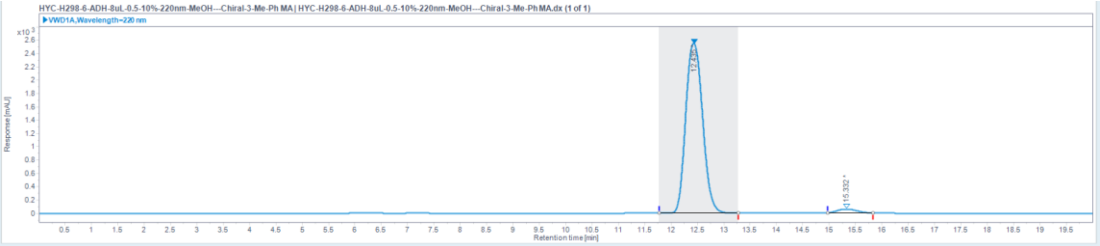

Injection Results

Peaks Summary

#	RT (min)	Area%	Start time (min)	End time (min)	Symmetry
1	12.435	97.535	11.767	13.269	0.89125
2	15.332	2.465	14.965	15.825	0.87035

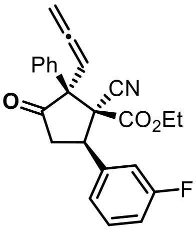

10

ethyl (1S,2S,5R)-1-cyano-5-(3-fluorophenyl)-3-oxo-2-phenyl-2-(propa-1,2-dien-1-yl)cyclopentane-1-carboxylate (10): white solid; 25.3 mg, 65% yield; 92% *ee*; $[\alpha]_D^{20}$ = 29.3 (*c* = 0.100, $CHCl_3$); m.p. 78-79 ^oC; ^1H NMR (400 MHz, $CDCl_3$) δ 7.57 (d, *J* = 7.5 Hz, 2H), 7.40-7.22 (m, 5H), 7.13 (d, *J* = 10.0 Hz, 1H), 7.04 (dd, *J* = 19.2, 10.9 Hz, 1H), 5.85 (t, *J* = 6.7 Hz, 1H), 5.21-5.01 (m, 2H), 4.29-4.15 (m, 1H), 3.81-3.58 (m, 2H), 3.33 (dd, *J* = 18.9, 11.6 Hz, 1H), 2.92 (dd, *J* = 18.9, 9.6 Hz, 1H), 0.73 (t, *J* = 7.1 Hz, 3H); ^{13}C NMR (100 MHz, $CDCl_3$) δ 207.8, 205.3, 165.6, 164.4 (d, *J* = 136.0 Hz), 137.1 (d, *J* = 7.2 Hz), 135.9, 130.6 (d, *J* = 8.3 Hz), 128.5, 128.4, 128.2, 124.2 (d, *J* = 2.9 Hz), 117.1, 115.8 (d, *J* = 21.0 Hz), 115.2 (d, *J* = 22.0 Hz), 91.3, 80.7, 64.7, 63.04, 62.99, 45.7, 38.6, 13.4; ^{19}F NMR (376 MHz, $CDCl_3$) δ -111.6 (s); IR (neat, cm^{-1}) 2962, 2926, 2854, 1952, 1752, 1734, 1590, 1490, 1448, 1317, 1256, 1148, 1016, 855, 795, 695; HRMS (ESI): *m/z*: $[M+Na]^+$ calcd for $C_{24}H_{20}NO_3NaF$: 412.1325, found: 412.1323.

The *ee* was determined by HPLC analysis: CHIRALPAK ADH (4.6 mm i.d. × 250 mm); hexane/2-propanol = 90/10; flow rate 0.5 mL/min; 35 ^oC; 220 nm; retention time: 14.6 min (major) and 18.1 min (minor).

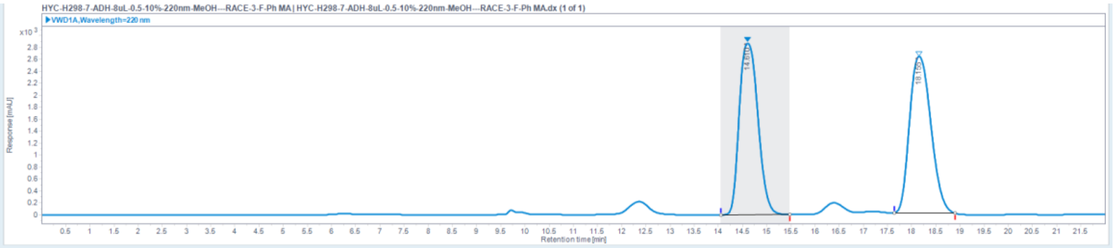

Injection Results

Peaks Summary

#	RT (min)	Area%	Start time (min)	End time (min)	Symmetry
1	14.610	48.940	14.045	15.485	0.83511
2	18.155	51.060	17.633	18.891	0.81903

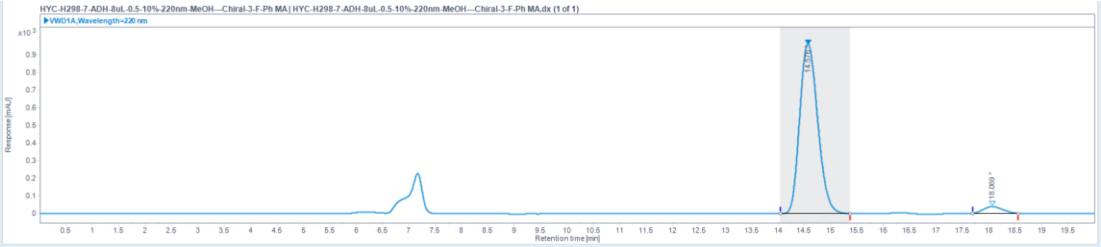

Injection Results

Peaks Summary

#	RT (min)	Area%	Start time (min)	End time (min)	Symmetry
1	14.576	95.984	14.038	15.361	0.84352
2	18.069	4.016	17.686	18.546	0.87797

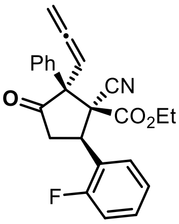

11

ethyl (1S,2S,5S)-1-cyano-5-(2-fluorophenyl)-3-oxo-2-phenyl-2-(propa-1,2-dien-1-yl)cyclopentane-1-carboxylate (11): yellow oil; 23.7 mg, 61% yield; 81% *ee*; $[\alpha]_D^{20}$ = 17.5 (*c* = 0.12, $CHCl_3$); ^{1}H NMR (400 MHz, $CDCl_3$) δ 7.60 (d, *J* = 7.5 Hz, 2H), 7.37-7.29 (m, 5H), 7.21-7.09 (m, 2H), 5.85 (t, *J* = 6.7 Hz, 1H), 5.22-5.04 (m, 2H), 4.80-4.66 (m, 1H), 3.82-3.62 (m, 2H), 3.32 (dd, *J* = 18.9, 11.7 Hz, 1H), 2.91 (dd, *J* = 18.9, 9.8 Hz, 1H), 0.75 (t, *J* = 7.1 Hz, 3H); ^{13}C NMR (100 MHz, $CDCl_3$) (two carbon signals were overlapped) δ 207.9, 205.4, 165.9, 161.6 (d, *J* = 247.0 Hz), 136.0, 130.4 (d, *J* = 8.6 Hz), 128.4, 128.3, 124.5 (d, *J* = 3.6 Hz), 121.8 (d, *J* = 13.5 Hz), 116.5 (d, *J* = 3.8 Hz), 116.3, 91.2, 80.8, 64.8, 63.0, 62.1, 38.7, 38.3, 13.5; ^{19}F NMR (376 MHz, $CDCl_3$) δ -114.3 (s); IR (neat, cm^{-1}) 2960, 2925, 2854, 1952, 1750, 1734, 1493, 1455, 1233, 1095, 1015, 855, 795, 757, 699; HRMS (ESI): *m/z*: $[M+Na]^+$ calcd for $C_{24}H_{20}NO_3NaF$: 412.1325, found: 412.1316.

The *ee* was determined by HPLC analysis: CHIRALPAK ADH (4.6 mm i.d. × 250 mm); hexane/2-propanol = 90/10; flow rate 0.5 mL/min; 35 $^{\circ}$C; 220 nm; retention time: 14.5 min (major) and 19.0 min (minor).

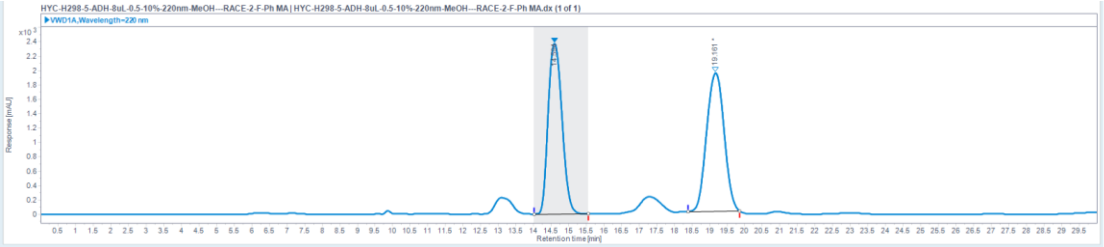

Injection Results

Peaks | Summary

#	RT (min)	Area%	Start time (min)	End time (min)	Symmetry
1	14.594	49.496	14.009	15.556	0.83658
2	19.161	50.504	18.382	19.846	0.98874

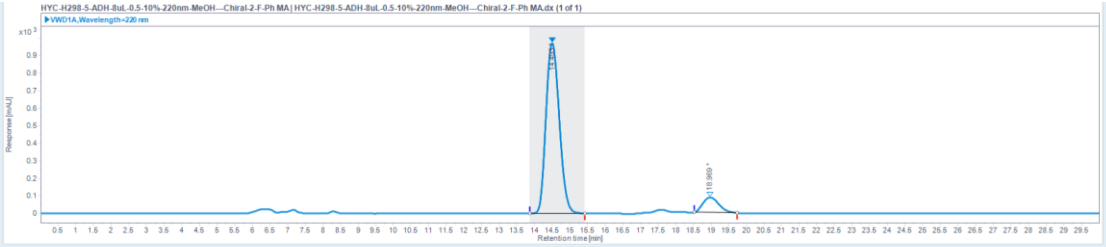

Injection Results

Peaks | Summary

#	RT (min)	Area%	Start time (min)	End time (min)	Symmetry
1	14.493	90.299	13.854	15.414	0.86145
2	18.969	9.701	18.515	19.729	0.80742

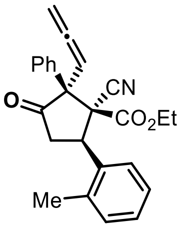

12

ethyl (1S,2S,5R)-1-cyano-3-oxo-2-phenyl-2-(propa-1,2-dien-1-yl)-5-(o-tolyl)cyclo-pentane-1-carboxylate (12): white solid; 22.3 mg, 58% yield; 84% *ee*; $[\alpha]_D^{20}$ = -23.5 (*c* = 0.115, $CHCl_3$); m.p. 74-76 °C; ^{1}H NMR (400 MHz, $CDCl_3$) δ 7.62 (d, *J* = 7.6 Hz, 2H), 7.41-7.26 (m, 4H), 7.25-7.11 (m, 3H), 5.81 (t, *J* = 6.7 Hz, 1H), 5.27-5.04 (m, 2H), 4.72 (dd, *J* = 11.4, 10.0 Hz, 1H), 3.94-3.78 (m, 1H), 3.77-3.57 (m, 1H), 3.26 (dd, *J* = 19.0, 11.8 Hz, 1H), 2.89 (dd, *J* = 19.0, 9.6 Hz, 1H), 2.64 (s, 3H), 0.78 (t, *J* = 7.1 Hz, 3H); ^{13}C NMR (100 MHz, $CDCl_3$) δ 207.7, 205.8, 166.0, 138.2, 136.3, 132.9, 131.7, 128.44, 128.37, 128.3, 128.2, 126.5, 126.4, 117.4, 91.7, 80.7, 64.9, 63.0, 62.1, 41.1, 40.3, 20.5, 13.5; IR (neat, cm^{-1}) 3062, 2962, 2926, 2854, 1952, 1750, 1733, 1446, 1257, 1097, 1016, 855, 799, 756, 733, 698; HRMS (ESI): *m/z*: $[M+Na]^+$ calcd for $C_{25}H_{23}NO_3Na$: 408.1576, found: 408.1573.

The *ee* was determined by HPLC analysis: CHIRALPAK ADH (4.6 mm i.d. × 250 mm); hexane/2-propanol = 90/10; flow rate 0.5 mL/min; 35 °C; 220 nm; retention time: 11.3 min (major) and 15.8 min (minor).

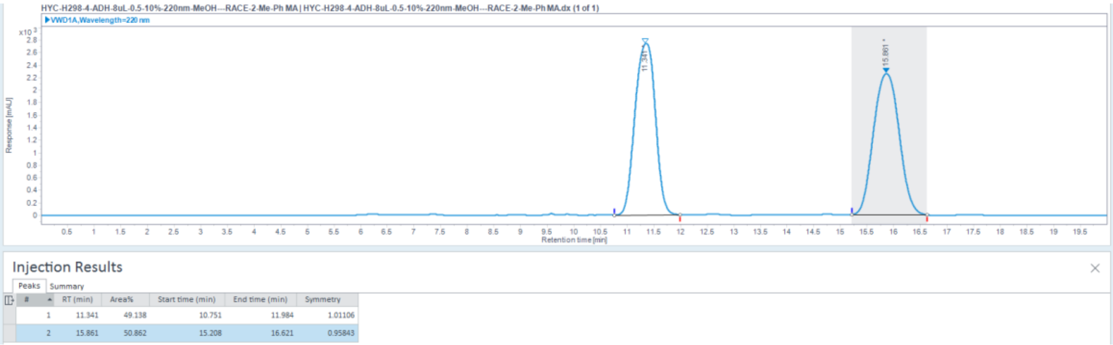

Injection Results

#	RT (min)	Area%	Start time (min)	End time (min)	Symmetry
1	11.341	49.138	10.751	11.984	1.01106
2	15.861	50.862	15.208	16.621	0.95843

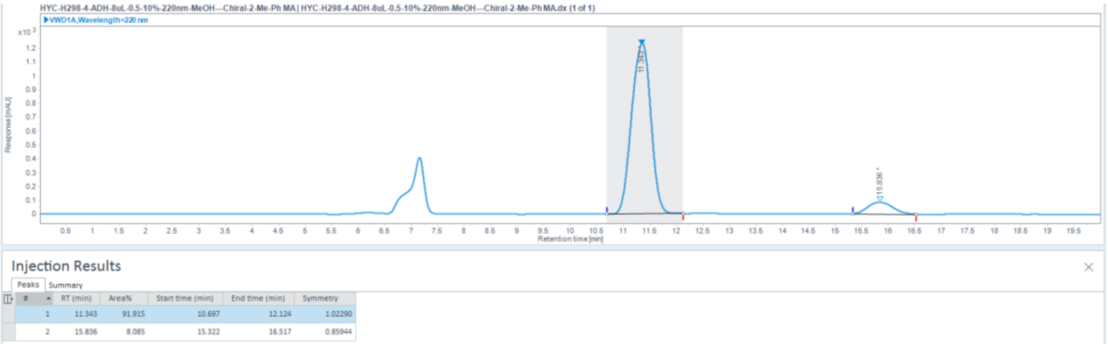

Injection Results

#	RT (min)	Area%	Start time (min)	End time (min)	Symmetry
1	11.343	91.915	10.697	12.124	1.02290
2	15.836	8.085	15.322	16.517	0.85944

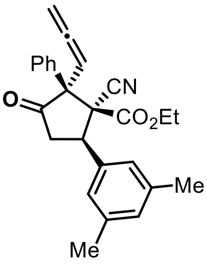

13

ethyl (1S,2S,5R)-1-cyano-5-(3,5-dimethylphenyl)-3-oxo-2-phenyl-2-(propa-1,2-dien-1-yl)cyclopentane-1-carboxylate (13): white solid; 29.5 mg, 74% yield; 95% *ee*; $[\alpha]_D^{20}$ = 28.2 (*c* = 0.110, $CHCl_3$); m.p. 68-70 ^oC; ^1H NMR (400 MHz, $CDCl_3$) δ 7.59 (d, *J* = 7.8 Hz, 2H), 7.33 (t, *J* = 7.5 Hz, 2H), 7.28 (d, *J* = 7.2 Hz, 1H), 7.01 (s, 2H), 6.97 (s, 1H), 5.84 (t, *J* = 6.7 Hz, 1H), 5.17-4.99 (m, 2H), 4.15 (t, *J* = 12.0 Hz, 1H), 3.81-3.57 (m, 2H), 3.34 (dd, *J* = 18.9, 11.7 Hz, 1H), 2.87 (dd, *J* = 18.9, 9.6 Hz, 1H), 2.31 (s, 6H), 0.74 (t, *J* = 7.1 Hz, 3H); ^{13}C NMR (100 MHz, $CDCl_3$) (one carbon signal was overlapped) δ 207.9, 206.1, 165.8, 138.6, 136.3, 134.2, 130.4, 128.4, 128.2, 126.0, 117.3, 91.5, 80.4, 64.6, 63.1, 62.8, 46.2, 38.8, 21.5, 13.5; IR (neat, cm^{-1}) 2962, 2923, 2854, 1952, 1750, 1735, 1604, 1447, 1260, 1228, 1096, 1016, 851, 799, 736, 699; HRMS (ESI): *m/z*: $[M+Na]^+$ calcd for $C_{26}H_{25}NO_3Na$: 422.1732, found: 422.1731.

The *ee* was determined by HPLC analysis: CHIRALPAK ADH (4.6 mm i.d. × 250 mm); hexane/2-propanol = 90/10; flow rate 0.5 mL/min; 35 ^oC; 220 nm; retention time: 10.6 min (major) and 12.1 min (minor).

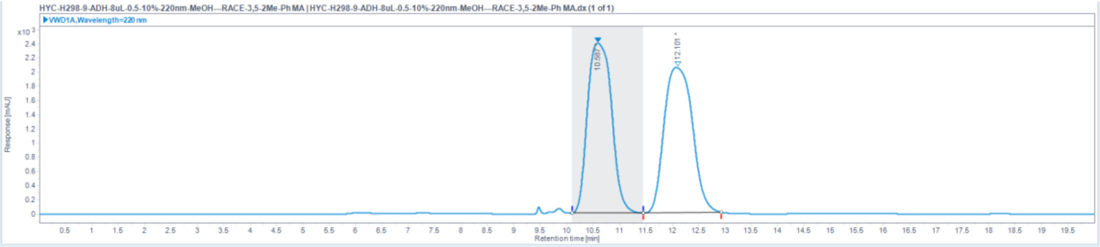

Injection Results

Peaks | Summary

#	RT (min)	Area%	Start time (min)	End time (min)	Symmetry
1	10.587	49.910	10.097	11.446	0.72699
2	12.101	50.090	11.446	12.923	0.84349

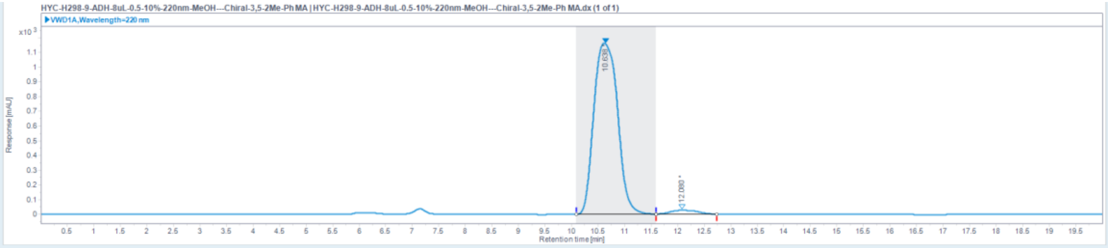

Injection Results

Peaks | Summary

#	RT (min)	Area%	Start time (min)	End time (min)	Symmetry
1	10.638	97.465	10.083	11.586	0.88525
2	12.080	2.535	11.586	12.729	0.73696

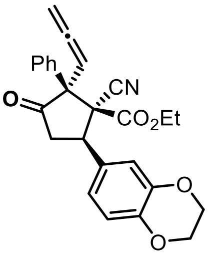

14

ethyl (1S,2S,5R)-1-cyano-5-(2,3-dihydrobenzo[b][1,4]dioxin-6-yl)-3-oxo-2-phenyl-2-(propa-1,2-dien-1-yl)cyclopentane-1-carboxylate (14): white solid; 24.5 mg, 57% yield; 99% *ee*; $[\alpha]_D^{20}$ = 29.3 (*c* = 0.140, $CHCl_3$); m.p. 84-85 ^oC; ^1H NMR (400 MHz, $CDCl_3$) δ 7.58 (d, *J* = 7.4 Hz, 2H), 7.36-7.27 (m, 3H), 7.00-6.76 (m, 3H), 5.83 (t, *J* = 6.7 Hz, 1H), 5.08 (m, 2H), 4.25 (s, 4H), 4.12 (t, *J* = 12.0 Hz, 1H), 3.84-3.62 (m, 2H), 3.27 (dd, *J* = 18.9, 11.8 Hz, 1H), 2.86 (dd, *J* = 18.9, 9.6 Hz, 1H), 0.77 (t, *J* = 7.1 Hz, 3H); ^{13}C NMR (100 MHz, $CDCl_3$) δ 207.9, 206.0, 165.7, 143.9, 143.8, 136.3, 128.4, 128.2, 128.1, 127.4, 121.3, 117.7, 117.4, 117.1, 91.5, 80.5, 64.6, 64.5, 64.4, 63.2, 62.9, 45.7, 38.9, 13.5; IR (neat, cm^{-1}) 2959, 2925, 2854, 1951, 1748, 1734, 1588, 1508, 1442, 1288, 1258, 1229, 1102, 1066, 856, 800, 736, 699; HRMS (ESI): *m/z*: $[M+Na]^+$ calcd for $C_{26}H_{23}NO_5Na$: 452.1474, found: 452.1470.

The *ee* was determined by HPLC analysis: CHIRALPAK ADH (4.6 mm i.d. × 250 mm); hexane/2-propanol = 90/10; flow rate 0.5 mL/min; 35 ^oC; 220 nm; retention time: 46.0 min (major) and 57.3 min (minor).

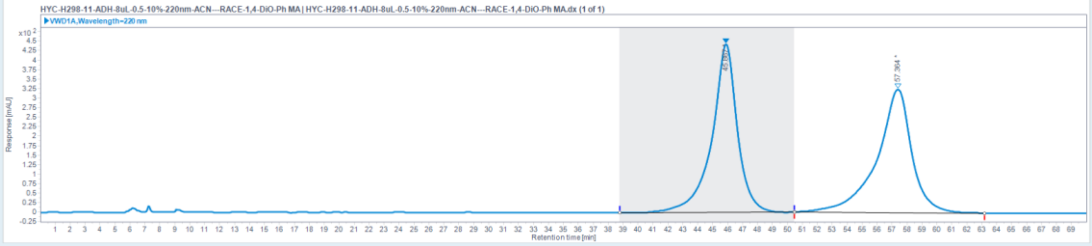

Injection Results

Peaks | Summary

#	RT (min)	Area%	Start time (min)	End time (min)	Symmetry
1	45.867	50.134	38.777	50.441	1.26537
2	57.364	49.866	50.441	63.163	1.47902

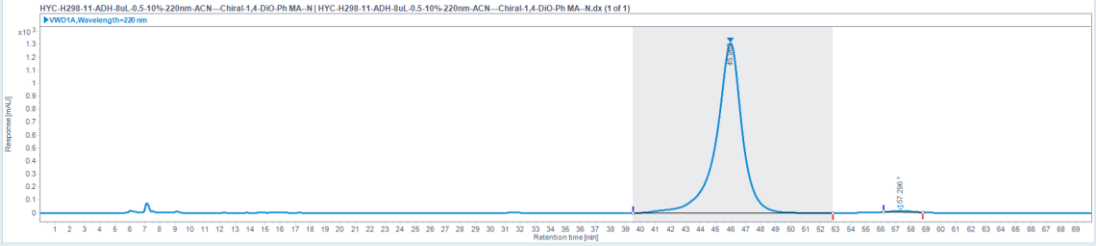

Injection Results

Peaks | Summary

#	RT (min)	Area%	Start time (min)	End time (min)	Symmetry
1	45.962	99.428	39.475	52.780	1.24642
2	57.296	0.572	56.152	58.759	0.79414

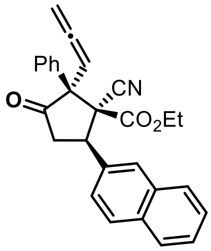

15

ethyl (1S,2S,5R)-1-cyano-5-(naphthalen-2-yl)-3-oxo-2-phenyl-2-(propa-1,2-dien-1-yl)cyclopentane-1-carboxylate (15): white solid; 21.5 mg, 51% yield; 98% *ee*; $[\alpha]_D^{20}$ = 42.5 (*c* = 0.105, $CHCl_3$); m.p. 95-97 ^oC; ^1H NMR (400 MHz, $CDCl_3$) δ 7.89-7.67 (m, 4H), 7.60-7.37 (m, 5H), 7.31-7.20 (m, 3H), 5.83 (t, *J* = 6.7 Hz, 1H), 5.17-4.90 (m, 2H), 4.43-4.24 (m, 1H), 3.67-3.52 (m, 1H), 3.52-3.29 (m, 2H), 2.92 (dd, *J* = 18.9, 9.6 Hz, 1H), 0.51 (t, *J* = 7.1 Hz, 3H); ^{13}C NMR (100 MHz, $CDCl_3$) (two carbon signals were overlapped) δ 207.9, 205.8, 165.8, 136.2, 133.3, 131.8, 128.8, 128.5, 128.3, 128.21, 128.18, 127.8, 127.6, 126.7, 125.8, 117.4, 91.5, 80.6, 64.8, 63.2, 62.9, 46.4, 38.7, 13.3; IR (neat, cm^{-1}) 2958, 2924, 2854, 1951, 1751, 1733, 1447, 1260, 1232, 1100, 1015, 856, 798, 736, 699; HRMS (ESI): *m/z*: $[M+Na]^+$ calcd for $C_{28}H_{23}NO_3Na$: 444.1576, found: 444.1573.

The *ee* was determined by HPLC analysis: CHIRALPAK ADH (4.6 mm i.d. × 250 mm); hexane/2-propanol = 90/10; flow rate 0.5 mL/min; 35 ^oC; 220 nm; retention time: 20.9 min (major) and 28.6 min (minor).

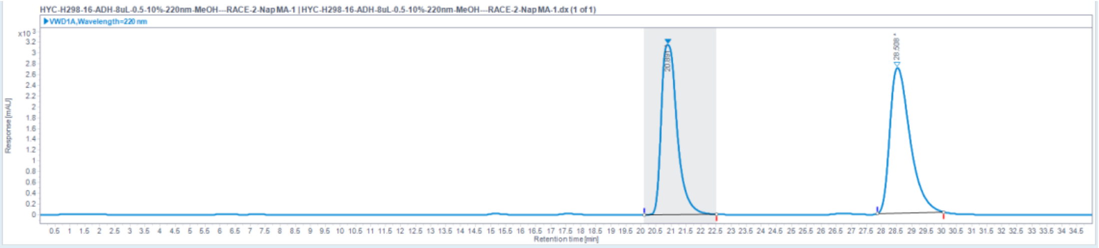

Injection Results

Peaks Summary

#	RT (min)	Area%	Start time (min)	End time (min)	Symmetry
1	20.891	47.820	20.109	22.502	0.68688
2	28.508	52.180	27.852	30.054	0.55050

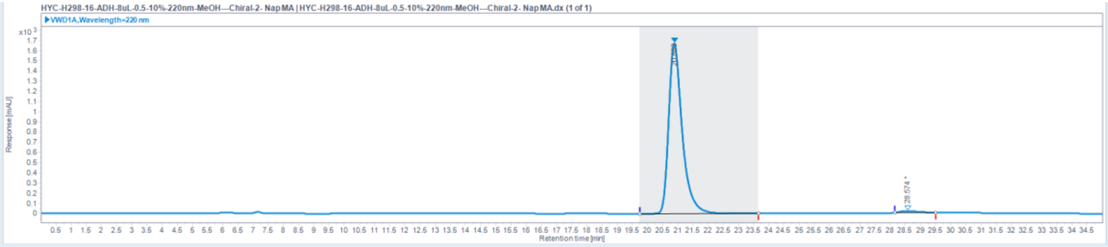

Injection Results

Peaks Summary

#	RT (min)	Area%	Start time (min)	End time (min)	Symmetry
1	20.883	98.975	19.757	23.657	0.68833
2	28.574	1.025	28.142	29.490	0.67151

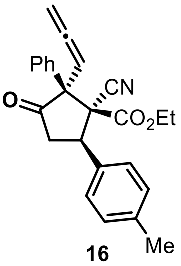

16

ethyl (1S,2S,5R)-1-cyano-3-oxo-2-phenyl-2-(propa-1,2-dien-1-yl)-5-(p-tolyl) cyclopentane-1-carboxylate (16): white solid; 31.3 mg, 70% yield; 94% *ee*; $[\alpha]_D^{20}$ = 26.7 (*c* = 0.12, $CHCl_3$); m.p. 67-69 ^oC; ^1H NMR (400 MHz, $CDCl_3$) δ 7.52 (d, *J* = 7.7 Hz, 2H), 7.33-7.16 (m, 5H), 7.11 (d, *J* = 7.9 Hz, 2H), 5.78 (t, *J* = 6.7 Hz, 1H), 5.09-4.88 (m, 2H), 4.13 (t, *J* = 12.0 Hz, 1H), 3.73-3.47 (m, 2H), 3.28 (dd, *J* = 18.9, 11.7 Hz, 1H), 2.81 (dd, *J* = 18.9, 9.6 Hz, 1H), 2.27 (s, 3H), 0.64 (t, *J* = 7.1 Hz, 3H); ^{13}C NMR (100 MHz, $CDCl_3$) δ 207.9, 206.1, 165.8, 138.6, 136.3, 131.2, 129.7, 128.4, 128.20, 128.16, 128.1, 117.4, 91.5, 80.5, 64.6, 63.3, 62.8, 45.9, 38.7, 21.2, 13.4; IR (neat, cm^{-1}) 2984, 2926, 1952, 1749, 1733, 1516, 1446, 1231, 854, 736, 698; HRMS (ESI): *m/z*: $[M+Na]^+$ calcd for $C_{25}H_{23}NO_3Na$: 408.1576, found: 408.1572.

The *ee* was determined by HPLC analysis: CHIRALPAK ADH (4.6 mm i.d. × 250 mm); hexane/2-propanol = 90/10; flow rate 0.5 mL/min; 35 ^oC; 220 nm; retention time: 16.2 min (major) and 22.2 min (minor).

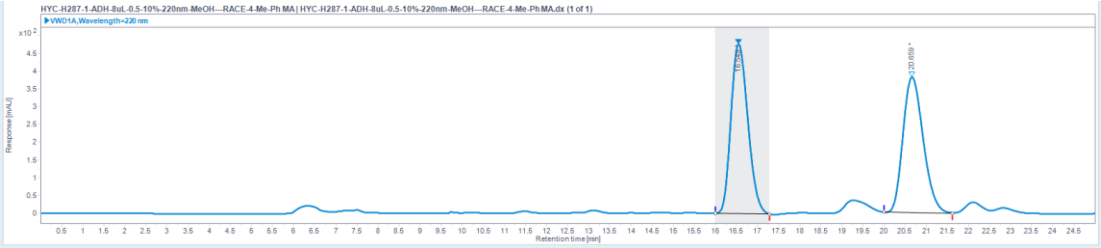

Injection Results

Peaks Summary

#	RT (min)	Area%	Start time (min)	End time (min)	Symmetry
1	16.549	51.142	15.990	17.275	0.84033
2	20.659	48.858	19.988	21.621	0.80049

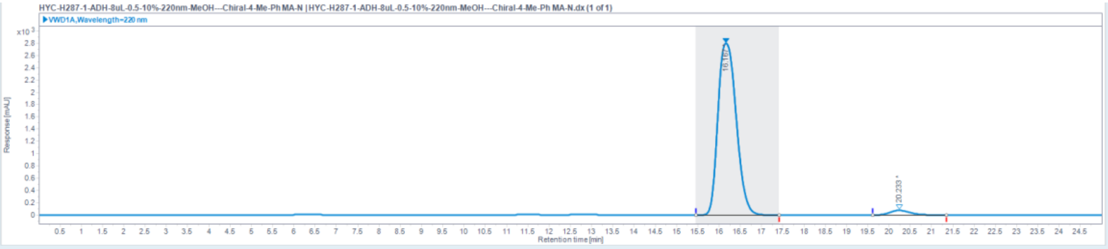

Injection Results

Peaks Summary

#	RT (min)	Area%	Start time (min)	End time (min)	Symmetry
1	16.167	97.098	15.446	17.405	0.81578
2	20.233	2.902	19.605	21.338	0.79936

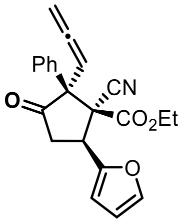

17

ethyl (1S,2S,5S)-1-cyano-5-(furan-2-yl)-3-oxo-2-phenyl-2-(propa-1,2-dien-1-yl)cyclopentane-1-carboxylate (17): yellow oil; 17.4 mg, 48% yield; 94% *ee*; $[\alpha]_D^{20}$ = 24.5 (c = 0.100, $CHCl_3$); ^{1}H NMR (400 MHz, $CDCl_3$) δ 7.57 (d, J = 8.1 Hz, 2H), 7.42 (s, 1H), 7.37-7.28 (m, 3H), 6.43-6.32 (m, 2H), 5.79 (t, J = 6.7 Hz, 1H), 5.18-5.02 (m, 2H), 4.32 (t, J = 10.5 Hz, 1H), 3.89-3.70 (m, 2H), 3.25 (dd, J = 18.8, 11.4 Hz, 1H), 2.93 (dd, J = 18.8, 9.7 Hz, 1H), 0.85 (t, J = 7.1 Hz, 3H); ^{13}C NMR (100 MHz, $CDCl_3$) δ 207.7, 204.9, 165.6, 149.3, 143.3, 135.9, 128.5, 128.3, 128.1, 117.1, 110.8, 108.8, 91.2, 80.7, 64.2, 63.1, 61.2, 40.6, 38.0, 13.5; IR (neat, cm^{-1}) 2960, 2924, 2853, 1951, 1738, 1447, 1258, 1236, 1094, 1013, 855, 797, 746, 699; HRMS (ESI): m/z: $[M+Na]^+$ calcd for $C_{22}H_{19}NO_4Na$: 384.1212, found: 384.1203.

The *ee* was determined by HPLC analysis: CHIRALPAK ODH (4.6 mm i.d. × 250 mm); hexane/2-propanol = 90/10; flow rate 0.5 mL/min; 35 °C; 220 nm; retention time: 18.0 min (major) and 20.8 min (minor).

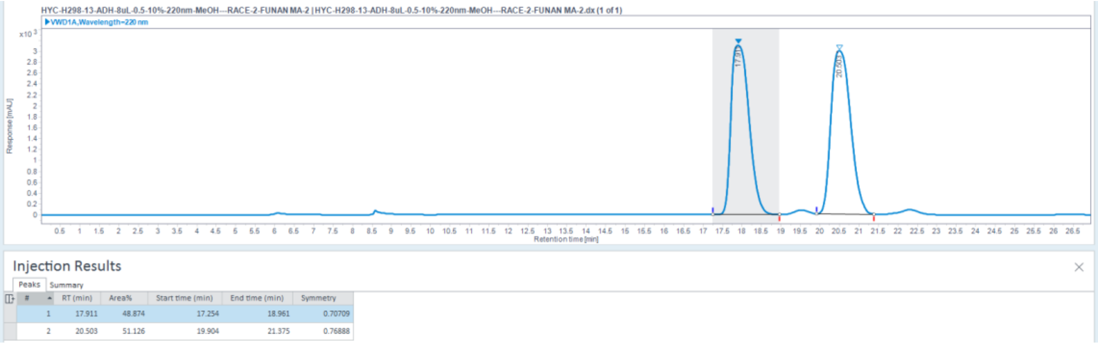

#	RT (min)	Area%	Start time (min)	End time (min)	Symmetry
1	17.911	48.874	17.254	18.961	0.70709
2	20.503	51.126	19.904	21.375	0.76888

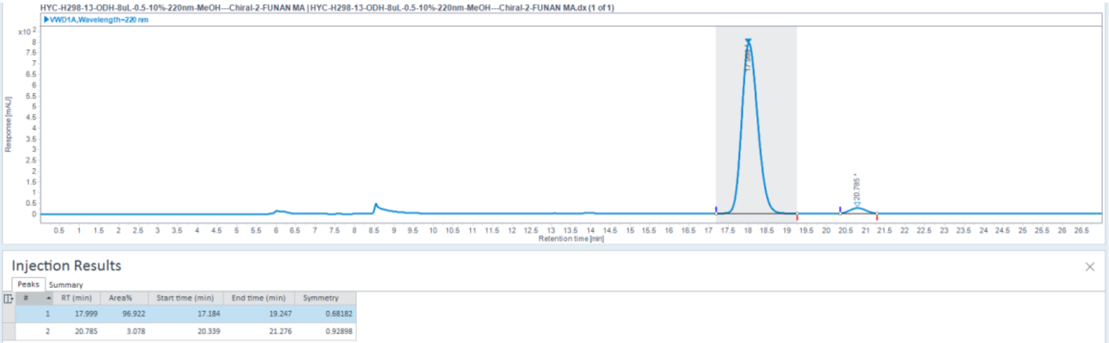

#	RT (min)	Area%	Start time (min)	End time (min)	Symmetry
1	17.999	96.922	17.184	19.247	0.68182
2	20.785	3.078	20.339	21.276	0.92898

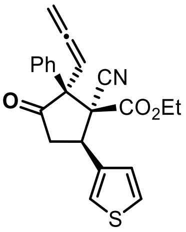

18

ethyl (1S,2S,5S)-1-cyano-3-oxo-2-phenyl-2-(propa-1,2-dien-1-yl)-5-(thiophen-3-yl)cyclopentane-1-carboxylate (18): white solid; 17.8 mg, 47% yield; 99% *ee*; $[\alpha]_D^{20}$ = 21.2 (c = 0.165, $CHCl_3$); m.p. 58-60 ^oC; ^1H NMR (400 MHz, $CDCl_3$) δ 7.57 (d, J = 7.8 Hz, 2H), 7.41-7.27 (m, 5H), 7.21 (d, J = 5.0 Hz, 1H), 5.83 (t, J = 6.7 Hz, 1H), 5.20-4.91 (m, 2H), 4.32 (t, J = 10.5 Hz, 1H), 3.81-3.56 (m, 2H), 3.29 (dd, J = 18.8, 11.5 Hz, 1H), 2.97 (dd, J = 18.8, 9.6 Hz, 1H), 0.74 (t, J = 7.1 Hz, 3H); ^{13}C NMR (100 MHz, $CDCl_3$) δ 207.8, 205.6, 165.9, 136.1, 135.9, 128.5, 128.3, 128.2, 127.1, 126.8, 123.4, 117.6, 91.3, 80.6, 64.5, 62.9, 62.7, 42.0, 39.7, 13.4; IR (neat, cm^{-1}) 3361, 2962, 2921, 2851, 1951, 1748, 1733, 1446, 1258, 1234, 1094, 1015, 853, 792, 698, 649; HRMS (ESI): *m/z*: $[M+H]^+$ calcd for $C_{22}H_{20}NO_3S$: 378.1164, found: 378.1168.

The *ee* was determined by HPLC analysis: CHIRALPAK ADH (4.6 mm i.d. $\times$ 250 mm); hexane/2-propanol = 90/10; flow rate 0.5 mL/min; 35 ^oC; 220 nm; retention time: 17.6 min (major) and 22.5 min (minor).

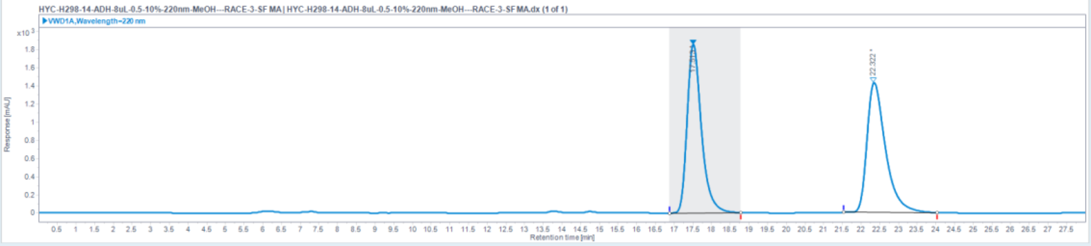

Injection Results

#	RT (min)	Area%	Start time (min)	End time (min)	Symmetry
1	17.513	50.115	16.868	18.773	0.74370
2	22.322	49.885	21.524	24.023	0.52607

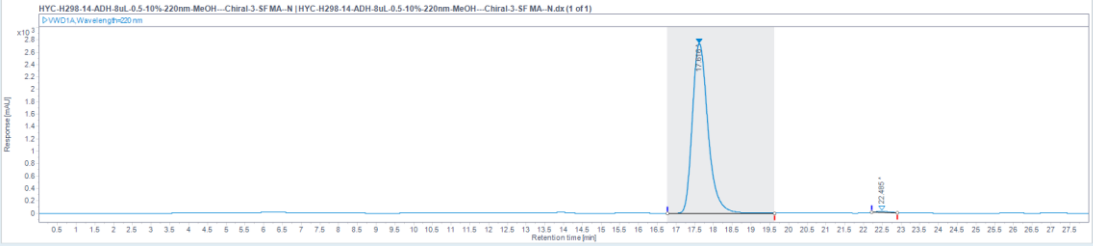

Injection Results

#	RT (min)	Area%	Start time (min)	End time (min)	Symmetry
1	17.616	99.500	16.761	19.618	0.77348
2	22.485	0.500	22.208	22.891	0.78730

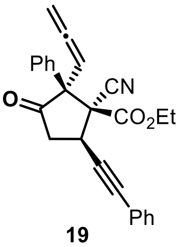

19

ethyl (1S,2S,5R)-1-cyano-3-oxo-2-phenyl-5-(phenylethynyl)-2-(propa-1,2-dien-1-yl)cyclopentane-1-carboxylate (19): white solid; 17.0 mg, 43% yield; 94% *ee*; $[\alpha]_D^{20}$ = 96.0 (*c* = 0.100, $CHCl_3$); m.p. 72-74 ^oC; ^1H NMR (400 MHz, $CDCl_3$) δ 7.60-7.52 (m, 2H), 7.41-7.28 (m, 8H), 5.71 (t, *J* = 6.7 Hz, 1H), 5.19-5.02 (m, 2H), 4.07 (t, *J* = 10.2 Hz, 1H), 4.03-3.90 (m, 2H), 3.16-2.94 (m, 2H), 0.96 (t, *J* = 7.1 Hz, 3H); ^{13}C NMR (100 MHz, $CDCl_3$) (one carbon signal was overlapped) δ 207.6, 204.6, 165.7, 135.6, 131.9, 129.0, 128.6, 128.5, 128.3, 122.1, 116.6, 90.9, 85.7, 83.9, 81.0, 63.5, 63.3, 61.5, 40.6, 33.9, 13.8; IR (neat, cm^{-1}) 2957, 2923, 2853, 1951, 1743, 1448, 1259, 1231, 1098, 1016, 856, 796, 756, 695; HRMS (ESI): *m/z*: $[M+Na]^+$ calcd for $C_{26}H_{21}NO_3Na$: 418.1419, found: 418.1411.

The *ee* was determined by HPLC analysis: CHIRALPAK ADH (4.6 mm i.d. × 250 mm); hexane/2-propanol = 90/10; flow rate 0.5 mL/min; 35 ^oC; 220 nm; retention time: 28.2 min (major) and 26.6 min (minor).

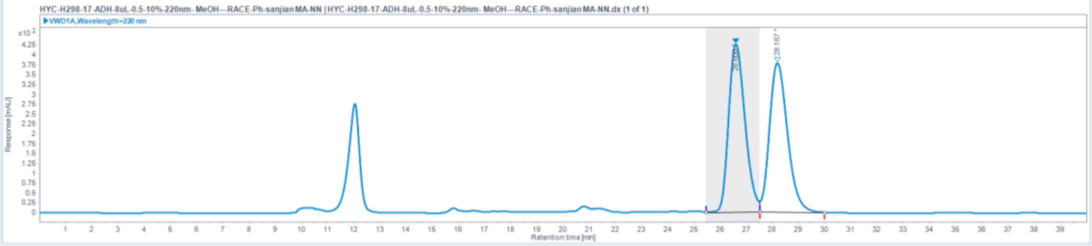

Injection Results

Peaks Summary

#	RT (min)	Area%	Start time (min)	End time (min)	Symmetry
1	26.600	50.611	25.478	27.513	0.77796
2	28.187	49.389	27.513	30.005	0.75224

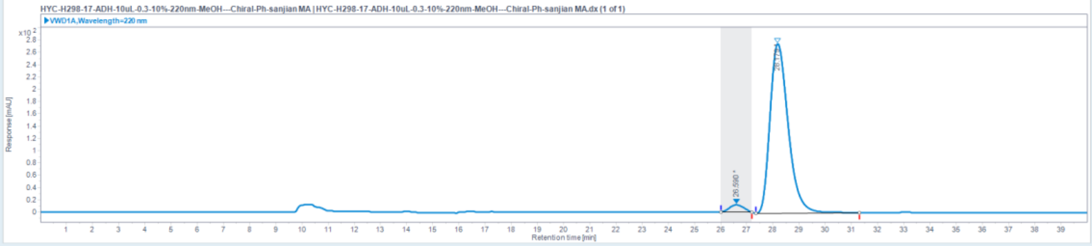

Injection Results

Peaks Summary

#	RT (min)	Area%	Start time (min)	End time (min)	Symmetry
1	26.590	3.065	25.999	27.181	0.93614
2	28.179	96.935	27.335	31.291	0.72295

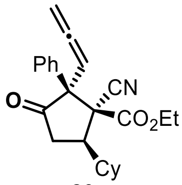

20

ethyl (1S,2S,5R)-1-cyano-5-cyclohexyl-3-oxo-2-phenyl-2-(propa-1,2-dien-1-yl)cyclopentane-1-carboxylate (20): white solid; 18.5 mg, 49% yield; 94% *ee*; $[\alpha]_D^{20}$ = 61.8 (c = 0.110, $CHCl_3$); m.p. 75-76 °C; ^{1}H NMR (400 MHz, $CDCl_3$) δ 7.51 (d, J = 7.8 Hz, 2H), 7.37-7.27 (m, 3H), 5.79 (t, J = 6.7 Hz, 1H), 5.15-4.87 (m, 2H), 4.01-3.78 (m, 2H), 2.86-2.50 (m, 3H), 2.15 (d, J = 12.3 Hz, 1H), 1.88-1.63 (m, 4H), 1.48-1.11 (m, 6H), 0.94 (t, J = 7.1 Hz, 3H); ^{13}C NMR (100 MHz, $CDCl_3$) δ 208.0, 206.6, 166.4, 135.8, 128.6, 128.33, 128.27, 118.5, 91.2, 80.2, 65.1, 62.9, 59.7, 47.0, 41.4, 40.2, 32.4, 31.4, 26.1, 26.0, 25.8, 13.8; IR (neat, cm^{-1}) 2983, 2926, 2853, 1952, 1734, 1447, 1245, 1222, 1003, 852, 754, 699; HRMS (ESI): m/z: $[M+Na]^+$ calcd for $C_{24}H_{27}NO_3Na$: 400.1889, found: 400.1887.

The *ee* was determined by HPLC analysis: CHIRALPAK ADH (4.6 mm i.d. × 250 mm); hexane/2-propanol = 90/10; flow rate 0.5 mL/min; 35 °C; 220 nm; retention time: 10.9 min (major) and 14.2 min (minor).

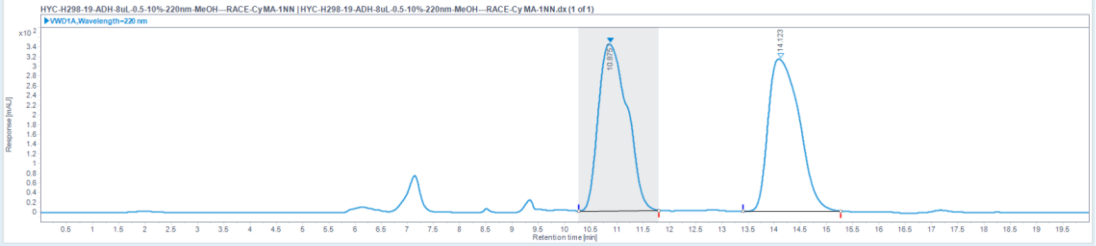

Injection Results

Peaks | Summary

#	RT (min)	Area%	Start time (min)	End time (min)	Symmetry
1	10.875	50.348	10.263	11.791	0.66955
2	14.123	49.652	13.396	15.259	0.62566

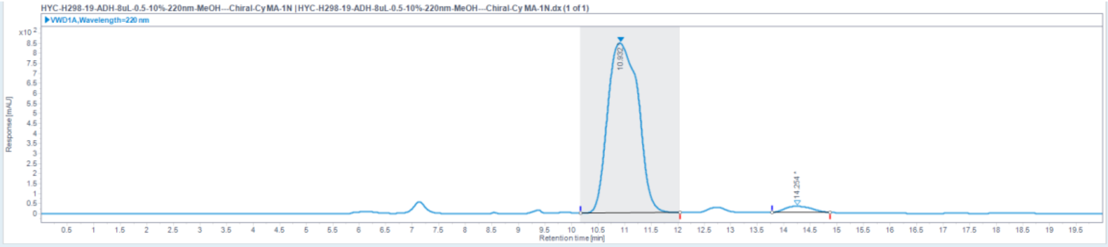

Injection Results

Peaks | Summary

#	RT (min)	Area%	Start time (min)	End time (min)	Symmetry
1	10.932	96.942	10.162	12.037	0.75029
2	14.254	3.058	13.771	14.862	0.84062

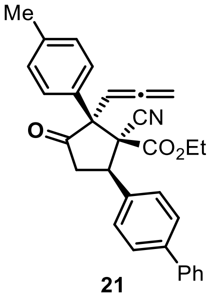

21

ethyl (1S,2S,5R)-5-([1,1'-biphenyl]-4-yl)-1-cyano-3-oxo-2-(propa-1,2-dien-1-yl)-2-(p-tolyl)cyclopentane-1-carboxylate (21): white solid; 27.7 mg, 60% yield; 94% *ee*; $[\alpha]_D^{20}$ = 19.2 (*c* = 0.125, $CHCl_3$); m.p. 99-101 ^oC; ^1H NMR (400 MHz, $CDCl_3$) δ 7.60 (t, *J* = 8.9 Hz, 4H), 7.53-7.42 (m, 6H), 7.40-7.34 (m, 1H), 7.15 (d, *J* = 8.0 Hz, 2H), 5.86 (t, *J* = 6.6 Hz, 1H), 5.19-4.98 (m, 2H), 4.28 (t, *J* = 10.6 Hz, 1H), 3.80-3.59 (m, 2H), 3.40 (dd, *J* = 18.8, 11.6 Hz, 1H), 2.93 (dd, *J* = 18.9, 9.6 Hz, 1H), 2.31 (s, 3H), 0.73 (t, *J* = 7.1 Hz, 3H); ^{13}C NMR (100 MHz, $CDCl_3$) δ 207.8, 206.0, 165.8, 141.6, 140.3, 138.1, 133.4, 133.1, 129.1, 129.0, 128.7, 128.0, 127.8, 127.6, 127.2, 117.5, 91.6, 80.5, 64.6, 63.2, 62.9, 45.9, 38.6, 21.2, 13.4; IR (neat, cm^{-1}) 3031, 2958, 2920, 2852, 1951, 1749, 1736, 1488, 1230, 1105, 1005, 850, 809, 764, 732, 698; HRMS (ESI): *m/z*: $[M+Na]^+$ calcd for $C_{31}H_{27}NO_3Na$: 484.1889, found: 484.1888.

The *ee* was determined by HPLC analysis: CHIRALPAK ODH (4.6 mm i.d. × 250 mm); hexane/2-propanol = 90/10; flow rate 0.5 mL/min; 35 ^oC; 220 nm; retention time: 27.1 min (major) and 24.3 min (minor).

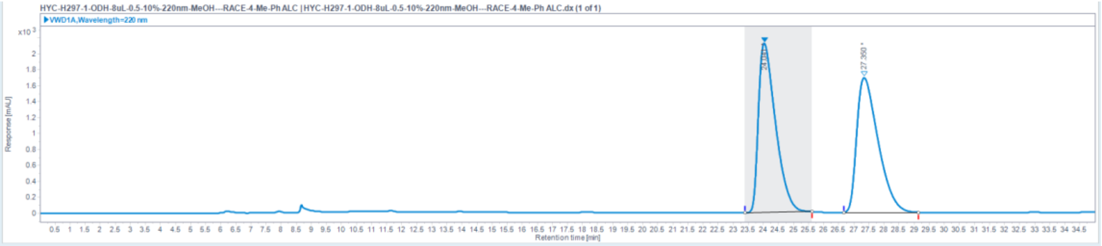

Injection Results

Peaks Summary

#	RT (min)	Area%	Start time (min)	End time (min)	Symmetry
1	24.041	50.154	23.378	25.603	0.54668
2	27.350	49.846	26.659	29.132	0.54376

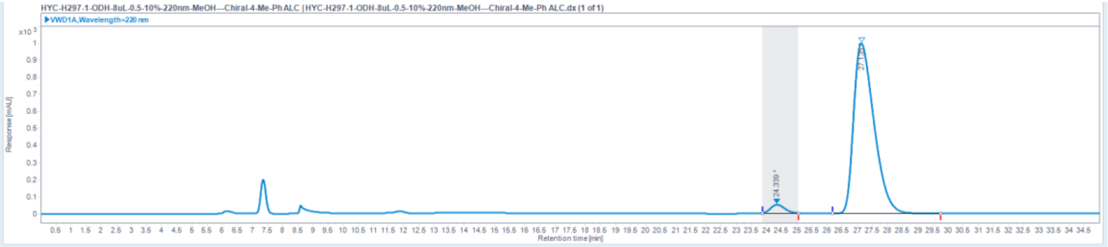

Injection Results

Peaks Summary

#	RT (min)	Area%	Start time (min)	End time (min)	Symmetry
1	24.339	3.250	23.850	25.041	0.83052
2	27.135	96.750	26.165	29.739	0.61714

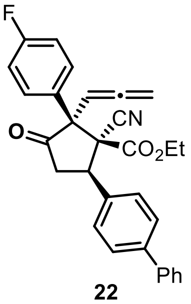

22

ethyl (1S,2S,5R)-5-([1,1'-biphenyl]-4-yl)-1-cyano-2-(4-fluorophenyl)-3-oxo-2-(propa-1,2-dien-1-yl)cyclopentane-1-carboxylate (22): white solid; 28.9 mg, 62% yield; 93% *ee*; $[\alpha]_D^{20}$ = 49.2 (*c* = 0.195, $CHCl_3$); m.p. 66-68 °C; ^{1}H NMR (400 MHz, $CDCl_3$) δ 7.65-7.52 (m, 6H), 7.52-7.40 (m, 4H), 7.37 (t, *J* = 7.3 Hz, 1H), 7.04 (t, *J* = 8.7 Hz, 2H), 5.84 (t, *J* = 6.7 Hz, 1H), 5.23-4.96 (m, 2H), 4.37-4.16 (m, 1H), 3.84-3.56 (m, 2H), 3.41 (dd, *J* = 19.0, 11.6 Hz, 1H), 2.95 (dd, *J* = 18.9, 9.6 Hz, 1H), 0.73 (t, *J* = 7.1 Hz, 3H); ^{13}C NMR (100 MHz, $CDCl_3$) δ 208.0, 205.6, 165.7, 162.4 (d, *J* = 246.0 Hz), 141.7, 140.3, 133.2, 132.0, 130.3 (d, *J* = 8.1 Hz), 129.0, 128.6, 127.8, 127.7, 127.1, 117.3, 115.4 (d, *J* = 21.0 Hz), 91.2, 80.8, 64.1, 63.2, 63.0, 45.9, 38.3, 13.5; ^{19}F NMR (376 MHz, $CDCl_3$) δ -113.3 (s); IR (neat, cm^{-1}) 2957, 2924, 2853, 1951, 1753, 1737, 1603, 1511, 1264, 1234, 833, 734, 700; HRMS (ESI): *m/z*: $[M+Na]^+$ calcd for $C_{30}H_{24}NO_3NaF$: 488.1638, found: 488.1644.

The *ee* was determined by HPLC analysis: CHIRALPAK ODH (4.6 mm i.d. × 250 mm); hexane/2-propanol = 90/10; flow rate 0.5 mL/min; 35 °C; 220 nm; retention time: 28.0 min (major) and 26.2 min (minor).

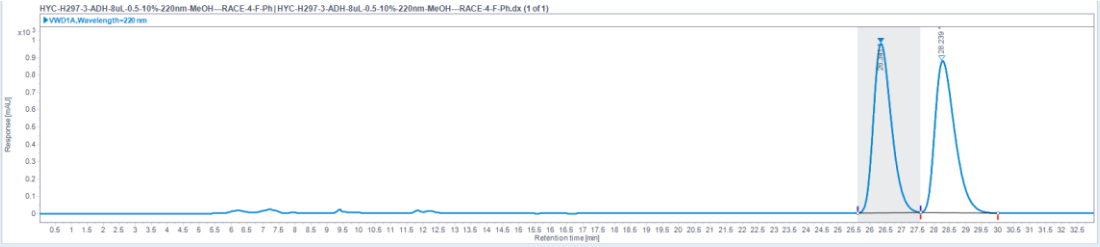

Injection Results

#	RT (min)	Area%	Start time (min)	End time (min)	Symmetry
1	26.341	50.152	25.602	27.573	0.72503
2	28.239	49.848	27.573	29.988	0.54849

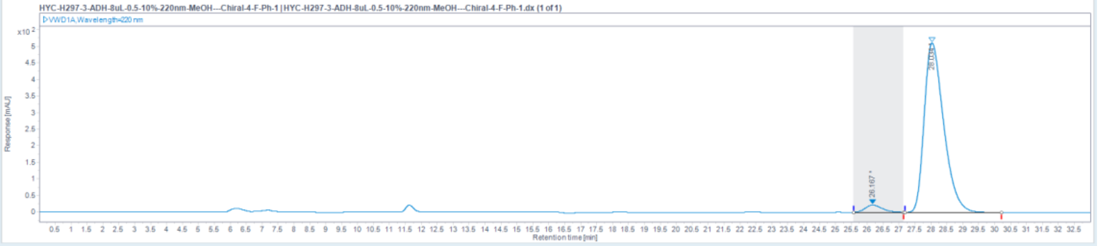

Injection Results

#	RT (min)	Area%	Start time (min)	End time (min)	Symmetry
1	26.167	3.569	25.559	27.141	0.77618
2	28.034	96.431	27.185	30.201	0.66919

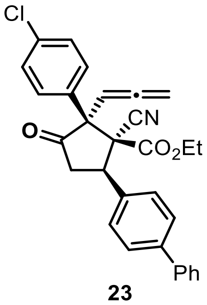

23

ethyl (1S,2S,5R)-5-([1,1'-biphenyl]-4-yl)-2-(4-chlorophenyl)-1-cyano-3-oxo-2-(propa-1,2-dien-1-yl)cyclopentane-1-carboxylate (23): white solid; 27.5 mg, 57% yield; 94% *ee*; $[\alpha]_D^{20}$ = 18.6 (*c* = 0.07, $CHCl_3$); m.p. 63-65 °C; ^1H NMR (400 MHz, $CDCl_3$) δ 7.64-7.54 (m, 6H), 7.46 (dd, *J* = 17.1, 8.1 Hz, 4H), 7.40-7.29 (m, 3H), 5.82 (t, *J* = 6.7 Hz, 1H), 5.21-5.01 (m, 2H), 4.28 (t, *J* = 12.0 Hz, 1H), 3.81-3.60 (m, 2H), 3.39 (dd, *J* = 19.0, 11.6 Hz, 1H), 2.94 (dd, *J* = 19.0, 9.6 Hz, 1H), 0.74 (t, *J* = 7.1 Hz, 3H); ^{13}C NMR (100 MHz, $CDCl_3$) (one carbon signal was overlapped) δ 208.1, 205.4, 165.7, 141.8, 140.2, 134.8, 134.4, 133.0, 129.7, 129.0, 128.6, 127.8, 127.7, 127.1, 117.2, 91.1, 80.8, 64.0, 63.1, 63.0, 45.9, 38.3, 13.5; IR (neat, cm^{-1}) 3057, 2926, 1951, 1753, 1736, 1492, 1264, 1234, 1096, 1012, 852, 732, 700; HRMS (ESI): *m/z*: $[M+Na]^+$ calcd for $C_{30}H_{24}NO_3NaCl$: 504.1342, found: 504.1344.

The *ee* was determined by HPLC analysis: CHIRALPAK ODH (4.6 mm i.d. × 250 mm); hexane/2-propanol = 90/10; flow rate 0.5 mL/min; 35 °C; 220 nm; retention time: 49.1 min (major) and 46.7 min (minor).

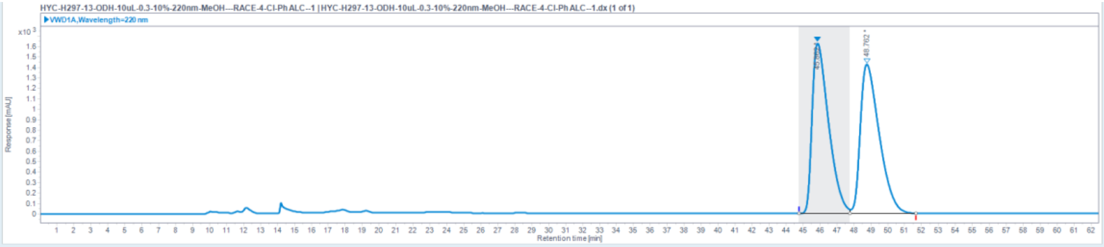

Injection Results

Peaks | Summary

#	RT (min)	Area%	Start time (min)	End time (min)	Symmetry
1	45.862	49.878	44.746	47.764	0.49688
2	48.762	50.122	47.764	51.642	0.51716

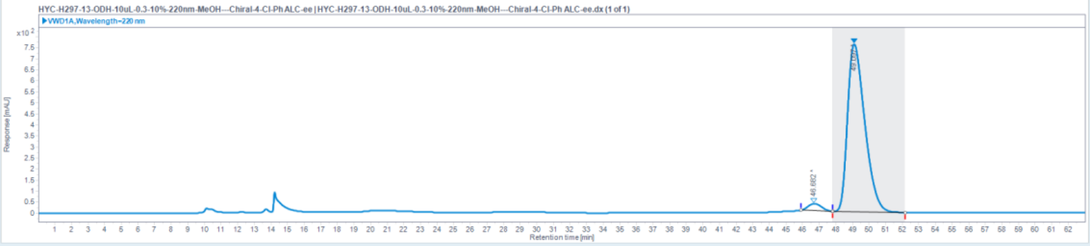

Injection Results

Peaks | Summary

#	RT (min)	Area%	Start time (min)	End time (min)	Symmetry
1	46.682	3.106	45.915	47.784	0.80362
2	49.097	96.894	47.784	52.186	0.61096

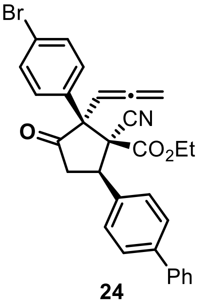

24

ethyl (1S,2S,5R)-5-([1,1'-biphenyl]-4-yl)-2-(4-bromophenyl)-1-cyano-3-oxo-2-(propa-1,2-dien-1-yl)cyclopentane-1-carboxylate (24): white solid; 28.4 mg, 54% yield; 93% *ee*; $[\alpha]_D^{20}$ = -2.6 (*c* = 0.115, $CHCl_3$); m.p. 65-67 ^oC; ^1H NMR (400 MHz, $CDCl_3$) δ 7.65-7.56 (m, 4H), 7.53-7.42 (m, 8H), 7.37 (t, *J* = 7.3 Hz, 1H), 5.81 (t, *J* = 6.7 Hz, 1H), 5.21-5.01 (m, 2H), 4.28 (dd, *J* = 11.2, 10.0 Hz, 1H), 3.81-3.56 (m, 2H), 3.39 (dd, *J* = 19.0, 11.6 Hz, 1H), 2.94 (dd, *J* = 19.0, 9.6 Hz, 1H), 0.74 (t, *J* = 7.1 Hz, 3H); ^{13}C NMR (100 MHz, $CDCl_3$) δ 208.1, 205.2, 165.7, 141.8, 140.2, 135.3, 133.1, 131.6, 130.0, 129.0, 128.6, 127.8, 127.7, 127.1, 122.6, 117.2, 91.0, 80.8, 64.1, 63.1, 62.9, 46.0, 38.3, 13.5; IR (neat, cm^{-1}) 2958, 2923, 2853, 1951, 1751, 1733, 1489, 1231, 1009, 850, 810, 763, 734, 698; HRMS (ESI): *m/z*: $[M+Na]^+$ calcd for $C_{30}H_{24}NO_3NaBr$: 548.0837, found: 548.0841.

The *ee* was determined by HPLC analysis: CHIRALPAK ODH (4.6 mm i.d. × 250 mm); hexane/2-propanol = 90/10; flow rate 0.5 mL/min; 35 ^oC; 220 nm; retention time: 33.2 min (major) and 45.6 min (minor).

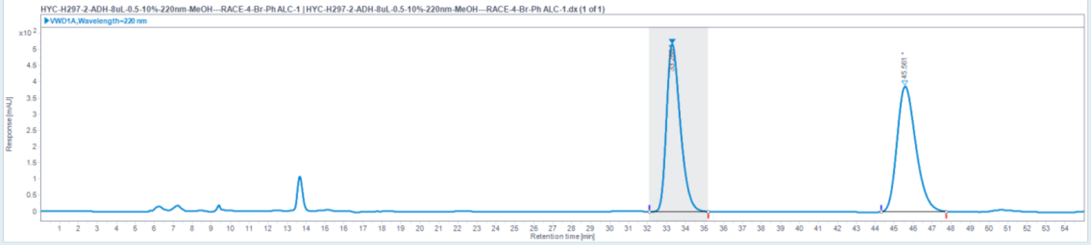

Injection Results

Peaks Summary

#	RT (min)	Area%	Start time (min)	End time (min)	Symmetry
1	33.286	49.394	32.073	35.182	0.69084
2	45.561	50.606	44.295	47.721	0.73486

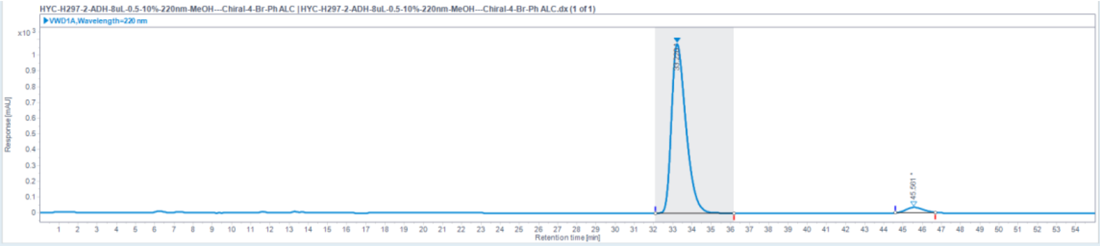

Injection Results

Peaks Summary

#	RT (min)	Area%	Start time (min)	End time (min)	Symmetry
1	33.220	96.469	32.073	36.171	0.63854
2	45.561	3.531	44.577	46.661	0.88470

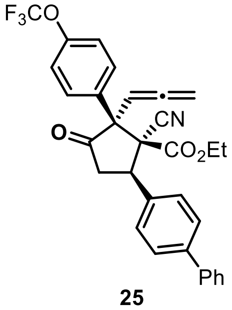

25

ethyl (1S,2S,5R)-5-([1,1'-biphenyl]-4-yl)-1-cyano-3-oxo-2-(propa-1,2-dien-1-yl)-2-(4-(trifluoromethoxy)phenyl)cyclopentane-1-carboxylate (25): white solid; 28.4 mg, 54% yield; 93% *ee*; $[\alpha]_D^{20}$ = 35.0 (*c* = 0.160, $CHCl_3$); m.p. 81-83 °C; ^{1}H NMR (400 MHz, $CDCl_3$) δ 7.67-7.56 (m, 6H), 7.51-7.42 (m, 4H), 7.37 (t, *J* = 7.3 Hz, 1H), 7.20 (d, *J* = 8.6 Hz, 2H), 5.84 (t, *J* = 6.7 Hz, 1H), 5.22-5.01 (m, 2H), 4.29 (dd, *J* = 11.2, 10.0 Hz, 1H), 3.81-3.58 (m, 2H), 3.41 (dd, *J* = 19.0, 11.6 Hz, 1H), 2.95 (dd, *J* = 19.0, 9.6 Hz, 1H), 0.70 (t, *J* = 7.1 Hz, 3H); ^{13}C NMR (100 MHz, $CDCl_3$) δ 208.1, 205.3, 165.7, 149.0, 141.8, 140.2, 134.9, 133.1, 130.1, 129.0, 128.7, 127.8, 127.7, 127.1, 120.8, 120.5 (dd, *J* = 256.0 Hz), 117.2, 91.0, 80.8, 64.0, 63.1, 63.1, 45.9, 38.3, 13.4; ^{19}F NMR (376 MHz, $CDCl_3$) δ -57.8 (s); IR (neat, cm^{-1}) 2925, 2854, 1952, 1753, 1736, 1510, 1257, 1215, 1167, 849, 734, 700; HRMS (ESI): *m/z*: $[M+Na]^+$ calcd for $C_{31}H_{24}NO_4NaF_3$: 554.1555, found: 554.1553.

The *ee* was determined by HPLC analysis: CHIRALPAK ADH (4.6 mm i.d. × 250 mm); hexane/2-propanol = 90/10; flow rate 0.5 mL/min; 35 °C; 220 nm; retention time: 19.0 min (major) and 28.5 min (minor).

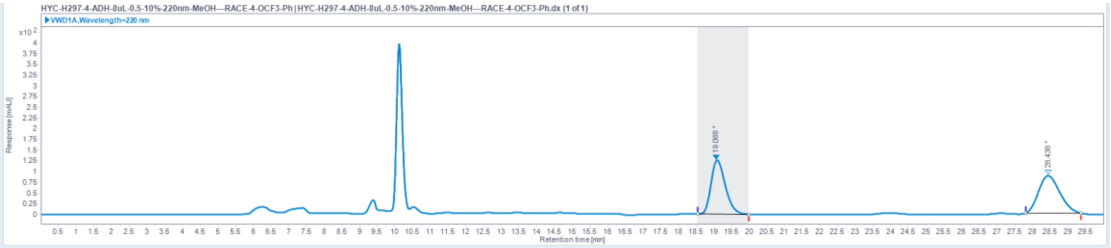

Injection Results

Peaks Summary

#	RT (min)	Area%	Start time (min)	End time (min)	Symmetry
1	19.068	49.247	18.535	19.980	0.61804
2	28.438	50.753	27.801	29.361	0.79922

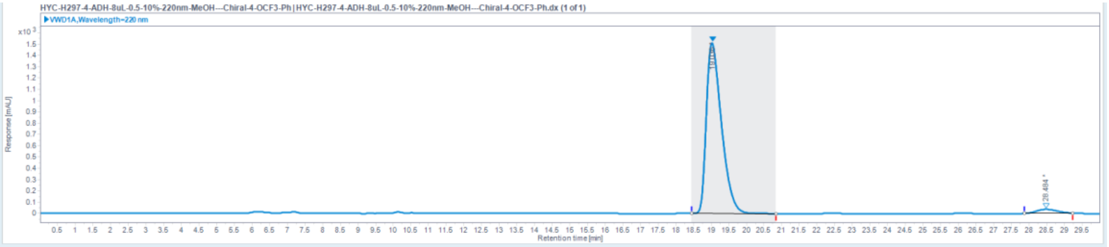

Injection Results

Peaks Summary

#	RT (min)	Area%	Start time (min)	End time (min)	Symmetry
1	19.036	96.984	18.439	20.828	0.64391
2	28.484	3.016	27.860	29.228	0.85705

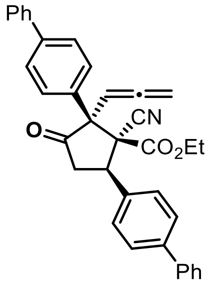

26

ethyl (1S,2S,5R)-2,5-di([1,1'-biphenyl]-4-yl)-1-cyano-3-oxo-2-(propa-1,2-dien-1-yl)cyclopentane-1-carboxylate (26): white solid; 37.7 mg, 72% yield; 94% *ee*; $[\alpha]_D^{20}$ = -32.1 (c = 0.135, $CHCl_3$); m.p. 102-103 °C; ^{1}H NMR (400 MHz, $CDCl_3$) δ 7.69 (d, J = 8.4 Hz, 2H), 7.67-7.50 (m, 10H), 7.45 (dd, J = 14.6, 7.3 Hz, 4H), 7.37 (dd, J = 14.1, 7.1 Hz, 2H), 5.93 (t, J = 6.7 Hz, 1H), 5.28-5.03 (m, 2H), 4.46-4.20 (m, 1H), 3.89-3.61 (m, 2H), 3.45 (dd, J = 18.9, 11.6 Hz, 1H), 2.97 (dd, J = 18.9, 9.6 Hz, 1H), 0.72 (t, J = 7.1 Hz, 3H); ^{13}C NMR (100 MHz, $CDCl_3$) δ 207.9, 205.8, 165.8, 141.7, 141.0, 140.4, 140.3, 135.1, 133.3, 129.0, 129.0, 128.7, 128.6, 127.8, 127.7, 127.7, 127.2, 127.1, 127.1, 117.4, 91.3, 80.6, 64.6, 63.2, 63.1, 46.0, 38.5, 13.4; IR (neat, cm^{-1}) 3058, 2924, 2853, 1951, 1752, 1488, 1233, 1107, 1005, 850, 764, 731, 698; HRMS (ESI): m/z: $[M+Na]^+$ calcd for $C_{36}H_{29}NO_3Na$: 546.2045, found: 546.2045.

The *ee* was determined by HPLC analysis: CHIRALPAK ODH (4.6 mm i.d. × 250 mm); hexane/2-propanol = 90/10; flow rate 0.5 mL/min; 35 °C; 220 nm; retention time: 39.8 min (major) and 35.4 min (minor).

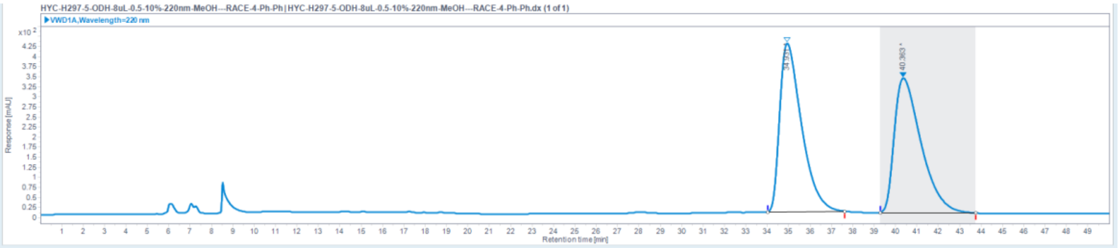

Injection Results

Peaks Summary

#	RT (min)	Area%	Start time (min)	End time (min)	Symmetry
1	34.931	50.490	34.006	37.602	0.51773
2	40.363	49.510	39.272	43.735	0.49538

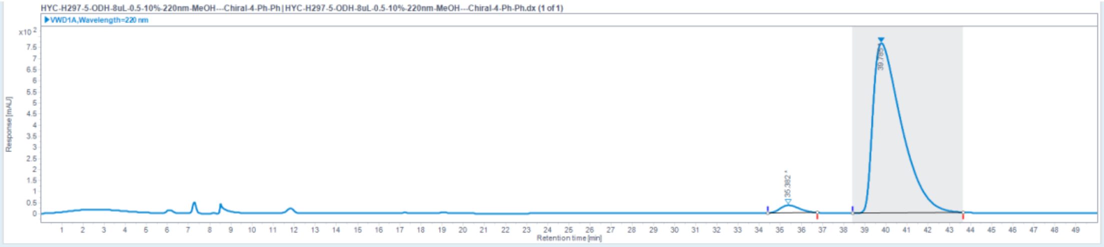

Injection Results

Peaks Summary

#	RT (min)	Area%	Start time (min)	End time (min)	Symmetry
1	35.382	2.963	34.393	36.737	0.76996
2	39.785	97.037	38.407	43.641	0.40050

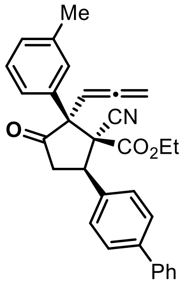

27

ethyl (1S,2S,5R)-5-([1,1'-biphenyl]-4-yl)-1-cyano-3-oxo-2-(propa-1,2-dien-1-yl)-2-(m-tolyl)cyclopentane-1-carboxylate (27): white solid; 33.7 mg, 73% yield; 95% *ee*; $[\alpha]_D^{20}$ = 30.7 (*c* = 0.140, $CHCl_3$); m.p. 112-113 °C; ^{1}H NMR (400 MHz, $CDCl_3$) δ 7.61 (dd, *J* = 10.3, 7.8 Hz, 4H), 7.51 (d, *J* = 8.3 Hz, 2H), 7.49-7.34 (m, 5H), 7.24 (t, *J* = 8.0 Hz, 1H), 7.11 (d, *J* = 7.5 Hz, 1H), 5.88 (t, *J* = 6.7 Hz, 1H), 5.21-5.03 (m, 2H), 4.29 (dd, *J* = 11.5, 9.7 Hz, 1H), 3.81-3.59 (m, 2H), 3.43 (dd, *J* = 18.9, 11.7 Hz, 1H), 2.94 (dd, *J* = 18.9, 9.6 Hz, 1H), 2.36 (s, 3H), 0.73 (t, *J* = 7.1 Hz, 3H); ^{13}C NMR (100 MHz, $CDCl_3$) (one carbon signal was overlapped) δ 207.8, 205.9, 165.7, 141.6, 140.3, 138.0, 136.0, 133.4, 129.0, 128.71, 128.67, 128.3, 127.7, 127.6, 127.1, 125.2, 117.4, 91.5, 80.5, 64.7, 63.2, 62.8, 45.9, 38.6, 21.7, 13.4; IR (neat, cm^{-1}) 2923, 2853, 1951, 1750, 1736, 1604, 1488, 1230, 1108, 1005, 849, 764, 733, 699; HRMS (ESI): *m/z*: $[M+Na]^+$ calcd for $C_{31}H_{27}NO_3Na$: 484.1889, found: 484.1888.

The *ee* was determined by HPLC analysis: CHIRALPAK ODH (4.6 mm i.d. × 250 mm); hexane/2-propanol = 90/10; flow rate 0.5 mL/min; 35 °C; 220 nm; retention time: 24.1 min (major) and 26.8 min (minor).

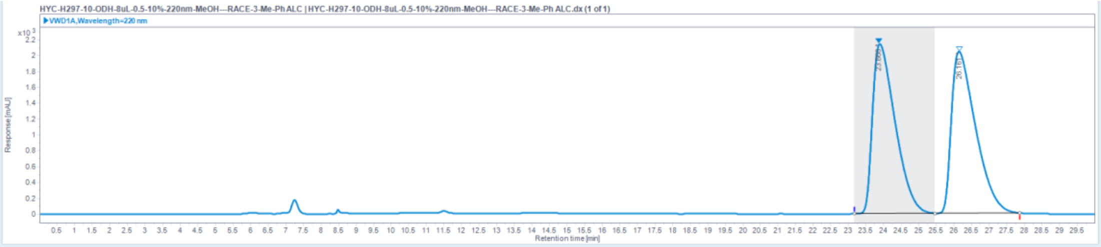

Injection Results

Peaks Summary

#	RT (min)	Area%	Start time (min)	End time (min)	Symmetry
1	23.866	49.621	23.159	25.453	0.42026
2	26.161	50.379	25.453	27.860	0.48287

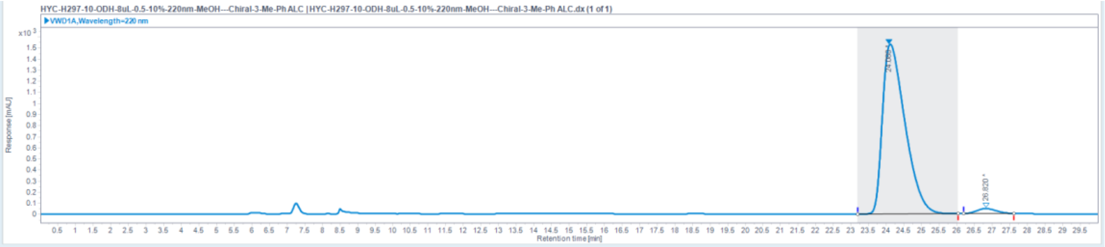

Injection Results

Peaks Summary

#	RT (min)	Area%	Start time (min)	End time (min)	Symmetry
1	24.080	97.531	23.178	26.029	0.44589
2	26.820	2.469	26.184	27.609	0.87626

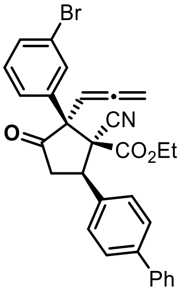

28

ethyl (1S,2S,5R)-5-([1,1'-biphenyl]-4-yl)-2-(3-bromophenyl)-1-cyano-3-oxo-2-(propa-1,2-dien-1-yl)cyclopentane-1-carboxylate (28): white solid; 27.3 mg, 52% yield; 95% *ee*; $[\alpha]_D^{20}$ = -5.0 (*c* = 0.200, $CHCl_3$); m.p. 70-72 °C; ^1H NMR (400 MHz, $CDCl_3$) δ 7.77 (s, 1H), 7.65-7.53 (m, 5H), 7.52-7.41 (m, 5H), 7.38 (t, *J* = 7.2 Hz, 1H), 7.22 (t, *J* = 8.0 Hz, 1H), 5.81 (t, *J* = 6.7 Hz, 1H), 5.24-5.03 (m, 2H), 4.27 (t, *J* = 12.0 Hz, 1H), 3.87-3.62 (m, 2H), 3.39 (dd, *J* = 19.0, 11.6 Hz, 1H), 2.93 (dd, *J* = 19.0, 9.6 Hz, 1H), 0.77 (t, *J* = 7.1 Hz, 3H); ^{13}C NMR (100 MHz, $CDCl_3$) δ 208.0, 205.1, 165.6, 141.8, 140.2, 138.4, 133.0, 131.5, 131.3, 130.0, 129.0, 128.6, 127.8, 127.7, 127.1, 126.8, 122.5, 117.1, 91.0, 80.9, 64.1, 63.2, 63.0, 46.0, 38.3, 13.5; IR (neat, cm^{-1}) 2959, 2922, 2852, 1950, 1751, 1733, 1563, 1473, 1413, 1230, 1106, 1001, 848, 763, 733, 696; HRMS (ESI): *m/z*: $[M+Na]^+$ calcd for $C_{30}H_{24}NO_3NaBr$: 548.0837, found: 548.0840.

The *ee* was determined by HPLC analysis: CHIRALPAK IG (4.6 mm i.d. × 250 mm); hexane/2-propanol = 90/10; flow rate 0.5 mL/min; 35 °C; 220 nm; retention time: 23.3 min (major) and 27.1 min (minor).

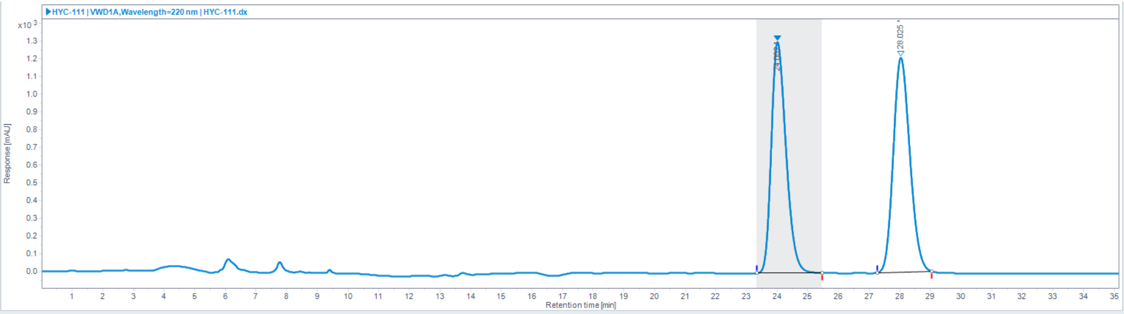

Injection Results

#	RT (min)	Area%	Height (mAU)	Start time (min)	End time (min)	S/N	Symmetry
1	24.002	48.732	1304.851	23.325	25.459		0.69758
2	28.025	51.268	1211.844	27.257	29.031		0.80680

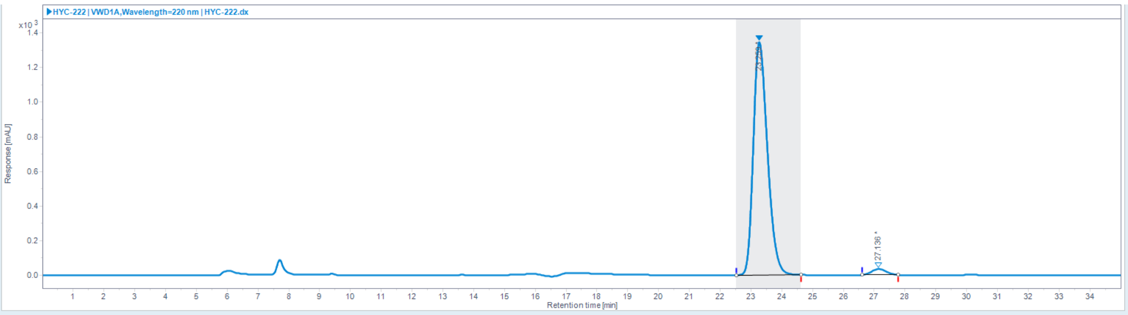

Injection Results

#	RT (min)	Area%	Height (mAU)	Start time (min)	End time (min)	S/N	Symmetry
1	23.259	97.444	1347.934	22.537	24.614		0.70029
2	27.136	2.556	33.970	26.596	27.765		0.92066

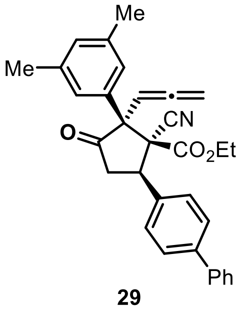

29

ethyl (1S,2S,5R)-5-([1,1'-biphenyl]-4-yl)-1-cyano-2-(3,5-dimethylphenyl)-3-oxo-2-(propa-1,2-dien-1-yl)cyclopentane-1-carboxylate (29): white solid; 28.5 mg, 60% yield; 95% *ee*; $[\alpha]_D^{20}$ = 17.4 (*c* = 0.115, $CHCl_3$); m.p. 114-116 °C; ^1H NMR (400 MHz, $CDCl_3$) δ 7.60 (t, *J* = 8.4 Hz, 4H), 7.51 (d, *J* = 8.1 Hz, 2H), 7.45 (t, *J* = 7.5 Hz, 2H), 7.37 (t, *J* = 7.2 Hz, 1H), 7.19 (s, 2H), 6.92 (s, 1H), 5.87 (t, *J* = 6.6 Hz, 1H), 5.19-5.00 (m, 2H), 4.27 (t, *J* = 10.6 Hz, 1H), 3.84-3.59 (m, 2H), 3.41 (dd, *J* = 18.8, 11.7 Hz, 1H), 2.92 (dd, *J* = 18.8, 9.6 Hz, 1H), 2.31 (s, 6H), 0.75 (t, *J* = 7.1 Hz, 3H); ^{13}C NMR (100 MHz, $CDCl_3$) δ 207.7, 206.1, 165.8, 141.6, 140.3, 137.9, 135.9, 133.4, 129.9, 129.0, 128.7, 127.8, 127.6, 127.1, 125.8, 117.5, 91.6, 80.4, 64.8 63.2, 62.8, 46.0, 38.7, 21.6, 13.4; IR (neat, cm^{-1}) 2956, 2921, 2853, 1952, 1751, 1602, 1487, 1463, 1231, 849, 764, 731, 701; HRMS (ESI): *m/z*: $[M+Na]^+$ calcd for $C_{32}H_{29}NO_3Na$: 498.2045, found: 498.2044.

The *ee* was determined by HPLC analysis: CHIRALPAK ADH (4.6 mm i.d. × 250 mm); hexane/2-propanol = 90/10; flow rate 0.5 mL/min; 35 °C; 220 nm; retention time: 31.1 min (major) and 25.5 min (minor).

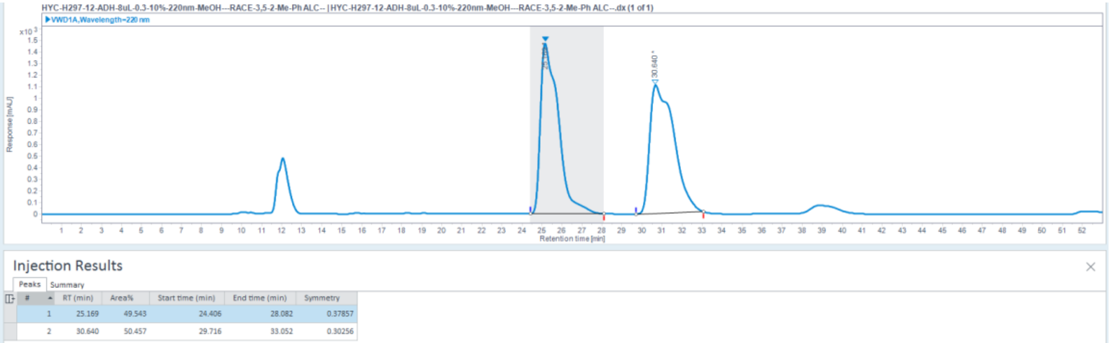

#	RT (min)	Area%	Start time (min)	End time (min)	Symmetry
1	25.169	49.543	24.406	28.082	0.37857
2	30.640	50.457	29.716	33.052	0.30256

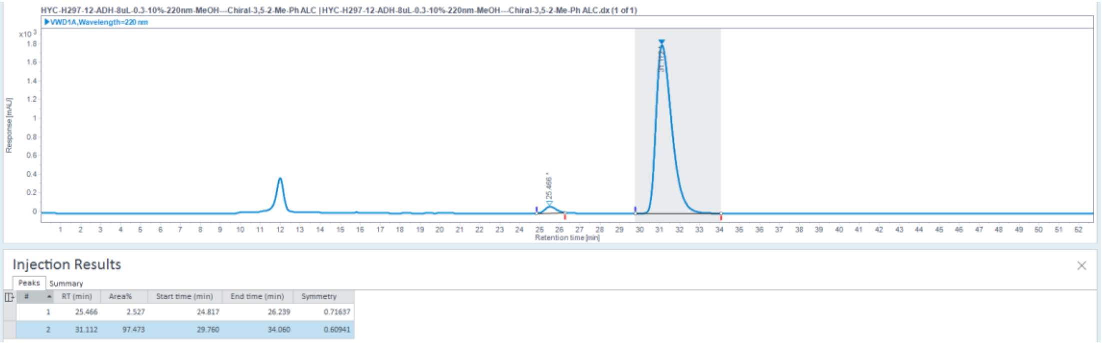

#	RT (min)	Area%	Start time (min)	End time (min)	Symmetry
1	25.466	2.527	24.817	26.239	0.71637
2	31.112	97.473	29.760	34.060	0.60941

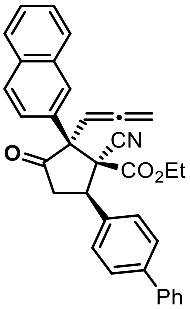

30

ethyl (1S,2S,5R)-5-([1,1'-biphenyl]-4-yl)-1-cyano-2-(naphthalen-2-yl)-3-oxo-2-(propa-1,2-dien-1-yl)cyclopentane-1-carboxylate (30): white solid; 32.3 mg, 65% yield; 93% *ee*; $[\alpha]_D^{20}$ = -51.3 (*c* = 0.115, $CHCl_3$); m.p. 112-113 °C; ^{1}H NMR (400 MHz, $CDCl_3$) δ 8.08 (s, 1H), 7.93-7.72 (m, 4H), 7.62 (dd, *J* = 14.0, 7.9 Hz, 4H), 7.54 (d, *J* = 8.1 Hz, 2H), 7.51-7.42 (m, 4H), 7.37 (t, *J* = 7.3 Hz, 1H), 5.98 (t, *J* = 6.7 Hz, 1H), 5.28-5.03 (m, 2H), 4.36 (t, *J* = 10.6 Hz, 1H), 3.69-3.54 (m, 2H), 3.49 (dd, *J* = 18.9, 11.7 Hz, 1H), 3.00 (dd, *J* = 18.9, 9.6 Hz, 1H), 0.59 (t, *J* = 7.1 Hz, 3H); ^{13}C NMR (100 MHz, $CDCl_3$) δ 208.1, 205.9, 165.7, 141.7, 140.3, 133.6, 133.3, 132.9, 132.8, 129.0, 128.69, 128.66, 128.1, 127.8, 127.7, 127.6, 127.5, 127.1, 126.7, 126.3, 125.5, 117.5, 91.5, 80.6, 64.8, 63.2, 62.9, 46.0, 38.6, 13.3; IR (neat, cm^{-1}) 2959, 2922, 2852, 1950, 1748, 1487, 1463, 1312, 1260, 1099, 1012, 850, 802, 734, 698; HRMS (ESI): *m/z*: $[M+Na]^+$ calcd for $C_{34}H_{27}NO_3Na$: 520.1889, found: 520.1892.

The *ee* was determined by HPLC analysis: CHIRALPAK ODH (4.6 mm i.d. × 250 mm); hexane/2-propanol = 90/10; flow rate 0.5 mL/min; 35 °C; 220 nm; retention time: 37.6 min (major) and 32.9 min (minor).

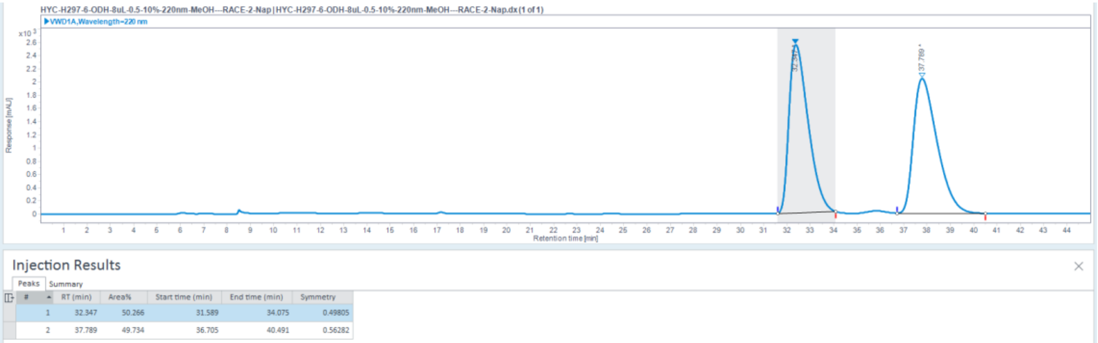

#	RT (min)	Area%	Start time (min)	End time (min)	Symmetry
1	32.347	50.266	31.589	34.075	0.49805
2	37.789	49.734	36.705	40.491	0.56282

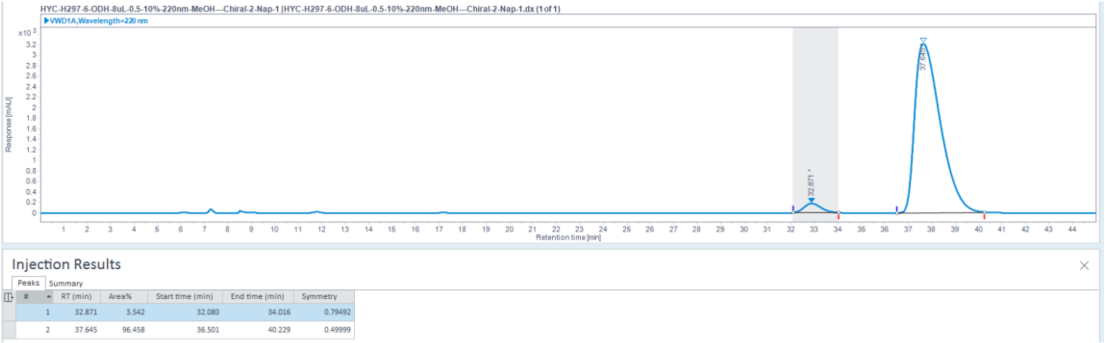

#	RT (min)	Area%	Start time (min)	End time (min)	Symmetry
1	32.871	3.542	32.080	34.016	0.79492
2	37.645	96.458	36.501	40.229	0.49999

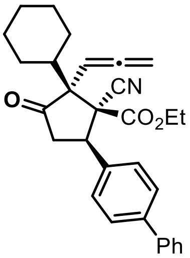

31

ethyl (1S,2R,5R)-5-([1,1'-biphenyl]-4-yl)-1-cyano-2-cyclohexyl-3-oxo-2-(propa-1,2-dien-1-yl)cyclopentane-1-carboxylate (31): white solid; 40.0 mg, 88% yield; 99% *ee*; $[\alpha]_D^{20}$ = 230.0 (*c* = 0.110, $CHCl_3$); m.p. 132-134 °C; ^{1}H NMR (400 MHz, $CDCl_3$) δ 7.60 (t, *J* = 7.4 Hz, 4H), 7.52-7.40 (m, 4H), 7.37 (t, *J* = 7.3 Hz, 1H), 5.61 (t, *J* = 6.7 Hz, 1H), 5.11-4.84 (m, 2H), 4.18-3.96 (m, 2H), 3.96-3.81 (m, 1H), 3.07 (dd, *J* = 18.9, 11.4 Hz, 1H), 2.69 (dd, *J* = 18.9, 9.9 Hz, 1H), 2.52 (d, *J* = 12.6 Hz, 1H), 2.05-1.96 (m, 1H), 1.84-1.72 (m, 2H), 1.69-1.60 (m, 2H), 1.40-1.30 (m, 2H), 1.21-1.09 (m, 3H), 0.98 (t, *J* = 7.1 Hz, 3H); ^{13}C NMR (100 MHz, $CDCl_3$) δ 209.0, 208.0, 166.2, 141.6, 140.3, 133.2, 129.0, 128.7, 127.7, 127.5, 127.1, 117.9, 86.8, 78.3, 65.5, 62.9, 60.4, 47.0, 41.2, 38.4, 29.4, 29.2, 26.8, 26.4, 26.2, 13.7; IR (neat, cm^{-1}) 2959, 2925, 2853, 1953, 1752, 1730, 1488, 1449, 1260, 1090, 1011, 846, 801, 763, 733, 698; HRMS (ESI): *m/z*: $[M+Na]^+$ calcd for $C_{30}H_{31}NO_3Na$: 476.2202, found: 476.2203.

The *ee* was determined by HPLC analysis: CHIRALPAK ODH (4.6 mm i.d. × 250 mm); hexane/2-propanol = 90/10; flow rate 0.5 mL/min; 35 ^{o}C; 220 nm; retention time: 15.1 min (major) and 16.3 min (minor).

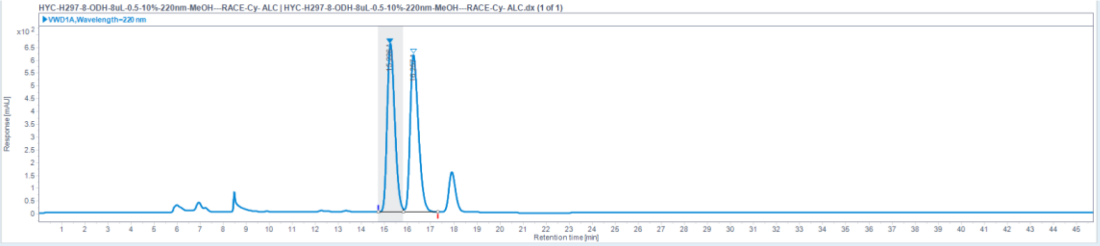

Injection Results

Peaks Summary

#	RT (min)	Area%	Start time (min)	End time (min)	Symmetry
1	15.226	49.604	14.711	15.805	0.64009
2	16.257	50.396	15.805	17.295	0.75697

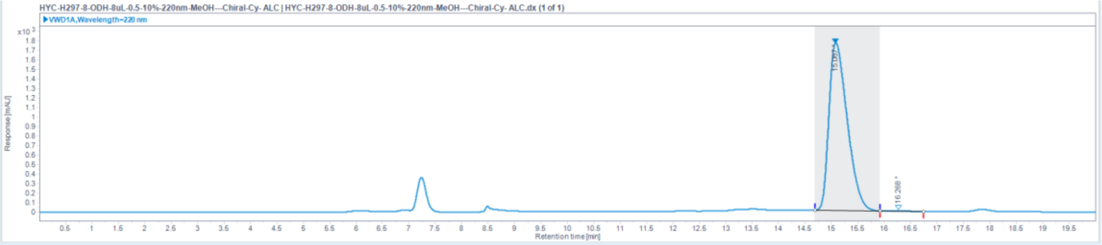

Injection Results

Peaks Summary

#	RT (min)	Area%	Start time (min)	End time (min)	Symmetry
1	15.067	99.721	14.682	15.915	0.48539
2	16.268	0.279	15.915	16.737	0.52668

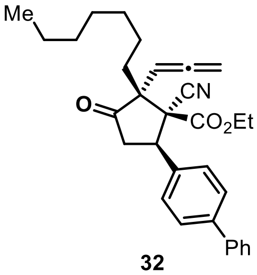

32

ethyl (1S,2R,5R)-5-([1,1'-biphenyl]-4-yl)-1-cyano-2-heptyl-3-oxo-2-(propa-1,2-dien-1-yl)cyclopentane-1-carboxylate (32): colorless oil; 30.5 mg, 65% yield; 77% *ee*; $[\alpha]_D^{20}$ = 162.0 (*c* = 0.110, $CHCl_3$); ^{1}H NMR (400 MHz, $CDCl_3$) δ 7.66-7.55 (m, 4H), 7.51 (d, *J* = 8.3 Hz, 2H), 7.45 (t, *J* = 7.6 Hz, 2H), 7.36 (t, *J* = 7.3 Hz, 1H), 5.39 (t, *J* = 6.7 Hz, 1H), 4.97 (dd, *J* = 6.7, 2.3 Hz, 2H), 4.36-4.14 (m, 3H), 2.95 (dd, *J* = 19.2, 12.7 Hz, 1H), 2.82 (dd, *J* = 19.2, 8.6 Hz, 1H), 2.18-2.06 (m, 1H), 1.99-1.84 (m, 1H), 1.58-1.38 (m, 2H), 1.36-1.21 (m, 11H), 0.89 (t, *J* = 6.7 Hz, 3H); ^{13}C NMR (100 MHz, $CDCl_3$) δ 209.5, 208.2, 165.5, 141.6, 140.4, 133.7, 129.2, 129.0, 127.7, 127.5, 127.2, 115.9, 87.2, 78.8, 63.4, 63.1, 62.1, 44.6, 39.8, 34.1, 31.9, 30.2, 29.2, 23.8, 22.8, 14.2, 14.0; IR (neat, cm^{-1}) 2954, 2925, 2855, 1953, 1744, 1488, 1462, 1250, 1167, 1026, 848, 767, 735, 697; HRMS (ESI): *m/z*: $[M+Na]^+$ calcd for $C_{31}H_{35}NO_3Na$: 492.2515, found: 492.2512.

The *ee* was determined by HPLC analysis: CHIRALPAK ADH (4.6 mm i.d. × 250 mm); hexane/2-propanol = 90/10; flow rate 0.3 mL/min; 35 °C; 220 nm; retention time: 27.3 min (major) and 30.5 min (minor).

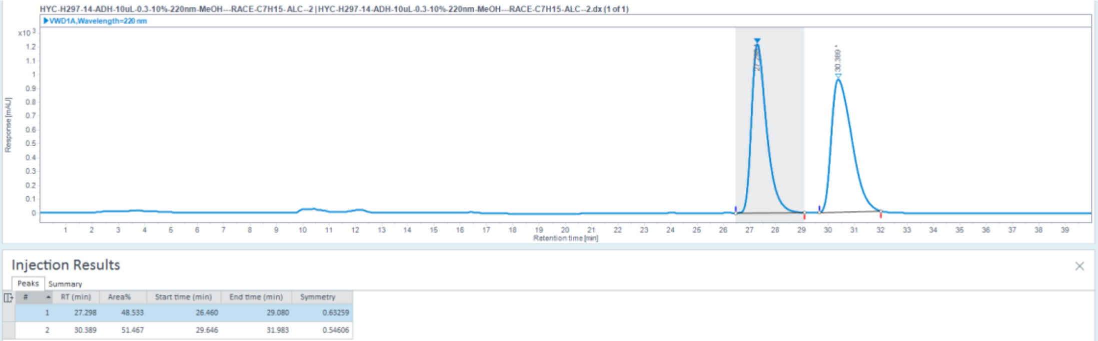

#	RT (min)	Area%	Start time (min)	End time (min)	Symmetry
1	27.298	48.533	26.460	29.080	0.63259
2	30.389	51.467	29.646	31.983	0.54606

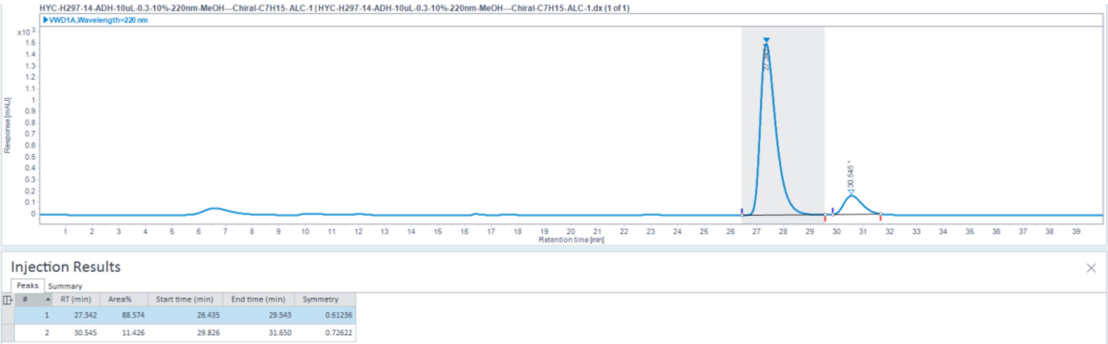

#	RT (min)	Area%	Start time (min)	End time (min)	Symmetry
1	27.342	88.574	26.435	29.543	0.61236
2	30.545	11.426	29.826	31.650	0.72622

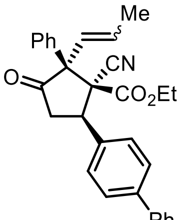

33

ethyl (1S,2S,5R)-5-([1,1'-biphenyl]-4-yl)-1-cyano-3-oxo-2-phenyl-2-(prop-1-en-1-yl)cyclopentane-1-carboxylate (33): white solid; 42.7 mg, 95% yield; m.p. 69-70 °C; ^{1}H NMR (400 MHz, $CDCl_3$) δ 7.64-7.51 (m, 8H), 7.45 (t, *J* = 7.6 Hz, 2H), 7.39-7.27 (m, 4H), 6.20 (dd, *J* = 11.0, 1.6 Hz, 1H), 6.13-6.01 (m, 1H), 4.23 (t, *J* = 10.7 Hz, 1H), 3.69-3.54 (m, 2H), 3.38 (dd, *J* = 18.8, 11.4 Hz, 1H), 3.01 (dd, *J* = 18.8, 10.1 Hz, 1H), 1.31 (dd, *J* = 7.2, 1.4 Hz, 3H), 0.66 (t, *J* = 7.1 Hz, 3H); ^{13}C NMR (100 MHz, $CDCl_3$) δ 204.1, 165.5, 141.5, 140.3, 137.4, 136.5, 133.5, 129.2, 129.0, 128.7, 128.2, 127.9, 127.7, 127.6, 127.1, 125.3, 117.8, 66.4, 65.0, 62.7, 45.4, 38.1, 15.1, 13.4; IR (neat, cm^{-1}) 2931, 1741, 1640, 1489, 1446, 1370, 1316, 1265, 1235, 1178, 1038, 846, 733, 697; HRMS (ESI): *m/z*: $[M+Na]^+$ calcd for $C_{30}H_{27}NO_3Na$: 472.1889, found: 472.1887.

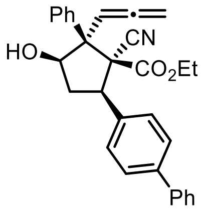

34

ethyl (1S,2S,5R)-5-([1,1'-biphenyl]-4-yl)-1-cyano-3-hydroxy-2-phenyl-2-(propa-1,2-dien-1-yl)cyclopentane-1-carboxylate (34): white solid; 38.2 mg, 85% yield; m.p. 175-177 °C; ^{1}H NMR (400 MHz, $CDCl_3$) δ 7.62 (t, *J* = 7.7 Hz, 4H), 7.53-7.44 (m, 4H), 7.41-7.32 (m, 5H), 7.27 (m, 1H), 5.70 (t, *J* = 6.6 Hz, 1H), 5.17-5.01 (m, 2H), 5.02-4.89 (m, 1H), 4.67 (d, *J* = 12.7 Hz, 1H), 4.11-3.92 (m, 2H), 3.93-3.67 (m, 1H), 3.07-2.79 (m, 1H), 2.67-2.40 (m, 1H), 0.93 (t, *J* = 7.1 Hz, 3H); ^{13}C NMR (100 MHz, $CDCl_3$) δ 206.3, 169.4, 141.6, 140.5, 139.7, 133.9, 129.0, 128.9, 128.5, 128.2, 127.7, 127.5, 127.2, 127.1, 118.5, 98.2, 79.8, 76.2, 63.7, 63.2, 62.8, 53.2, 38.6, 13.5; IR (neat, cm^{-1}) 3498, 3360, 2937, 2922, 2832, 1951, 1719, 1442, 1320, 1232, 1083, 1004, 842, 763, 734, 696, 644; HRMS (ESI): *m/z*: $[M+Na]^+$ calcd for $C_{30}H_{27}NO_3Na$: 472.1889, found: 472.1888.

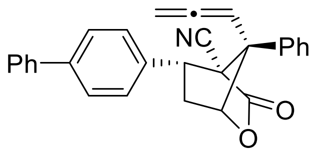

35

(1R,4S,5R,7R)-5-([1,1'-biphenyl]-4-yl)-3-oxo-7-phenyl-7-(propa-1,2-dien-1-yl)-2-oxabicyclo[2.2.1]heptane-4-carbonitrile (35): white solid; 30.6 mg, 76% yield; m.p. 121-123 °C; ^{1}H NMR (400 MHz, $CDCl_3$) δ 7.66-7.32 (m, 15H), 5.62 (t, *J* = 6.7 Hz, 1H), 5.26 (s, 1H), 5.15 (d, *J* = 6.8 Hz, 2H), 4.25 (dd, *J* = 10.6, 5.3 Hz, 1H), 2.95-2.84 (m, 1H), 2.49 (dd, *J* = 14.2, 5.3 Hz, 1H); ^{13}C NMR (100 MHz, $CDCl_3$) δ 207.6, 167.1, 141.8, 140.4, 136.2, 133.9, 129.3, 129.1, 128.9, 128.7, 127.8, 127.7, 127.4, 127.3, 114.9, 90.1, 84.6, 80.4, 64.4, 59.0, 47.4, 33.4; IR (neat, cm^{-1}) 2956, 2923, 2853, 1796, 1741, 1707, 1462, 1377, 1262, 1093, 1079, 1013, 800, 738, 698; HRMS (ESI): *m/z*: $[M+Na]^+$ calcd for $C_{28}H_{21}NO_2Na$: 426.1470, found: 426.1463.

X-ray crystallographic information of product 3

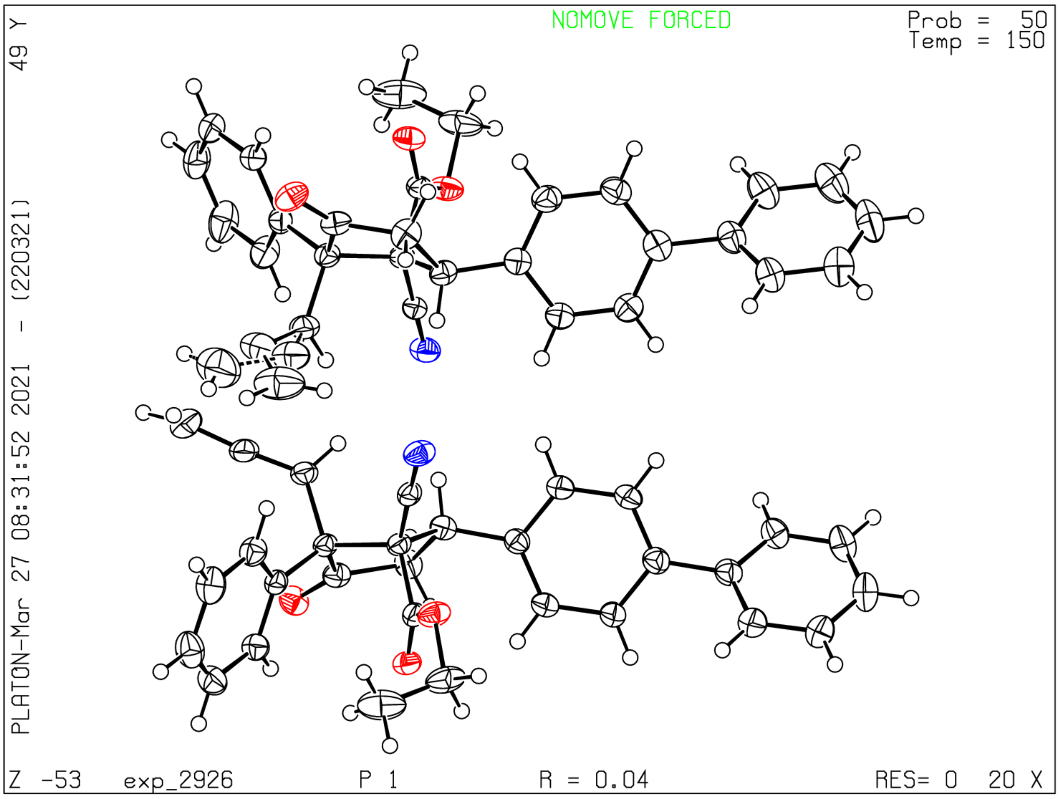

X-ray crystallography of **3**

Table S2 Crystal data and structure refinement for 3.

Identification code	**3**
Empirical formula	$C_{30}H_{25}NO_3$
Formula weight	447.51
Temperature/K	149.99(10)
Crystal system	triclinic
Space group	P1
a/Å	9.9067(2)
b/Å	11.5214(3)
c/Å	11.6032(3)
α/°	93.702(2)
β/°	109.012(2)
γ/°	103.102(2)
Volume/Å^3	1205.68(5)
Z	2
ρ_{calc}g/cm^3	1.233
μ/mm^{-1}	0.630
F(000)	472.0
Crystal size/mm^3	0.08 × 0.10 × 0.13
Radiation	Cu Kα (λ = 1.54184)
2Θ range for data collection/°	7.97 to 147.69
Index ranges	$-12 \leq h \leq 11$, $-14 \leq k \leq 14$, $-14 \leq l \leq 13$
Reflections collected	8318

Independent reflections	5676 [R_{int} = 0.0204, R_{sigma} = 0.0266]
Data/restraints/parameters	5676/50/644
Goodness-of-fit on F^2	1.040
Final R indexes [I>=2σ (I)]	R_1 = 0.0392, wR_2 = 0.1031
Final R indexes [all data]	R_1 = 0.0403, wR_2 = 0.1045
Largest diff. peak/hole / e Å^{-3}	0.55/-0.68
Flack/Hooft parameter	0.0 (2)

Crystal structure determination of 3

Crystal Data for $C_{30}H_{25}NO_3$ (*M* =447.51 g/mol): triclinic, space group P1 (no. 1), *a* = 9.9067(2) Å, *b* = 11.5214(3) Å, *c* = 11.6032(3) Å, *α* = 93.702(2)°, *β* = 109.012(2)°, *γ* = 103.102(2)°, *V* = 1205.68(5) Å^3, *Z* = 2, *T* = 149.99(10) K, μ(Cu Kα) = 0.630 mm^{-1}, *Dcalc* = 1.233 g/cm^3, 8318 reflections measured (7.97° ≤ 2Θ ≤ 147.69°), 5676 unique (R_{int} = 0.0204, R_{sigma} = 0.0266) which were used in all calculations. The final R_1 was 0.0392 (I > 2σ(I)) and wR_2 was 0.1045 (all data).

Refinement model description

Table S3 Fractional Atomic Coordinates (×10^4) and Equivalent Isotropic Displacement Parameters (Å^2×10^3) for 3. U_{eq} is defined as 1/3 of of the trace of the orthogonalised U_{IJ} tensor.

Atom	*x*	*y*	*z*	U(eq)
O1	6468(2)	7340.9(17)	7010.6(18)	30.7(4)
O2	8876(2)	7399.2(17)	7438(2)	34.1(5)
O3	4867(2)	9842.2(18)	6681.8(19)	33.5(4)
N1	10753(3)	9652(2)	6586(3)	35.1(5)
C00J	7997(3)	8946(2)	6433(2)	23.6(5)
C1	6944(3)	7646(2)	4251(2)	26.3(5)
C2	6124(3)	6489(3)	4256(3)	31.9(6)
C3	6128(3)	5503(3)	3516(3)	31.1(6)
C4	6935(3)	5633(3)	2718(3)	30.6(6)
C5	7733(3)	6788(3)	2696(3)	32.7(6)
C6	7740(3)	7772(2)	3446(3)	30.5(6)
C7	6941(3)	4559(3)	1946(3)	30.7(6)
C8	7179(3)	3513(3)	2452(3)	35.2(6)
C9	7240(4)	2536(3)	1742(3)	38.3(7)
C10	7037(4)	2557(3)	507(3)	45.1(8)

Table S3 Fractional Atomic Coordinates (×10^4) and Equivalent Isotropic Displacement Parameters (Å^2×10^3) for 3. U_{eq} is defined as 1/3 of of the trace of the orthogonalised U_{IJ} tensor.

**Atom**	***x***	***y***	***z***	**U(eq)**
C11	6770(4)	3567(3)	-4(3)	42.4(7)
C12	6729(3)	4560(3)	703(3)	35.2(6)
C13	6971(3)	8756(2)	5034(2)	27.3(5)
C14	5479(3)	8860(3)	5083(3)	31.1(6)
C15	5788(3)	9556(2)	6337(3)	27.9(5)
C16	7468(3)	9918(2)	7077(2)	24.2(5)
C17	7909(3)	9951(2)	8466(2)	25.0(5)
C18	6920(3)	9403(2)	9013(3)	31.3(6)
C19	7383(4)	9411(3)	10286(3)	39.7(7)
C20	8830(4)	9954(3)	11020(3)	43.5(7)
C21	9828(4)	10484(3)	10481(3)	42.3(7)
C22	9383(3)	10493(3)	9223(3)	33.1(6)
C23	9557(3)	9342(2)	6549(2)	25.9(5)
C24	8001(3)	11142(2)	6704(3)	28.9(6)
C25	8292(4)	12182(3)	7343(3)	37.3(7)
C26	8594(7)	13257(3)	7907(5)	63.9(13)
C27	7684(3)	7793(2)	6996(2)	23.9(5)
C28	8719(4)	6346(3)	8076(4)	45.9(8)
C29	9002(5)	6747(4)	9397(4)	63.1(11)
O4	15080(2)	14376.8(18)	7355(2)	36.3(5)
O5	13437(2)	13945.4(18)	4097(2)	33.6(4)
O6	11080(2)	13055.6(19)	2896.9(19)	36.4(5)
N2	9212(3)	11246(2)	4215(2)	32.9(5)
C31	13144(3)	8540(3)	1101(3)	36.7(6)
C32	13529(4)	7474(3)	1336(3)	42.3(7)

Table S3 Fractional Atomic Coordinates (×10^4) and Equivalent Isotropic Displacement Parameters (Å^2×10^3) for 3. U_{eq} is defined as 1/3 of of the trace of the orthogonalised U_{IJ} tensor.

Atom	*x*	*y*	*z*	U(eq)
C33	13641(5)	6717(4)	409(4)	53.2(9)
C34	13359(5)	7021(4)	-764(4)	58.0(10)
C35	12979(5)	8082(4)	-1009(3)	56.1(10)
C36	12884(4)	8841(4)	-83(3)	45.8(8)
C37	13044(3)	9359(3)	2104(3)	33.2(6)
C38	12174(3)	8940(3)	2793(3)	32.6(6)
C39	12152(3)	9682(3)	3773(3)	29.2(5)
C40	13006(3)	10885(2)	4091(3)	27.8(5)
C41	13847(3)	11309(3)	3376(3)	36.0(6)
C42	13864(3)	10559(3)	2394(3)	36.4(6)
C43	13007(3)	11649(2)	5201(3)	27.7(5)
C44	14501(3)	12481(3)	6045(3)	34.0(6)
C45	14166(3)	13555(2)	6601(3)	29.5(6)
C46	12481(3)	13414(2)	6143(2)	25.9(5)
C47	11960(3)	12521(2)	4896(2)	24.2(5)
C48	10409(3)	11825(2)	4501(2)	24.3(5)
C49	12247(3)	13261(2)	3916(2)	25.9(5)
C50	11248(4)	13784(4)	1937(3)	52.2(9)
C51	11019(5)	14992(4)	2203(5)	68.4(13)
C52	11964(3)	14560(2)	5974(2)	27.2(5)
C53	10461(3)	14486(3)	5708(3)	39.7(7)
C54	9945(4)	15499(3)	5527(4)	49.2(8)
C55	10882(4)	16601(3)	5599(3)	44.2(8)
C56	12373(4)	16685(3)	5864(3)	37.6(7)
C57	12915(3)	15679(2)	6047(3)	30.2(6)

Table S3 Fractional Atomic Coordinates (×10^4) and Equivalent Isotropic Displacement Parameters (Å^2×10^3) for 3. U_{eq} is defined as 1/3 of of the trace of the orthogonalised U_{IJ} tensor.

Atom	*x*	*y*	*z*	U(eq)
C58	11925(3)	12743(3)	7069(3)	34.7(6)
C59	12491(8)	12943(8)	8252(7)	51.1(16)
C60	13290(9)	12086(8)	8948(8)	70(2)
C61	12905(13)	13116(11)	9294(9)	84(3)
C62	12640(9)	12413(8)	8051(7)	47.1(17)

Table S4 Anisotropic Displacement Parameters (Å^2×10^3) for 3. The Anisotropic displacement factor exponent takes the form: $-2\pi^2[h^2a^{*2}U_{11}+2hka^*b^*U_{12}+…]$.

Atom	U_{11}	U_{22}	U_{33}	U_{23}	U_{13}	U_{12}
O1	30.0(10)	26.0(9)	35.8(10)	6.2(8)	14.2(8)	2.7(8)
O2	32.6(10)	26.1(9)	49.9(12)	15.9(9)	17.4(9)	12.5(8)
O3	28.7(10)	39.2(11)	39.9(11)	9.0(9)	16.0(9)	16.5(9)
N1	28.6(13)	28.3(12)	54.4(15)	11.2(11)	20.4(11)	9.3(9)
C00J	23.4(12)	19.9(11)	29.0(13)	4.7(9)	10.9(10)	5.8(9)
C1	24.3(13)	29.0(13)	25.8(13)	4.3(10)	9.2(10)	6.7(10)
C2	31.5(14)	32.5(14)	34.1(14)	4.9(11)	17.8(12)	3.7(11)
C3	30.8(14)	29.6(13)	33.2(14)	0.9(11)	16.1(12)	2.5(11)
C4	27.9(13)	32.9(14)	31.6(14)	3.7(11)	12.4(11)	6.7(11)
C5	33.6(15)	33.5(14)	37.1(15)	7.2(12)	21.3(12)	7.2(11)
C6	31.7(14)	29.2(13)	33.5(14)	7.1(11)	16.5(12)	5.5(11)
C7	25.3(13)	33.8(14)	33.4(15)	3.1(11)	12.6(11)	5.6(11)
C8	35.2(15)	35.4(15)	34.7(15)	5.1(12)	11.5(12)	9.6(12)
C9	39.8(16)	33.5(15)	39.5(16)	2.6(12)	10.3(13)	11.7(13)
C10	50(2)	45.7(18)	40.1(18)	-5.9(14)	13.6(15)	20.3(15)

Table S4 Anisotropic Displacement Parameters (Å^2×10^3) for 3. The Anisotropic displacement factor exponent takes the form: $-2\pi^2[h^2a^{*2}U_{11}+2hka^*b^*U_{12}+…]$.

Atom	U_{11}	U_{22}	U_{33}	U_{23}	U_{13}	U_{12}
C11	48.4(19)	52.5(19)	29.9(15)	1.6(13)	13.7(13)	21.4(15)
C12	35.7(15)	40.1(15)	33.8(15)	5.4(12)	14.5(12)	14.3(12)
C13	27.1(13)	28.8(12)	28.6(13)	7.6(10)	11.7(11)	8.6(10)
C14	24.5(13)	38.2(15)	30.4(14)	3.8(11)	7.9(11)	10.9(11)
C15	27.3(13)	26.2(12)	34.4(14)	10.1(11)	12.7(11)	11.0(10)
C16	25.6(12)	19.9(11)	30.8(13)	6.2(10)	12.6(10)	8.3(9)
C17	27.5(13)	20.3(11)	29.2(13)	3.1(9)	11.3(11)	8.2(10)
C18	32.9(14)	30.3(13)	32.2(14)	6.1(11)	13.2(12)	8.5(11)
C19	50.9(19)	39.8(16)	34.5(15)	9.1(13)	21.1(14)	13.9(14)
C20	53(2)	50.0(18)	30.5(15)	6.0(13)	11.5(14)	24.3(16)
C21	37.6(17)	46.1(17)	34.6(16)	-2.4(13)	1.2(13)	13.9(14)
C22	28.2(14)	31.7(14)	36.8(15)	0.7(12)	9.9(12)	6.4(11)
C23	28.7(14)	19.5(11)	33.1(13)	5.2(10)	13.7(11)	8.9(10)
C24	32.1(14)	23.3(12)	37.2(15)	9.4(11)	17.8(12)	8.7(10)
C25	43.2(17)	25.6(13)	58.6(19)	12.6(13)	35.4(15)	11.2(12)
C26	94(4)	28.0(17)	87(3)	5.5(18)	61(3)	7.3(19)
C27	25.1(13)	20.8(11)	26.7(12)	2.5(9)	10.0(10)	6.5(10)
C28	45.4(18)	32.7(15)	69(2)	27.7(16)	23.8(17)	17.5(13)
C29	60(2)	64(2)	66(3)	39(2)	20(2)	13(2)
O4	27.7(10)	30.5(10)	39.2(11)	4.8(9)	0.7(8)	1.4(8)
O5	26.4(10)	35.8(10)	40.6(11)	11.3(9)	16.2(8)	4.1(8)
O6	33.6(11)	42.0(11)	29.4(10)	14.9(9)	9.1(9)	2.1(9)
N2	29.0(13)	36.9(13)	29.0(12)	6.6(10)	9.9(10)	1.0(10)
C31	28.1(15)	47.1(17)	33.2(15)	-0.5(13)	10.2(12)	9.1(12)
C32	44.8(18)	49.6(19)	33.6(16)	2.4(14)	13.8(14)	15.6(15)

Table S4 Anisotropic Displacement Parameters (Å^2 ×10^3) for 3. The Anisotropic displacement factor exponent takes the form: $-2\pi^2[h^2a^{*2}U_{11}+2hka^*b^*U_{12}+\ldots]$.

**Atom**	U_{11}	U_{22}	U_{33}	U_{23}	U_{13}	U_{12}
C33	57(2)	54(2)	49(2)	-2.4(16)	18.0(17)	19.4(18)
C34	60(2)	74(3)	41.6(19)	-10.5(17)	21.9(17)	19(2)
C35	57(2)	78(3)	34.6(18)	2.4(17)	17.3(16)	19(2)
C36	45.7(19)	63(2)	32.5(16)	7.3(14)	13.8(14)	22.5(16)
C37	29.1(14)	40.3(15)	31.0(15)	5.7(12)	8.2(12)	13.6(12)
C38	25.7(13)	33.7(14)	34.6(15)	3.5(11)	7.2(11)	5.7(11)
C39	22.2(12)	33.5(13)	32.4(14)	7.0(11)	9.4(11)	7.9(10)
C40	24.4(13)	30.2(13)	30.8(13)	7.2(10)	9.0(11)	11.1(10)
C41	32.2(15)	32.3(14)	47.4(17)	8.1(12)	18.8(13)	8.4(12)
C42	34.1(15)	40.7(16)	39.4(16)	10.1(13)	17.8(13)	11.2(12)
C43	25.4(13)	25.2(12)	34.3(14)	7.6(10)	10.6(11)	8.9(10)
C44	24.2(13)	36.0(14)	36.9(15)	3.6(12)	4.0(11)	8.8(11)
C45	24.5(13)	28.6(13)	31.9(14)	9.6(11)	6.9(11)	3.5(10)
C46	22.0(13)	25.4(12)	27.7(13)	2.8(10)	7.8(10)	3.0(10)
C47	21.4(12)	24.7(12)	26.7(13)	5.3(10)	9.1(10)	5.0(10)
C48	26.4(13)	23.8(11)	23.4(12)	5.4(9)	10.1(10)	5.6(10)
C49	24.7(13)	26.3(12)	30.3(13)	6.4(10)	13.6(11)	7.0(10)
C50	48.4(19)	65(2)	37.0(17)	28.4(17)	12.6(15)	1.9(17)
C51	54(2)	73(3)	88(3)	56(3)	26(2)	20(2)
C52	27.8(13)	26.9(12)	26.2(12)	0.3(10)	9.7(10)	6.4(10)
C53	28.8(15)	35.7(15)	52.3(19)	-2.6(14)	13.8(14)	7.3(12)
C54	33.9(16)	49.1(19)	60(2)	-9.3(16)	8.5(15)	19.0(14)
C55	54(2)	38.1(16)	44.0(17)	1.0(13)	13.9(15)	24.9(15)
C56	48.8(18)	27.3(14)	39.3(16)	2.9(12)	18.9(14)	10.4(13)
C57	32.8(14)	27.6(13)	32.8(14)	5.3(11)	15.2(12)	7.4(11)

Table S4 Anisotropic Displacement Parameters (Å^2×10^3) for 3. The Anisotropic displacement factor exponent takes the form: $-2\pi^2[h^2a^{*2}U_{11}+2hka^*b^*U_{12}+\ldots]$.

Atom	U_{11}	U_{22}	U_{33}	U_{23}	U_{13}	U_{12}
C58	33.9(14)	31.1(13)	35.3(12)	4.6(11)	14.5(11)	-2.4(11)
C59	47(2)	60(3)	42.3(16)	12.5(17)	18.0(15)	0.7(17)
C60	55(3)	79(4)	62(3)	32(3)	10(3)	1(3)
C61	78(4)	97(4)	53(3)	-4(3)	22(3)	-16(4)
C62	45(2)	47(2)	46(2)	15.2(18)	15.3(15)	4.2(18)

Table S5 Bond Lengths for 3.

Atom	Atom	Length/Å	Atom	Atom	Length/Å
O1	C27	1.205(3)	O5	C49	1.205(3)
O2	C27	1.320(3)	O6	C49	1.319(3)
O2	C28	1.466(3)	O6	C50	1.466(3)
O3	C15	1.207(3)	N2	C48	1.148(4)
N1	C23	1.143(4)	C31	C32	1.384(5)
C00J	C13	1.579(4)	C31	C36	1.395(5)
C00J	C16	1.581(3)	C31	C37	1.494(4)
C00J	C23	1.467(4)	C32	C33	1.389(5)
C00J	C27	1.531(3)	C33	C34	1.382(6)
C1	C2	1.396(4)	C34	C35	1.379(6)
C1	C6	1.401(4)	C35	C36	1.382(5)
C1	C13	1.510(4)	C37	C38	1.388(4)
C2	C3	1.380(4)	C37	C42	1.391(4)
C3	C4	1.401(4)	C38	C39	1.386(4)
C4	C5	1.392(4)	C39	C40	1.403(4)
C4	C7	1.482(4)	C40	C41	1.393(4)

Table S5 Bond Lengths for 3.

Atom	Atom	Length/Å	Atom	Atom	Length/Å
C5	C6	1.381(4)	C40	C43	1.512(4)
C7	C8	1.408(4)	C41	C42	1.392(4)
C7	C12	1.388(4)	C43	C44	1.536(4)
C8	C9	1.376(4)	C43	C47	1.578(3)
C9	C10	1.383(5)	C44	C45	1.509(4)
C10	C11	1.379(5)	C45	C46	1.544(4)
C11	C12	1.380(4)	C46	C47	1.581(4)
C13	C14	1.528(4)	C46	C52	1.524(3)
C14	C15	1.518(4)	C46	C58	1.535(4)
C15	C16	1.546(4)	C47	C48	1.466(4)
C16	C17	1.521(4)	C47	C49	1.525(3)
C16	C24	1.531(3)	C50	C51	1.488(6)
C17	C18	1.392(4)	C52	C53	1.400(4)
C17	C22	1.405(4)	C52	C57	1.393(4)
C18	C19	1.395(4)	C53	C54	1.379(5)
C19	C20	1.379(5)	C54	C55	1.371(5)
C20	C21	1.386(5)	C55	C56	1.386(5)
C21	C22	1.381(4)	C56	C57	1.384(4)
C24	C25	1.288(4)	C58	C59	1.286(8)
C25	C26	1.285(5)	C58	C62	1.264(8)
C28	C29	1.487(6)	C59	C60	1.515(8)
O4	C45	1.212(3)	C61	C62	1.519(8)

Table S6 Bond Angles for 3.

Atom	Atom	Atom	Angle/°	Atom	Atom	Atom	Angle/°
C27	O2	C28	116.4(2)	C32	C31	C36	118.5(3)
C13	C00J	C16	103.82(19)	C32	C31	C37	120.5(3)
C23	C00J	C13	110.1(2)	C36	C31	C37	120.9(3)
C23	C00J	C16	113.4(2)	C31	C32	C33	120.7(3)
C23	C00J	C27	111.5(2)	C34	C33	C32	120.1(4)
C27	C00J	C13	110.7(2)	C35	C34	C33	119.9(3)
C27	C00J	C16	107.1(2)	C34	C35	C36	120.0(4)
C2	C1	C6	117.5(2)	C35	C36	C31	120.8(3)
C2	C1	C13	123.4(2)	C38	C37	C31	121.2(3)
C6	C1	C13	119.1(2)	C38	C37	C42	118.4(3)
C3	C2	C1	121.2(3)	C42	C37	C31	120.4(3)
C2	C3	C4	121.1(3)	C39	C38	C37	121.2(3)
C3	C4	C7	120.2(3)	C38	C39	C40	120.8(3)
C5	C4	C3	117.8(3)	C39	C40	C43	119.2(2)
C5	C4	C7	122.0(3)	C41	C40	C39	117.8(3)
C6	C5	C4	121.0(3)	C41	C40	C43	123.0(3)
C5	C6	C1	121.4(3)	C42	C41	C40	121.1(3)
C8	C7	C4	121.1(3)	C37	C42	C41	120.7(3)
C12	C7	C4	120.9(3)	C40	C43	C44	117.0(2)
C12	C7	C8	118.0(3)	C40	C43	C47	114.9(2)
C9	C8	C7	120.8(3)	C44	C43	C47	103.6(2)
C8	C9	C10	120.3(3)	C45	C44	C43	106.6(2)
C11	C10	C9	119.2(3)	O4	C45	C44	124.9(3)
C10	C11	C12	121.0(3)	O4	C45	C46	124.6(3)
C11	C12	C7	120.6(3)	C44	C45	C46	110.3(2)
C1	C13	C00J	115.4(2)	C45	C46	C47	100.9(2)

Table S6 Bond Angles for 3.

Atom	Atom	Atom	Angle/°	Atom	Atom	Atom	Angle/°
C1	C13	C14	116.2(2)	C52	C46	C45	117.2(2)
C14	C13	C00J	104.1(2)	C52	C46	C47	113.0(2)
C15	C14	C13	106.8(2)	C52	C46	C58	110.8(2)
O3	C15	C14	125.0(3)	C58	C46	C45	105.5(2)
O3	C15	C16	124.8(3)	C58	C46	C47	108.6(2)
C14	C15	C16	110.0(2)	C43	C47	C46	103.6(2)
C15	C16	C00J	101.2(2)	C48	C47	C43	110.0(2)
C17	C16	C00J	113.61(19)	C48	C47	C46	113.4(2)
C17	C16	C15	116.0(2)	C48	C47	C49	111.3(2)
C17	C16	C24	113.2(2)	C49	C47	C43	110.5(2)
C24	C16	C00J	108.6(2)	C49	C47	C46	107.7(2)
C24	C16	C15	103.0(2)	N2	C48	C47	177.5(3)
C18	C17	C16	122.3(2)	O5	C49	O6	125.9(2)
C18	C17	C22	118.4(3)	O5	C49	C47	121.2(2)
C22	C17	C16	119.2(2)	O6	C49	C47	112.9(2)
C17	C18	C19	120.5(3)	O6	C50	C51	110.5(3)
C20	C19	C18	120.5(3)	C53	C52	C46	118.7(2)
C19	C20	C21	119.4(3)	C57	C52	C46	123.0(2)
C22	C21	C20	120.8(3)	C57	C52	C53	118.3(3)
C21	C22	C17	120.4(3)	C54	C53	C52	120.5(3)
N1	C23	C00J	177.0(3)	C55	C54	C53	121.2(3)
C25	C24	C16	126.2(3)	C54	C55	C56	118.8(3)
C26	C25	C24	175.6(4)	C57	C56	C55	121.0(3)
O1	C27	O2	126.6(2)	C56	C57	C52	120.2(3)
O1	C27	C00J	121.3(2)	C59	C58	C46	128.1(4)

Table S6 Bond Angles for 3.

Atom	Atom	Atom	Angle/°	Atom	Atom	Atom	Angle/°
O2	C27	C00J	112.1(2)	C62	C58	C46	130.0(4)
O2	C28	C29	109.6(3)	C58	C59	C60	120.3(8)
C49	O6	C50	116.0(2)	C58	C62	C61	120.0(9)

Table S7 Torsion Angles for 3.

A	B	C	D	Angle/°	A	B	C	D	Angle/°
O3	C15	C16	C00J	160.6(2)	C31	C32	C33	C34	-0.3(6)
O3	C15	C16	C17	37.2(4)	C31	C37	C38	C39	176.0(3)
O3	C15	C16	C24	-87.1(3)	C31	C37	C42	C41	-176.0(3)
C00J	C13	C14	C15	21.6(3)	C32	C31	C36	C35	1.4(5)
C00J	C16	C17	C18	-100.7(3)	C32	C31	C37	C38	-53.7(4)
C00J	C16	C17	C22	75.7(3)	C32	C31	C37	C42	124.2(3)
C00J	C16	C24	C25	-152.0(3)	C32	C33	C34	C35	0.5(6)
C1	C2	C3	C4	-1.1(4)	C33	C34	C35	C36	0.2(6)
C1	C13	C14	C15	149.7(2)	C34	C35	C36	C31	-1.1(6)
C2	C1	C6	C5	-0.8(4)	C36	C31	C32	C33	-0.6(5)
C2	C1	C13	C00J	78.6(3)	C36	C31	C37	C38	127.8(3)
C2	C1	C13	C14	-43.7(4)	C36	C31	C37	C42	-54.3(4)
C2	C3	C4	C5	0.0(4)	C37	C31	C32	C33	-179.2(3)
C2	C3	C4	C7	179.0(3)	C37	C31	C36	C35	179.9(3)
C3	C4	C5	C6	0.7(4)	C37	C38	C39	C40	0.4(4)
C3	C4	C7	C8	-47.0(4)	C38	C37	C42	C41	1.9(4)
C3	C4	C7	C12	133.7(3)	C38	C39	C40	C41	1.3(4)
C4	C5	C6	C1	-0.2(4)	C38	C39	C40	C43	-177.2(2)

Table S7 Torsion Angles for 3.

A	B	C	D	Angle/°	A	B	C	D	Angle/°
C4	C7	C8	C9	-177.5(3)	C39	C40	C41	C42	-1.3(4)
C4	C7	C12	C11	178.5(3)	C39	C40	C43	C44	138.5(2)
C5	C4	C7	C8	132.1(3)	C39	C40	C43	C47	-99.6(3)
C5	C4	C7	C12	-47.2(4)	C40	C41	C42	C37	-0.3(4)
C6	C1	C2	C3	1.5(4)	C40	C43	C44	C45	150.1(2)
C6	C1	C13	C00J	-103.3(3)	C40	C43	C47	C46	-166.1(2)
C6	C1	C13	C14	134.3(3)	C40	C43	C47	C48	72.4(3)
C7	C4	C5	C6	-178.4(3)	C40	C43	C47	C49	-50.9(3)
C7	C8	C9	C10	-1.4(5)	C41	C40	C43	C44	-39.8(4)
C8	C7	C12	C11	-0.8(4)	C41	C40	C43	C47	82.1(3)
C8	C9	C10	C11	-0.1(5)	C42	C37	C38	C39	-1.9(4)
C9	C10	C11	C12	1.1(5)	C43	C40	C41	C42	177.1(3)
C10	C11	C12	C7	-0.7(5)	C43	C44	C45	O4	176.9(3)
C12	C7	C8	C9	1.8(4)	C43	C44	C45	C46	0.9(3)
C13	C00J	C16	C15	35.5(2)	C43	C47	C49	O5	-61.7(3)
C13	C00J	C16	C17	160.6(2)	C43	C47	C49	O6	118.0(2)
C13	C00J	C16	C24	-72.5(3)	C44	C43	C47	C46	-37.1(3)
C13	C00J	C27	O1	-58.5(3)	C44	C43	C47	C48	-158.7(2)
C13	C00J	C27	O2	121.7(2)	C44	C43	C47	C49	78.0(3)
C13	C1	C2	C3	179.5(3)	C44	C45	C46	C47	-23.6(3)
C13	C1	C6	C5	-179.0(3)	C44	C45	C46	C52	-146.7(2)
C13	C14	C15	O3	177.4(3)	C44	C45	C46	C58	89.4(3)
C13	C14	C15	C16	1.1(3)	C45	C46	C47	C43	36.6(2)
C14	C15	C16	C00J	-23.1(3)	C45	C46	C47	C48	155.9(2)
C14	C15	C16	C17	-146.5(2)	C45	C46	C47	C49	-80.5(2)

Table S7 Torsion Angles for 3.

A	B	C	D	Angle/°	A	B	C	D	Angle/°
C14	C15	C16	C24	89.2(2)	C45	C46	C52	C53	-172.2(3)
C15	C16	C17	C18	16.0(3)	C45	C46	C52	C57	9.1(4)
C15	C16	C17	C22	-167.6(2)	C45	C46	C58	C59	45.3(6)
C15	C16	C24	C25	101.2(3)	C45	C46	C58	C62	3.9(7)
C16	C00J	C13	C1	-164.6(2)	C46	C47	C49	O5	50.8(3)
C16	C00J	C13	C14	-36.0(2)	C46	C47	C49	O6	-129.4(2)
C16	C00J	C27	O1	54.0(3)	C46	C52	C53	C54	-178.8(3)
C16	C00J	C27	O2	-125.8(2)	C46	C52	C57	C56	178.9(3)
C16	C17	C18	C19	177.3(2)	C46	C58	C59	C60	-107.1(7)
C16	C17	C22	C21	-176.9(2)	C46	C58	C62	C61	103.4(8)
C17	C16	C24	C25	-24.9(4)	C47	C43	C44	C45	22.4(3)
C17	C18	C19	C20	-0.4(4)	C47	C46	C52	C53	71.1(3)
C18	C17	C22	C21	-0.4(4)	C47	C46	C52	C57	-107.6(3)
C18	C19	C20	C21	-0.7(5)	C47	C46	C58	C59	152.9(6)
C19	C20	C21	C22	1.3(5)	C47	C46	C58	C62	111.4(6)
C20	C21	C22	C17	-0.7(4)	C48	C47	C49	O5	175.8(2)
C22	C17	C18	C19	1.0(4)	C48	C47	C49	O6	-4.5(3)
C23	C00J	C13	C1	73.7(3)	C49	O6	C50	C51	-81.7(4)
C23	C00J	C13	C14	-157.7(2)	C50	O6	C49	O5	-3.5(4)
C23	C00J	C16	C15	155.0(2)	C50	O6	C49	C47	176.8(3)
C23	C00J	C16	C17	-79.9(3)	C52	C46	C47	C43	162.5(2)
C23	C00J	C16	C24	47.0(3)	C52	C46	C47	C48	-78.2(3)
C23	C00J	C27	O1	178.6(2)	C52	C46	C47	C49	45.4(3)
C23	C00J	C27	O2	-1.2(3)	C52	C46	C58	C59	-82.5(6)
C24	C16	C17	C18	134.8(2)	C52	C46	C58	C62	-123.9(6)

Table S7 Torsion Angles for 3.

A	B	C	D	Angle/°	A	B	C	D	Angle/°
C24	C16	C17	C22	-48.8(3)	C52	C53	C54	C55	0.1(6)
C27	O2	C28	C29	-87.0(3)	C53	C52	C57	C56	0.2(4)
C27	C00J	C13	C1	-50.0(3)	C53	C54	C55	C56	-0.2(6)
C27	C00J	C13	C14	78.6(2)	C54	C55	C56	C57	0.3(5)
C27	C00J	C16	C15	-81.6(2)	C55	C56	C57	C52	-0.3(5)
C27	C00J	C16	C17	43.5(3)	C57	C52	C53	C54	-0.1(5)
C27	C00J	C16	C24	170.4(2)	C58	C46	C47	C43	-74.1(3)
C28	O2	C27	O1	-3.9(4)	C58	C46	C47	C48	45.2(3)
C28	O2	C27	C00J	175.9(3)	C58	C46	C47	C49	168.8(2)
O4	C45	C46	C47	160.4(3)	C58	C46	C52	C53	-51.1(3)
O4	C45	C46	C52	37.3(4)	C58	C46	C52	C57	130.3(3)
O4	C45	C46	C58	-86.6(3)					

Table S8 Hydrogen Atom Coordinates (Å×10^4) and Isotropic Displacement Parameters (Å^2×10^3) for 3.

Atom	*x*	*y*	*z*	U(eq)
H2	5566.46	6380.26	4767.21	38
H3	5585.37	4740.2	3546.74	37
H5	8269.13	6899.09	2168.61	39
H6	8285.58	8533.9	3416	37
H8	7295.63	3482.59	3277.13	42
H9	7418.69	1858.58	2094.04	46
H10	7079.12	1897.43	27.2	54
H11	6615.58	3579.18	-837.14	51
H12	6558.13	5235.94	342.88	42

Table S8 Hydrogen Atom Coordinates (Å×10^4) and Isotropic Displacement Parameters (Å^2×10^3) for 3.

Atom	*x*	*y*	*z*	U(eq)
H13	7354.43	9452.45	4681.99	33
H14A	5017.62	9283.35	4432.11	37
H14B	4821.51	8065.55	4979.61	37
H18	5942.63	9029.58	8526.86	38
H19	6710.46	9046.88	10641.88	48
H20	9133.83	9965.74	11869.54	52
H21	10809.62	10836.86	10971.03	51
H22	10062.95	10859.45	8875.73	40
H24	8125.85	11132.49	5943.21	35
H26A	9480(40)	13420(30)	8480(30)	16(7)
H26B	8070(100)	14010(80)	8000(90)	180(40)
H28A	9417.85	5898.93	8009.19	55
H28B	7728.39	5817.93	7695.3	55
H29A	8248.81	7117.37	9461.42	95
H29B	9952.63	7319.28	9752.61	95
H29C	8988.1	6063.89	9828.58	95
H32	13716.05	7262.95	2122.21	51
H33	13904.49	6004.33	577.51	64
H34	13426.01	6510.39	-1385.9	70
H35	12786.6	8286.54	-1798.46	67
H36	12644.87	9562.51	-251.61	55
H38	11596.17	8147.6	2595.06	39
H39	11565.15	9379.7	4224.5	35
H41	14406.12	12106.64	3557.16	43
H42	14429.88	10862.96	1926.29	44

Table S8 Hydrogen Atom Coordinates (Å×10^4) and Isotropic Displacement Parameters (Å^2×10^3) for 3.

**Atom**	***x***	***y***	***z***	**U(eq)**
H43	12650.03	11093.24	5706.6	33
H44A	14997.11	12065.77	6688.12	41
H44B	15135.87	12736.74	5574.5	41
H50A	10532.63	13375.91	1142.61	63
H50B	12230.41	13878.09	1900.71	63
H51A	11087.49	15443.9	1547.98	103
H51B	11764.54	15413.79	2966.12	103
H51C	10058.75	14896.72	2265.16	103
H53	9805.41	13747.25	5652.43	48
H54	8943.6	15434.59	5352.43	59
H55	10523.39	17278.95	5472.6	53
H56	13018.46	17428.05	5918.68	45
H57	13918.07	15750.93	6220.14	36
H58A	10905.33	12548.22	6876.88	42
H58	11072.03	12111.78	6731.21	42
H60A	13372.56	11410.58	8518.54	84
H60B	13698.18	12234.08	9804.24	84
H61A	12541.42	13788.47	9331.41	101
H61B	13430.79	12872.82	10016.65	101

Table S9 Atomic Occupancy for 3.

**Atom**	***Occupancy***	**Atom**	***Occupancy***	**Atom**	***Occupancy***
H58A	0.474(9)	H58	0.526(9)	C59	0.526(9)
C60	0.526(9)	H60A	0.526(9)	H60B	0.526(9)

Table S9 Atomic Occupancy for 3.

Atom	*Occupancy*	Atom	*Occupancy*	Atom	*Occupancy*
C61	0.474(9)	H61A	0.474(9)	H61B	0.474(9)
C62	0.474(9)				

X-ray crystallographic information of product 34

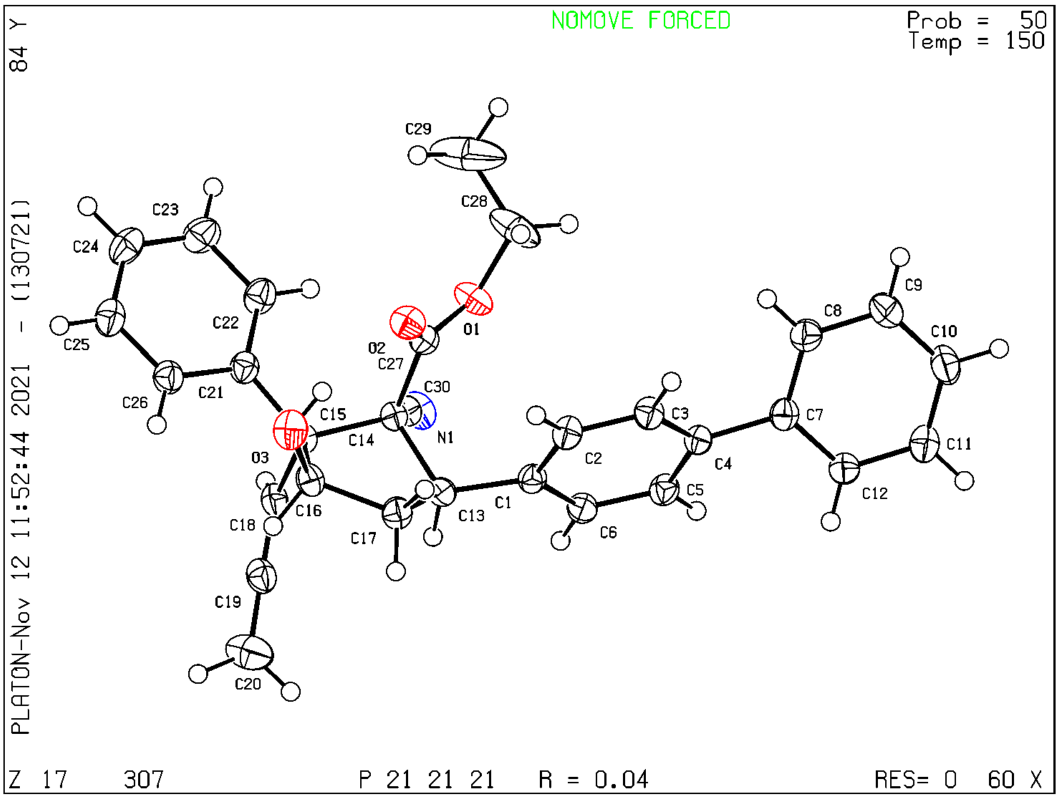

X-ray crystallography of **34**

Table S10 Crystal data and structure refinement for 34.

Identification code	**34**
Empirical formula	$C_{30}H_{27}NO_3$
Formula weight	449.52
Temperature/K	149.99(10)
Crystal system	orthorhombic

Space group	$P2_12_12_1$
a/Å	7.51317(15)
b/Å	15.2775(3)
c/Å	21.4630(4)
α/°	90
β/°	90
γ/°	90
Volume/Å^3	2463.57(8)
Z	4
ρ_{calc}g/cm^3	1.212
μ/mm^{-1}	0.617
F(000)	952.0
Crystal size/mm^3	0.15 × 0.11 × 0.09
Radiation	Cu Kα (λ = 1.54184)
2Θ range for data collection/°	7.102 to 148.196
Index ranges	$-9 \le h \le 9$, $-11 \le k \le 18$, $-26 \le l \le 23$
Reflections collected	12587
Independent reflections	4889 [R_{int} = 0.0256, R_{sigma} = 0.0284]
Data/restraints/parameters	4889/0/317
Goodness-of-fit on F^2	1.050
Final R indexes [I>=2σ (I)]	R_1 = 0.0351, wR_2 = 0.0884
Final R indexes [all data]	R_1 = 0.0367, wR_2 = 0.0903
Largest diff. peak/hole / e Å^{-3}	0.27/-0.18
Flack/Hooft parameter	0.09(10)/0.09(9)

Crystal structure determination of 34

Crystal Data for $C_{30}H_{27}NO_3$ (M = 449.52 g/mol): orthorhombic, space group $P2_12_12_1$ (no. 19), a = 7.51317 (15) Å, b = 15.2775 (3) Å, c = 21.4630 (4) Å, V = 2463.57 (8) Å^3, Z = 4, T =

149.99 (10) K, μ(Cu Kα) = 0.617 mm^{-1}, *Dcalc* = 1.212 g/cm^3, 12587 reflections measured (7.102° ≤ 2Θ ≤ 148.196°), 4889 unique (R_{int} = 0.0256, R_{sigma} = 0.0284) which were used in all calculations. The final R_1 was 0.0351 (I > 2σ(I)) and wR_2 was 0.0903 (all data).

Table S11 Fractional Atomic Coordinates (×10^4) and Equivalent Isotropic Displacement Parameters (Å^2×10^3) for 34. U_{eq} is defined as 1/3 of of the trace of the orthogonalised U_{IJ} tensor.

**Atom**	***x***	***y***	***z***	**U(eq)**
O1	6719(2)	6028.2(10)	7205.3(7)	33.6(3)
O2	9156.5(19)	5171.8(9)	7187.0(7)	30.3(3)
O3	10342(2)	3613.8(10)	6616.3(7)	34.4(3)
N1	3374(2)	5299.4(11)	6410.9(9)	33.6(4)
C1	7921(3)	6144.5(12)	5768.8(9)	23.9(4)
C2	9312(3)	6574.9(13)	6071.4(9)	26.7(4)
C3	9400(3)	7483.2(12)	6071.3(9)	26.6(4)
C4	8135(3)	7987.9(12)	5759.3(8)	24.0(4)
C5	6810(3)	7557.0(13)	5426.3(9)	26.9(4)
C6	6704(3)	6649.0(12)	5436.3(9)	26.6(4)
C7	8187(3)	8960.2(12)	5795.9(9)	25.2(4)
C8	8593(3)	9375.4(13)	6356.1(10)	32.6(5)
C9	8599(3)	10282.7(14)	6398.3(11)	39.0(5)
C10	8246(4)	10785.7(13)	5874.2(11)	37.7(5)
C11	7879(3)	10378.5(13)	5314.6(10)	32.1(5)
C12	7822(3)	9472.4(13)	5273.8(9)	27.1(4)
C13	7628(3)	5166.3(12)	5828.4(9)	23.3(4)
C14	6748(3)	4886.7(11)	6468.7(8)	22.1(4)
C15	7048(3)	3866.2(11)	6474.9(9)	23.1(4)
C16	8949(3)	3751.9(12)	6181.7(9)	26.0(4)
C17	9267(3)	4573.0(12)	5779.1(9)	27.4(4)

Table S11 Fractional Atomic Coordinates (×10^4) and Equivalent Isotropic Displacement Parameters (Å^2×10^3) for 34. U_{eq} is defined as 1/3 of of the trace of the orthogonalised U_{IJ} tensor.

Atom	*x*	*y*	*z*	U(eq)
C18	5656(3)	3459.7(12)	6041.0(9)	26.0(4)
C19	5973(3)	3114.3(13)	5499.9(10)	30.4(4)
C20	6301(4)	2784(2)	4954.7(13)	48.9(7)
C21	6810(3)	3427.0(12)	7108.3(9)	26.0(4)
C22	5901(3)	3809.7(14)	7599.8(10)	32.3(5)
C23	5647(3)	3355.4(15)	8159.2(10)	37.4(5)
C24	6271(3)	2510.5(15)	8222.5(10)	34.9(5)
C25	7166(3)	2118.4(14)	7733.5(10)	32.7(5)
C26	7442(3)	2573.0(13)	7184.5(10)	29.5(4)
C27	7690(3)	5361.2(13)	7004.7(9)	24.8(4)
C28	7488(4)	6612.2(19)	7672.5(13)	54.7(8)
C29	6944(5)	6327(3)	8305.8(14)	87.5(14)
C30	4851(3)	5125.0(12)	6454.5(9)	24.6(4)

Table S12 Anisotropic Displacement Parameters (Å^2×10^3) for 34. The Anisotropic displacement factor exponent takes the form: $-2\pi^2[h^2a^{*2}U_{11}+2hka^*b^*U_{12}+\ldots]$.

Atom	U_{11}	U_{22}	U_{33}	U_{23}	U_{13}	U_{12}
O1	31.1(8)	33.5(7)	36.1(8)	-14.0(6)	-6.7(6)	6.0(6)
O2	24.0(7)	35.0(7)	31.8(7)	-1.3(6)	-6.1(6)	1.1(6)
O3	25.4(8)	33.7(7)	44.0(8)	4.3(7)	-3.4(6)	4.4(6)
N1	26.2(10)	31.1(8)	43.6(10)	-5.9(8)	-3.6(8)	1.0(7)
C1	23.5(10)	24.2(9)	24.0(9)	1.0(7)	1.0(8)	-2.0(7)
C2	22.1(9)	27.4(9)	30.7(9)	4.6(8)	-4.0(8)	-1.2(8)

Table S12 Anisotropic Displacement Parameters ($Å^2 \times 10^3$) for 34. The Anisotropic displacement factor exponent takes the form: $-2\pi^2[h^2a^{*2}U_{11}+2hka^*b^*U_{12}+…]$.

Atom	U_{11}	U_{22}	U_{33}	U_{23}	U_{13}	U_{12}
C3	22.8(10)	26.5(9)	30.6(9)	1.6(8)	-5.2(8)	-4.3(8)
C4	25.1(10)	22.5(8)	24.3(8)	1.1(7)	1.2(8)	-1.9(8)
C5	27.0(10)	26.3(9)	27.3(9)	3.1(8)	-4.3(8)	0.7(8)
C6	27.3(10)	26.6(9)	25.8(9)	0.2(7)	-5.9(8)	-4.3(8)
C7	21.6(10)	24.8(9)	29.4(9)	1.6(7)	1.5(8)	-2.1(7)
C8	38.6(12)	30.2(10)	29.1(10)	1.8(8)	-5.0(9)	-4.5(9)
C9	49.7(15)	30.3(10)	37.0(11)	-5.5(9)	-6.0(11)	-6.4(10)
C10	44.4(14)	22.5(9)	46.3(12)	-1.1(9)	-4.8(11)	-3.2(9)
C11	30.8(11)	26.8(9)	38.6(11)	5.3(8)	-3.7(9)	-0.9(8)
C12	26.0(10)	27.1(9)	28.1(9)	0.6(7)	-1.8(8)	-0.1(8)
C13	23.1(9)	22.7(9)	24.2(8)	-0.9(7)	-0.5(7)	-0.9(7)
C14	21.6(9)	20.4(8)	24.2(8)	-0.3(7)	-0.7(7)	0.2(7)
C15	22.4(9)	20.3(8)	26.5(9)	-0.4(7)	-0.3(8)	0.1(7)
C16	23.7(10)	21.9(8)	32.5(9)	-1.3(7)	0.9(8)	1.4(7)
C17	25.7(10)	26.1(9)	30.6(9)	0.6(8)	5.1(8)	0.0(8)
C18	26.5(10)	20.2(8)	31.1(9)	0.8(7)	-2.3(8)	-2.7(7)
C19	31.6(11)	23.9(9)	35.5(10)	-0.4(8)	-7.0(9)	1.5(8)
C20	49.6(16)	56.8(16)	40.2(13)	-13.5(12)	-8.1(12)	15.8(13)
C21	24.6(10)	24.9(9)	28.6(9)	2.3(7)	-2.6(8)	-2.9(8)
C22	30.1(11)	31.3(10)	35.5(11)	5.4(8)	4.8(9)	3.1(9)
C23	34.2(12)	43.5(12)	34.5(11)	3.8(9)	8.6(10)	-0.4(10)
C24	30.2(11)	40.6(11)	33.8(10)	11.4(9)	-0.7(9)	-6.9(9)
C25	31.9(12)	28.3(9)	38.0(11)	6.7(8)	-5.7(9)	-1.9(9)
C26	31.4(11)	25.6(9)	31.6(10)	1.4(8)	-2.7(9)	0.9(8)

Table S12 Anisotropic Displacement Parameters (Å^2×10^3) for 34. The Anisotropic displacement factor exponent takes the form: $-2\pi^2[h^2a^{*2}U_{11}+2hka^*b^*U_{12}+\ldots]$.

Atom	U_{11}	U_{22}	U_{33}	U_{23}	U_{13}	U_{12}
C27	24.0(10)	25.2(9)	25.1(9)	0.1(7)	1.0(8)	-0.8(7)
C28	51.5(16)	54.4(15)	58.2(16)	-34.3(13)	-21.1(13)	11.3(13)
C29	71(2)	147(4)	44.4(16)	-40(2)	-19.6(16)	41(2)
C30	25.0(11)	20.9(8)	27.7(9)	-1.5(7)	0.5(7)	-0.8(7)

Table S13 Bond Lengths for 34.

Atom	Atom	Length/Å	Atom	Atom	Length/Å
O1	C27	1.325(2)	C13	C14	1.584(3)
O1	C28	1.461(3)	C13	C17	1.533(3)
O2	C27	1.204(3)	C14	C15	1.575(2)
O3	C16	1.418(2)	C14	C27	1.533(3)
N1	C30	1.145(3)	C14	C30	1.471(3)
C1	C2	1.395(3)	C15	C16	1.570(3)
C1	C6	1.393(3)	C15	C18	1.531(3)
C1	C13	1.516(2)	C15	C21	1.527(3)
C2	C3	1.389(3)	C16	C17	1.542(3)
C3	C4	1.395(3)	C18	C19	1.298(3)
C4	C5	1.392(3)	C19	C20	1.298(3)
C4	C7	1.488(2)	C21	C22	1.386(3)
C5	C6	1.390(3)	C21	C26	1.398(3)
C7	C8	1.393(3)	C22	C23	1.400(3)
C7	C12	1.394(3)	C23	C24	1.380(3)
C8	C9	1.389(3)	C24	C25	1.383(3)

Table S13 Bond Lengths for 34.

Atom	Atom	Length/Å	Atom	Atom	Length/Å
C9	C10	1.388(3)	C25	C26	1.383(3)
C10	C11	1.380(3)	C28	C29	1.485(5)
C11	C12	1.388(3)			

Table S14 Bond Angles for 34.

Atom	Atom	Atom	Angle/°	Atom	Atom	Atom	Angle/°
C27	O1	C28	118.34(18)	C30	C14	C27	110.24(16)
C2	C1	C13	122.28(17)	C16	C15	C14	103.70(15)
C6	C1	C2	118.00(17)	C18	C15	C14	107.37(15)
C6	C1	C13	119.57(17)	C18	C15	C16	109.39(15)
C3	C2	C1	120.42(18)	C21	C15	C14	115.20(15)
C2	C3	C4	121.32(18)	C21	C15	C16	114.48(16)
C3	C4	C7	120.58(18)	C21	C15	C18	106.46(15)
C5	C4	C3	118.20(17)	O3	C16	C15	115.11(16)
C5	C4	C7	121.20(18)	O3	C16	C17	112.03(16)
C6	C5	C4	120.35(19)	C17	C16	C15	105.98(15)
C5	C6	C1	121.53(18)	C13	C17	C16	108.52(16)
C8	C7	C4	120.38(17)	C19	C18	C15	125.7(2)
C8	C7	C12	118.76(18)	C18	C19	C20	178.8(2)
C12	C7	C4	120.85(18)	C22	C21	C15	123.37(17)
C9	C8	C7	120.75(19)	C22	C21	C26	118.17(18)
C10	C9	C8	119.9(2)	C26	C21	C15	118.33(17)
C11	C10	C9	119.58(19)	C21	C22	C23	120.7(2)
C10	C11	C12	120.71(19)	C24	C23	C22	120.1(2)

Table S14 Bond Angles for 34.

Atom	Atom	Atom	Angle/°	Atom	Atom	Atom	Angle/°
C11	C12	C7	120.21(19)	C23	C24	C25	119.72(19)
C1	C13	C14	113.57(15)	C24	C25	C26	120.1(2)
C1	C13	C17	117.40(17)	C25	C26	C21	121.1(2)
C17	C13	C14	103.65(15)	O1	C27	C14	110.67(17)
C15	C14	C13	102.39(14)	O2	C27	O1	125.65(18)
C27	C14	C13	109.32(15)	O2	C27	C14	123.56(18)
C27	C14	C15	113.30(15)	O1	C28	C29	109.9(3)
C30	C14	C13	108.65(15)	N1	C30	C14	176.4(2)
C30	C14	C15	112.55(15)				

Table S15 Torsion Angles for 34.

A	B	C	D	Angle/°	A	B	C	D	Angle/°
O3	C16	C17	C13	-126.00(17)	C14	C15	C18	C19	108.6(2)
C1	C2	C3	C4	-1.5(3)	C14	C15	C21	C22	18.7(3)
C1	C13	C14	C15	-166.29(16)	C14	C15	C21	C26	-165.49(18)
C1	C13	C14	C27	-45.9(2)	C15	C14	C27	O1	-144.25(17)
C1	C13	C14	C30	74.5(2)	C15	C14	C27	O2	39.6(3)
C1	C13	C17	C16	149.55(17)	C15	C16	C17	C13	0.3(2)
C2	C1	C6	C5	-2.9(3)	C15	C21	C22	C23	176.6(2)
C2	C1	C13	C14	76.7(2)	C15	C21	C26	C25	-175.76(19)
C2	C1	C13	C17	-44.3(3)	C16	C15	C18	C19	-3.3(3)
C2	C3	C4	C5	-2.3(3)	C16	C15	C21	C22	138.8(2)
C2	C3	C4	C7	176.28(19)	C16	C15	C21	C26	-45.4(2)
C3	C4	C5	C6	3.5(3)	C17	C13	C14	C15	-37.79(18)

Table S15 Torsion Angles for 34.

A	B	C	D	Angle/°	A	B	C	D	Angle/°
C3	C4	C7	C8	-40.0(3)	C17	C13	C14	C27	82.61(18)
C3	C4	C7	C12	140.4(2)	C17	C13	C14	C30	-157.04(15)
C4	C5	C6	C1	-0.9(3)	C18	C15	C16	O3	-145.35(15)
C4	C7	C8	C9	-178.3(2)	C18	C15	C16	C17	90.24(18)
C4	C7	C12	C11	-179.9(2)	C18	C15	C21	C22	-100.2(2)
C5	C4	C7	C8	138.6(2)	C18	C15	C21	C26	75.6(2)
C5	C4	C7	C12	-41.0(3)	C21	C15	C16	O3	-26.0(2)
C6	C1	C2	C3	4.1(3)	C21	C15	C16	C17	-150.40(16)
C6	C1	C13	C14	-98.8(2)	C21	C15	C18	C19	-127.5(2)
C6	C1	C13	C17	140.09(19)	C21	C22	C23	C24	-1.4(4)
C7	C4	C5	C6	-175.10(19)	C22	C21	C26	C25	0.3(3)
C7	C8	C9	C10	-1.8(4)	C22	C23	C24	C25	0.7(3)
C8	C7	C12	C11	0.5(3)	C23	C24	C25	C26	0.3(3)
C8	C9	C10	C11	0.4(4)	C24	C25	C26	C21	-0.9(3)
C9	C10	C11	C12	1.4(4)	C26	C21	C22	C23	0.8(3)
C10	C11	C12	C7	-1.9(3)	C27	O1	C28	C29	-94.3(3)
C12	C7	C8	C9	1.3(3)	C27	C14	C15	C16	-79.64(18)
C13	C1	C2	C3	-171.56(19)	C27	C14	C15	C18	164.62(15)
C13	C1	C6	C5	172.85(18)	C27	C14	C15	C21	46.2(2)
C13	C14	C15	C16	37.96(17)	C28	O1	C27	O2	2.3(3)
C13	C14	C15	C18	-77.78(17)	C28	O1	C27	C14	-173.8(2)
C13	C14	C15	C21	163.84(16)	C30	C14	C15	C16	154.44(16)
C13	C14	C27	O1	102.27(18)	C30	C14	C15	C18	38.7(2)
C13	C14	C27	O2	-73.9(2)	C30	C14	C15	C21	-79.7(2)

Table S15 Torsion Angles for 34.

A	B	C	D	Angle/°	A	B	C	D	Angle/°
C14	C13	C17	C16	23.4(2)	C30	C14	C27	O1	-17.1(2)
C14	C15	C16	O3	100.35(18)	C30	C14	C27	O2	166.71(18)
C14	C15	C16	C17	-24.06(18)					

Table S16 Hydrogen Atom Coordinates (Å×10^4) and Isotropic Displacement Parameters (Å^2×10^3) for 34.

Atom	*x*	*y*	*z*	U(eq)
H3	10420.72	4039.49	6847.3	52
H2	10185.85	6251.64	6274.44	32
H3A	10320.61	7760.32	6283.44	32
H5	5990.26	7878.34	5195.73	32
H6	5799.53	6371.81	5215.86	32
H8	8862.59	9041.34	6705.81	39
H9	8838.88	10552.81	6777.35	47
H10	8256.8	11393.36	5899.85	45
H11	7667.38	10714.94	4961.19	39
H12	7539.44	9206.05	4896.96	33
H13	6806.1	4993.08	5495.9	28
H16	8910.08	3244.37	5902.77	31
H17A	10315.1	4882.37	5924.76	33
H17B	9459.53	4405.06	5348.51	33
H18	4481.36	3460.32	6177.3	31
H20A	6650(40)	2170(20)	4892(14)	53(8)
H20B	6290(40)	3119(19)	4584(14)	52(8)

Table S16 Hydrogen Atom Coordinates (Å×10[4]) and Isotropic Displacement Parameters (Å[2]×10[3]) for 34.

**Atom**	***x***	***y***	***z***	**U(eq)**
H22	5454.84	4374.45	7557.45	39
H23	5057.21	3623.51	8488.86	45
H24	6090.79	2206.08	8592.36	42
H25	7582.72	1547.96	7773.8	39
H26	8059.81	2305.97	6860.61	35
H28A	7084.7	7206.51	7599.33	66
H28B	8775.72	6604.01	7639.82	66
H29A	5671.13	6279.38	8323.8	131
H29B	7341.2	6748.89	8606.71	131
H29C	7468.49	5768.23	8396.33	131

X-ray crystallographic information of product 35

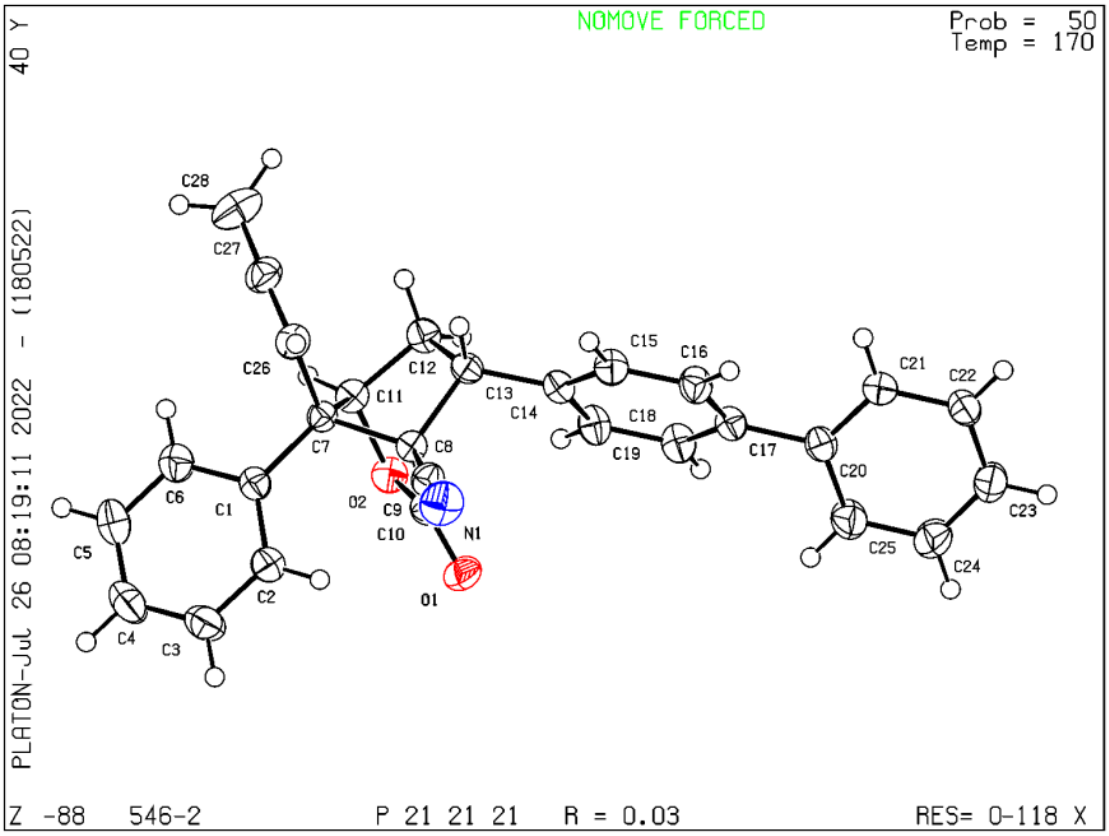

X-ray crystallography of **35**

Table S17 Crystal data and structure refinement for 35.

Identification code	**35**
Empirical formula	$C_{28}H_{21}NO_2$
Formula weight	403.46
Temperature/K	170.00(10)
Crystal system	orthorhombic
Space group	$P2_12_12_1$
a/Å	6.22984(13)
b/Å	15.4298(4)
c/Å	22.0803(5)
α/°	90
β/°	90
γ/°	90
Volume/Å^3	2122.48(8)
Z	4
ρ_{calc}g/cm^3	1.263
μ/mm^{-1}	0.624
F(000)	848.0
Crystal size/mm^3	0.14 × 0.12 × 0.1
Radiation	Cu Kα (λ = 1.54184)
2Θ range for data collection/°	6.99 to 146.926
Index ranges	$-5 \le h \le 7$, $-18 \le k \le 19$, $-26 \le l \le 26$
Reflections collected	11751
Independent reflections	4186 [R_{int} = 0.0305, R_{sigma} = 0.0314]
Data/restraints/parameters	4186/0/289

Goodness-of-fit on F^2	1.040
Final R indexes [I>=2σ (I)]	R_1 = 0.0337, wR_2 = 0.0863
Final R indexes [all data]	R_1 = 0.0357, wR_2 = 0.0879
Largest diff. peak/hole / e Å^{-3}	0.20/-0.15
Flack/Hooft parameter	0.07(12)/0.10(11)

Crystal structure determination of [35]

Crystal Data for $C_{28}H_{21}NO_2$ (*M* =403.46 g/mol): orthorhombic, space group $P2_12_12_1$ (no. 19), *a* = 6.22984(13) Å, *b* = 15.4298(4) Å, *c* = 22.0803(5) Å, *V* = 2122.48(8) Å^3, *Z* = 4, *T* = 170.00(10) K, μ(Cu Kα) = 0.624 mm^{-1}, *Dcalc* = 1.263 g/cm^3, 11751 reflections measured (6.99 ° ≤ 2Θ ≤ 146.926°), 4186 unique (R_{int} = 0.0305, R_{sigma} = 0.0314) which were used in all calculations. The final R_1 was 0.0337 (I > 2σ(I)) and wR_2 was 0.0879 (all data).

Table S18 Fractional Atomic Coordinates (×10^4) and Equivalent Isotropic Displacement Parameters (Å^2 ×10^3) for 35. U_{eq} is defined as 1/3 of the trace of the orthogonalised U_{IJ} tensor.

Atom	*x*	*y*	*z*	U(eq)
O1	1093(2)	2996.6(9)	2586.5(6)	37.9(3)
O2	1875(2)	1581.3(9)	2479.9(6)	31.2(3)
N1	6085(3)	4030.1(11)	2021.9(9)	45.5(5)
C1	3544(3)	1834.3(12)	1285.0(8)	27.5(4)
C2	2406(3)	2582.7(14)	1139.3(9)	33.8(4)
C3	1150(4)	2621.1(16)	622.2(9)	42.9(5)
C4	1007(4)	1919.7(17)	242.2(10)	47.0(6)
C5	2145(4)	1177.4(17)	374.3(10)	48.8(6)
C6	3411(4)	1133.8(14)	890.4(9)	39.7(5)
C7	4901(3)	1731.8(11)	1857.1(8)	24.3(4)
C8	4751(3)	2493.3(11)	2320.6(8)	24.1(4)
C9	5477(3)	3358.1(12)	2146.6(8)	29.4(4)
C10	2362(3)	2435.9(12)	2474.0(8)	27.7(4)
C11	3849(3)	1113.4(12)	2321.9(8)	27.7(4)

Table S18 Fractional Atomic Coordinates (×10^4) and Equivalent Isotropic Displacement Parameters (Å^2×10^3) for 35. U_{eq} is defined as 1/3 of the trace of the orthogonalised U_{IJ} tensor.

Atom	*x*	*y*	*z*	U(eq)
C12	5274(3)	1157.5(12)	2880.5(8)	30.0(4)
C13	5975(3)	2121.1(11)	2892.5(8)	26.2(4)
C14	5560(3)	2618.5(12)	3469.4(8)	27.2(4)
C15	7071(3)	3210.5(13)	3677.8(9)	31.4(4)
C16	6723(3)	3687.7(13)	4201.7(9)	32.8(4)
C17	4836(3)	3595.0(12)	4535.0(8)	29.1(4)
C18	3321(3)	3001.7(13)	4325.9(9)	33.4(4)
C19	3673(3)	2521.0(13)	3804.9(8)	32.1(4)
C20	4441(3)	4118.1(12)	5088.6(8)	30.0(4)
C21	6069(3)	4266.9(13)	5505.3(9)	34.0(4)
C22	5699(4)	4755.6(14)	6023.6(9)	37.7(5)
C23	3694(4)	5093.8(13)	6133.7(9)	38.5(5)
C24	2057(4)	4959.0(17)	5725.1(10)	45.9(6)
C25	2431(3)	4471.9(16)	5207.9(10)	43.0(5)
C26	7176(3)	1489.5(13)	1685.1(9)	30.5(4)
C27	7915(3)	706.2(14)	1714.6(10)	37.0(5)
C28	8581(5)	-86.4(17)	1766.2(16)	57.2(7)

Table S19 Anisotropic Displacement Parameters (Å^2×10^3) for 35. The Anisotropic displacement factor exponent takes the form: $-2\pi^2[h^2a^{*2}U_{11}+2hka^*b^*U_{12}+\ldots]$.

Atom	U_{11}	U_{22}	U_{33}	U_{23}	U_{13}	U_{12}
O1	29.5(7)	42.3(8)	41.8(8)	-5.4(6)	4.6(6)	7.3(6)
O2	24.0(6)	34.2(7)	35.2(7)	-0.1(6)	2.7(5)	-5.8(5)

Table S19 Anisotropic Displacement Parameters (Å^2 ×10^3) for 35. The Anisotropic displacement factor exponent takes the form: -2π^2[h^{2}a*^{2}U$_{11}$+2hka*b*U$_{12}$+…].

Atom	U_{11}	U_{22}	U_{33}	U_{23}	U_{13}	U_{12}
N1	55.4(12)	29.8(9)	51.4(11)	5.4(8)	3.1(10)	-6.9(8)
C1	25.8(8)	31.5(9)	25.3(8)	0.7(7)	1.2(7)	-2.3(7)
C2	35.5(10)	35.6(10)	30.3(9)	0.9(8)	-0.8(8)	2.0(9)
C3	40.2(11)	50.9(13)	37.7(11)	6.8(10)	-7.4(9)	5.7(11)
C4	42.5(12)	65.5(15)	33.0(11)	0.4(10)	-10.8(9)	-3.5(11)
C5	55.4(14)	52.9(14)	38.0(12)	-12.5(11)	-9.9(10)	-4.7(12)
C6	44.4(12)	36.9(11)	37.8(11)	-5.2(9)	-5.2(9)	0.9(10)
C7	24.6(8)	22.9(8)	25.5(8)	-0.8(7)	0.0(7)	-1.1(7)
C8	24.3(8)	22.9(8)	25.2(8)	1.0(7)	1.6(7)	-2.0(7)
C9	32.1(9)	26.0(9)	30.2(9)	0.0(7)	1.6(7)	-0.2(8)
C10	25.1(9)	32.7(9)	25.5(8)	-0.5(7)	0.1(7)	-1.4(8)
C11	27.9(9)	24.2(8)	30.9(9)	0.6(7)	1.8(7)	-3.7(7)
C12	33.5(10)	27.2(9)	29.4(9)	2.9(7)	-0.4(8)	-2.7(8)
C13	23.8(8)	27.8(9)	26.9(8)	1.7(7)	-1.4(7)	-1.9(7)
C14	27.8(9)	28.3(9)	25.4(8)	1.0(7)	-2.0(7)	-2.8(8)
C15	24.4(8)	37.3(10)	32.5(9)	-2.6(8)	2.1(7)	-5.1(8)
C16	29.9(9)	34.7(10)	33.7(10)	-5.7(8)	-2.4(8)	-6.1(8)
C17	28.8(9)	32.1(10)	26.6(9)	-0.8(7)	-1.7(7)	-2.4(8)
C18	29.4(10)	40.7(11)	30.1(9)	0.0(8)	4.1(8)	-8.0(9)
C19	31.7(9)	35.4(10)	29.2(9)	-1.8(8)	0.7(8)	-11.0(8)
C20	32.4(10)	31.7(10)	25.8(9)	1.7(7)	0.4(7)	-2.0(8)
C21	34.9(10)	34.0(10)	33.0(10)	-0.7(8)	-3.6(8)	5.1(9)
C22	47.3(12)	35.4(10)	30.6(10)	-2.5(8)	-10.0(9)	4.6(9)
C23	51.7(12)	32.9(10)	30.7(10)	-2.3(8)	2.9(9)	2.5(10)

Table S19 Anisotropic Displacement Parameters (Å^2×10^3) for 35. The Anisotropic displacement factor exponent takes the form: $-2\pi^2[h^2a^{*2}U_{11}+2hka^*b^*U_{12}+\ldots]$.

Atom	U_{11}	U_{22}	U_{33}	U_{23}	U_{13}	U_{12}
C24	35.2(11)	57.9(14)	44.7(12)	-10.8(11)	3.2(9)	7.8(11)
C25	30.3(11)	60.1(15)	38.7(11)	-10.3(10)	-1.9(9)	0.4(10)
C26	26.2(9)	31.5(10)	33.9(9)	-2.1(8)	2.9(7)	-3.1(8)
C27	29.8(10)	39.0(11)	42.2(11)	-7.7(9)	3.1(8)	3.0(9)
C28	54.1(15)	38.9(13)	78.5(19)	-4.7(13)	10.3(14)	11.9(12)

Table S20 Bond Lengths for 35.

Atom	Atom	Length/Å	Atom	Atom	Length/Å
O1	C10	1.198(2)	C12	C13	1.550(2)
O2	C10	1.353(2)	C13	C14	1.509(2)
O2	C11	1.468(2)	C14	C15	1.390(3)
N1	C9	1.138(3)	C14	C19	1.398(3)
C1	C2	1.393(3)	C15	C16	1.388(3)
C1	C6	1.391(3)	C16	C17	1.394(3)
C1	C7	1.528(2)	C17	C18	1.393(3)
C2	C3	1.385(3)	C17	C20	1.485(3)
C3	C4	1.372(3)	C18	C19	1.386(3)
C4	C5	1.378(4)	C20	C21	1.389(3)
C5	C6	1.388(3)	C20	C25	1.391(3)
C7	C8	1.561(2)	C21	C22	1.390(3)
C7	C11	1.547(2)	C22	C23	1.375(3)
C7	C26	1.514(2)	C23	C24	1.377(3)
C8	C9	1.460(2)	C24	C25	1.387(3)

Table S20 Bond Lengths for 35.

Atom	Atom	Length/Å	Atom	Atom	Length/Å
C8	C10	1.529(2)	C26	C27	1.295(3)
C8	C13	1.583(2)	C27	C28	1.296(3)
C11	C12	1.521(3)			

Table S21 Bond Angles for 35.

Atom	Atom	Atom	Angle/°	Atom	Atom	Atom	Angle/°
C10	O2	C11	106.79(13)	O2	C11	C12	105.91(14)
C2	C1	C7	123.95(16)	C12	C11	C7	105.26(14)
C6	C1	C2	117.99(18)	C11	C12	C13	102.77(14)
C6	C1	C7	118.06(17)	C12	C13	C8	101.46(13)
C3	C2	C1	120.89(19)	C14	C13	C8	113.97(14)
C4	C3	C2	120.5(2)	C14	C13	C12	117.00(15)
C3	C4	C5	119.5(2)	C15	C14	C13	119.84(16)
C4	C5	C6	120.4(2)	C15	C14	C19	117.72(16)
C5	C6	C1	120.7(2)	C19	C14	C13	122.44(16)
C1	C7	C8	115.52(14)	C16	C15	C14	121.22(17)
C1	C7	C11	112.19(14)	C15	C16	C17	121.15(17)
C11	C7	C8	90.23(13)	C16	C17	C20	121.22(16)
C26	C7	C1	109.63(14)	C18	C17	C16	117.62(17)
C26	C7	C8	113.98(14)	C18	C17	C20	121.16(17)
C26	C7	C11	114.25(15)	C19	C18	C17	121.28(17)
C7	C8	C13	102.77(13)	C18	C19	C14	121.01(17)
C9	C8	C7	119.78(14)	C21	C20	C17	120.95(17)
C9	C8	C10	114.36(15)	C21	C20	C25	117.84(18)

Table S21 Bond Angles for 35.

Atom	Atom	Atom	Angle/°	Atom	Atom	Atom	Angle/°
C9	C8	C13	113.10(15)	C25	C20	C17	121.21(17)
C10	C8	C7	99.21(14)	C20	C21	C22	120.94(19)
C10	C8	C13	105.73(14)	C23	C22	C21	120.1(2)
N1	C9	C8	178.2(2)	C22	C23	C24	119.96(19)
O1	C10	O2	123.62(17)	C23	C24	C25	119.8(2)
O1	C10	C8	130.28(18)	C24	C25	C20	121.3(2)
O2	C10	C8	106.07(15)	C27	C26	C7	123.40(19)
O2	C11	C7	102.09(13)	C26	C27	C28	177.0(3)

Table S22 Torsion Angles for 35.

A	B	C	D	Angle/°	A	B	C	D	Angle/°
O2	C11	C12	C13	-71.33(16)	C11	O2	C10	O1	-179.75(17)
C1	C2	C3	C4	0.0(3)	C11	O2	C10	C8	-1.37(18)
C1	C7	C8	C9	-63.1(2)	C11	C7	C8	C9	-177.92(16)
C1	C7	C8	C10	61.99(17)	C11	C7	C8	C10	-52.88(14)
C1	C7	C8	C13	170.55(14)	C11	C7	C8	C13	55.69(14)
C1	C7	C11	O2	-63.99(17)	C11	C7	C26	C27	25.9(3)
C1	C7	C11	C12	-174.42(15)	C11	C12	C13	C8	1.02(17)
C1	C7	C26	C27	-101.0(2)	C11	C12	C13	C14	125.68(16)
C2	C1	C6	C5	-1.3(3)	C12	C13	C14	C15	141.04(18)
C2	C1	C7	C8	6.5(2)	C12	C13	C14	C19	-40.2(2)
C2	C1	C7	C11	108.0(2)	C13	C8	C10	O1	109.0(2)
C2	C1	C7	C26	-123.90(19)	C13	C8	C10	O2	-69.22(17)
C2	C3	C4	C5	-0.9(4)	C13	C14	C15	C16	179.00(17)

Table S22 Torsion Angles for 35.

A	B	C	D	Angle/°	A	B	C	D	Angle/°
C3	C4	C5	C6	0.7(4)	C13	C14	C19	C18	-178.58(18)
C4	C5	C6	C1	0.4(4)	C14	C15	C16	C17	-0.5(3)
C6	C1	C2	C3	1.1(3)	C15	C14	C19	C18	0.3(3)
C6	C1	C7	C8	-172.46(17)	C15	C16	C17	C18	0.5(3)
C6	C1	C7	C11	-71.0(2)	C15	C16	C17	C20	-178.74(18)
C6	C1	C7	C26	57.1(2)	C16	C17	C18	C19	-0.1(3)
C7	C1	C2	C3	-177.89(18)	C16	C17	C20	C21	-41.5(3)
C7	C1	C6	C5	177.77(19)	C16	C17	C20	C25	138.8(2)
C7	C8	C10	O1	-144.8(2)	C17	C18	C19	C14	-0.3(3)
C7	C8	C10	O2	36.94(16)	C17	C20	C21	C22	-179.93(18)
C7	C8	C13	C12	-37.30(16)	C17	C20	C25	C24	179.9(2)
C7	C8	C13	C14	-163.96(14)	C18	C17	C20	C21	139.4(2)
C7	C11	C12	C13	36.34(18)	C18	C17	C20	C25	-40.4(3)
C8	C7	C11	O2	53.85(14)	C19	C14	C15	C16	0.1(3)
C8	C7	C11	C12	-56.58(15)	C20	C17	C18	C19	179.13(18)
C8	C7	C26	C27	127.8(2)	C20	C21	C22	C23	0.6(3)
C8	C13	C14	C15	-100.9(2)	C21	C20	C25	C24	0.1(3)
C8	C13	C14	C19	77.9(2)	C21	C22	C23	C24	-1.0(3)
C9	C8	C10	O1	-16.1(3)	C22	C23	C24	C25	0.9(4)
C9	C8	C10	O2	165.68(15)	C23	C24	C25	C20	-0.5(4)
C9	C8	C13	C12	-167.87(15)	C25	C20	C21	C22	-0.1(3)
C9	C8	C13	C14	65.46(19)	C26	C7	C8	C9	65.3(2)
C10	O2	C11	C7	-35.42(17)	C26	C7	C8	C10	-169.70(15)
C10	O2	C11	C12	74.51(17)	C26	C7	C8	C13	-61.14(18)

Table S22 Torsion Angles for 35.

A	B	C	D	Angle/°	A	B	C	D	Angle/°
C10	C8	C13	C12	66.25(17)	C26	C7	C11	O2	170.43(14)
C10	C8	C13	C14	-60.41(19)	C26	C7	C11	C12	60.00(19)

Table S23 Hydrogen Atom Coordinates (Å×10^4) and Isotropic Displacement Parameters (Å^2×10^3) for 35.

Atom	*x*	*y*	*z*	U(eq)
H2	2489.34	3063.02	1392.61	41
H3	400.04	3125.88	531.86	51
H4	147.43	1944.99	-101.75	56
H5	2063.48	702.64	115.87	59
H6	4179.38	630.78	973.31	48
H11	3608.31	524.81	2169.66	33
H12A	6503.03	775.6	2841.71	36
H12B	4481.15	1005.18	3243.36	36
H13	7518.61	2144.46	2808.69	31
H15	8339.24	3288.27	3462.21	38
H16	7766.1	4076.01	4332.54	39
H18	2049.05	2926.78	4540.12	40
H19	2637.32	2127.65	3676.51	39
H21	7426.76	4036.36	5436.58	41
H22	6809.1	4854.11	6296.63	45
H23	3443.24	5413.2	6483.82	46
H24	704.82	5193.87	5795.71	55
H25	1316.49	4380	4935.35	52

Table S23 Hydrogen Atom Coordinates (Å×10^4) and Isotropic Displacement Parameters (Å^2×10^3) for 35.

Atom	*x*	*y*	*z*	U(eq)
H26	8090.14	1925.82	1551.33	37
H28A	8350(60)	-500(20)	1443(16)	93(12)
H28B	9320(60)	-350(20)	2135(17)	95(12)

Computational details

All calculations were performed using Gaussian 16, Revision A.03 package.[6] All of the structures were optimized by DFT with the B3LYP-D3(BJ) functional.[7] We employed LANL2DZ basis set for Ni with effective core potentials, 6-31G(d) basis sets for H, C, and O. All the stationary structures were characterized with no imaginary frequency. The orbital contribution analysis is carried out Multiwfn 3.8.[8]

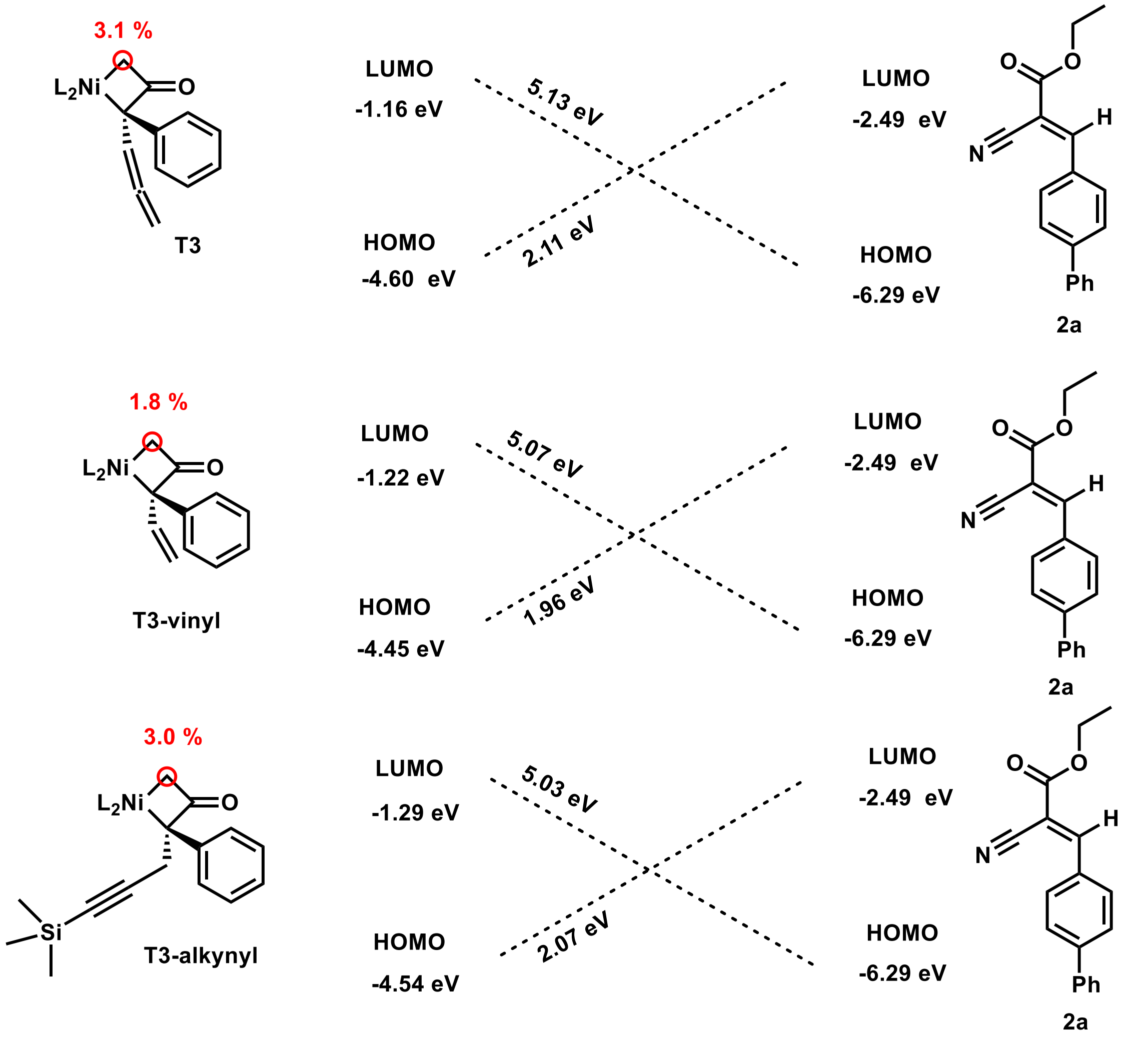

Cartesian coordinates of the optimized structures

2a

E = -900.201898 a.u.

0 1

C	-0.17326000	0.23647900	-0.68038900
H	0.51005700	-0.59704400	-0.54685200
C	0.38426500	1.45586900	-0.44047100
C	-0.31327000	2.69761700	-0.55124400
N	-0.89525900	3.70060800	-0.64647500
C	1.81243100	1.59697500	-0.03127300
O	2.34501400	2.66450800	0.19056500
O	2.44161500	0.40685900	0.06333000
C	3.83142200	0.46853000	0.45625100
C	4.34840600	-0.95553900	0.50345700
H	3.89835600	0.96655100	1.42825000
H	4.37318400	1.08262500	-0.26945800
H	5.40311400	-0.95749600	0.79820200
H	3.78623900	-1.55101300	1.22993000
H	4.26357800	-1.43428300	-0.47735500
C	-1.51371000	-0.14656300	-1.08192300
C	-2.58169700	0.74547400	-1.31781900
C	-1.76116600	-1.52594600	-1.24796500
C	-3.82644000	0.26831000	-1.69985400
H	-2.44314600	1.81111500	-1.19348100
C	-3.00743000	-1.99699800	-1.63150700
H	-0.95049100	-2.23033200	-1.08053400
C	-4.07014500	-1.10765500	-1.86654400
H	-4.63845000	0.97316800	-1.84721700
H	-3.15711200	-3.06173200	-1.77919800
C	-5.40205300	-1.60184200	-2.27614800
C	-6.19333800	-0.87185100	-3.17877800
C	-5.90618000	-2.81272300	-1.77284600
C	-7.44709400	-1.33886900	-3.56677900
H	-5.80935800	0.05236200	-3.59977800
C	-7.16121800	-3.27798000	-2.15875300
H	-5.32179300	-3.37608500	-1.05159000
C	-7.93648400	-2.54338900	-3.05798900
H	-8.03965800	-0.76478000	-4.27329400
H	-7.53797300	-4.21132700	-1.75022400
H	-8.91467700	-2.90650600	-3.35945200

T3

E = -3471.113938 a.u.

0 1

O	−0.53473900	14.60338900	14.84334600
C	−0.59607700	14.66875800	12.44700300
H	−0.39996800	14.02094900	11.59849300
H	−0.07469100	15.61873000	12.42304300
C	−0.80462400	14.06142600	13.74947500
C	−1.77741800	12.92602400	13.56871500

C	-2.51385100	12.40620700	14.75253000
C	-2.86312700	13.24747300	15.83007000
H	-2.49930700	14.26482100	15.82879500
C	-3.62494500	12.77680300	16.89453400
H	-3.88652600	13.45583600	17.70255800
C	-4.04076600	11.44296600	16.94040800
H	-4.62643400	11.07554100	17.77917600
C	-3.68456100	10.59083000	15.89660800
H	-4.00141300	9.55134200	15.90492600
C	-2.94268300	11.06827500	14.81755900
H	-2.72666900	10.39519200	13.99690500
C	-1.33853900	11.85959100	12.60613600
H	-0.81269300	11.01098700	13.05585900
C	-1.46555000	11.84751400	11.30281100
C	-1.59265300	11.83540900	9.99877800
H	-0.86080200	12.31189800	9.34728600
H	-2.43883400	11.35791400	9.50539100
Ni	-2.61588300	14.54084600	12.56998500
C	-5.67333200	17.02467400	11.82655400
C	-4.41173500	17.49115000	12.29548000
C	-4.34653800	18.54268400	13.21573200
C	-5.49705500	19.17504900	13.71800700
C	-6.70862300	18.71141900	13.25434500
C	-6.78505800	17.67934000	12.32250000
C	-5.85773600	15.92900400	10.83564100
C	-5.59749700	14.54483800	11.06899500
C	-5.83523900	13.61353000	10.05262200
C	-6.31035700	13.97850400	8.78113600
C	-6.55270300	15.31811700	8.57390800
C	-6.34925900	16.25386000	9.58341200
H	-3.37855300	18.88595300	13.55810900
H	-5.43483600	19.98373800	14.43723900
H	-5.66682300	12.56312500	10.24706500
H	-6.48285600	13.24118900	8.00539500
P	-2.87789600	16.61014800	11.78330900
P	-4.79068200	14.01284400	12.64972600
C	-2.98526800	16.65706800	9.95946000
C	-3.42917300	17.79048800	9.26769900
C	-2.75224100	15.46482100	9.26666400
C	-3.62823400	17.74732000	7.88610800
H	-3.66930500	18.69757000	9.81482500
C	-2.98016200	15.38750200	7.88873000
H	-2.44154100	14.58012100	9.81673300
C	-3.41151100	16.53623600	7.21714900
H	-3.60192400	16.48217600	6.14733800
C	-1.57403900	17.77687800	12.31675400
C	-1.09866000	18.82967600	11.52755600
C	-1.04832300	17.57828500	13.59864000
C	-0.10306900	19.68457800	12.01205700

H	-1.48407500	18.97870400	10.52437600
C	-0.04890100	18.41662100	14.10240200
H	-1.35389300	16.72030900	14.19018800
C	0.40579700	19.46693900	13.29897100
H	1.19151500	20.12017300	13.67435300
C	-5.78794600	14.87248600	13.91760400
C	-7.18764700	14.85586500	13.87445300
C	-5.13291000	15.60242600	14.90973800
C	-7.93616400	15.55703200	14.82107800
H	-7.69558900	14.31258300	13.08251800
C	-5.85932400	16.32947900	15.85802700
H	-4.04885000	15.62816100	14.92278500
C	-7.25564500	16.29248200	15.80018400
H	-7.83003100	16.86171000	16.52808100
C	-5.25324000	12.24860900	12.82639000
C	-6.12086900	11.79142300	13.82117000
C	-4.63132100	11.32027400	11.98294200
C	-6.38299600	10.42526200	13.96691700
H	-6.56834400	12.48992400	14.51787100
C	-4.88651700	9.95273800	12.09815400
H	-3.91220800	11.66513000	11.25146200
C	-5.76986300	9.52223400	13.09374300
H	-5.96546400	8.45780000	13.20656300
C	0.52654200	18.13919000	15.46939400
H	0.58719800	17.05791000	15.63410600
H	1.52500100	18.57502700	15.58113000
H	-0.10821300	18.55936300	16.26136000
C	0.44169200	20.79808800	11.14908100
H	1.40276900	20.51696400	10.69917800
H	-0.24454600	21.04135800	10.33150200
H	0.61223500	21.70986100	11.73235600
C	-9.44485000	15.51576600	14.78582400
H	-9.83248800	14.62126400	15.29055200
H	-9.88049200	16.38561600	15.28934200
H	-9.81308200	15.49117400	13.75438600
C	-5.13556200	17.16567100	16.88275000
H	-4.70942000	18.06196500	16.41411900
H	-5.80586000	17.49259800	17.68374000
H	-4.30576000	16.61046800	17.33419200
C	-4.08166800	18.98075500	7.14323100
H	-3.24922600	19.67754800	6.98146700
H	-4.48960300	18.72867600	6.15856900
H	-4.84936000	19.51977300	7.70960500
C	-2.80338900	14.07599700	7.16443700
H	-1.77447500	13.70710100	7.24959900
H	-3.45878400	13.30907100	7.59402200
H	-3.04251100	14.16949400	6.10066900
C	-7.26364600	9.94516500	15.09351100
H	-8.15643300	10.57155100	15.20087000

H	−7.58817300	8.91098100	14.93988000
H	−6.71978500	9.99041300	16.04582500
C	−4.18931600	8.97676100	11.18145500
H	−3.11516000	9.19051600	11.13224700
H	−4.31777500	7.94466000	11.52212900
H	−4.57930300	9.03841500	10.15710300
O	−7.97181500	19.11952400	13.59895100
O	−8.10366400	17.40491700	12.04917900
O	−6.66576900	17.50764600	9.11615500
O	−6.99642800	15.94543500	7.43815900
C	−8.85917500	18.41796300	12.72040000
C	−7.21469900	17.31150400	7.80921300
H	−6.70313300	17.96451200	7.09913100
H	−8.29352100	17.51558400	7.83300700
H	−9.65269400	17.94977100	13.30635300
H	−9.26946100	19.11788000	11.97986600

T3′
E = -3471.092842 a.u.
0 1

C	−7.06526800	15.77940300	10.35483900
C	−5.93567900	16.54208300	10.76606000
C	−6.08510400	17.90472200	11.06507700
C	−7.32814900	18.55254000	11.08627900
C	−8.42119900	17.77923700	10.75424900
C	−8.27953100	16.45059200	10.37754500
C	−7.08101000	14.39467600	9.79478300
C	−6.81480700	13.17336400	10.48392800
C	−6.94314100	11.94335400	9.82206000
C	−7.35182400	11.84295300	8.48393200
C	−7.60930200	13.02915700	7.83064800
C	−7.47696300	14.25632100	8.47404600
H	−5.19699400	18.49681000	11.24782900
H	−7.42390800	19.60302800	11.33539200
H	−6.69884000	11.03297400	10.35688700
H	−7.44243200	10.88542600	7.98446000
P	−4.24965700	15.80602300	10.81508100
P	−5.87939900	13.20465000	12.05426100
C	−3.45397800	16.84261400	9.53468400
C	−2.33257400	17.63045600	9.79136100
C	−4.03231700	16.84084400	8.25898300
C	−1.79281500	18.43969900	8.78402100
H	−1.87281000	17.61902700	10.77385800
C	−3.49816500	17.62260300	7.23625100
H	−4.91032400	16.23404000	8.06136500
C	−2.38632000	18.42552900	7.51999700
H	−1.96536500	19.04557600	6.73108600
C	−3.41700600	16.28228100	12.38900500

C	-2.32708600	15.45673800	12.71201300
C	-3.75968000	17.31870300	13.26283900
C	-1.57573000	15.65720400	13.87262500
H	-2.06163700	14.62862300	12.06366700
C	-3.01153100	17.55460300	14.42273700
H	-4.62856600	17.93549700	13.07557300
C	-1.93057600	16.71546600	14.71690400
H	-1.35775600	16.88700900	15.62664800
C	-6.70594200	14.39619400	13.17075300
C	-8.05835000	14.72473400	13.03903800
C	-5.95488800	14.93845200	14.21825100
C	-8.65573400	15.63681300	13.91188300
H	-8.64574300	14.29876800	12.23117200
C	-6.54740000	15.80664700	15.14126600
H	-4.90140900	14.69140200	14.31348500
C	-7.88889800	16.16040200	14.95944100
H	-8.35040600	16.85986600	15.65426300
C	-6.24764000	11.64259500	12.93507100
C	-7.51708300	11.04631400	12.89161400
C	-5.26721800	11.11847200	13.77861800
C	-7.79814300	9.91572300	13.65835500
H	-8.29010400	11.46462000	12.25382600
C	-5.52842500	9.98948700	14.56777000
H	-4.29127200	11.59139100	13.82501000
C	-6.79093800	9.40054400	14.48844600
H	-7.00227600	8.51909200	15.09075100
C	-3.35308400	18.71549800	15.32682900
H	-3.21577900	18.45924400	16.38272900
H	-2.70983300	19.57983400	15.11594200
H	-4.39025800	19.03648100	15.18789100
C	-0.41048600	14.74682800	14.17694600
H	-0.46380400	13.83102500	13.57980800
H	0.54203700	15.24013300	13.94288000
H	-0.38176600	14.47490200	15.23831600
C	-10.08342500	16.07883500	13.69757600
H	-10.59981500	16.25363000	14.64750800
H	-10.11833400	17.01649400	13.12793300
H	-10.64869500	15.32962200	13.13349200
C	-5.76275400	16.33070800	16.31854400
H	-6.02860800	15.79074500	17.23667700
H	-4.68814400	16.21127000	16.15941800
H	-5.96594600	17.39222100	16.49744300
C	-0.58755600	19.29830700	9.07466200
H	-0.25004400	19.83017200	8.17951700
H	-0.80494600	20.04540500	9.84834600
H	0.24275000	18.68195300	9.43602600
C	-4.08591300	17.56121000	5.84808300
H	-3.82764900	18.44768300	5.25962300
H	-3.71063300	16.68075300	5.31008600

H	−5.17791900	17.47653200	5.88111700
C	−9.15346600	9.25305800	13.59855400
H	−9.85751600	9.84022300	13.00095900
H	−9.08774800	8.25361600	13.15041700
H	−9.58150500	9.12951500	14.60032200
C	−4.45595200	9.43285100	15.47243400
H	−4.15860600	10.16623700	16.23195700
H	−4.79811100	8.53210900	15.99100200
H	−3.55434200	9.17531900	14.90421900
O	−9.74927800	18.13205600	10.73185300
O	−9.52168900	15.91885900	10.10403400
O	−7.74435900	15.26932500	7.57799300
O	−7.98256500	13.22648400	6.52572200
C	−10.42037800	17.03062300	10.10495800
C	−8.29047300	14.62032400	6.42474500
H	−7.82732500	15.03096700	5.52561600
H	−9.38151500	14.75436900	6.41260000
H	−11.31213500	16.77574900	10.68080300
H	−10.67236500	17.30108600	9.07018000
O	−0.97430100	12.32107400	11.29843500
C	−3.27106000	11.72588300	11.11519900
H	−3.90771400	11.14385400	10.44896900
H	−3.15410300	11.25358200	12.08677100
C	−2.01924600	12.33050700	10.61618000
C	−2.28085000	13.25188800	9.46712000
C	−1.27107400	14.26824300	9.07809900
C	−0.24006300	14.66538100	9.96212600
H	−0.17209100	14.18676100	10.92619200
C	0.72625000	15.59173100	9.58051400
H	1.50895700	15.85897400	10.28671300
C	0.71725500	16.15024100	8.30163600
H	1.48361900	16.85917900	7.99934900
C	−0.29497500	15.77985300	7.41831500
H	−0.33352100	16.20891400	6.42066800
C	−1.27034900	14.86282400	7.80003600
H	−2.03690600	14.60360300	7.08641800
C	−3.30561500	12.79305100	8.49420100
H	−3.66591000	11.78029900	8.66269000
C	−3.87659200	13.37793500	7.45820700
C	−4.48660000	13.86597200	6.40810600
H	−5.41285000	14.43229400	6.49093300
H	−4.08768800	13.75159100	5.39918200
Ni	−4.02496500	13.59614100	10.9820930

T3″

E = -3471.101548 a.u.

0 1

C	−0.68731600	14.17946700	14.00759400
C	−1.87439000	13.24605600	13.85522900

C	-2.63318200	12.88778900	15.07955600
C	-2.68401700	13.77100200	16.17934900
H	-2.10127000	14.68553400	16.13726400
C	-3.43777900	13.47750700	17.30941400
H	-3.45381700	14.17959800	18.13989300
C	-4.15884400	12.28101300	17.39156900
H	-4.74360200	12.04914900	18.27745900
C	-4.10137400	11.38616200	16.32504800
H	-4.64355400	10.44518800	16.37113100
C	-3.35385500	11.68565300	15.18498500
H	-3.34867500	10.98407100	14.35856100
C	-1.50697500	12.11333100	12.94182700
H	-1.28630700	11.15365400	13.41857100
C	-1.32101000	12.20281200	11.64736600
C	-1.16409400	12.26511700	10.34834000
H	-0.23100700	12.60131900	9.89826600
H	-1.96326900	11.99298100	9.65703100
Ni	-2.66750600	14.75124200	12.78449900
C	-5.77921600	17.16110200	11.91273700
C	-4.51950900	17.65001300	12.36481400
C	-4.45924000	18.66850500	13.32107400
C	-5.61373100	19.24520300	13.87830600
C	-6.82366600	18.76419000	13.42753900
C	-6.89612900	17.76683400	12.45844200
C	-5.95397000	16.10067700	10.88233100
C	-5.62944500	14.72002200	11.04104100
C	-5.85366400	13.82749500	9.98716400
C	-6.37512700	14.23043200	8.74611400
C	-6.67789100	15.56699300	8.60939200
C	-6.48846800	16.46185700	9.65811900
H	-3.49108500	19.03086200	13.64482600
H	-5.55587700	20.02927700	14.62468300
H	-5.63570400	12.77731900	10.12698100
H	-6.53381200	13.52398700	7.93932600
P	-2.98613500	16.80088600	11.81359800
P	-4.80543100	14.13325400	12.59131800
C	-3.12169100	16.80991700	9.99260500
C	-3.60717900	17.91448100	9.28195900
C	-2.82158800	15.62274800	9.31901300
C	-3.79232100	17.84288900	7.89985400
H	-3.88033700	18.82085300	9.81496500
C	-3.02718500	15.51858000	7.93912200
H	-2.46291400	14.76488200	9.88274800
C	-3.50768700	16.63562000	7.24873900
H	-3.68114600	16.56009100	6.17721700
C	-1.65197400	17.96831300	12.25163500
C	-1.09788200	18.87168100	11.33624300
C	-1.16661800	17.92876900	13.56329400
C	-0.07410900	19.73944200	11.72422000

H	-1.45234900	18.89304900	10.31127600
C	-0.14713800	18.79225100	13.97658000
H	-1.55740400	17.19410300	14.25766000
C	0.38413500	19.68985600	13.04725100
H	1.18640800	20.35805300	13.35513600
C	-5.85673200	14.87155600	13.89034000
C	-7.25455600	14.80720300	13.82308300
C	-5.24268600	15.57903400	14.92376000
C	-8.04126400	15.43881600	14.78739800
H	-7.73148500	14.28731400	12.99686500
C	-6.00890900	16.23757500	15.89051800
H	-4.16044100	15.63751500	14.95661700
C	-7.40168400	16.15544700	15.80757400
H	-8.00661200	16.67264000	16.54938400
C	-5.18439600	12.34207700	12.62697400
C	-6.07724300	11.77499500	13.53926500
C	-4.45473900	11.50550300	11.77282100
C	-6.25269100	10.38805900	13.60007500
H	-6.61030800	12.40482500	14.24155000
C	-4.60496600	10.11812300	11.81674900
H	-3.73402800	11.93789500	11.09108700
C	-5.50795500	9.57624700	12.73946000
H	-5.62104700	8.49566800	12.79824500
C	0.37066000	18.71918300	15.39283600
H	0.57751900	17.68068500	15.67562600
H	1.29206800	19.29745800	15.51327100
H	-0.36394300	19.11069900	16.10871400
C	0.54582000	20.69566200	10.73315000
H	1.55817700	20.37655100	10.45413900
H	-0.04603800	20.75545700	9.81431400
H	0.62910600	21.70662700	11.14901900
C	-9.54682800	15.34744400	14.72329300
H	-9.90678600	14.39068300	15.12319700
H	-10.02126600	16.14220200	15.30890100
H	-9.90080600	15.42015500	13.68898200
C	-5.32675100	17.03635400	16.97143400
H	-4.79188900	17.89157200	16.53984600
H	-6.04391300	17.42124200	17.70293000
H	-4.58944100	16.42339800	17.50243500
C	-4.29211600	19.04176200	7.13068700
H	-3.46590600	19.70451700	6.84211300
H	-4.80171800	18.74094200	6.20878900
H	-4.98769600	19.63391600	7.73504300
C	-2.79214700	14.20498100	7.23612300
H	-1.85107900	13.74447600	7.55518200
H	-3.59754300	13.49688400	7.47194900
H	-2.76394200	14.32763100	6.14891700
C	-7.21198500	9.79379800	14.60282000
H	-8.25484000	9.99523500	14.32624800

H	−7.09240100	8.70838300	14.67686300
H	−7.05246200	10.22310900	15.59851000
C	−3.78404600	9.24128700	10.90298400
H	−2.75070400	9.60107300	10.84671500
H	−3.76934300	8.20374500	11.25109800
H	−4.18590100	9.24078200	9.88113400
O	−8.08863700	19.12606900	13.81645800
O	−8.21351900	17.47172800	12.20094600
O	−6.85249300	17.72439800	9.25262300
O	−7.16428400	16.23169700	7.51277000
C	−8.97898400	18.43288500	12.93509900
C	−7.46281000	17.55609800	7.96971800
H	−7.04434900	18.28264700	7.27110400
H	−8.55121300	17.67274800	8.06524200
H	−9.74076000	17.91668300	13.52298500
H	−9.43195300	19.14836300	12.23541900
C	0.33345600	14.09616500	14.87712300
H	0.38414200	13.27911400	15.58778400
H	1.12333200	14.84178900	14.88036600
O	−0.88327800	15.14421300	13.09724300

T3-vinyl

E = -3433.043914 a.u.

0 1

O	0.19575200	14.56989800	14.43986100
C	−0.60991800	14.35547200	12.18891600
H	−0.74497600	13.54400900	11.47258500
H	0.00641000	15.18220600	11.84254900
C	−0.50969000	13.98895200	13.59665400
C	−1.68376800	13.09164400	13.88750900
C	−2.41752000	13.24313300	15.17821400
C	−2.15180900	14.31002800	16.06683000
H	−1.32151200	14.96716000	15.84996500
C	−2.89385000	14.48230700	17.23388100
H	−2.65097100	15.30873500	17.89753300
C	−3.93065400	13.60732600	17.56117400
H	−4.50687000	13.74477600	18.47195900
C	−4.20102400	12.54090100	16.70299100
H	−4.99936100	11.84066900	16.93659700
C	−3.45288300	12.35544900	15.54402400
H	−3.69548200	11.51218700	14.91148800
Ni	−2.56090800	14.49007300	12.59193500
C	−5.57264000	16.98146300	12.00635400
C	−4.26644300	17.37100000	12.42757500
C	−4.10595300	18.29851900	13.46143700
C	−5.20084900	18.88483300	14.12038500
C	−6.45478500	18.51058800	13.68957500
C	−6.62849800	17.59931000	12.65100000

C	-5.84637000	15.98701800	10.93395900
C	-5.57046300	14.59010700	11.03333200
C	-5.87156100	13.74293200	9.96299300
C	-6.43394600	14.20977800	8.76120400
C	-6.69604200	15.55939300	8.68495500
C	-6.42435800	16.41176900	9.75212500
H	-3.10537200	18.57904400	13.76813600
H	-5.06577600	19.59570400	14.92742000
H	-5.68298300	12.68161700	10.05642700
H	-6.65642100	13.53885300	7.93914800
P	-2.79416600	16.52297200	11.71923800
P	-4.75097600	13.94990600	12.56852500
C	-3.05491700	16.66227400	9.91589100
C	-3.53909200	17.83337700	9.32125800
C	-2.85508500	15.51951200	9.13637100
C	-3.81292200	17.87556800	7.95215000
H	-3.74615400	18.70490600	9.93552200
C	-3.15489600	15.52605100	7.77076400
H	-2.50632000	14.60827400	9.61502400
C	-3.62521900	16.71209300	7.19670100
H	-3.86860300	16.72526500	6.13638500
C	-1.40969700	17.64537900	12.13585600
C	-0.97275400	18.68595000	11.30535600
C	-0.75367200	17.40733900	13.34682000
C	0.10765800	19.48717800	11.68249400
H	-1.45511600	18.86243500	10.35031300
C	0.33785600	18.18948700	13.74248700
H	-1.04662700	16.56892300	13.96755900
C	0.75030600	19.22486200	12.90068400
H	1.60820900	19.83040600	13.18745200
C	-5.91666800	14.54431200	13.84632600
C	-7.29798600	14.40229700	13.67845400
C	-5.41482700	15.26288900	14.93506800
C	-8.18766300	14.96790700	14.59653300
H	-7.68572700	13.87671300	12.80985800
C	-6.28372100	15.86233800	15.84877800
H	-4.34402600	15.37632000	15.05360600
C	-7.66344600	15.70081700	15.66636200
H	-8.34726100	16.17012400	16.37147300
C	-5.01211900	12.14260300	12.44335300
C	-5.88209000	11.43054700	13.27646700
C	-4.21567700	11.44460500	11.52694900
C	-5.96691200	10.03720100	13.19556100
H	-6.47611200	11.95926500	14.01377400
C	-4.29242100	10.05290000	11.41461600
H	-3.49124200	11.98749600	10.92537900
C	-5.16928700	9.36625800	12.25964900
H	-5.22035000	8.28092900	12.20056500
C	1.05572800	17.86219100	15.02781300

H	1.20629000	16.77994800	15.09899000
H	2.02550600	18.36677300	15.08563100
H	0.46670700	18.17112200	15.90151000
C	0.58111900	20.61357700	10.79475000
H	1.66293900	20.56111500	10.62532400
H	0.08558900	20.58652500	9.81917000
H	0.37297600	21.59183500	11.24700100
C	-9.67726100	14.82028100	14.39774400
H	-9.98049100	13.76640700	14.40737500
H	-10.24003300	15.33458900	15.18359700
H	-9.98570000	15.23400400	13.42947100
C	-5.75097700	16.67547500	17.00129900
H	-4.72837400	17.00820400	16.80519800
H	-6.37423800	17.55848300	17.18116500
H	-5.73094500	16.08510800	17.92556000
C	-4.30546200	19.15081500	7.31215300
H	-3.47420000	19.83238100	7.09000300
H	-4.82180700	18.95125900	6.36704800
H	-4.99263500	19.68364800	7.97838200
C	-3.03593100	14.26063000	6.95894400
H	-2.13779300	13.69437900	7.22840800
H	-3.90047700	13.60888900	7.14152300
H	-2.99881600	14.47104400	5.88552700
C	-6.90742800	9.27150500	14.09497400
H	-7.83564200	9.01100400	13.56951200
H	-6.45658300	8.33468200	14.43989600
H	-7.18227800	9.86092000	14.97536300
C	-3.41365400	9.32535900	10.42679000
H	-2.38378200	9.69599300	10.48012200
H	-3.40084300	8.24824200	10.61929300
H	-3.76154300	9.47508400	9.39654500
O	-7.67531800	18.92061700	14.15906600
O	-7.96955400	17.41001300	12.42403800
O	-6.76276600	17.69864300	9.40689100
O	-7.21216100	16.27761000	7.63651200
C	-8.65642800	18.22391900	13.38075400
C	-7.43457800	17.59705900	8.14780900
H	-7.01678700	18.32853300	7.45381000
H	-8.51167900	17.75512500	8.29569000
H	-9.25135200	17.58543100	14.04042300
H	-9.28548100	18.94992700	12.85345300
C	-1.64437000	11.74708900	13.28740000
C	-0.59741200	11.16700300	12.67565000
H	-2.54846600	11.15585300	13.36964100
H	-0.68377600	10.16547700	12.26260900
H	0.36797400	11.65481500	12.58148900

T3-alkynyl

E = -3840.522195 a.u.

0 1

O	−0.38858800	14.50681400	14.82298900
C	−0.50290600	14.63657800	12.42214800
H	−0.31742400	13.99455100	11.56469800
H	0.01722500	15.58751100	12.40312500
C	−0.67595300	14.00132400	13.72025800
C	−1.63969400	12.85980300	13.54416200
C	−2.31136100	12.25902200	14.72580800
C	−2.73256200	13.06381300	15.79975600
H	−2.49162100	14.11765200	15.78674400
C	−3.41913200	12.51217900	16.87734300
H	−3.74298600	13.15653600	17.69088000
C	−3.68342900	11.14055000	16.92261500
H	−4.21063700	10.70989700	17.77007800
C	−3.25212100	10.32943900	15.87275100
H	−3.45182300	9.26154200	15.88970900
C	−2.57555600	10.88287000	14.78744400
H	−2.25548100	10.24905300	13.96785000
Ni	−2.52357400	14.46859500	12.55952400
C	−5.61276900	16.82996400	11.67452000
C	−4.38576000	17.35600900	12.16890900
C	−4.38428100	18.45473300	13.03375000
C	−5.57113900	19.07884700	13.45639900
C	−6.74967100	18.55749400	12.96985800
C	−6.76065500	17.47575500	12.09155900
C	−5.72102400	15.67696900	10.73863700
C	−5.45450700	14.31250700	11.06914500
C	−5.61978700	13.31927500	10.09872700
C	−6.02556600	13.60060900	8.78225300
C	−6.27459800	14.92114000	8.48151800
C	−6.14367800	15.91891100	9.44312500
H	−3.44023800	18.84223500	13.39534700
H	−5.56000500	19.92278000	14.13666200
H	−5.44277200	12.28584900	10.36136300
H	−6.14287300	12.81395800	8.04574200
P	−2.81399900	16.50541200	11.72912000
P	−4.68529600	13.88998300	12.69857500
C	−2.85269100	16.59845500	9.90236900
C	−3.28724300	17.75926600	9.25398000
C	−2.56614200	15.44794900	9.15664100
C	−3.41658600	17.79433800	7.86290100
H	−3.56386300	18.63208600	9.83811700
C	−2.72777200	15.44622300	7.76758400
H	−2.25705500	14.54303100	9.67351200
C	−3.14269300	16.62891000	7.14078600
H	−3.26486500	16.63538100	6.05947200

C	-1.55952100	17.68774300	12.34192800
C	-1.05091800	18.75194300	11.58931300
C	-1.09889900	17.47833100	13.64628200
C	-0.08910600	19.60861700	12.13478200
H	-1.38108500	18.90673100	10.56765000
C	-0.12670800	18.31167000	14.20808500
H	-1.42491100	16.60886200	14.20860800
C	0.36132800	19.37352500	13.44064100
H	1.13079500	20.01976800	13.85897600
C	-5.68244300	14.83025900	13.90683100
C	-7.08273800	14.81983500	13.86277900
C	-5.02405300	15.59910200	14.86618400
C	-7.82692100	15.57130600	14.77285700
H	-7.59300400	14.24011700	13.09879600
C	-5.74656600	16.37582000	15.77834000
H	-3.93898500	15.61830500	14.87930900
C	-7.14219600	16.34623400	15.71903200
H	-7.71392400	16.95480900	16.41662200
C	-5.14421700	12.14270100	13.02169500
C	-5.96937800	11.79041700	14.09448000
C	-4.57430000	11.12979100	12.23847800
C	-6.25413800	10.44962300	14.36944000
H	-6.37338700	12.55494100	14.74635600
C	-4.85789400	9.78415000	12.48495000
H	-3.85695300	11.38166300	11.46926700
C	-5.70858100	9.46193800	13.54602600
H	-5.92274600	8.41613900	13.75674300
C	0.39547900	18.00713300	15.59023300
H	0.51374200	16.92469700	15.70976200
H	1.35823500	18.49525700	15.77244700
H	-0.30300400	18.35103200	16.36459800
C	0.45472800	20.76674500	11.33244800
H	1.54273800	20.84759000	11.43398400
H	0.22130100	20.65862400	10.26849400
H	0.02710900	21.72004000	11.66993700
C	-9.33580600	15.54686400	14.73796800
H	-9.73850400	14.75205300	15.37926900
H	-9.75742500	16.49343400	15.09367500
H	-9.70222800	15.36335900	13.72247700
C	-5.01888000	17.26278700	16.75653300
H	-4.62088600	18.14916800	16.24541700
H	-5.67916400	17.60751700	17.55834900
H	-4.16874500	16.74238100	17.21118700
C	-3.86341900	19.06199300	7.17533000
H	-3.09823800	19.84479600	7.24712800
H	-4.06319000	18.89412700	6.11202300
H	-4.77451200	19.45872500	7.63888400
C	-2.52132700	14.18911800	6.96059700
H	-1.73472700	13.56062800	7.38534200

H	-3.44077200	13.59035600	6.94303300
H	-2.25625400	14.41927800	5.92360800
C	-7.08636300	10.08948700	15.57444800
H	-7.96363400	10.73947700	15.66787100
H	-7.43283100	9.05208100	15.52987500
H	-6.49401800	10.20794100	16.49059500
C	-4.21032700	8.70839100	11.64791900
H	-4.59209900	8.70830900	10.61921900
H	-3.12735600	8.86580500	11.59161000
H	-4.38948000	7.71369900	12.06772200
O	-8.03776700	18.93881000	13.24826900
O	-8.05980400	17.14243700	11.79218800
O	-6.47813700	17.13268000	8.89304000
O	-6.68683600	15.46610500	7.29401800
C	-8.86176700	18.18874000	12.34786200
C	-6.77577600	16.87895200	7.51552100
H	-6.04056000	17.38820900	6.88512300
H	-7.79369500	17.21850500	7.29668000
H	-9.69584000	17.75178100	12.89935600
H	-9.21438300	18.84646200	11.54133100
C	-1.45680500	12.04601400	12.38755400
C	-1.30815900	11.38650200	11.36313800
Si	-1.16924600	10.80564700	9.64679800
C	-2.84279600	11.00622900	8.77970900
H	-2.75406500	10.81844700	7.70247600
H	-3.58167400	10.30372500	9.18055000
H	-3.23881200	12.01803100	8.91492000
C	-0.63958200	8.99574400	9.56021100
H	-1.36834300	8.33976300	10.04951400
H	-0.54155100	8.66703900	8.51806000
H	0.32753400	8.84765900	10.05348400
C	0.12566800	11.88858100	8.79426500
H	1.11507300	11.72318700	9.23534600
H	0.19239400	11.67197600	7.72090900
H	-0.11086200	12.95222800	8.91384000

References

(1) Yan, B.; Zuo, L.; Chang, X.; Liu, T.; Cui, M.; Liu, Y.; Sun, H.; Chen, W.; Guo, W. *Org. Lett.* **2021**, *23*, 351.

(2) (a) Liu, T.; Fang, Y.; Zuo, L.; Yang, Y.; Liu, Y.; Chen, W.; Dang, L.; Guo, W. *Org. Chem. Front.* **2021**, *8*, 1902; (b) Zuo, L.; Ma, P.; Liu, T.; Chen, X.; Lavroff, R. H.; Chen, W.-P.; Houk, K. N.; Guo, W. *Org. Lett.* **2021**, *23*, 7330.

(3) (a) Brown, R. W.; Zamani, F.; Gardiner, M. G.; Yu, H.; Pyne, S. G.; Hyland, C. J. T. *Chem. Sci.* **2019**, *10*, 9051; (b) Zamani, F.; Pyne, S. G.; Hyland, C. J. T. *J. Org. Chem.* **2017**, *82*, 6819.

(4) Song, Q.; Chen, W.; Ma, R.; Yu, A.; Li, Q.; Chang, Y.; He, L. *ChemSusChem* **2015**, *8*, 821.

(5) Pan, T.; Gao, X.; Yang, S.; Wang, L.; Hu, Y.; Liu, M.; Wang, W.; Wu, Y.; Zheng, B.; Guo, H. *Org. Lett.* **2021**, *23*, 5750.

(6) Gaussian 16, Revision A.03, M. J. Frisch, G. W. Trucks, H. B. Schlegel, G. E. Scuseria, M. A. Robb, J. R. Cheeseman, G. Scalmani, V. Barone, G. A. Petersson, H. Nakatsuji, X. Li, M. Caricato, A. V. Marenich, J. Bloino, B. G. Janesko, R. Gomperts, B. Mennucci, H. P. Hratchian, J. V. Ortiz, A. F. Izmaylov, J. L. Sonnenberg, D. Williams-Young, F. Ding, F. Lipparini, F. Egidi, J. Goings, B. Peng, A. Petrone, T. Henderson, D. Ranasinghe, V. G. Zakrzewski, J. Gao, N. Rega, G. Zheng, W. Liang, M. Hada, M. Ehara, K. Toyota, R. Fukuda, J. Hasegawa, M. Ishida, T. Nakajima, Y. Honda, O. Kitao, H. Nakai, T. Vreven, K. Throssell, J. A. Montgomery, Jr., J. E. Peralta, F. Ogliaro, M. J. Bearpark, J. J. Heyd, E. N. Brothers, K. N. Kudin, V. N. Staroverov, T. A. Keith, R. Kobayashi, J. Normand, K. Raghavachari, A. P. Rendell, J. C. Burant, S. S. Iyengar, J. Tomasi, M. Cossi, J. M. Millam, M. Klene, C. Adamo, R. Cammi, J. W. Ochterski, R. L. Martin, K. Morokuma, O. Farkas, J. B. Foresman, and D. J. Fox, Gaussian, Inc., Wallingford CT, 2016.

(7) Grimme, S. *J. Comp. Chem.* **2006**, *27*, 1787.

(8) Lu, T.; Chen, F. *J. Comput. Chem.* **2012**, *33*, 580.

NMR spectra

^1H NMR (400 MHz, $CDCl_3$)

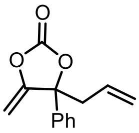

1b

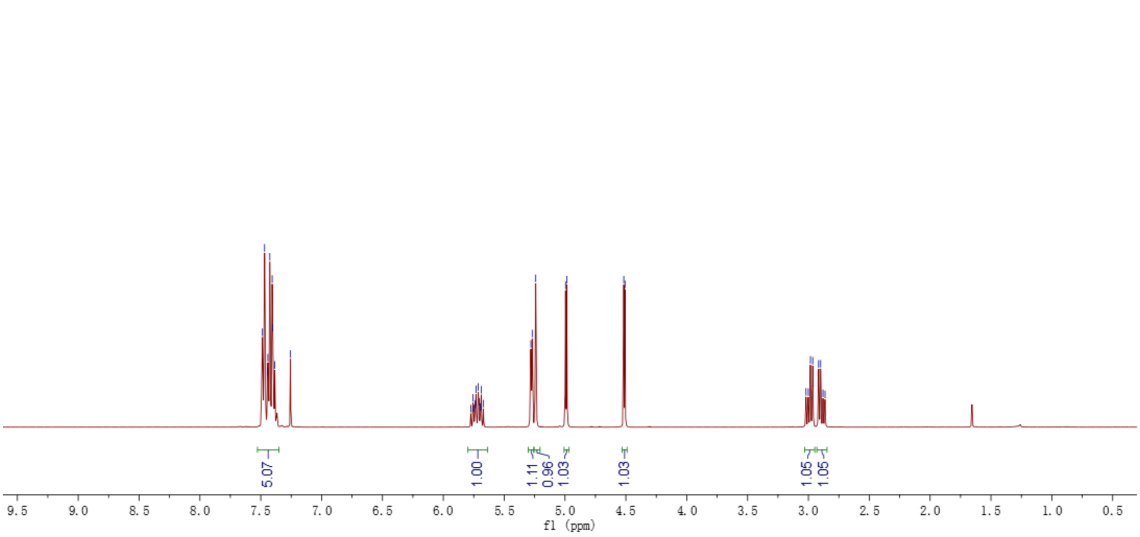

^{13}C NMR (100 MHz, $CDCl_3$)

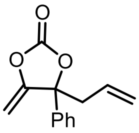

1b

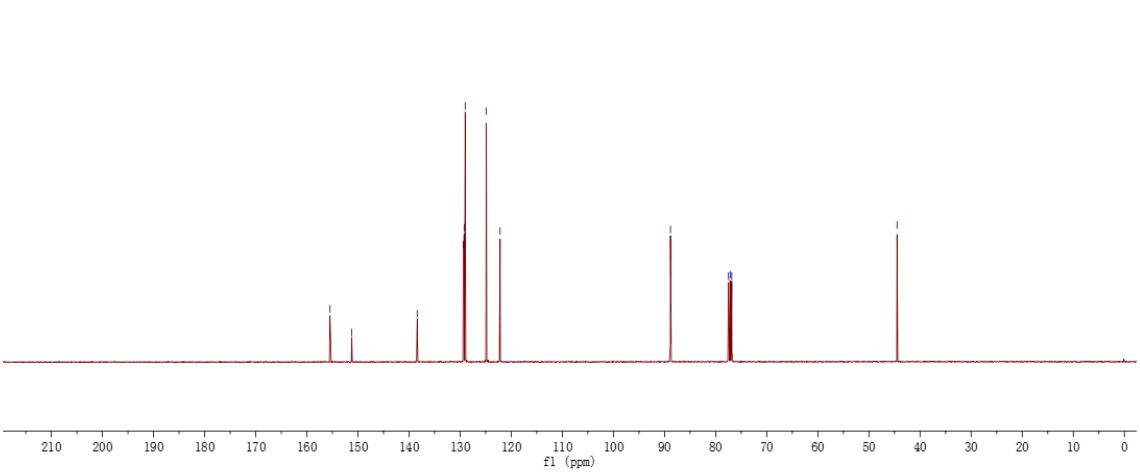

^{1}H NMR (400 MHz, $CDCl_3$)

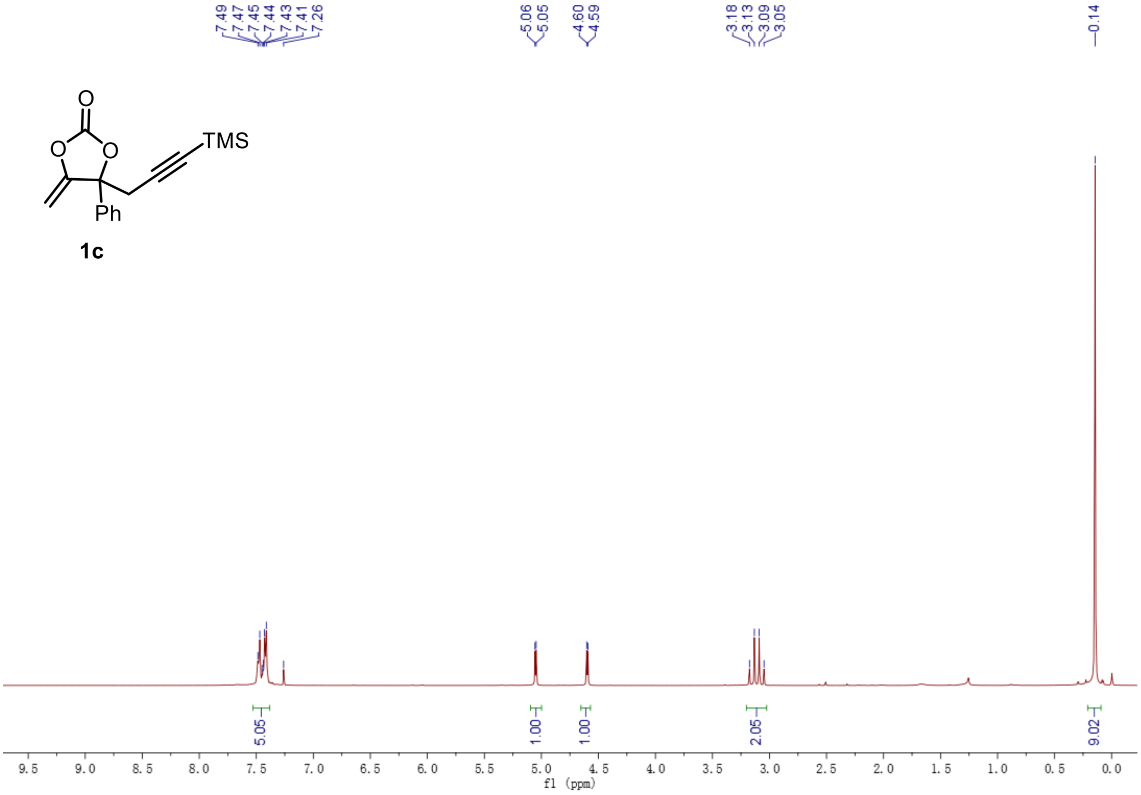

^{13}C NMR (100 MHz, $CDCl_3$)

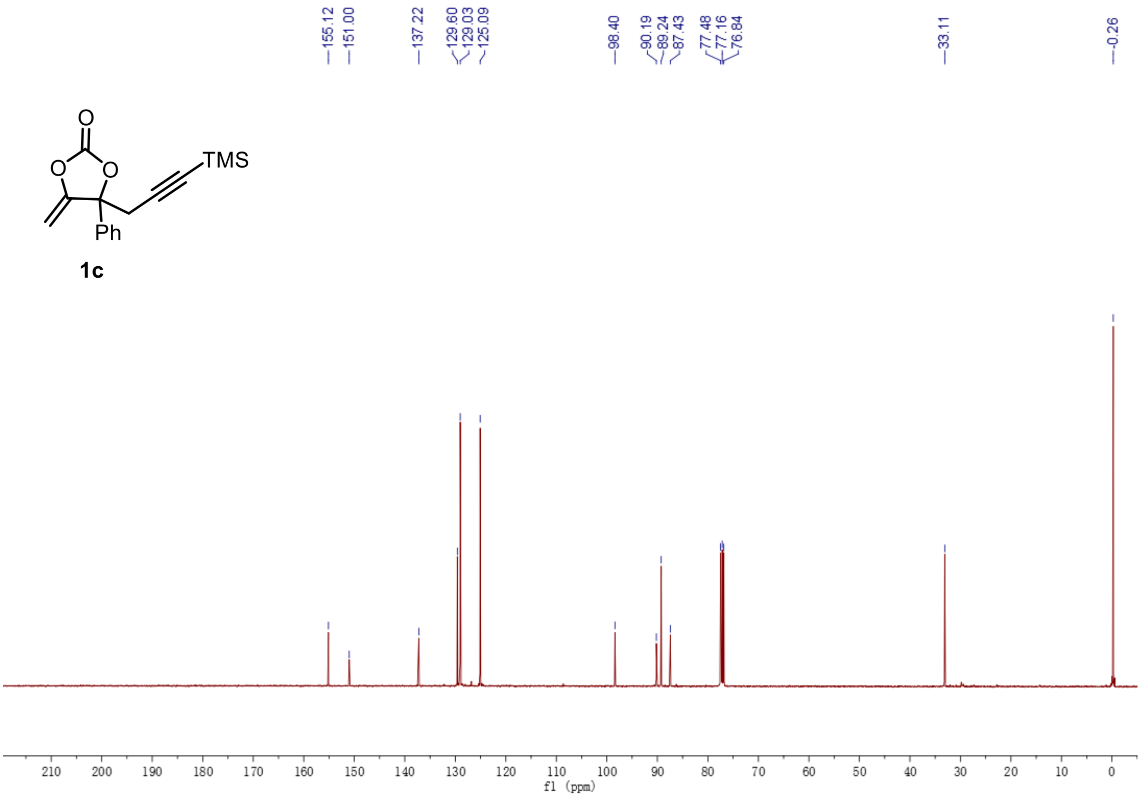

^{1}H NMR (400 MHz, $CDCl_3$)

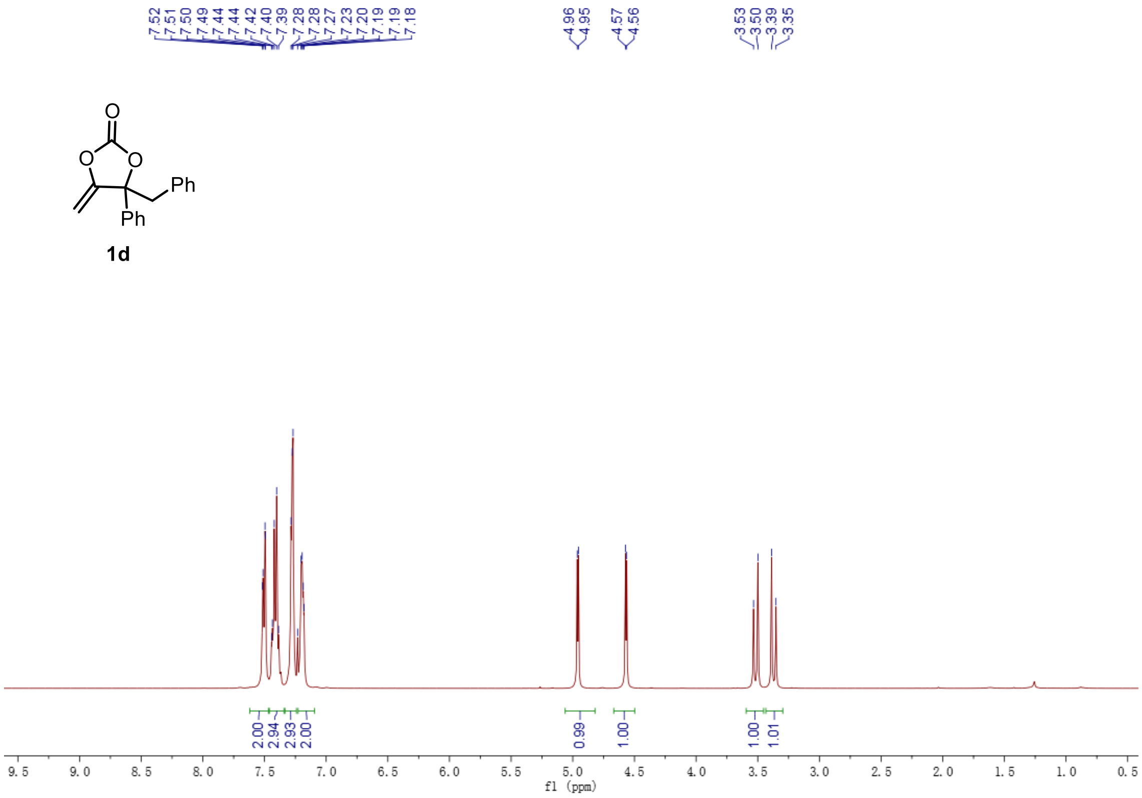

^{13}C NMR (100 MHz, $CDCl_3$)

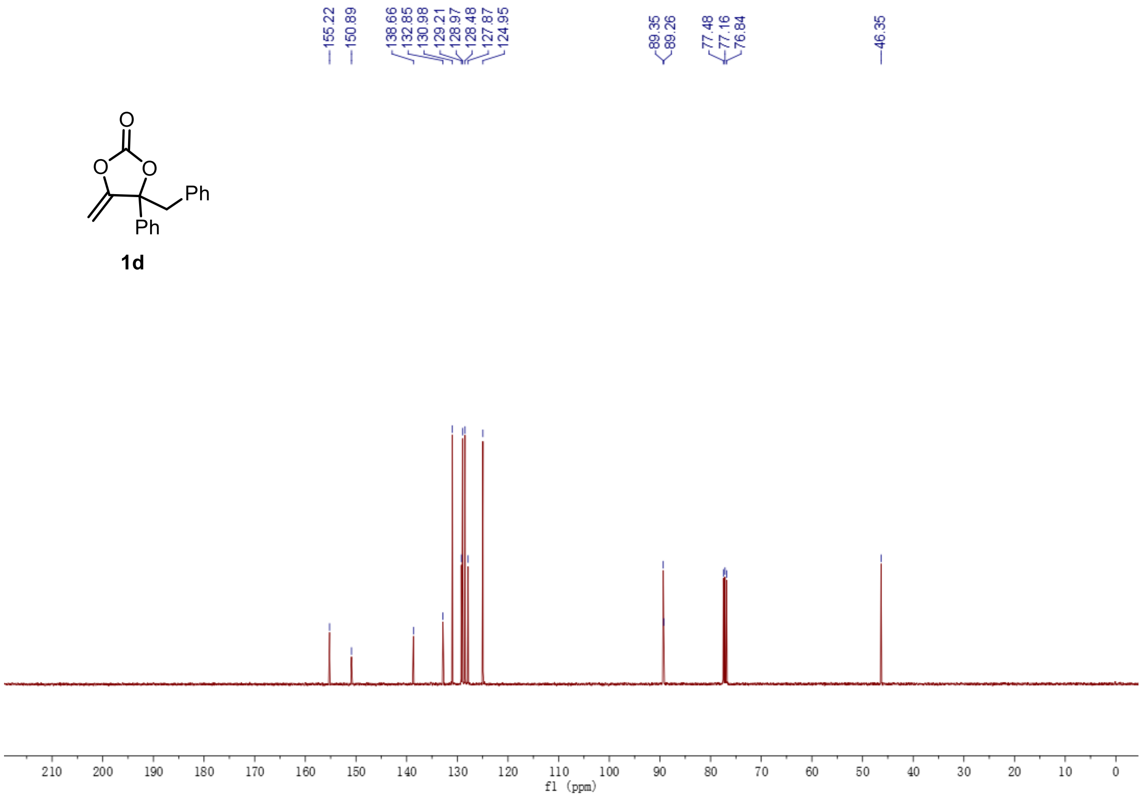

^{1}H NMR (400 MHz, $CDCl_3$)

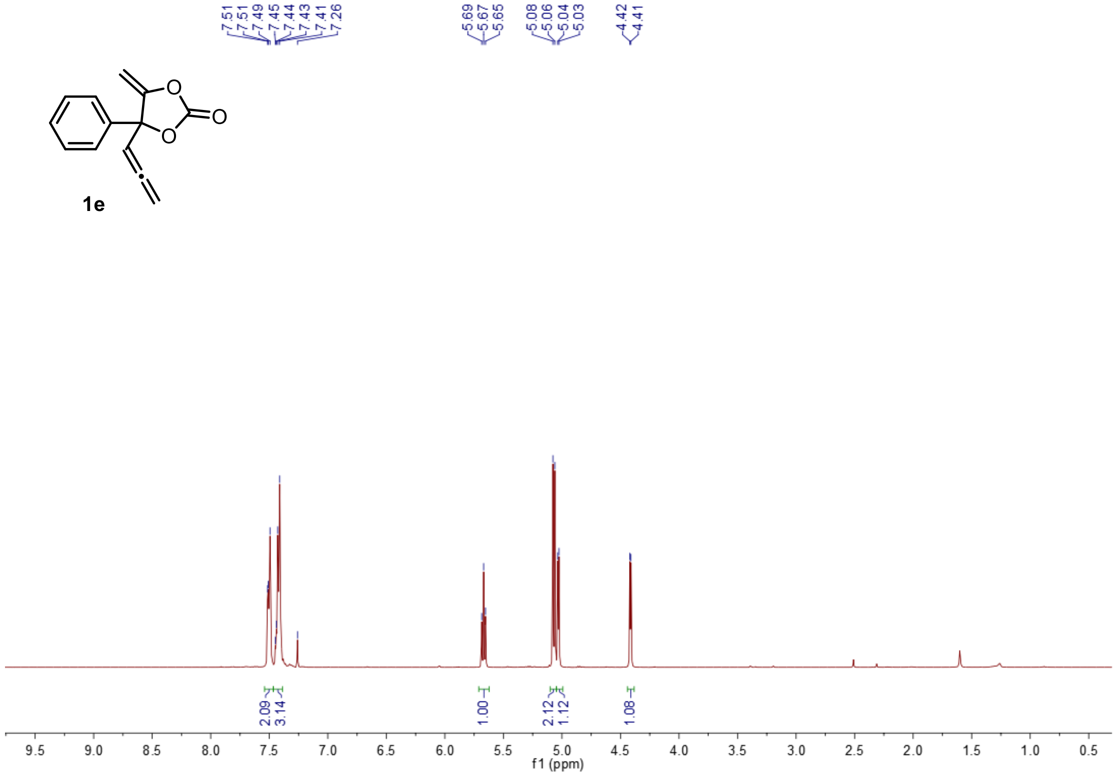

^{13}C NMR (100 MHz, $CDCl_3$)

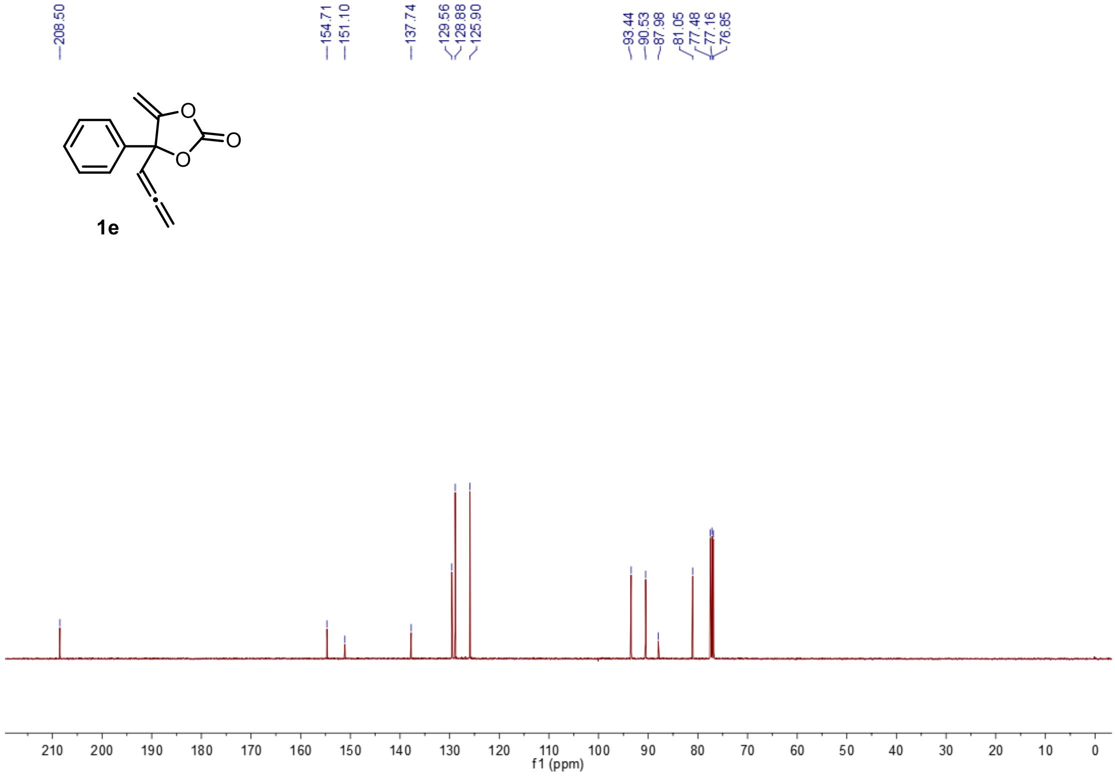

^{1}H NMR (400 MHz, $CDCl_3$)

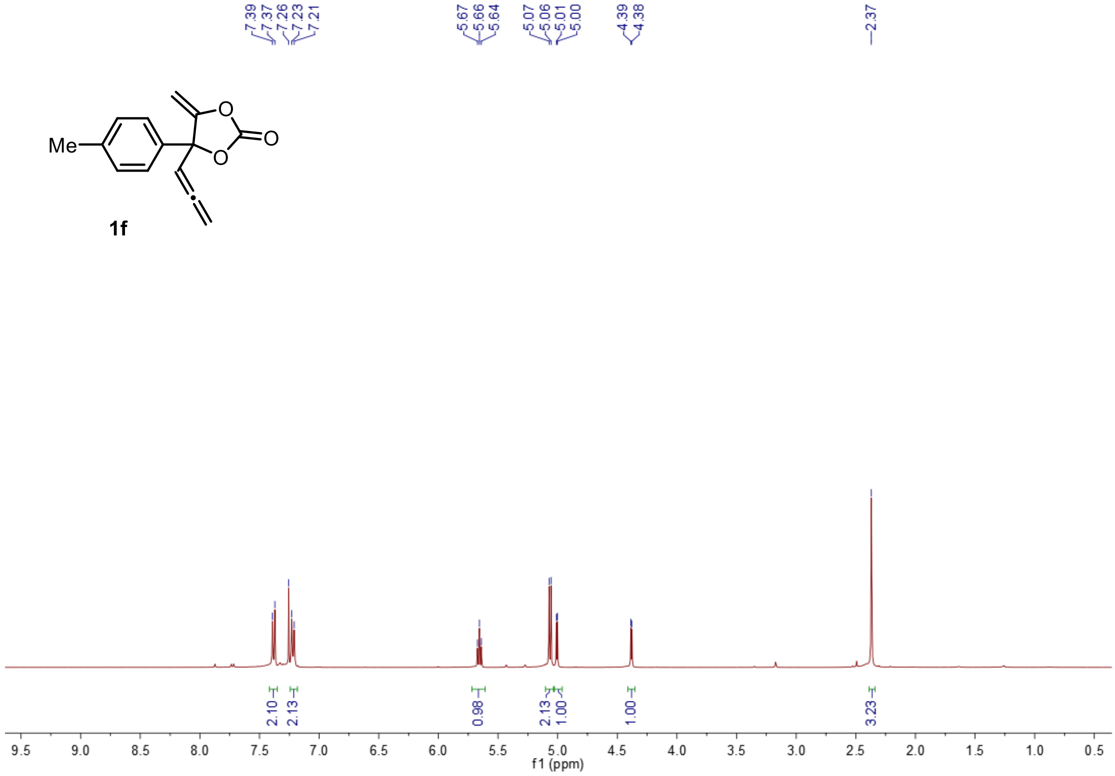

^{13}C NMR (100 MHz, $CDCl_3$)

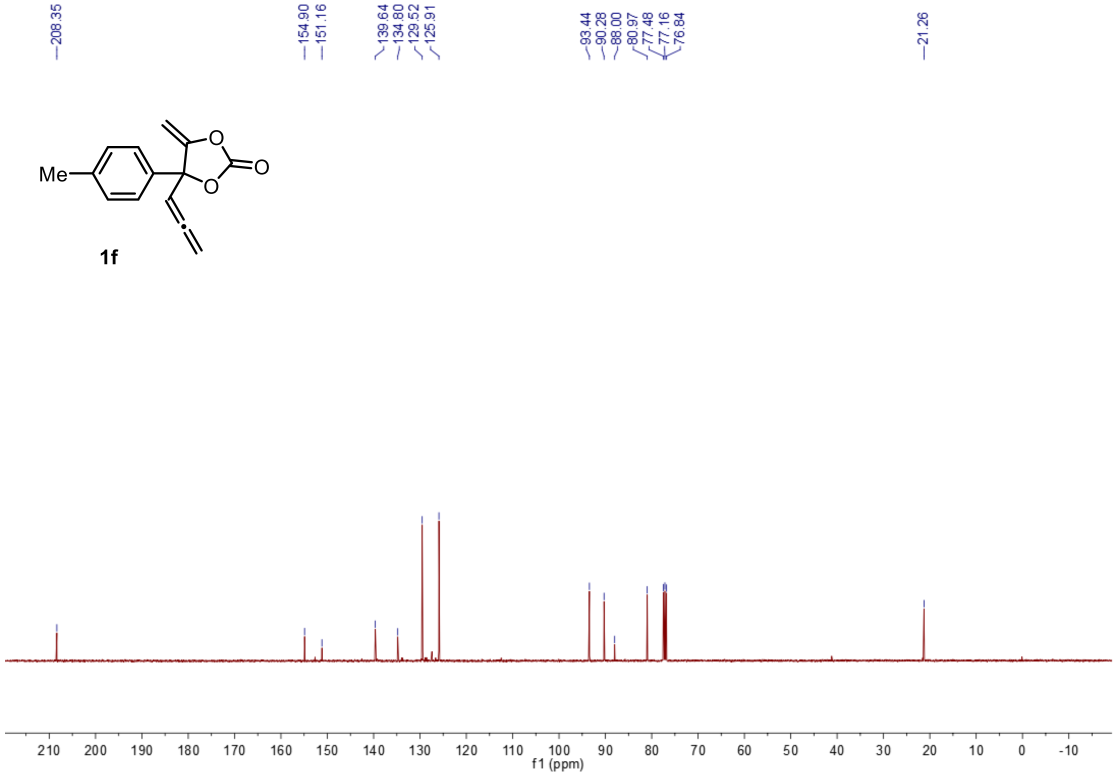

^{1}H NMR (400 MHz, $CDCl_3$)

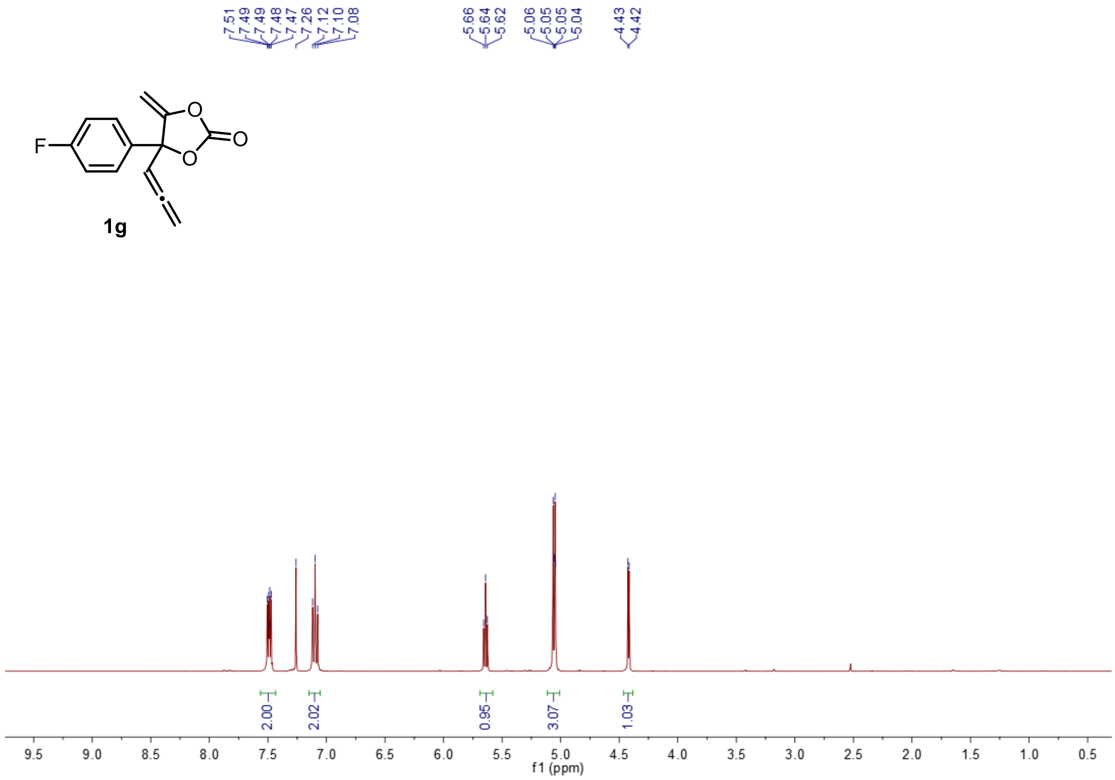

^{13}C NMR (100 MHz, $CDCl_3$)

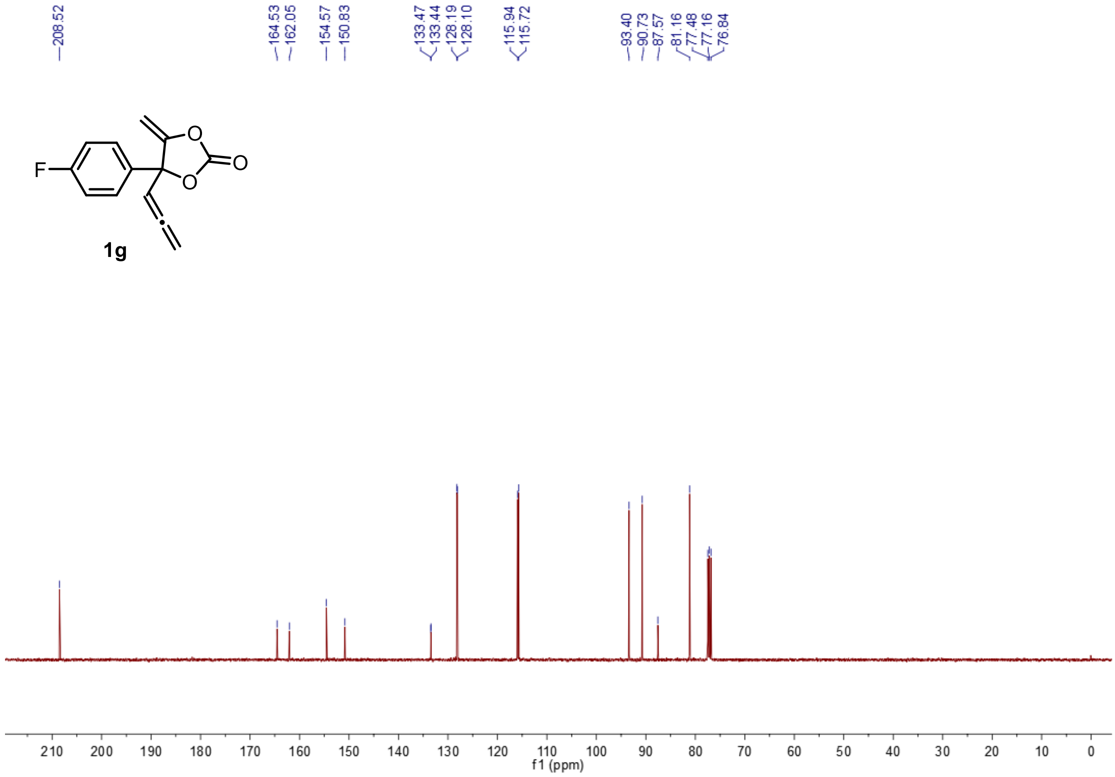

^{19}F NMR (376 MHz, $CDCl_3$)

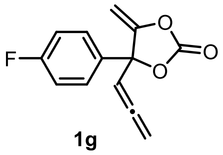

1g

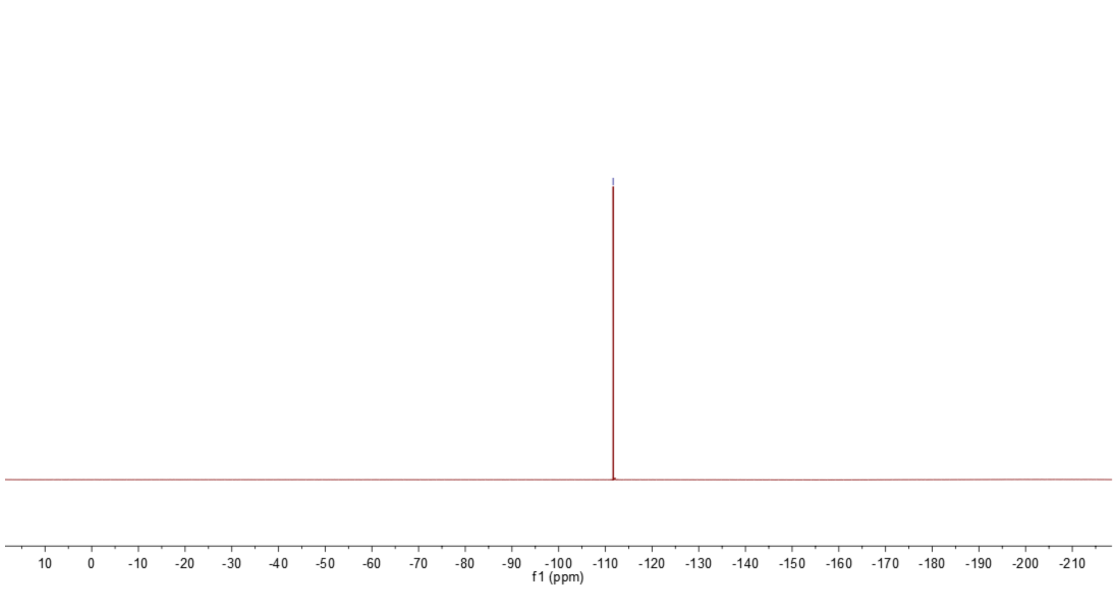

^{1}H NMR (400 MHz, $CDCl_3$)

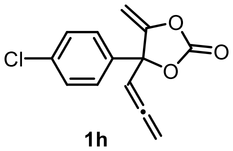

1h

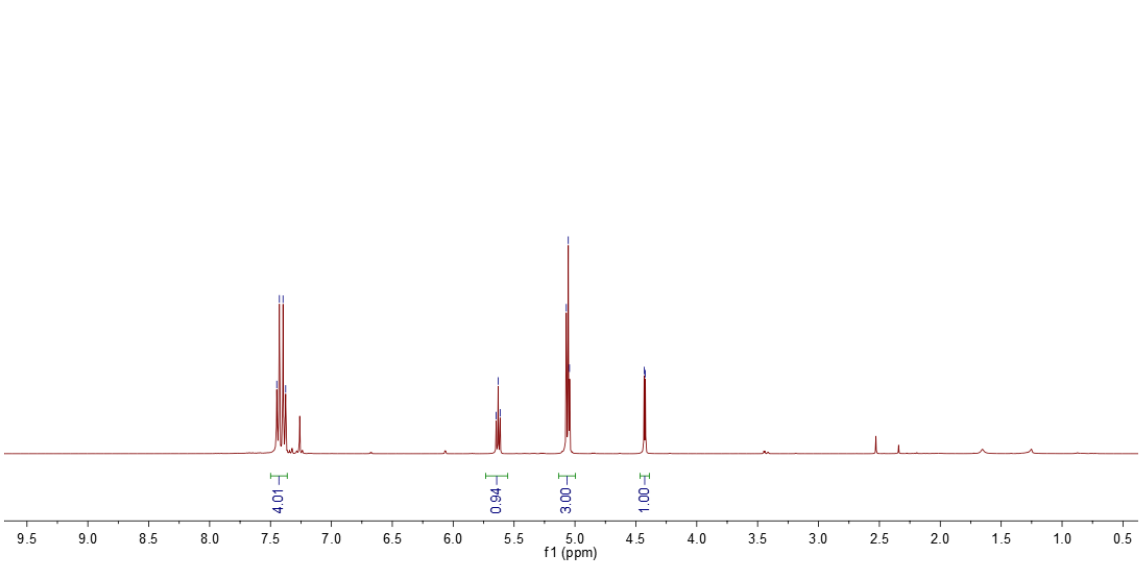

^{13}C NMR (100 MHz, $CDCl_3$)

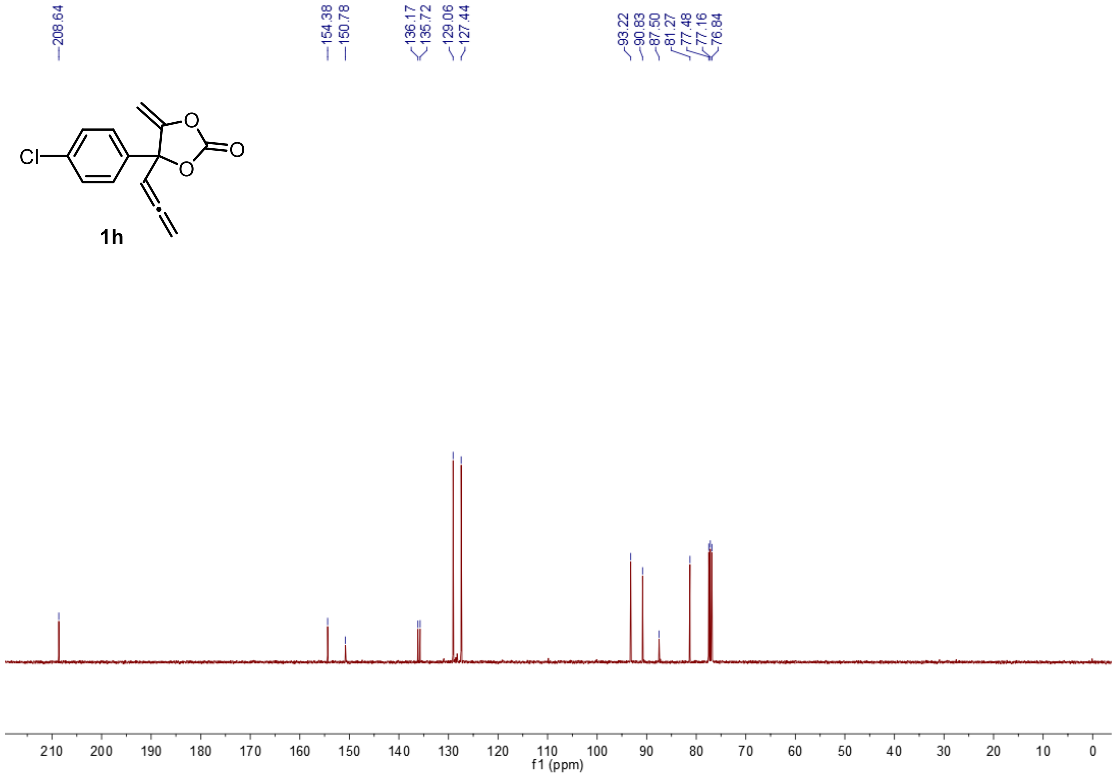

^{1}H NMR (400 MHz, $CDCl_3$)

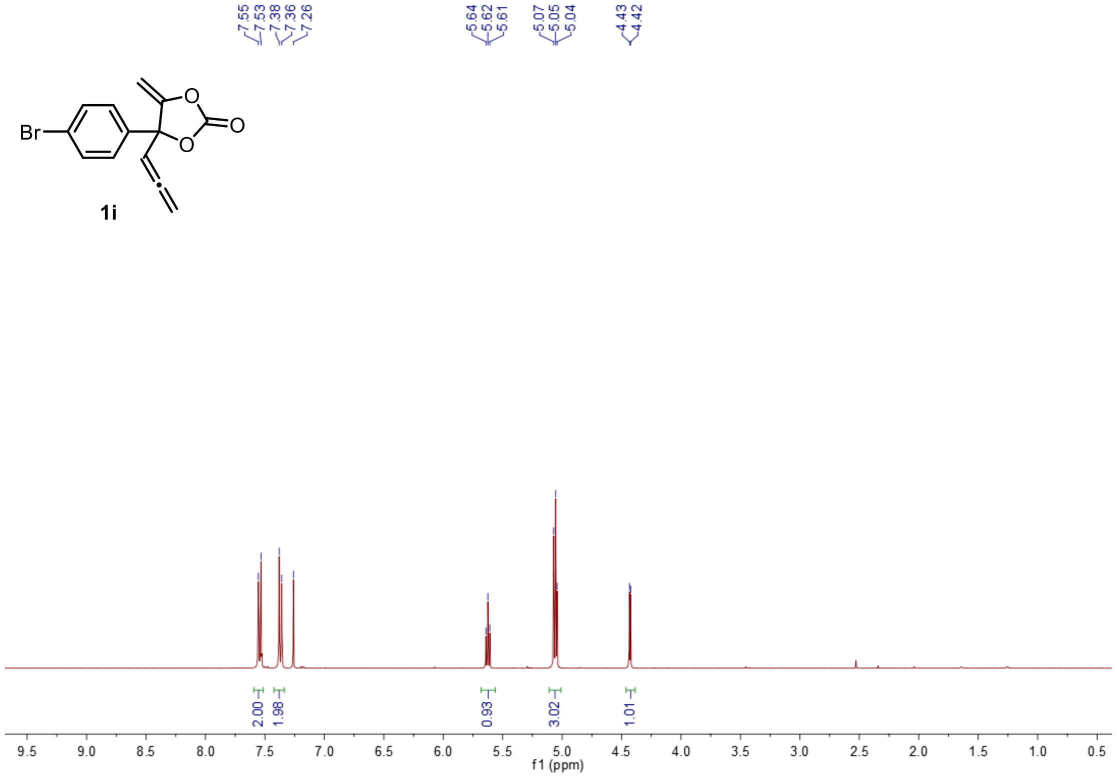

^{13}C NMR (100 MHz, $CDCl_3$)

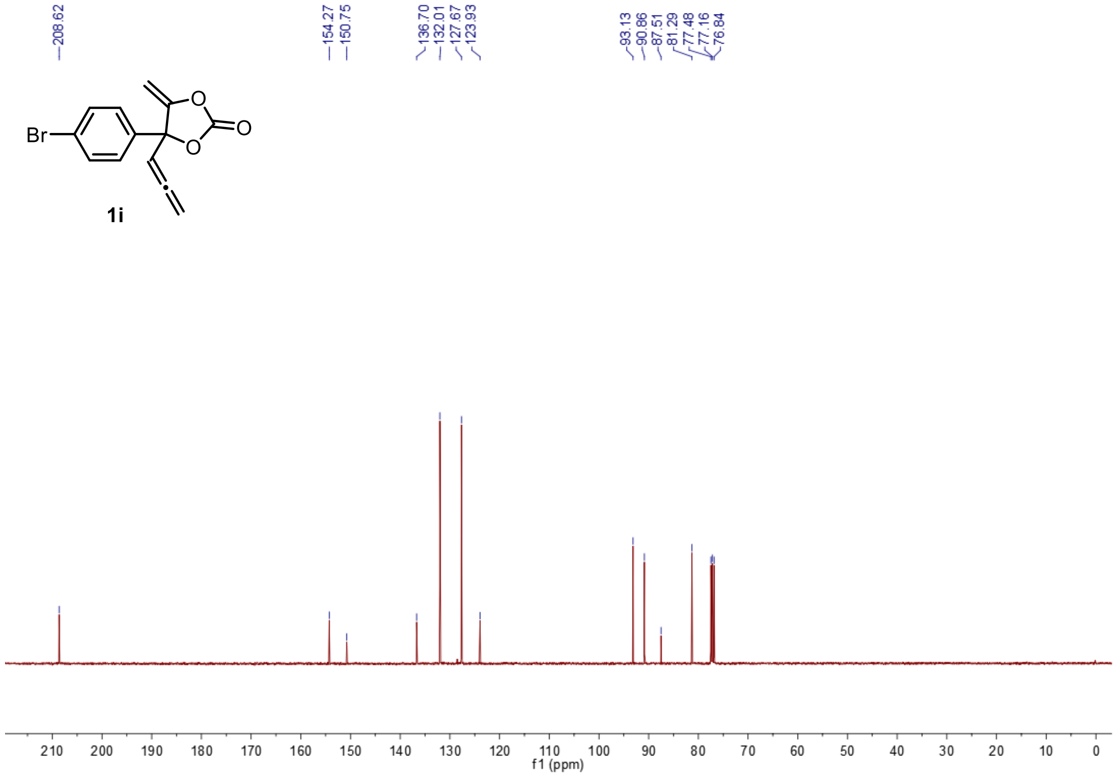

^{1}H NMR (400 MHz, $CDCl_3$)

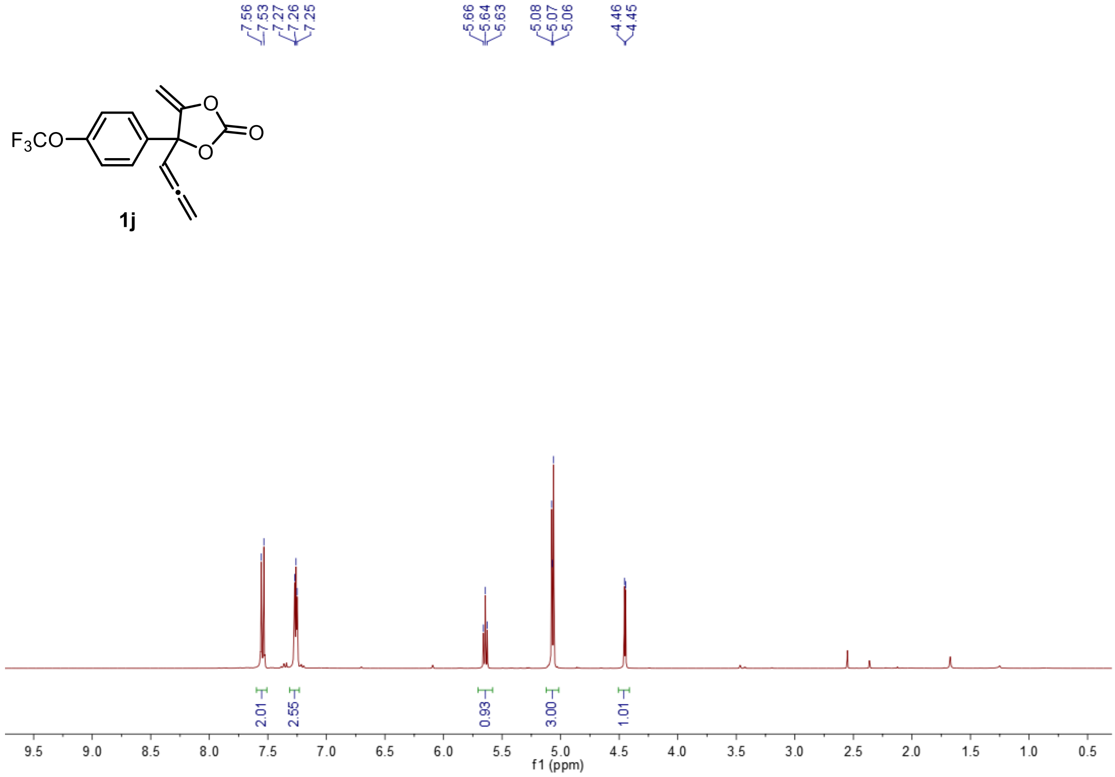

^{13}C NMR (100 MHz, $CDCl_3$)

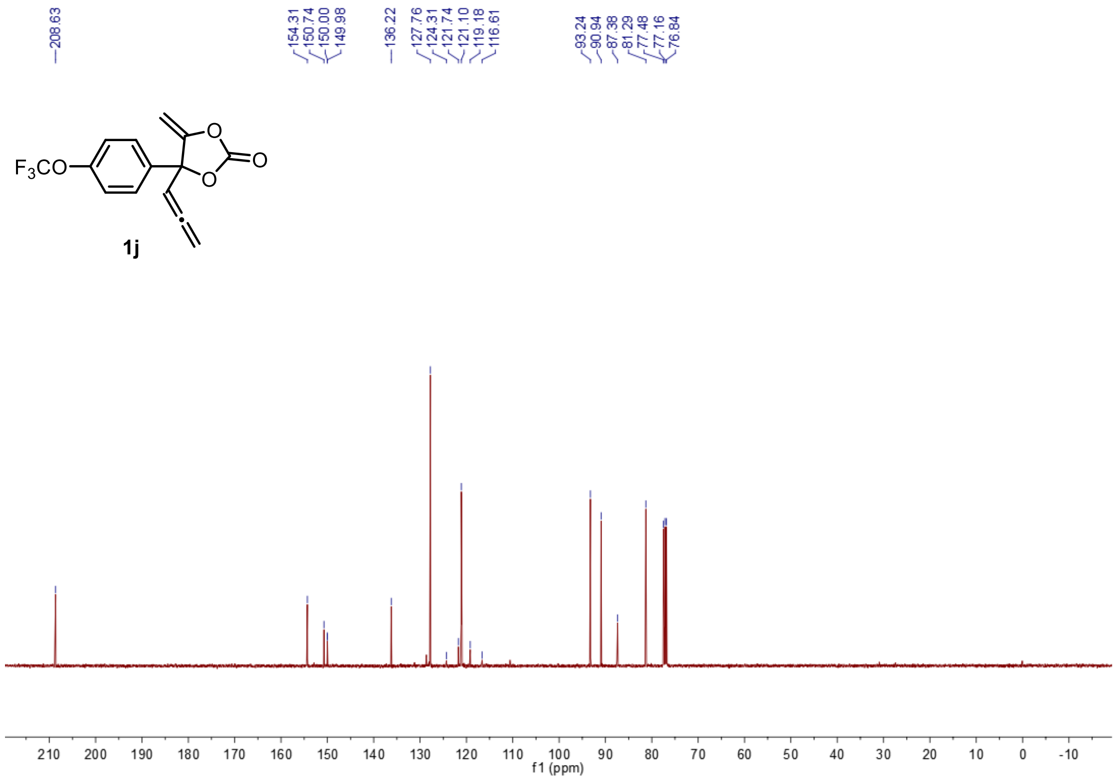

^{19}F NMR (376 MHz, $CDCl_3$)

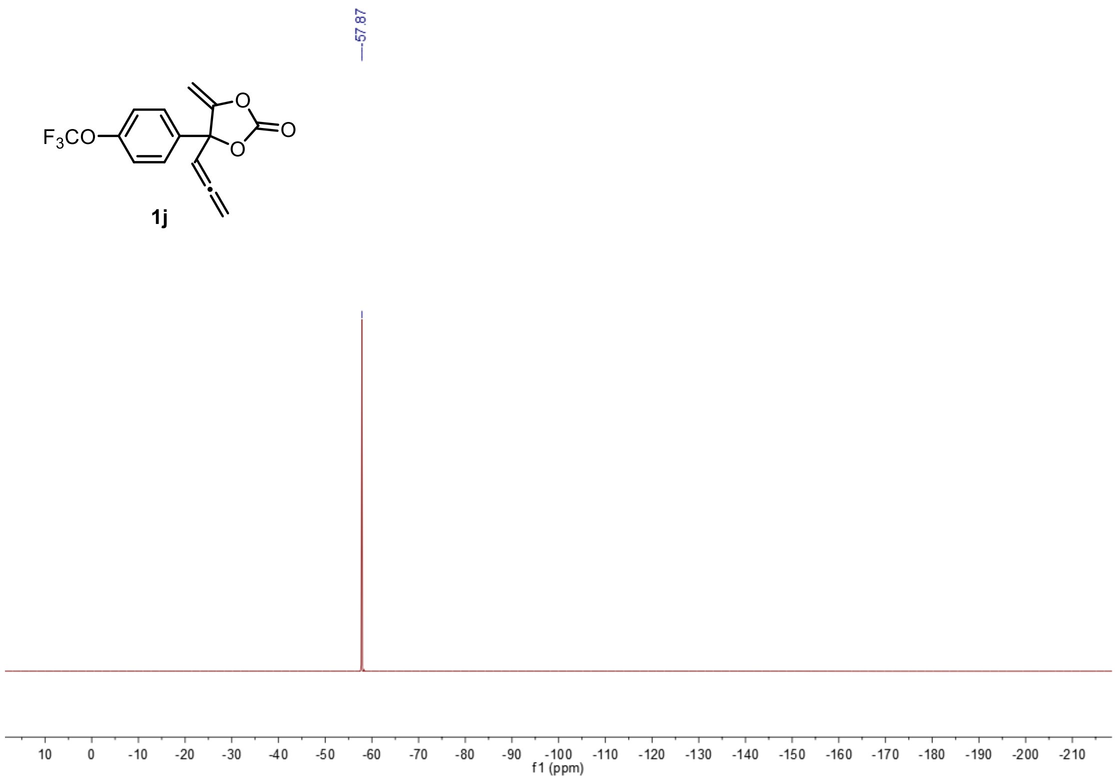

^{1}H NMR (400 MHz, $CDCl_3$)

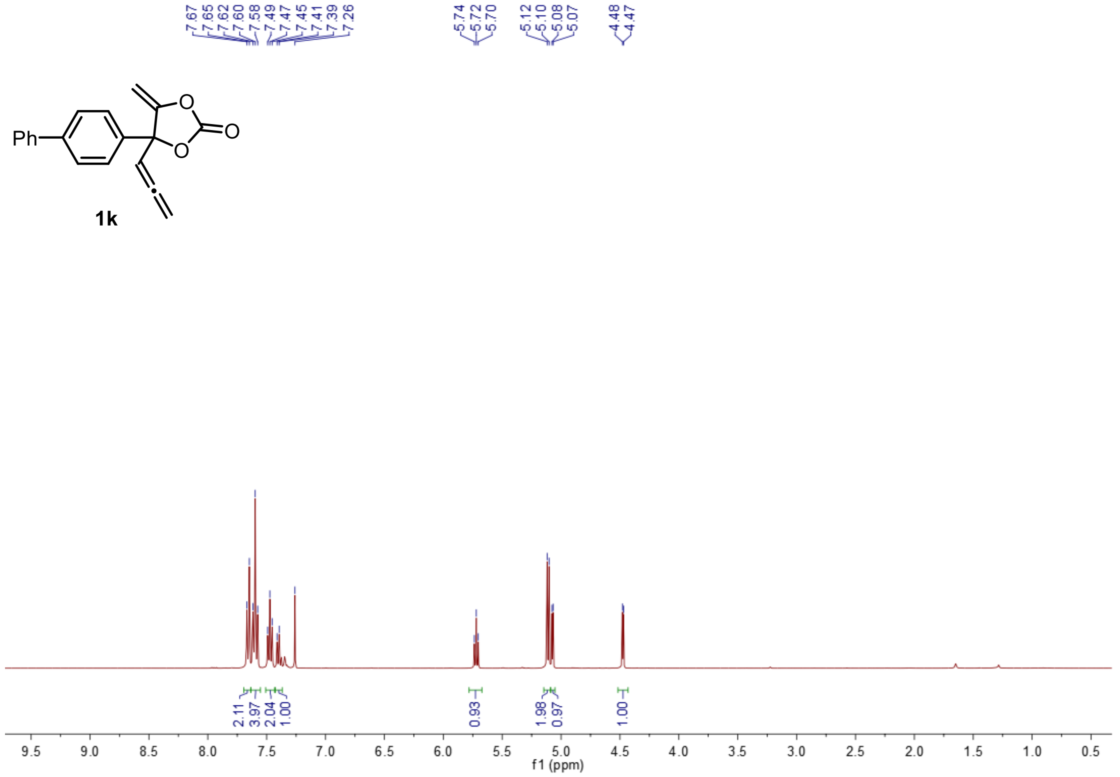

^{13}C NMR (100 MHz, $CDCl_3$)

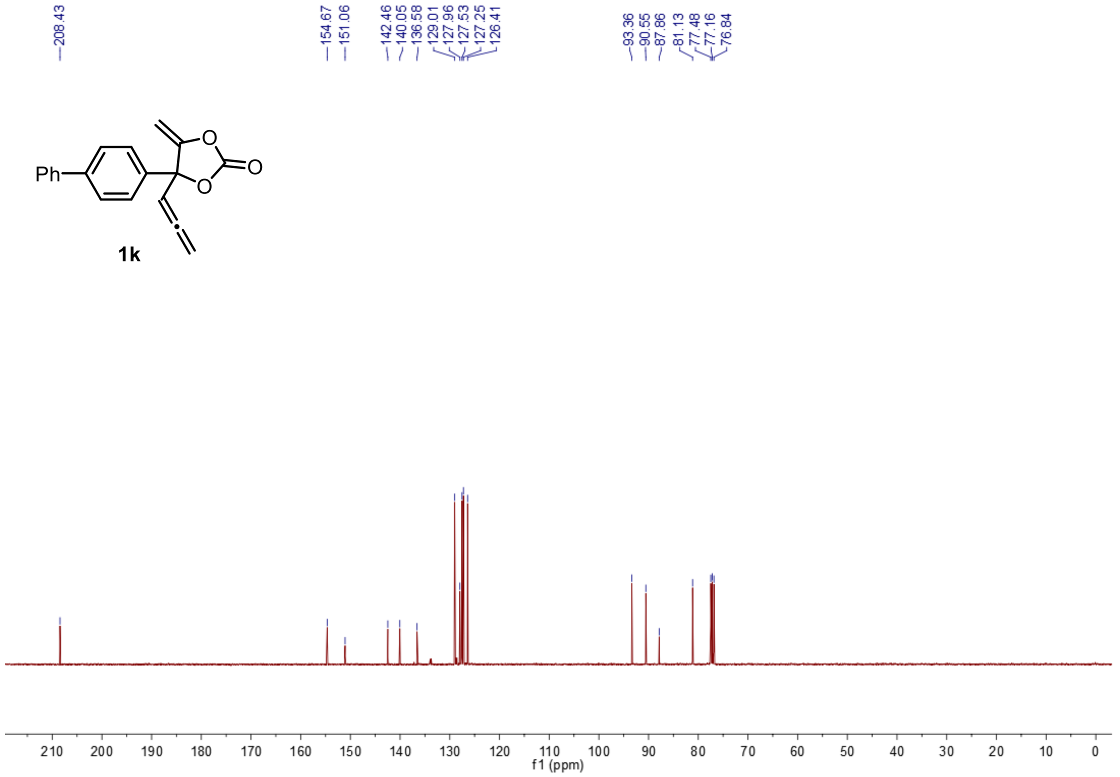

^{1}H NMR (400 MHz, $CDCl_3$)

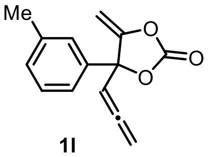

1l

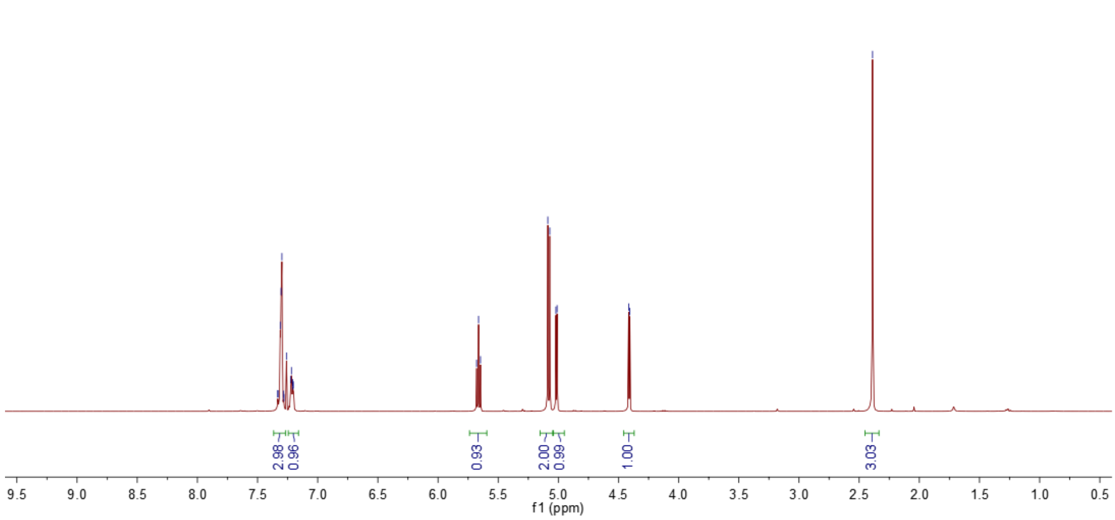

^{13}C NMR (100 MHz, $CDCl_3$)

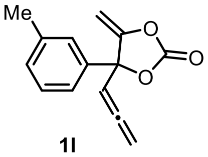

1l

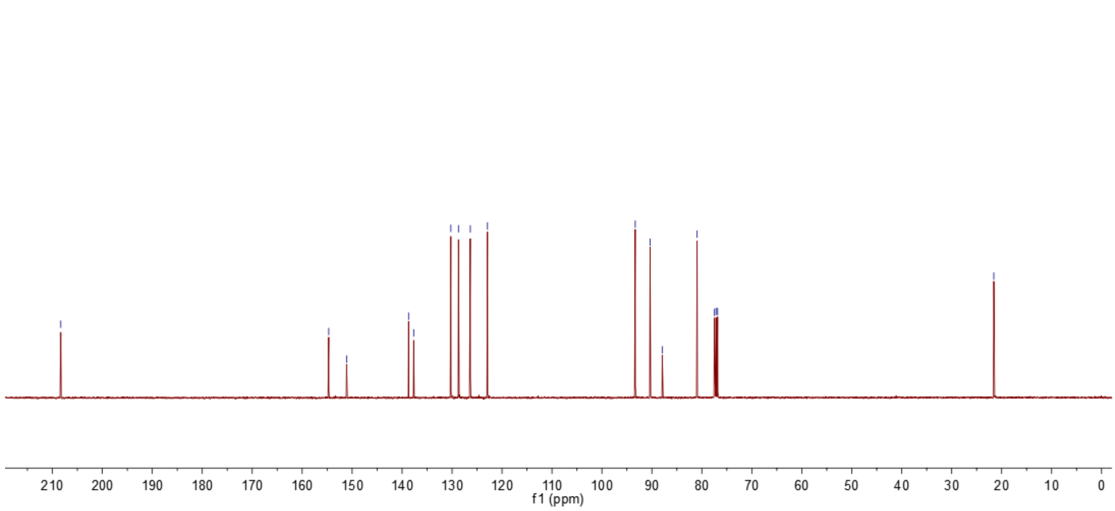

^{1}H NMR (400 MHz, $CDCl_3$)

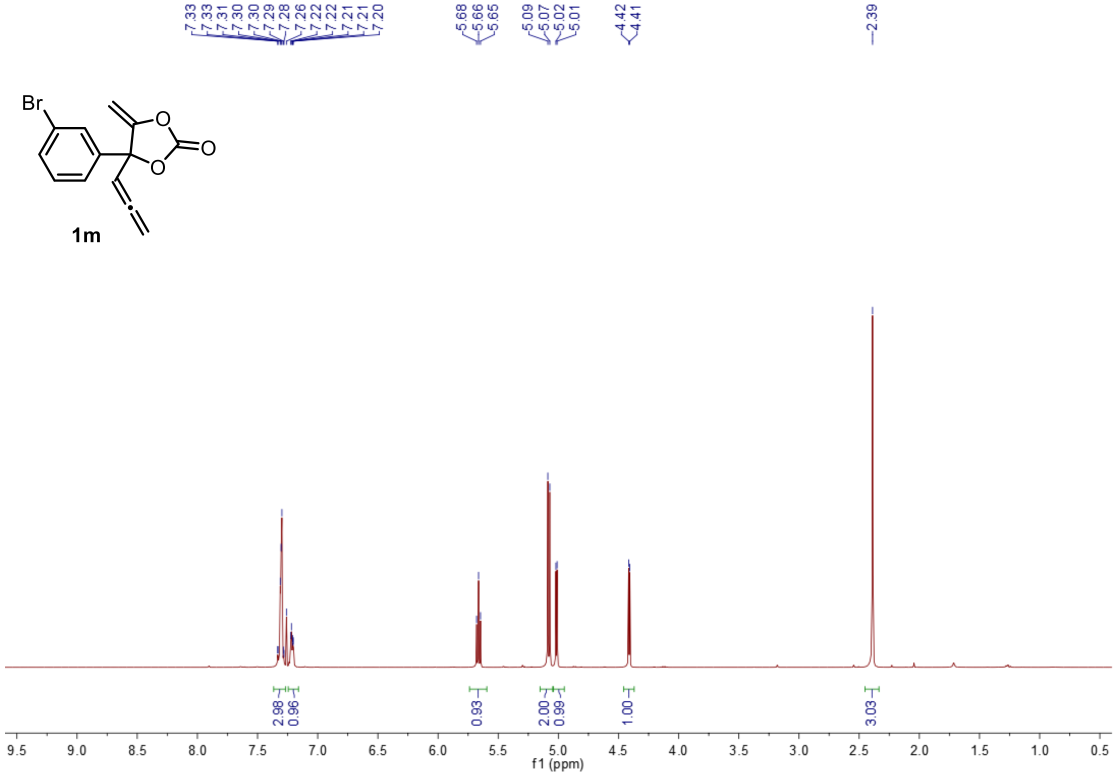

^{13}C NMR (100 MHz, $CDCl_3$)

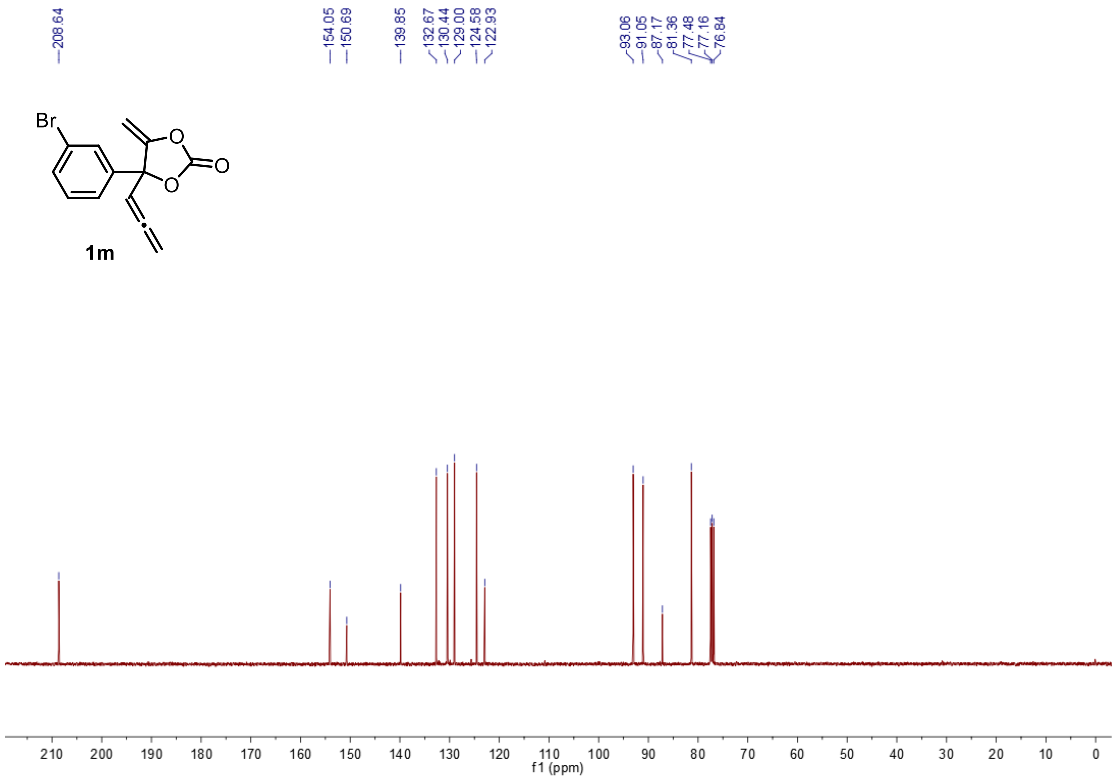

^{1}H NMR (400 MHz, $CDCl_3$)

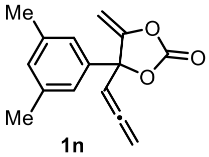

1n

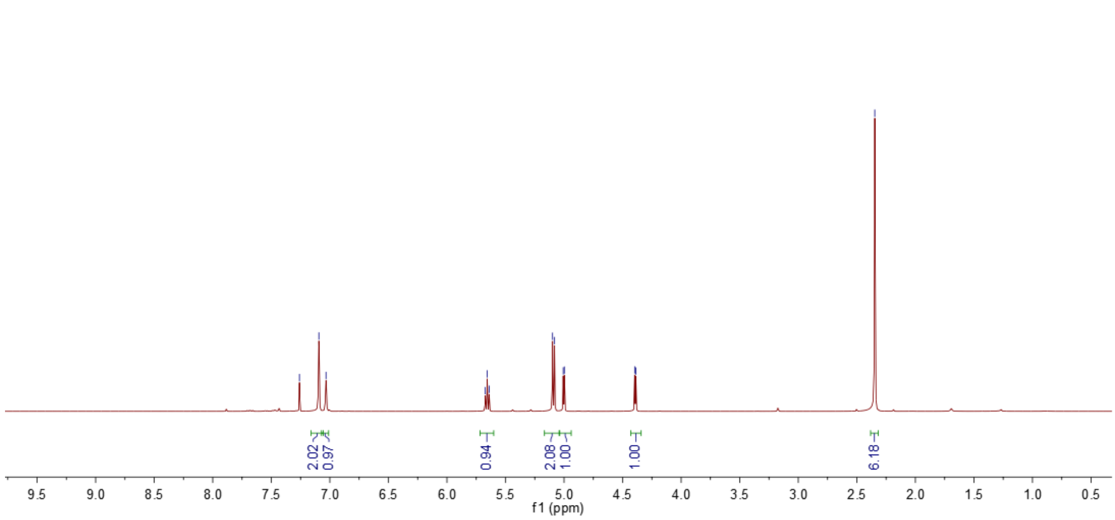

^{13}C NMR (100 MHz, $CDCl_3$)

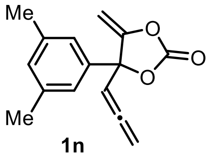

1n

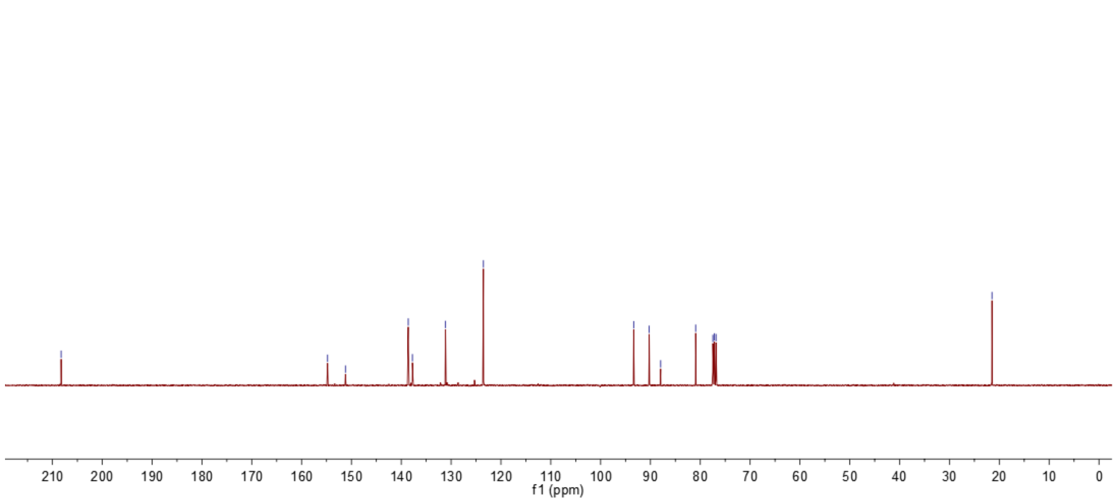

^{1}H NMR (400 MHz, $CDCl_3$)

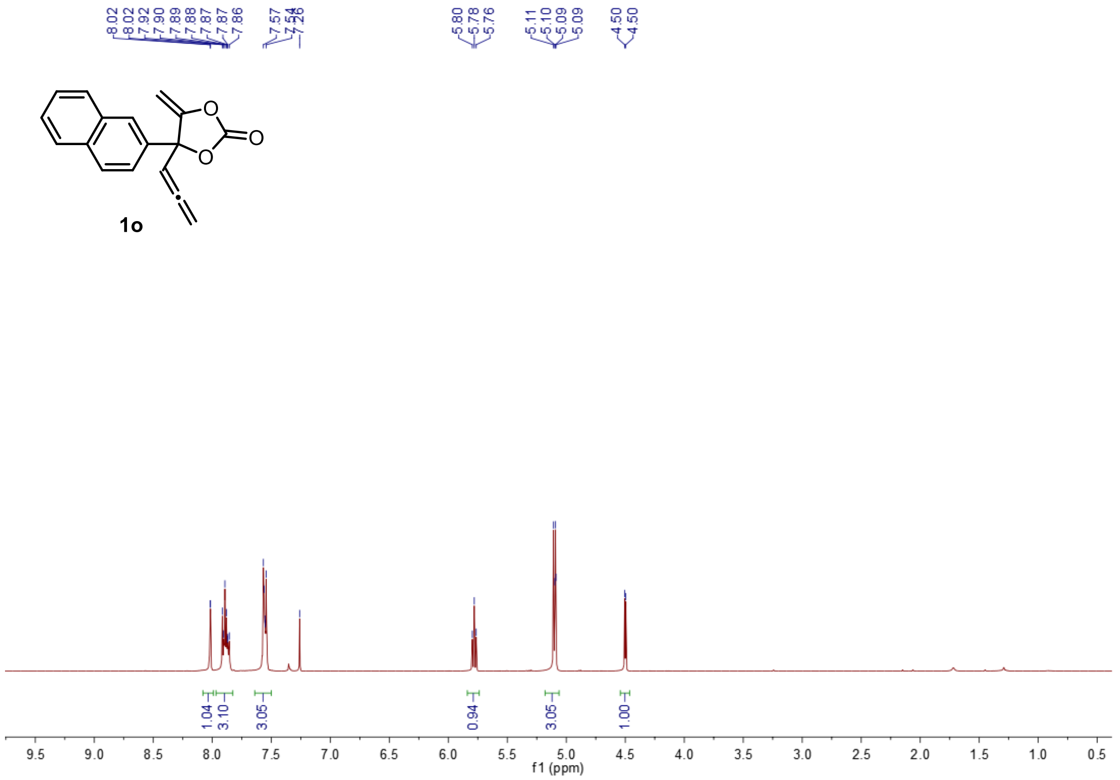

^{13}C NMR (100 MHz, $CDCl_3$)

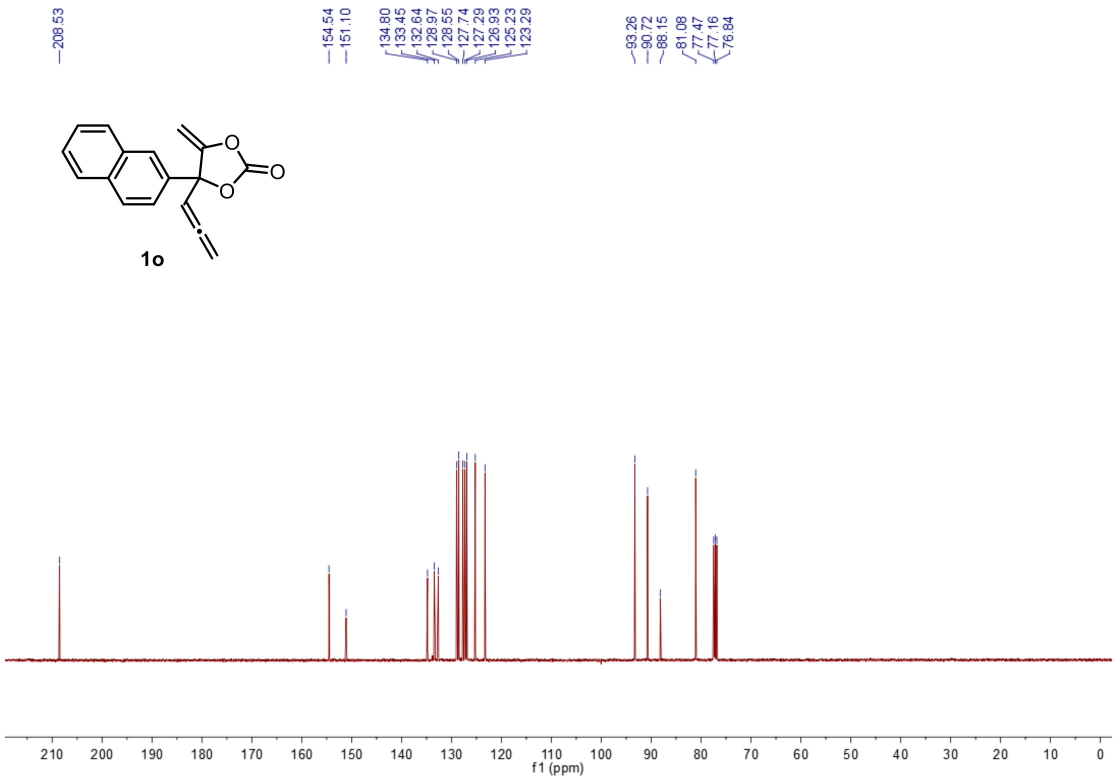

^{1}H NMR (400 MHz, $CDCl_3$)

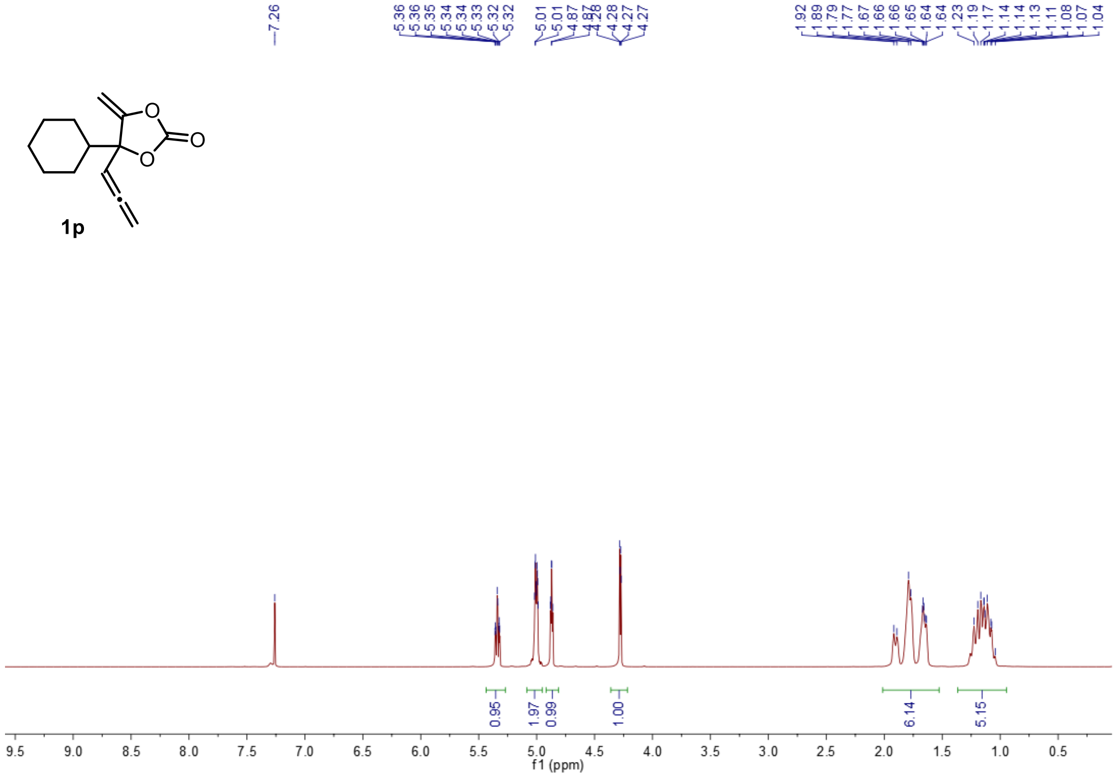

^{13}C NMR (100 MHz, $CDCl_3$)

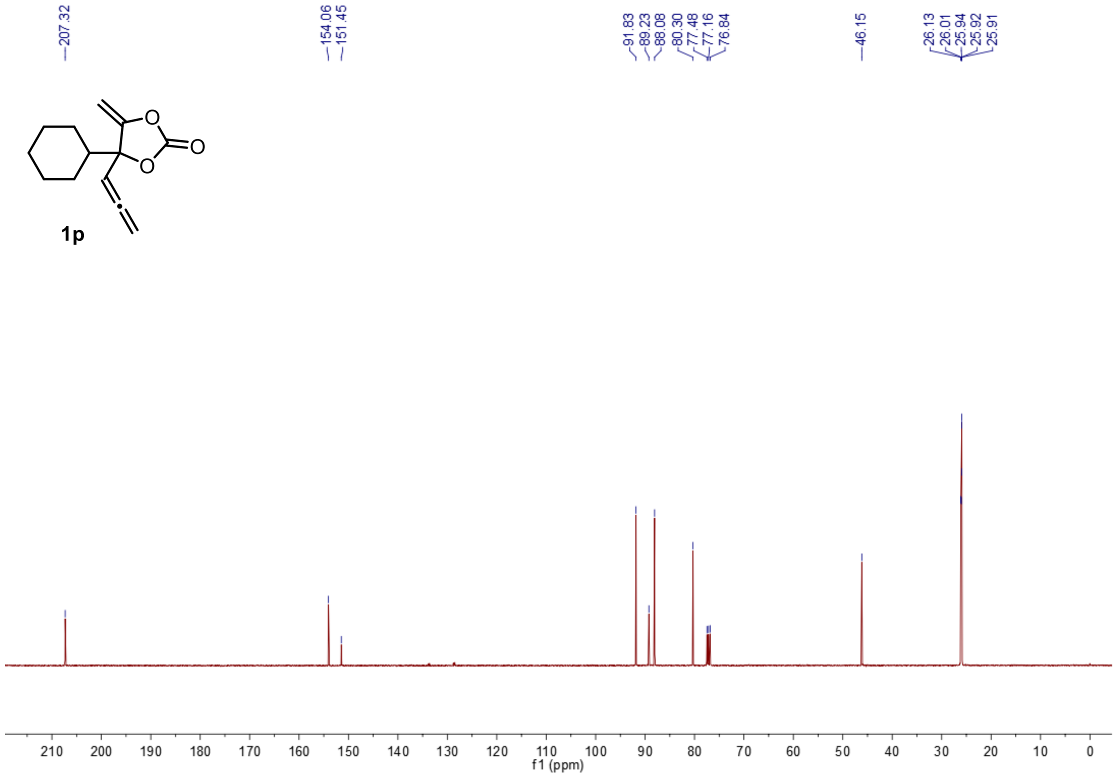

^{1}H NMR (400 MHz, $CDCl_3$)

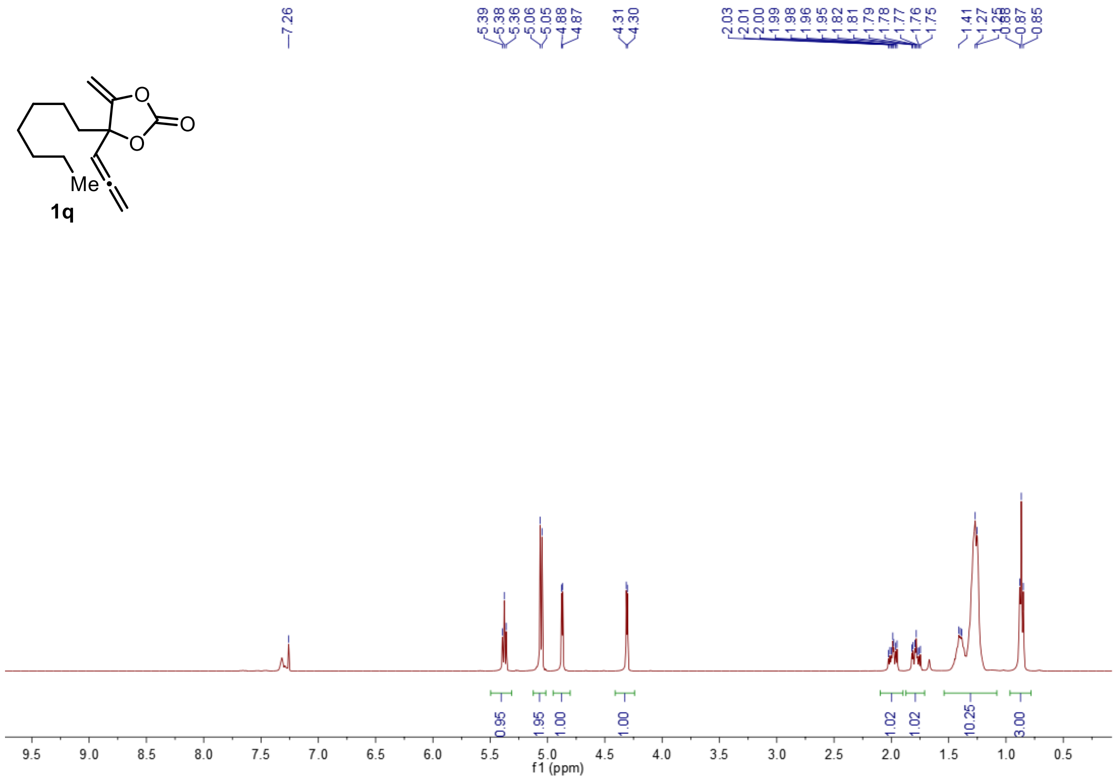

^{13}C NMR (100 MHz, $CDCl_3$)

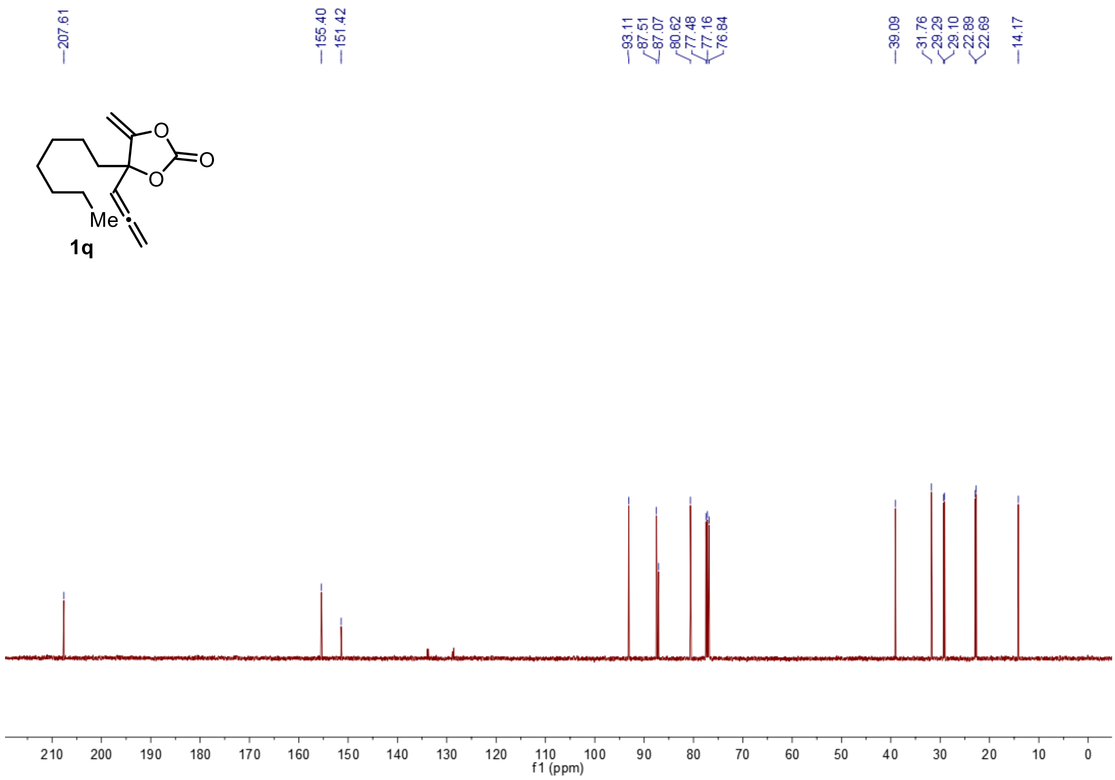

^{1}H NMR (400 MHz, $CDCl_3$)

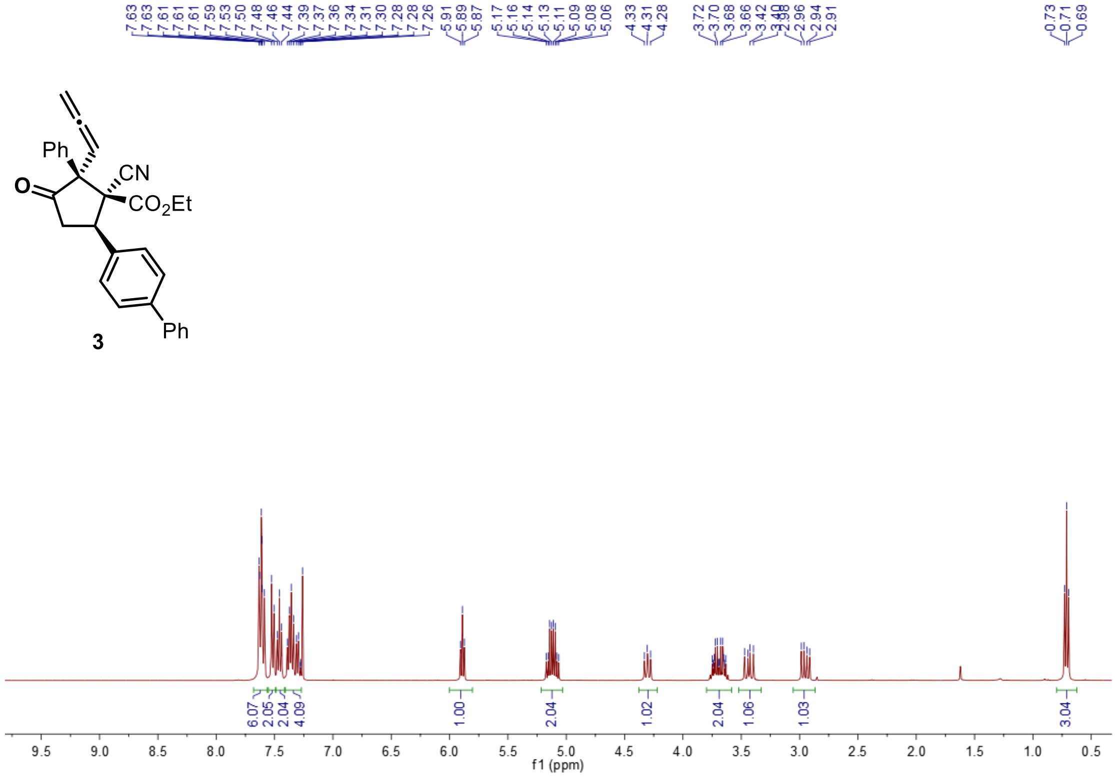

^{13}C NMR (100 MHz, $CDCl_3$)

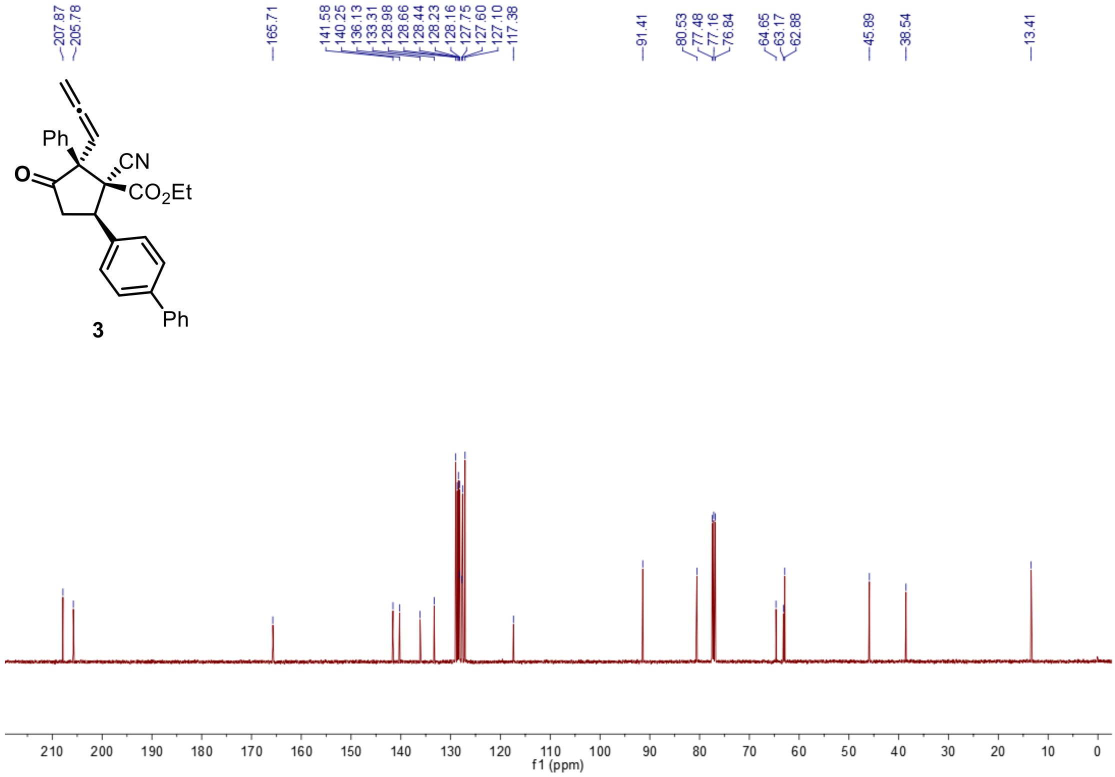

^{1}H NMR (400 MHz, $CDCl_3$)

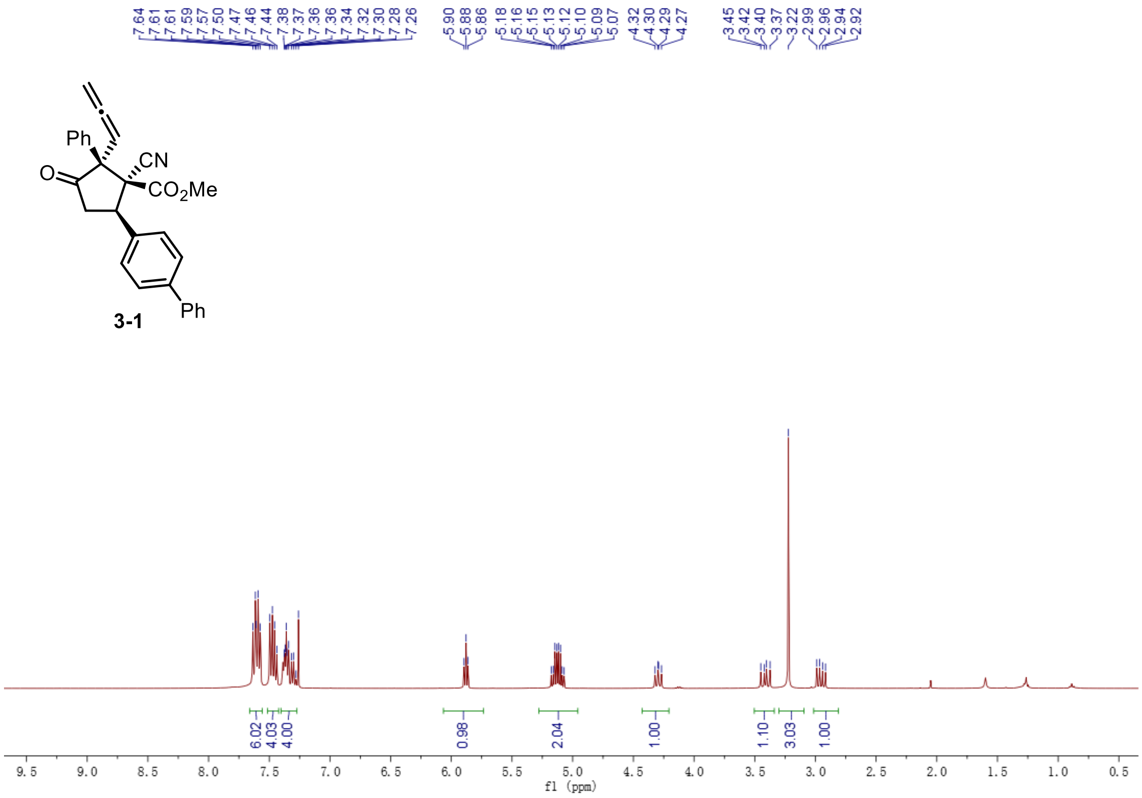

^{13}C NMR (100 MHz, $CDCl_3$)

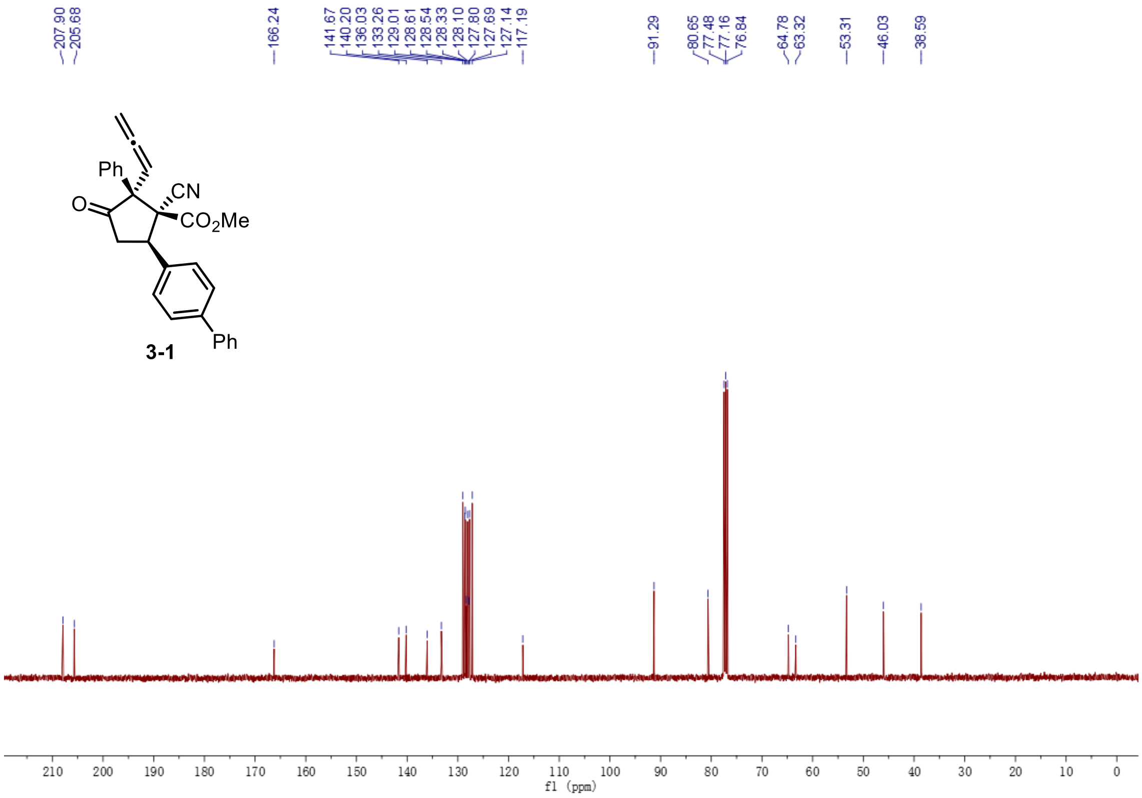

^1H NMR (400 MHz, $CDCl_3$)

3-2

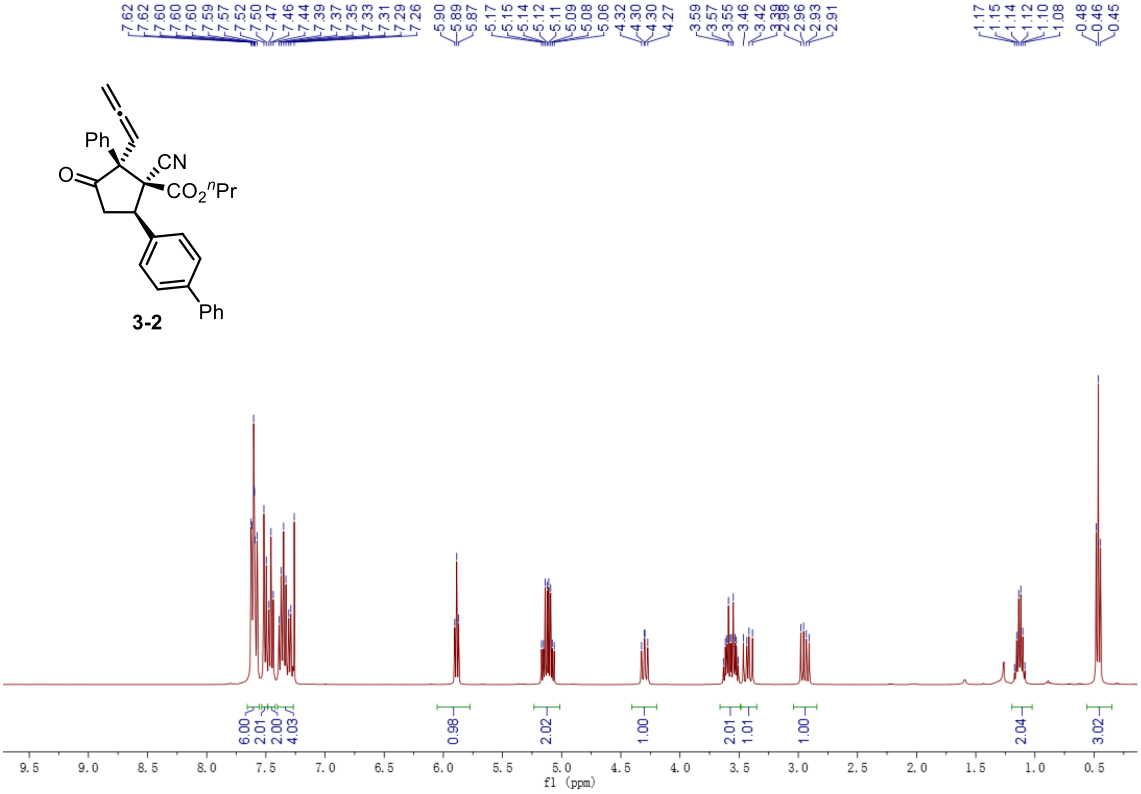

^{13}C NMR (100 MHz, $CDCl_3$)

3-2

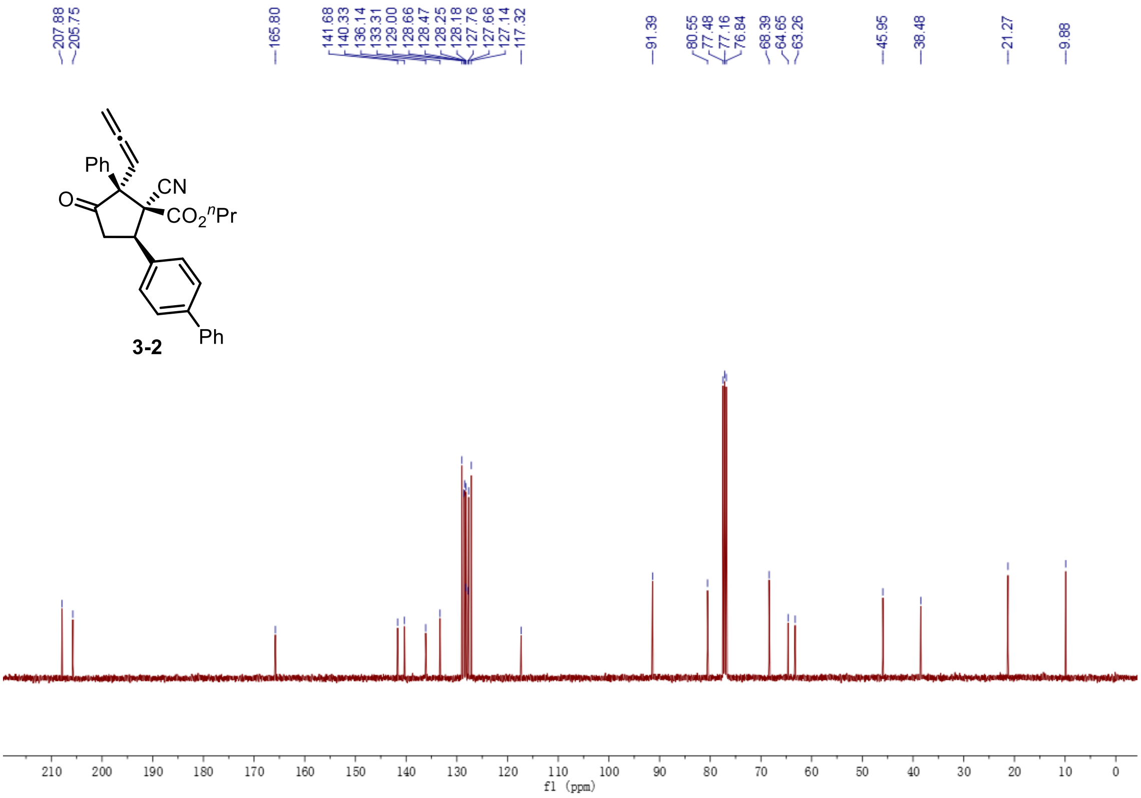

^{1}H NMR (400 MHz, $CDCl_3$)

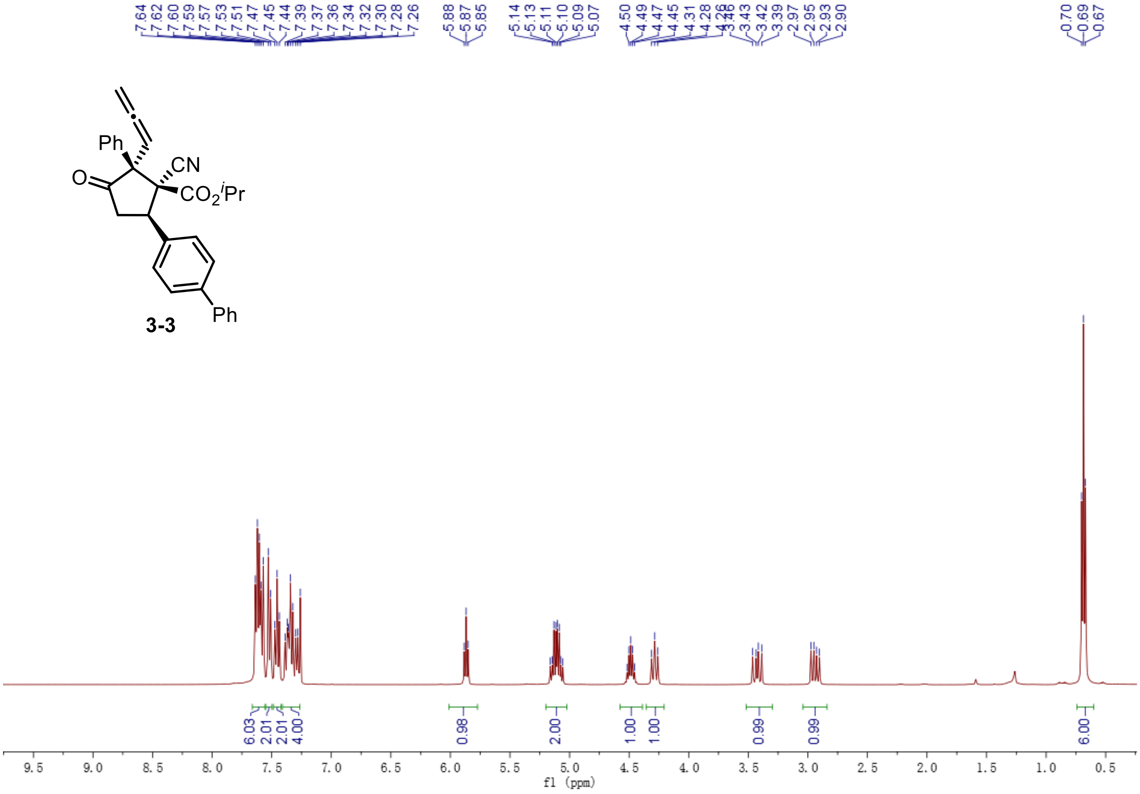

^{13}C NMR (100 MHz, $CDCl_3$)

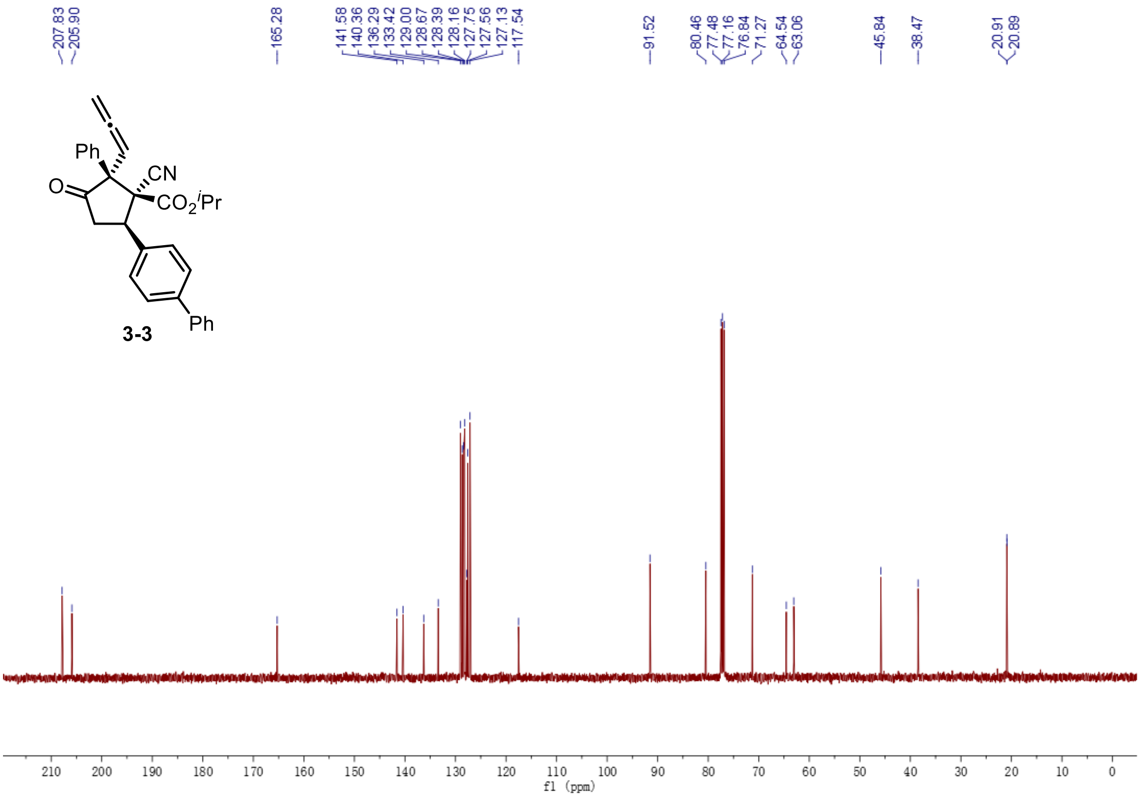

^{1}H NMR (400 MHz, $CDCl_3$)

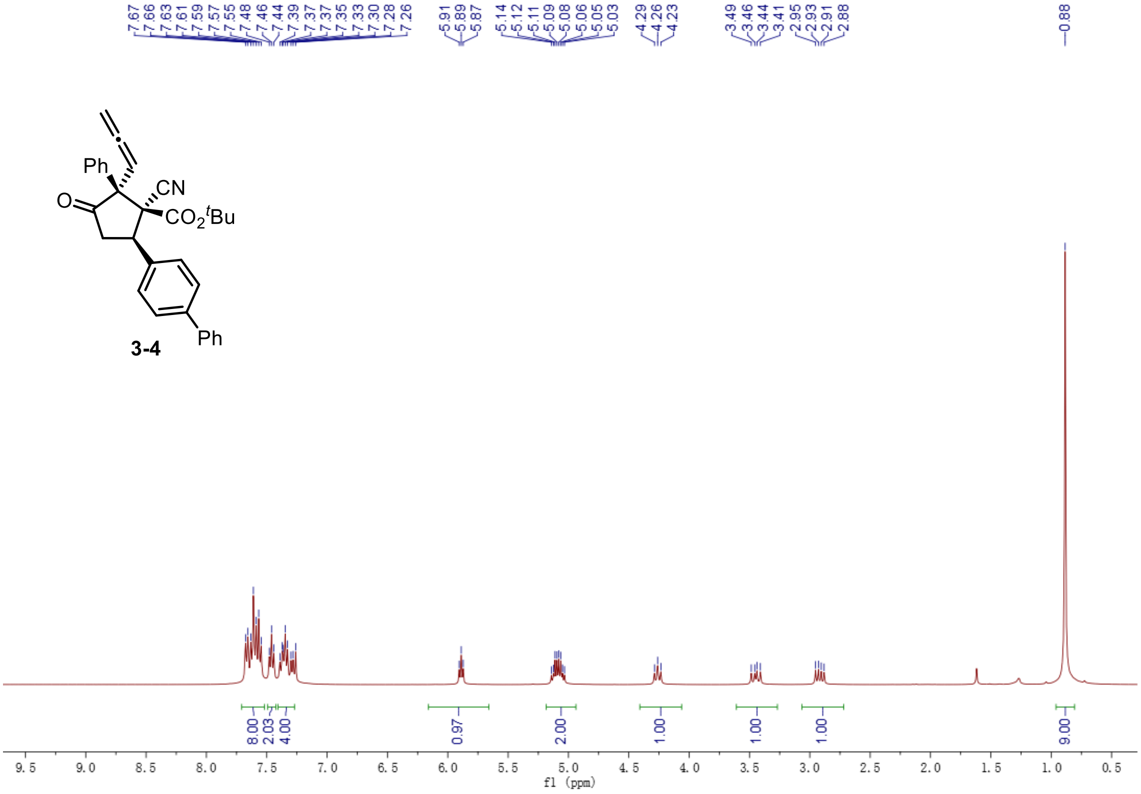

^{13}C NMR (100 MHz, $CDCl_3$)

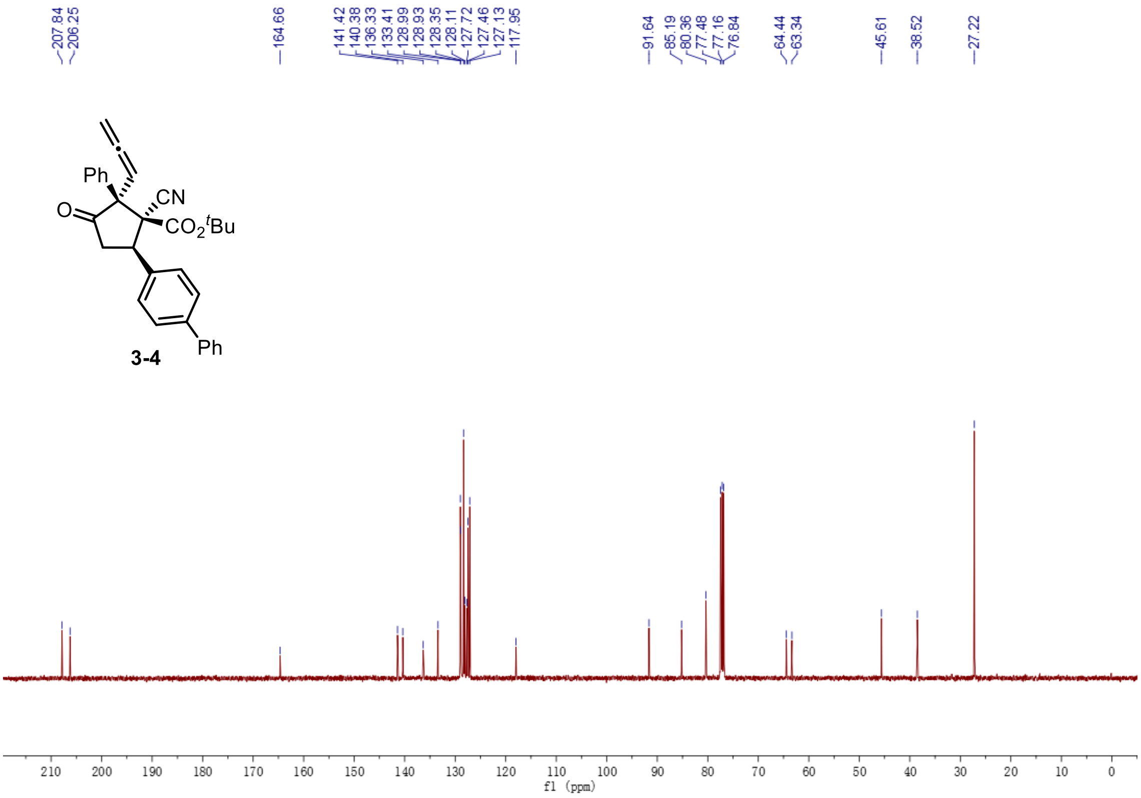

^{1}H NMR (400 MHz, $CDCl_3$)

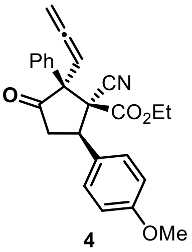

4

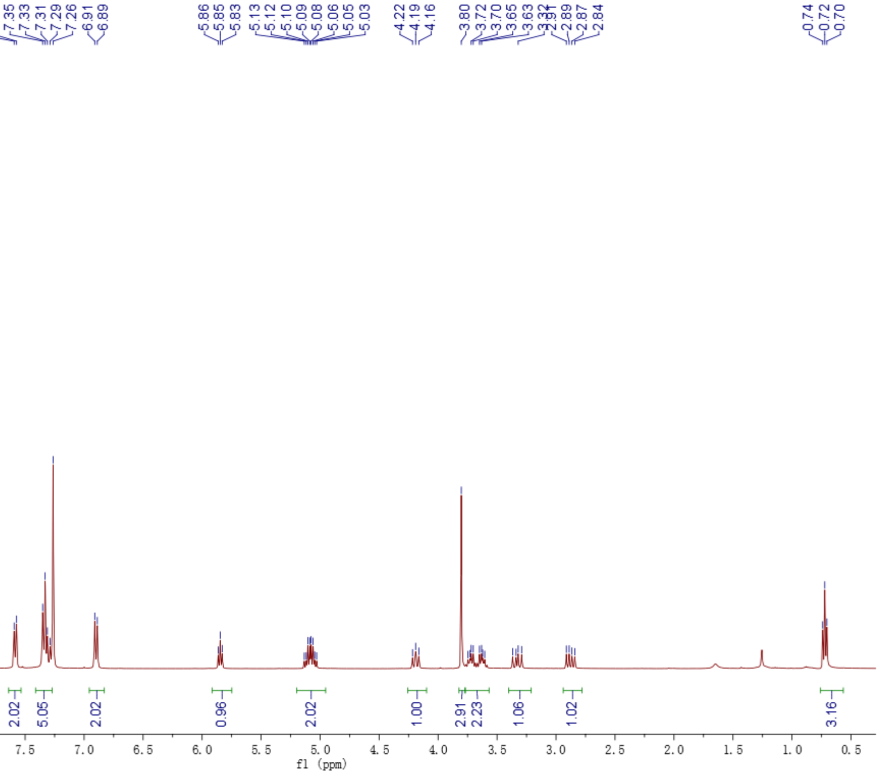

^{13}C NMR (100 MHz, $CDCl_3$)

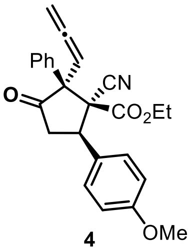

4

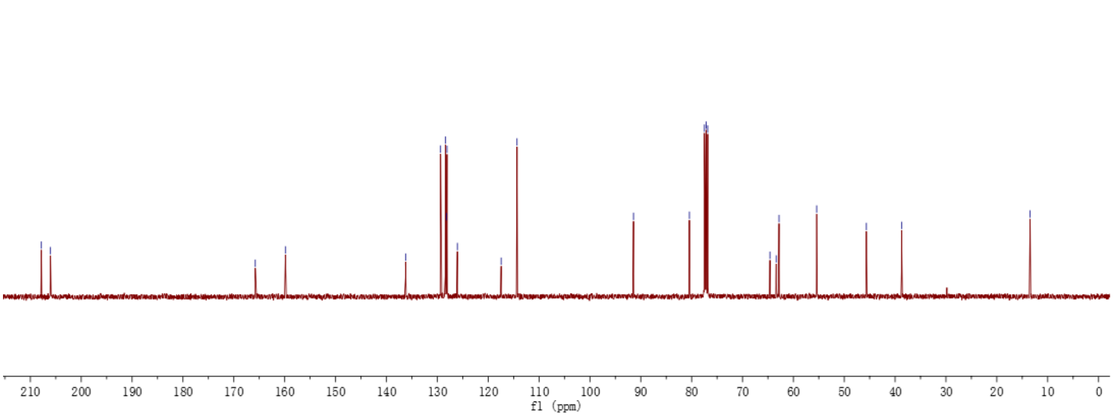

^{1}H NMR (400 MHz, $CDCl_3$)

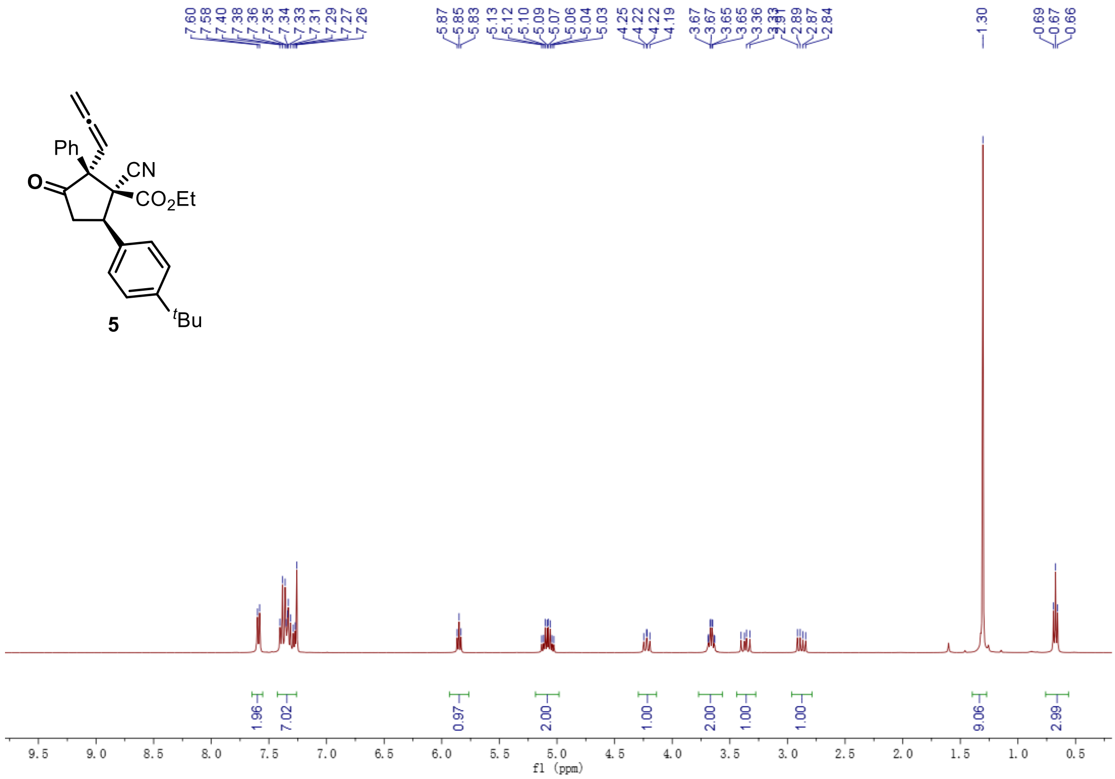

^{13}C NMR (100 MHz, $CDCl_3$)

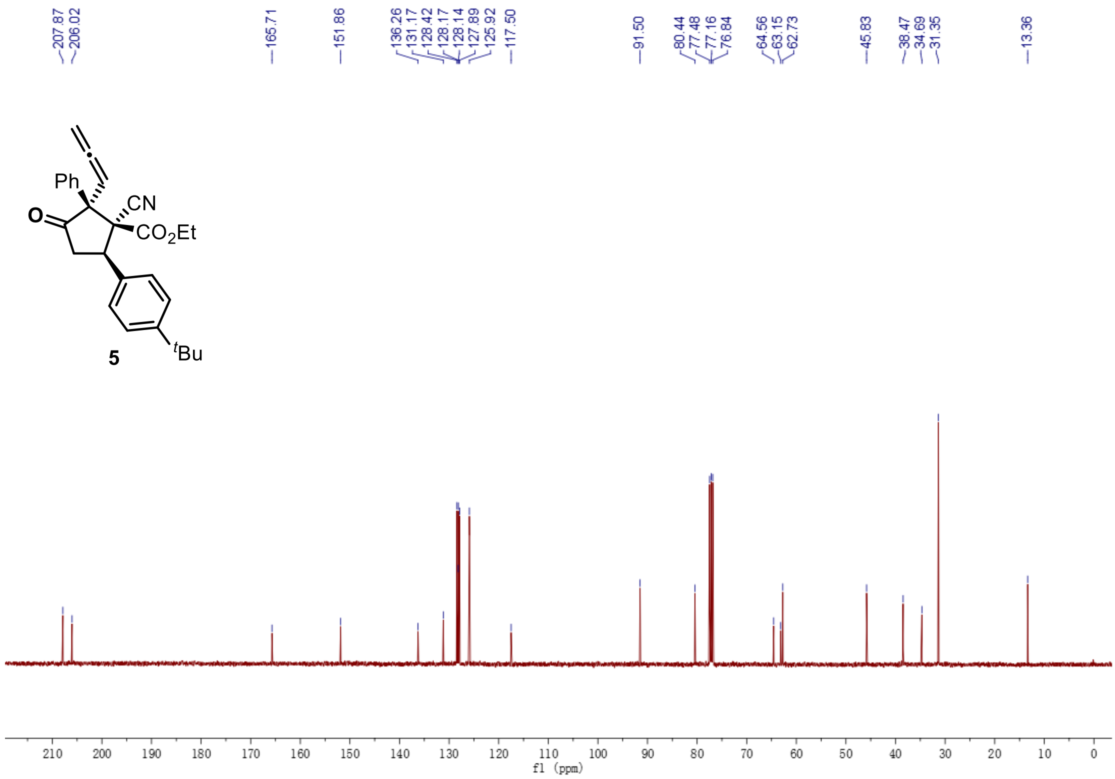

^{1}H NMR (400 MHz, $CDCl_3$)

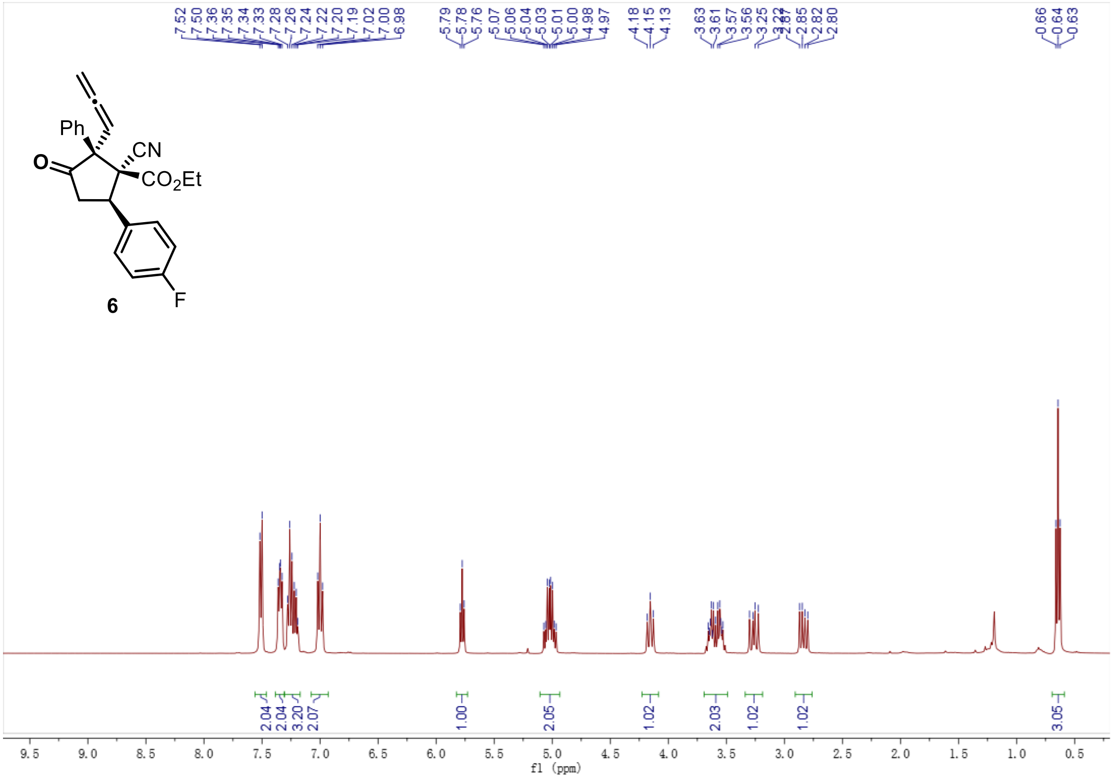

^{13}C NMR (100 MHz, $CDCl_3$)

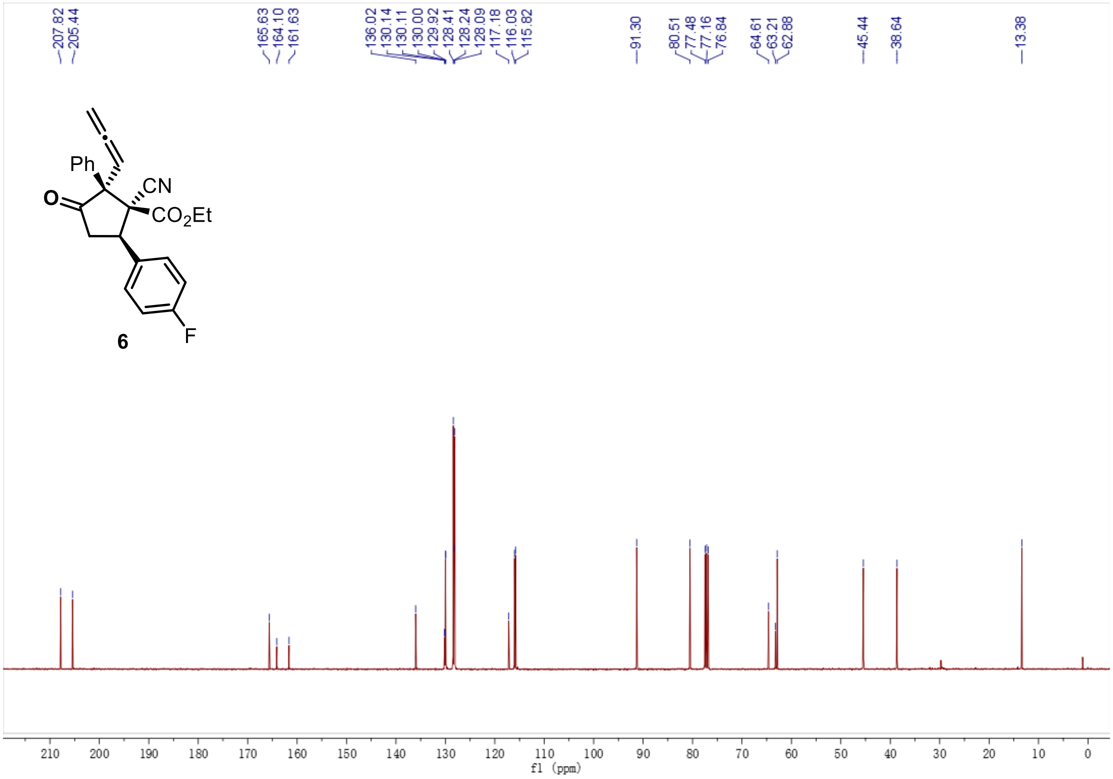

^{19}F NMR (376 MHz, $CDCl_3$)

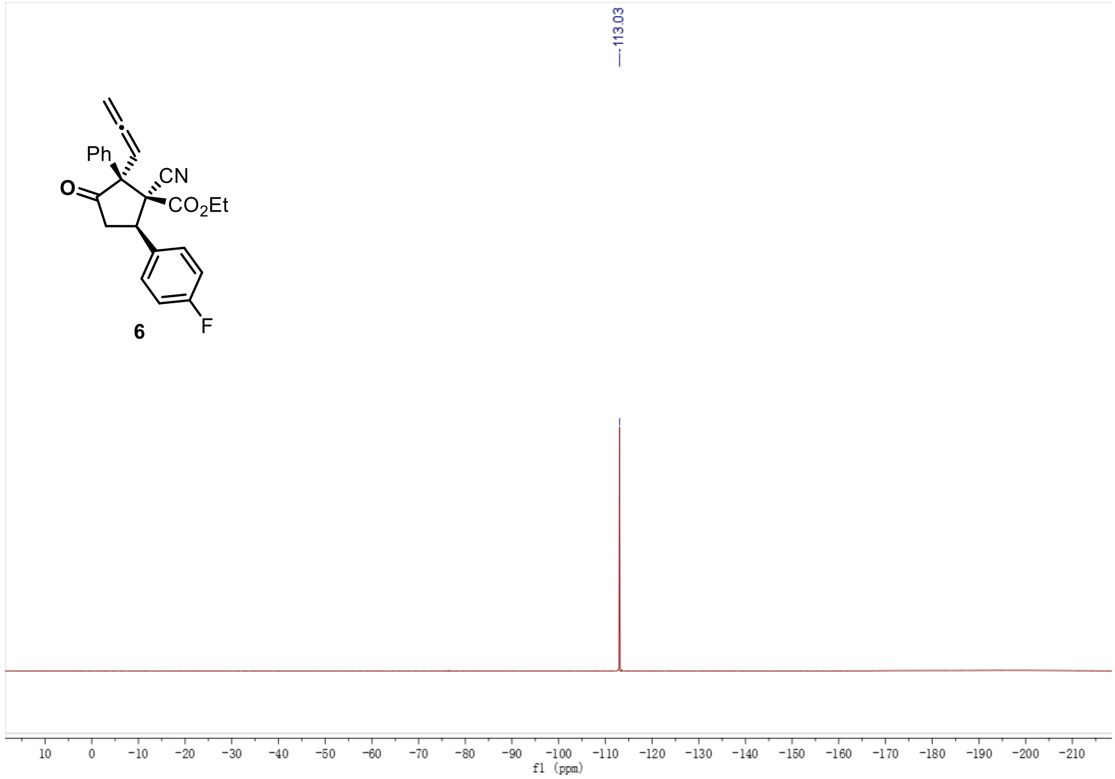

^{1}H NMR (400 MHz, $CDCl_3$)

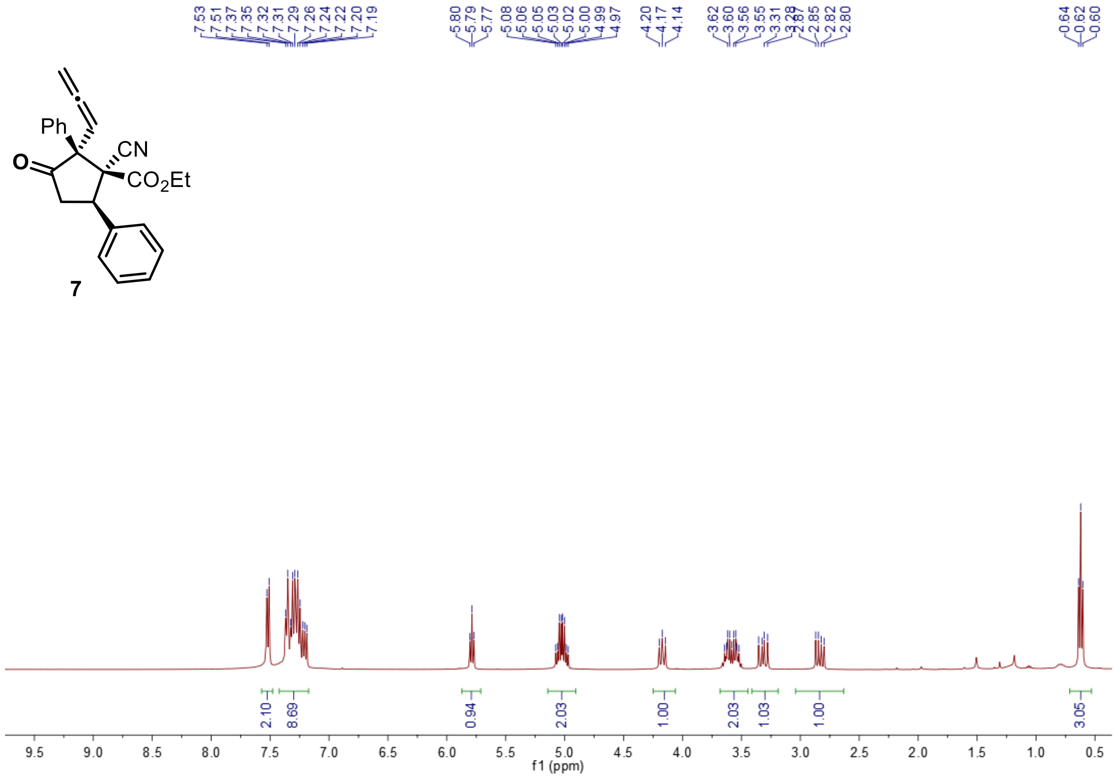

^{13}C NMR (100 MHz, $CDCl_3$)

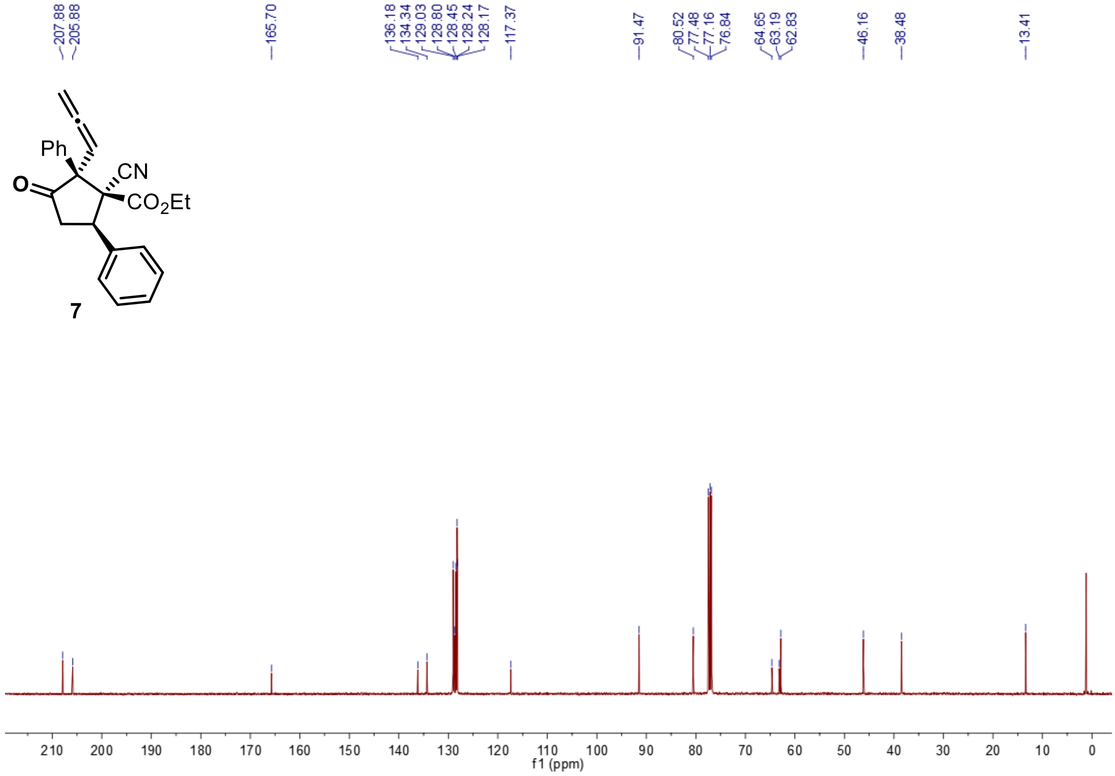

^{1}H NMR (400 MHz, $CDCl_3$)

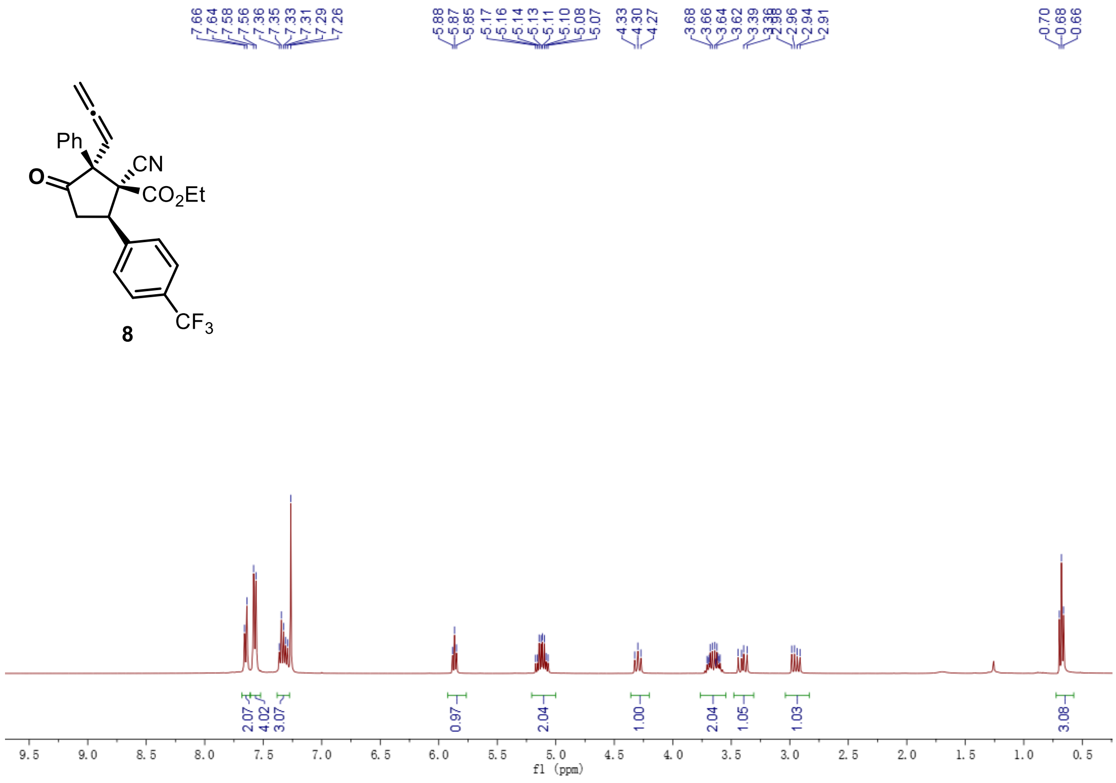

^{13}C NMR (100 MHz, $CDCl_3$)

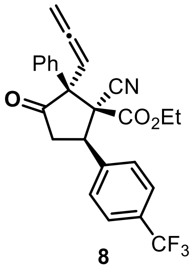

8

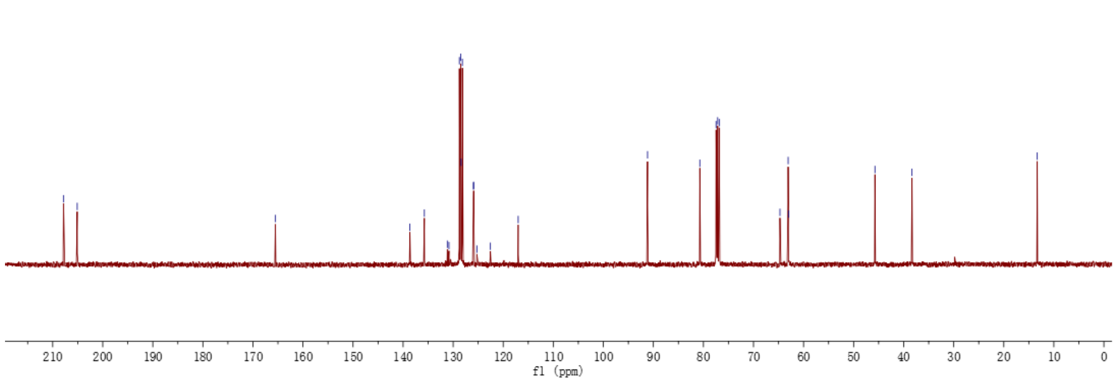

^{19}F NMR (376 MHz, $CDCl_3$)

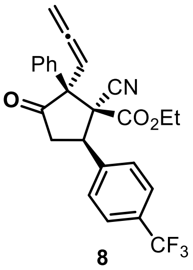

8

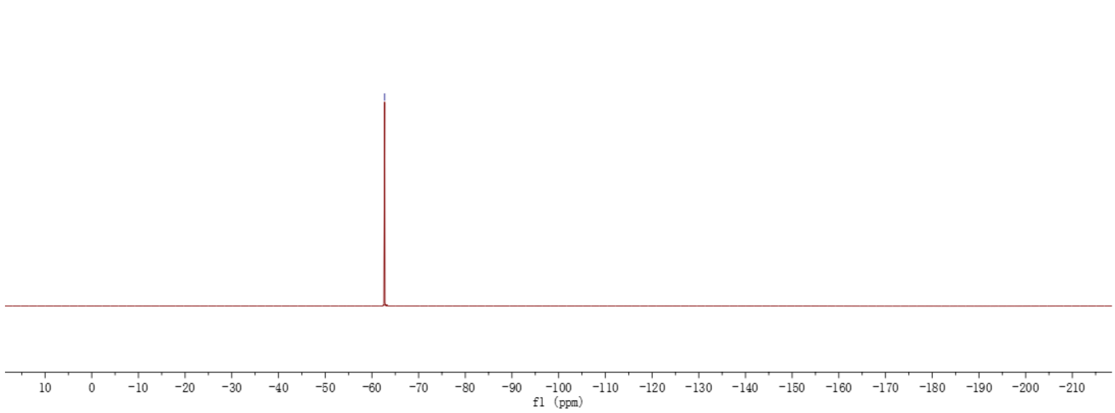

^{1}H NMR (400 MHz, $CDCl_3$)

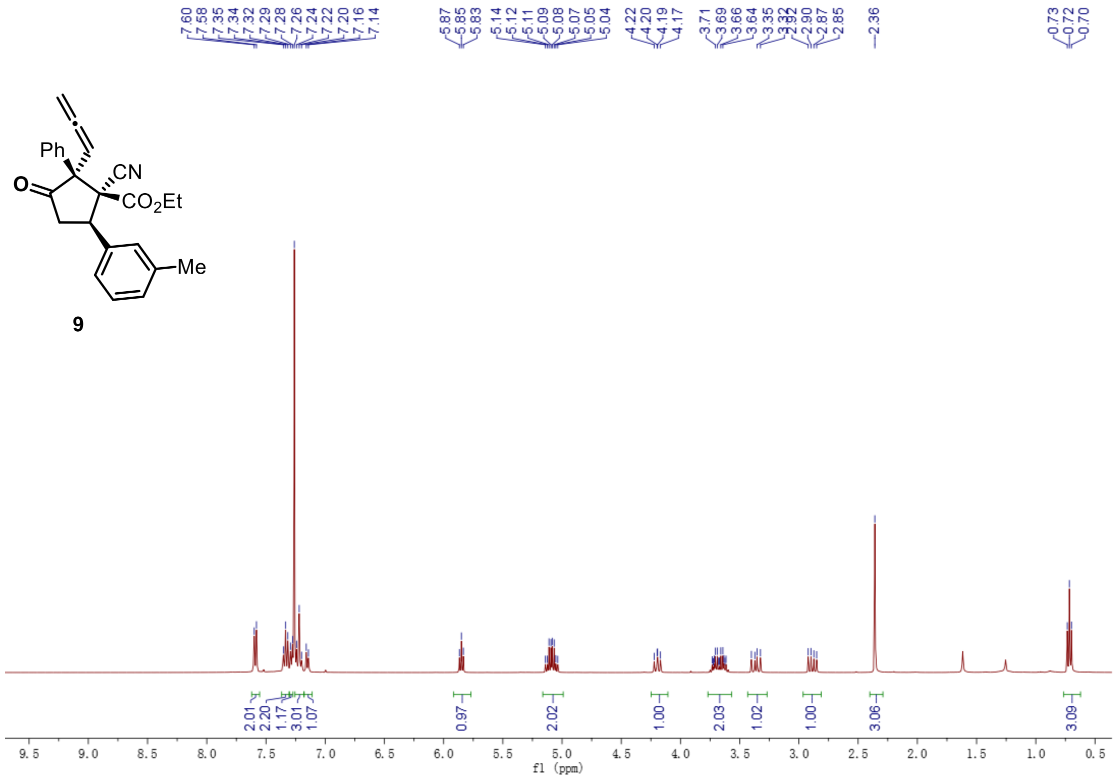

^{13}C NMR (100 MHz, $CDCl_3$)

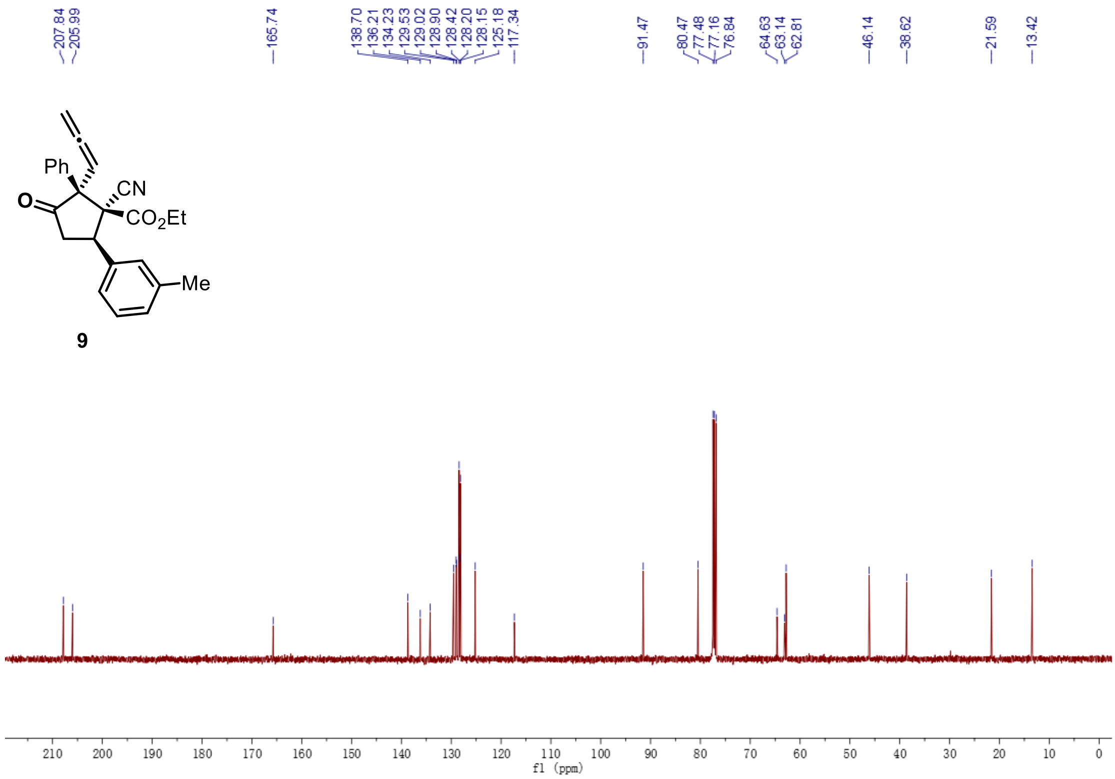

^{1}H NMR (400 MHz, $CDCl_3$)

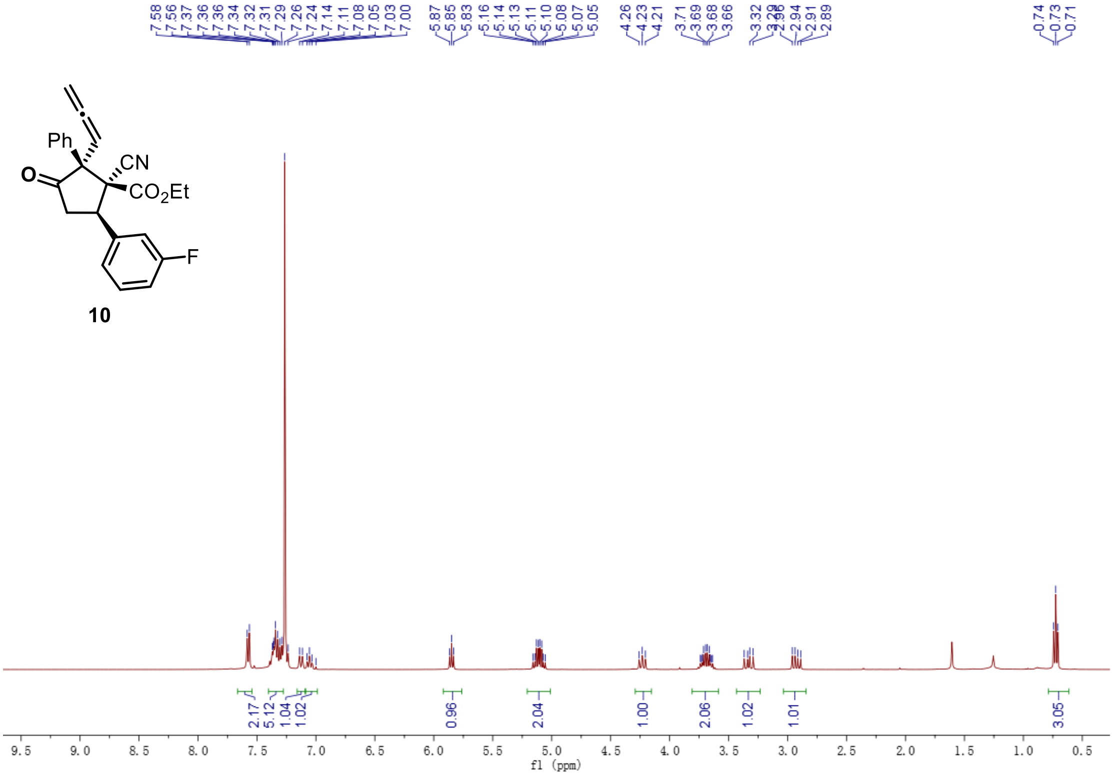

^{13}C NMR (100 MHz, $CDCl_3$)

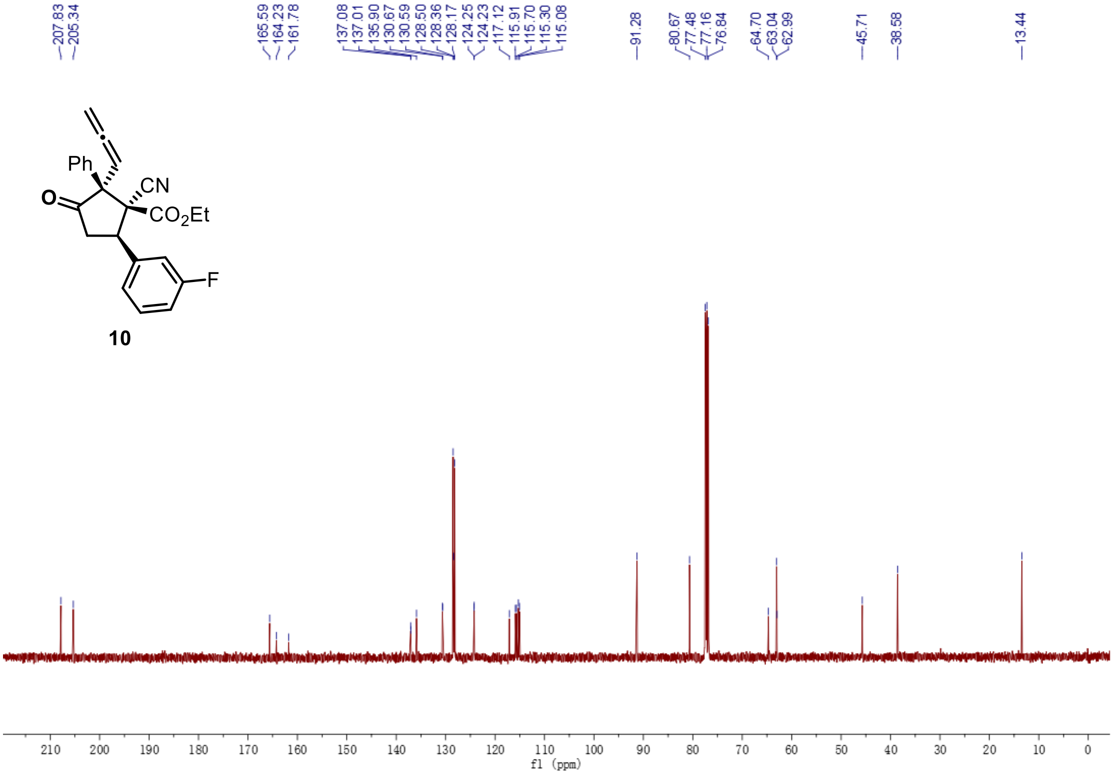

^{19}F NMR (376 MHz, $CDCl_3$)

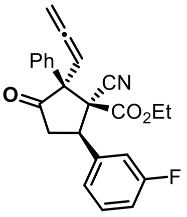

10

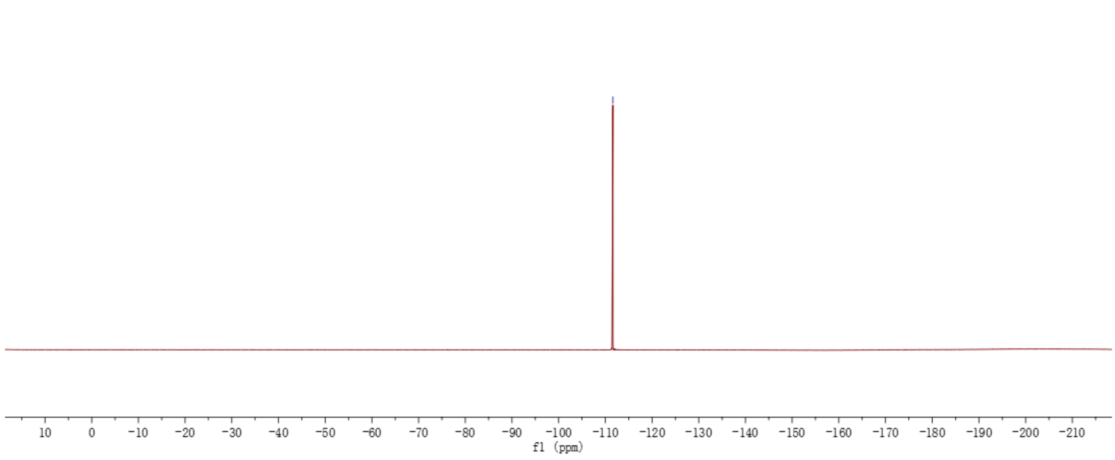

^{1}H NMR (400 MHz, $CDCl_3$)

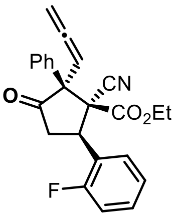

11

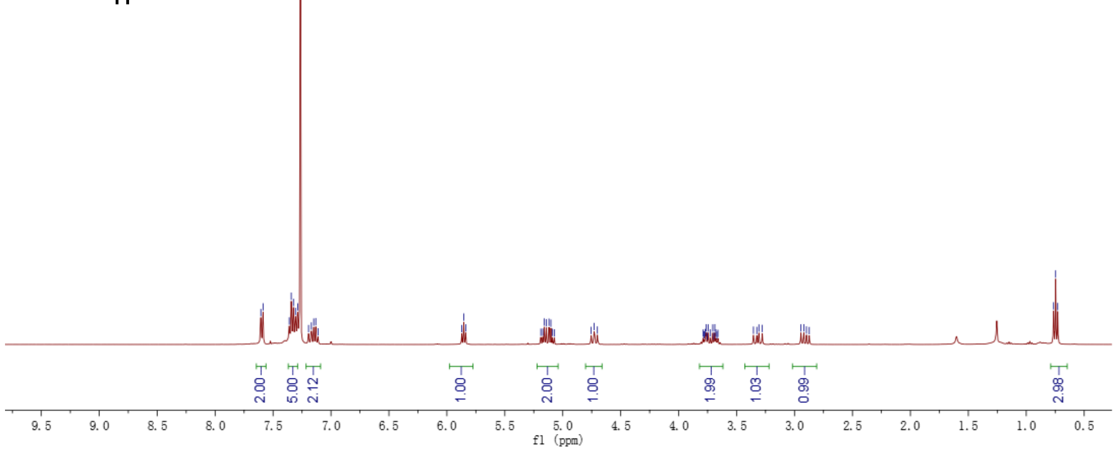

^{13}C NMR (100 MHz, $CDCl_3$)

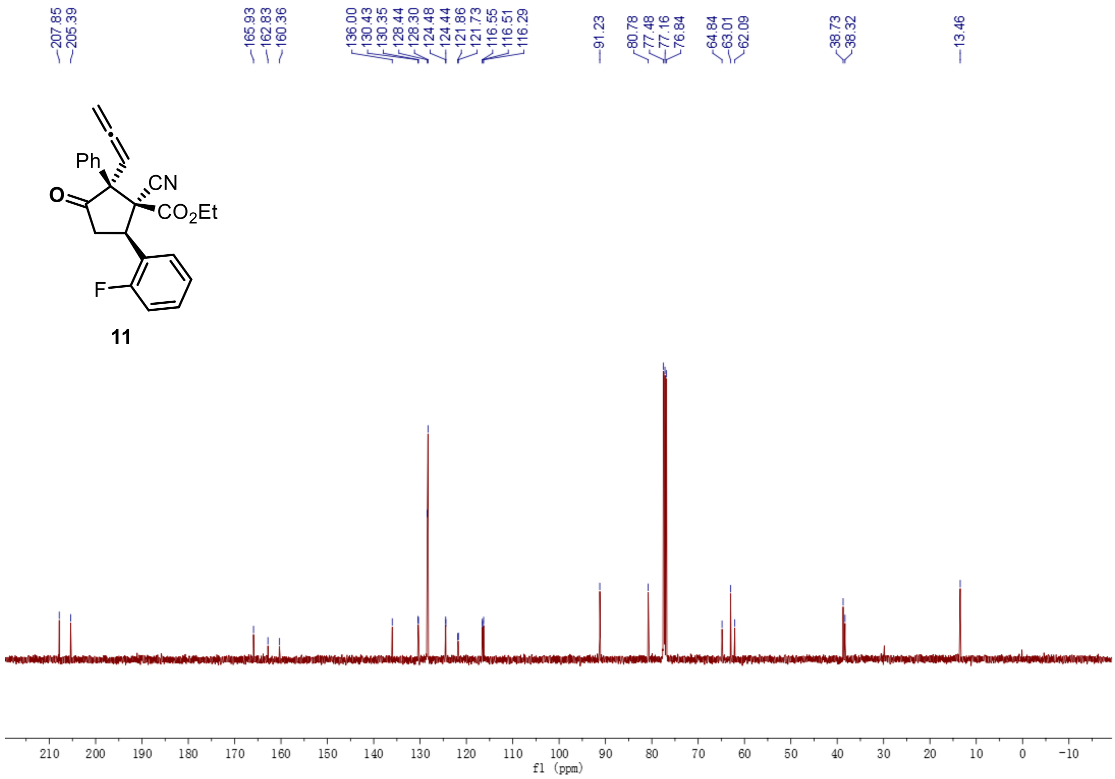

^{19}F NMR (376 MHz, $CDCl_3$)

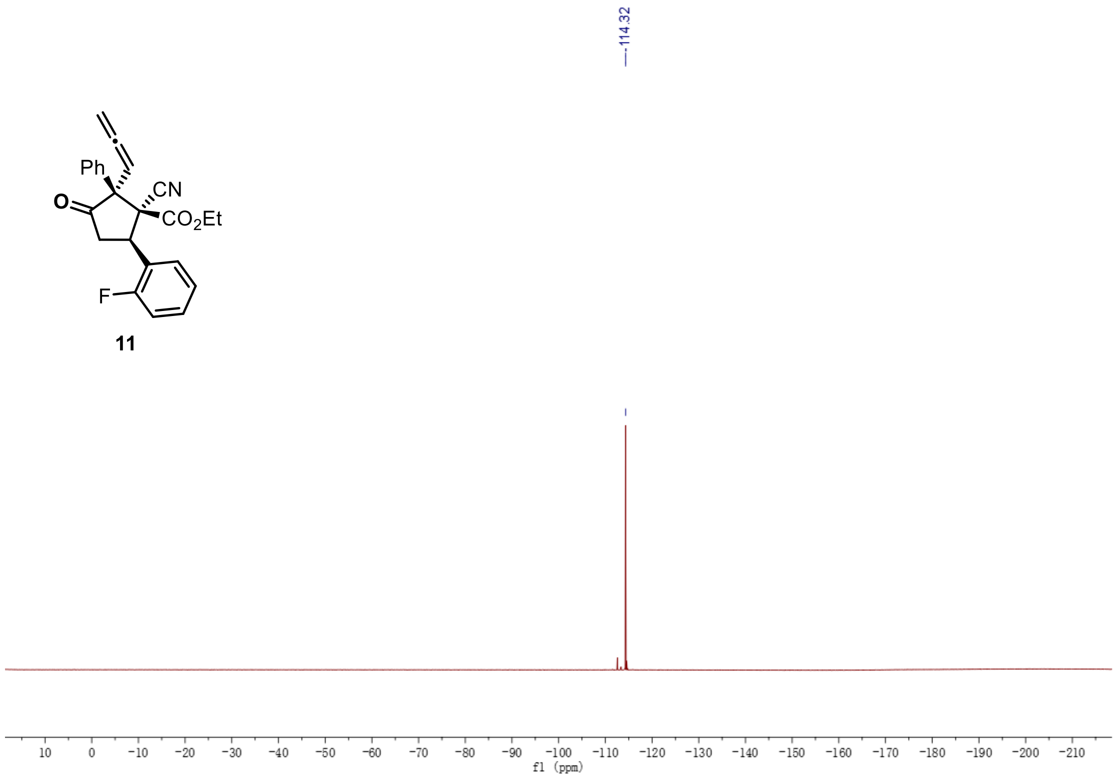

^{1}H NMR (400 MHz, $CDCl_3$)

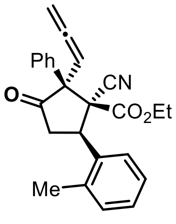

12

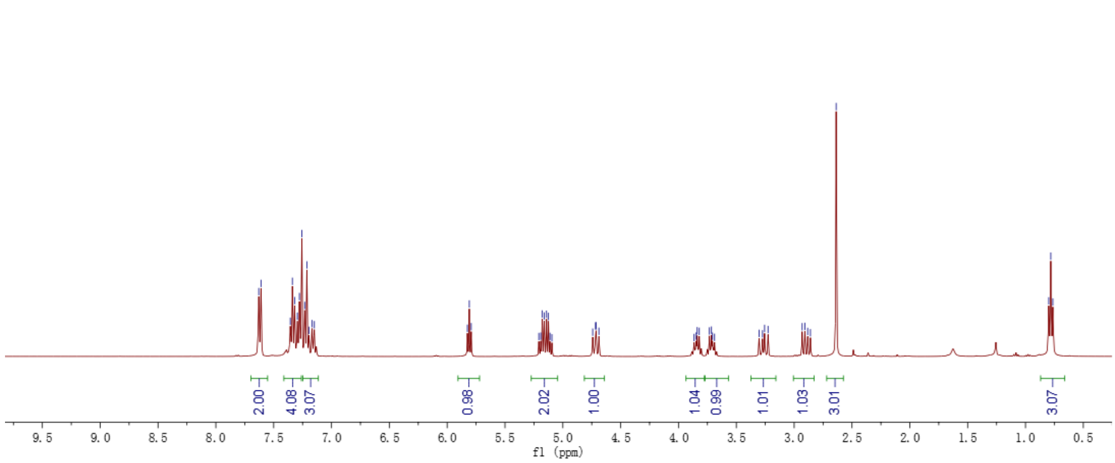

^{13}C NMR (100 MHz, $CDCl_3$)

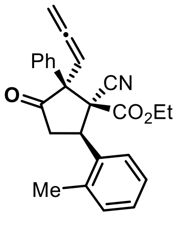

12

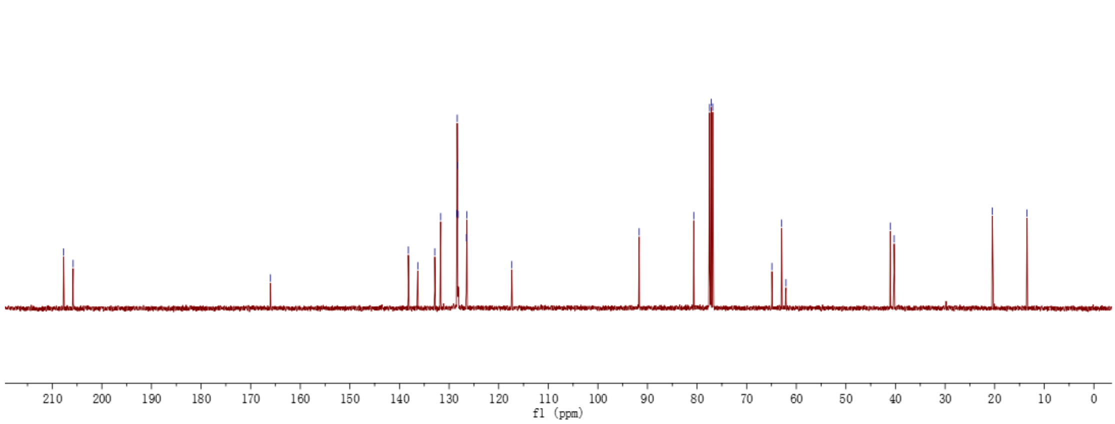

^{1}H NMR (400 MHz, $CDCl_3$)

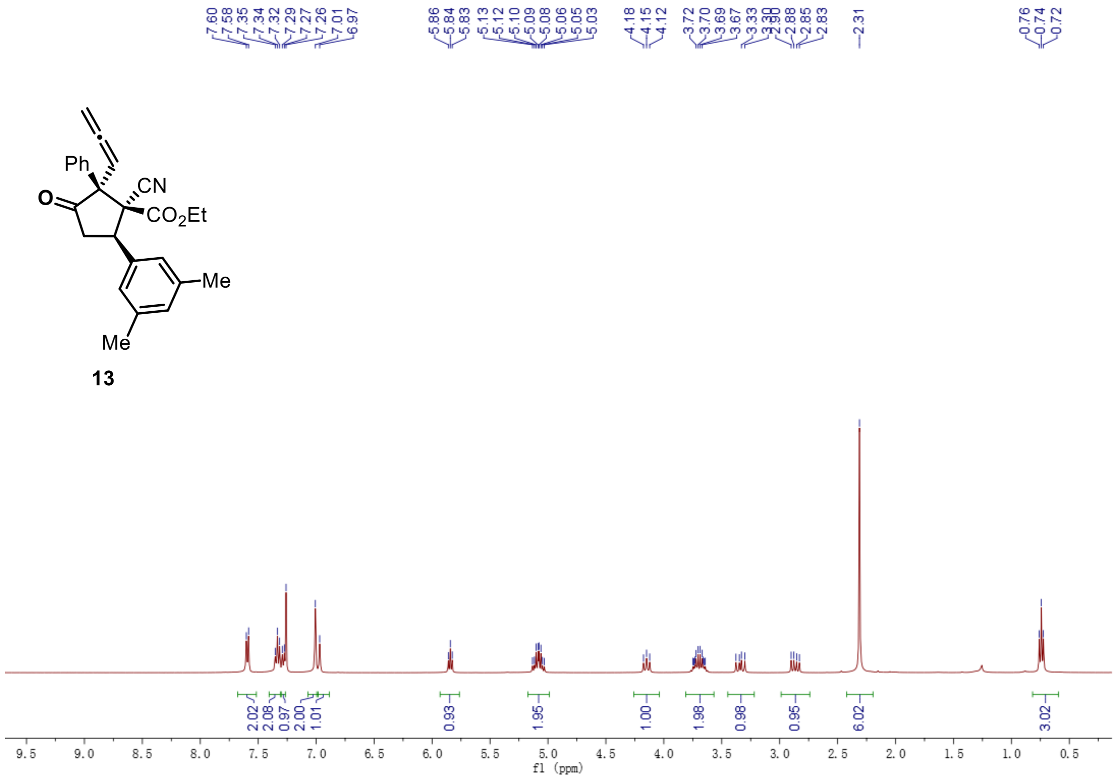

^{13}C NMR (100 MHz, $CDCl_3$)

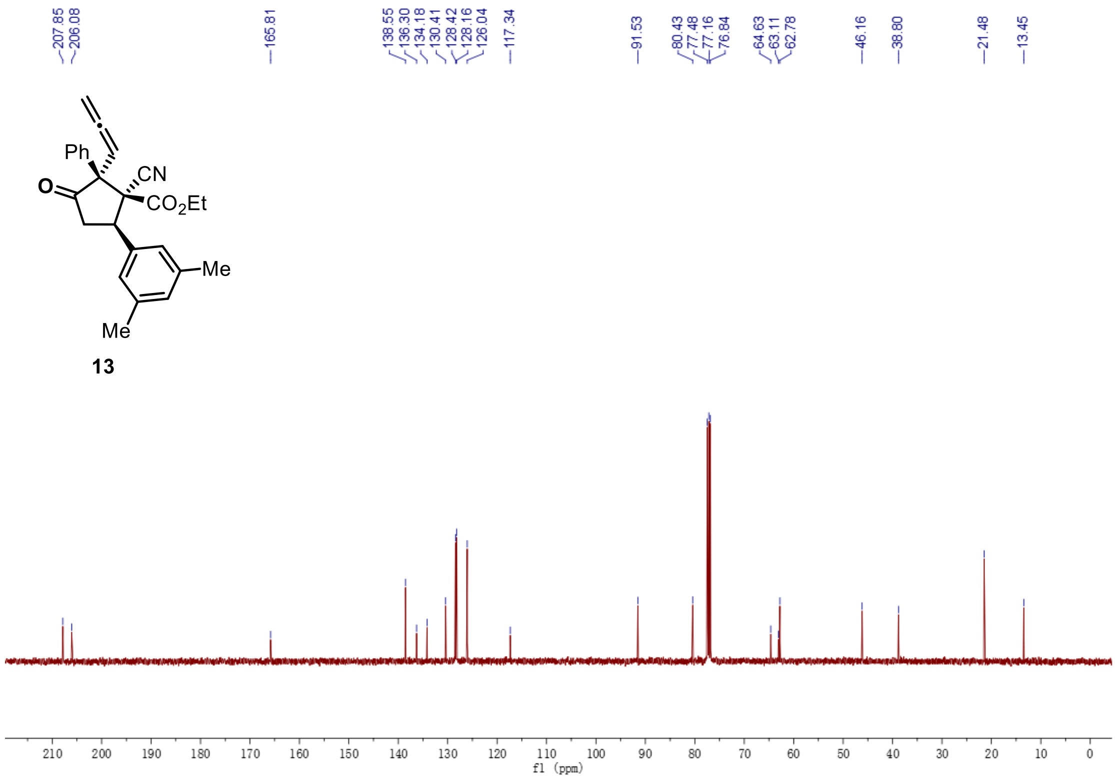

^{1}H NMR (400 MHz, $CDCl_3$)

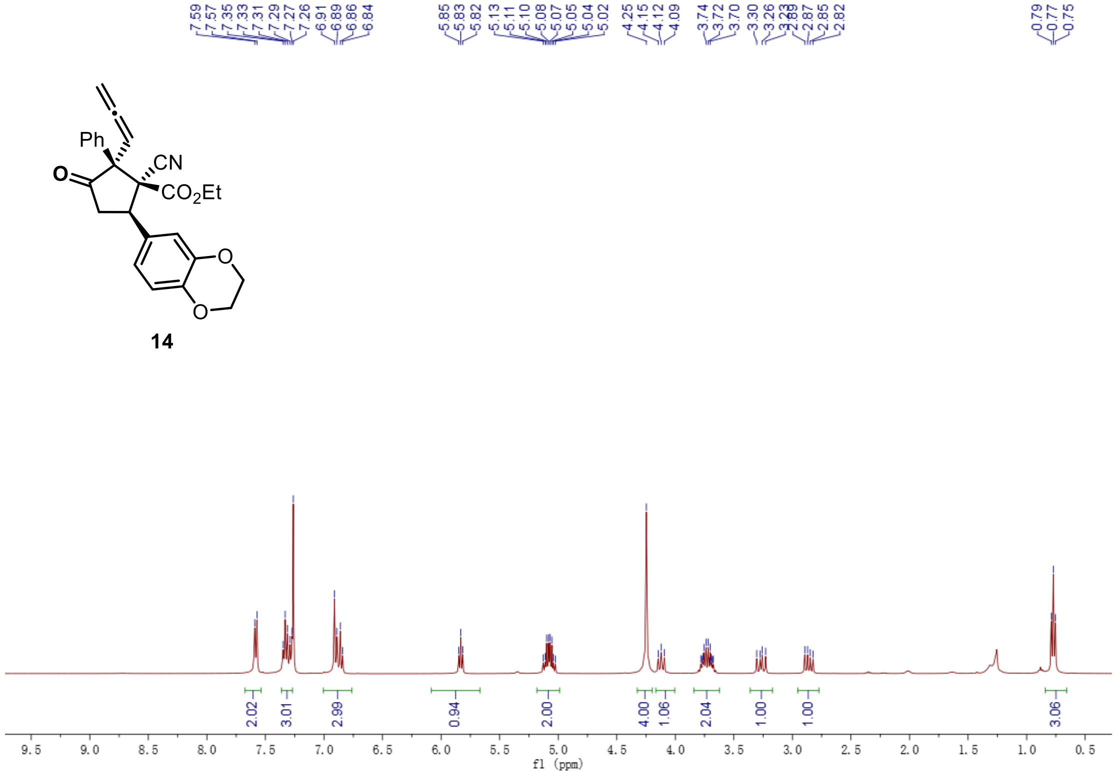

^{13}C NMR (100 MHz, $CDCl_3$)

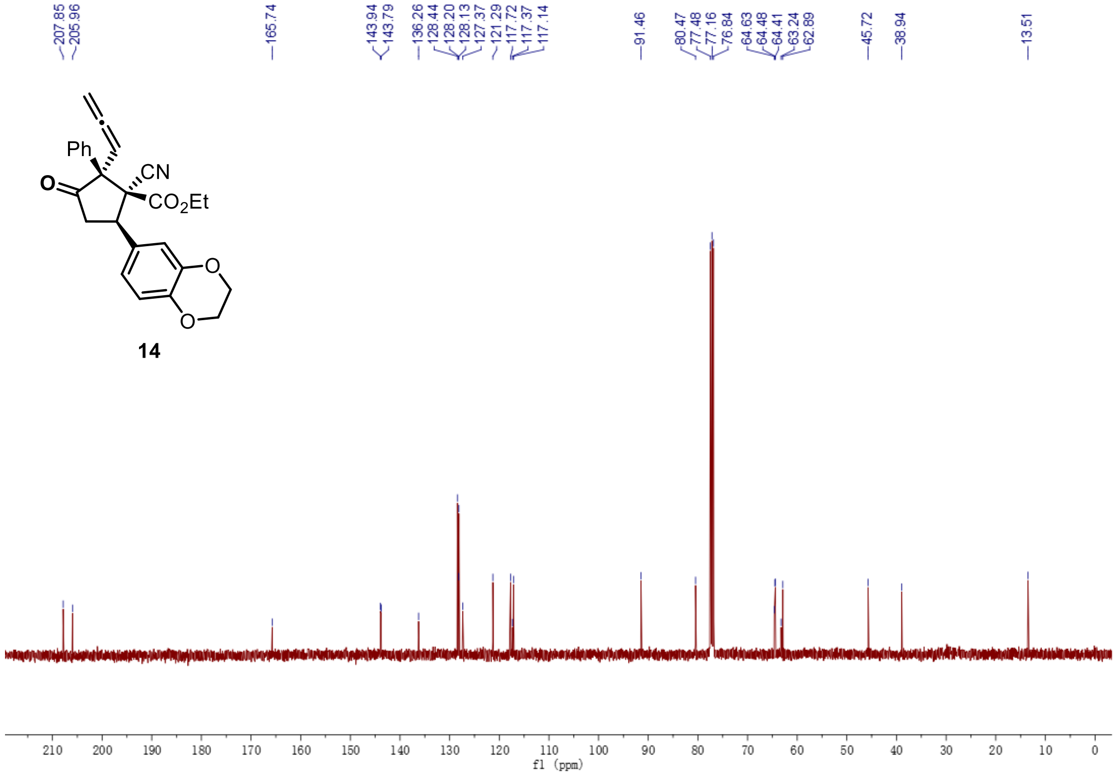

^{1}H NMR (400 MHz, $CDCl_3$)

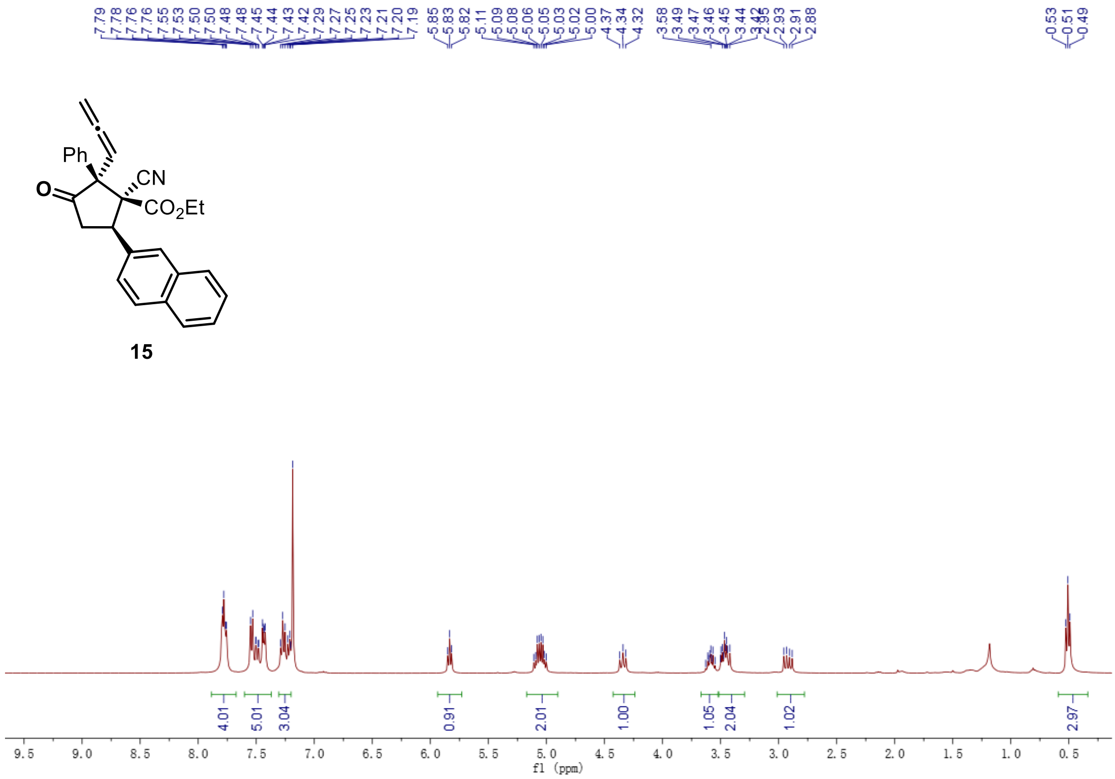

^{13}C NMR (100 MHz, $CDCl_3$)

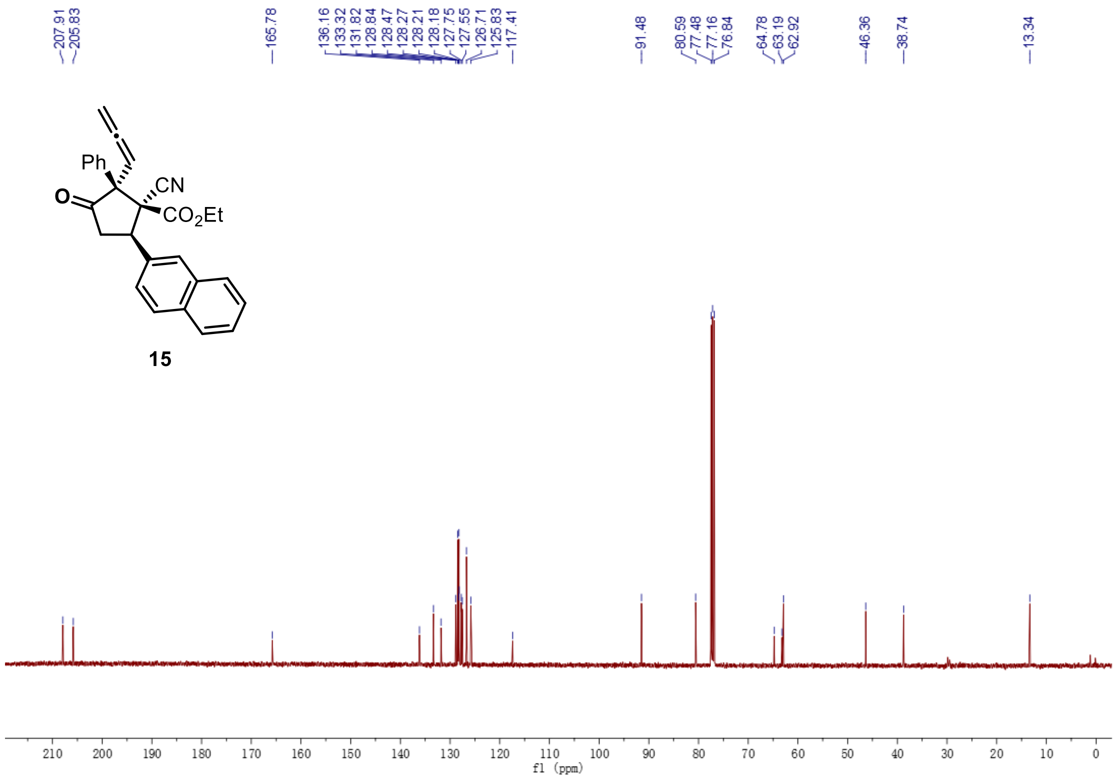

^{1}H NMR (400 MHz, $CDCl_3$)

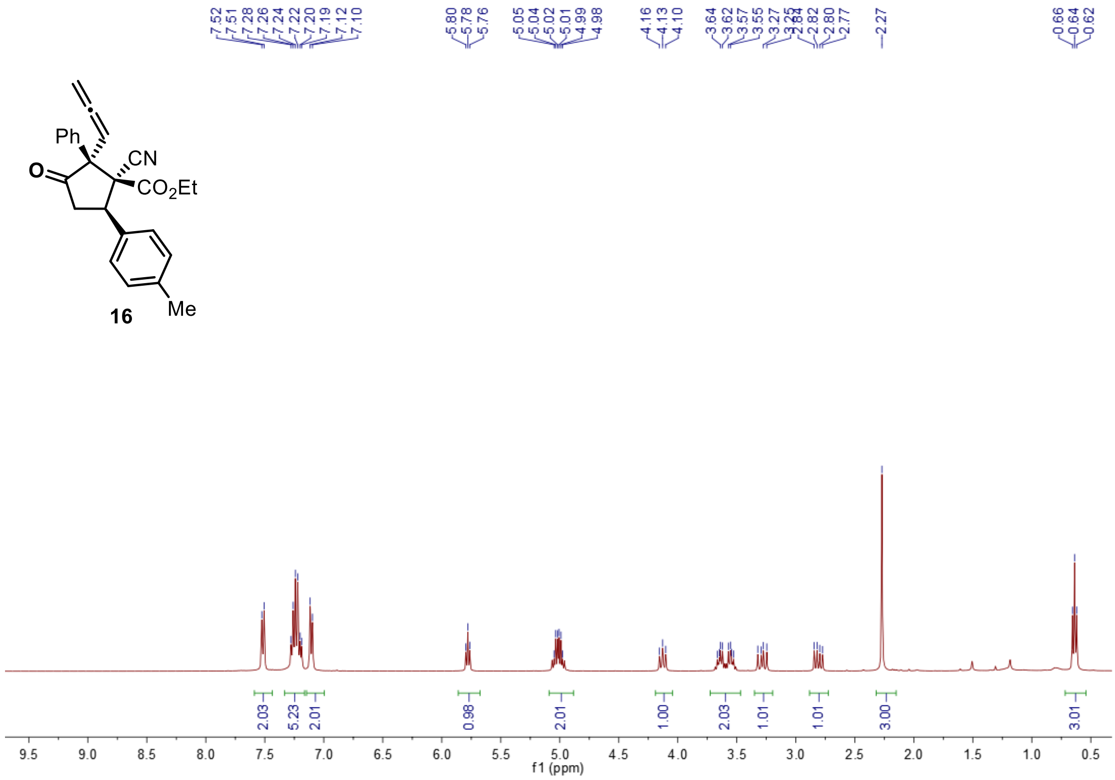

^{13}C NMR (100 MHz, $CDCl_3$)

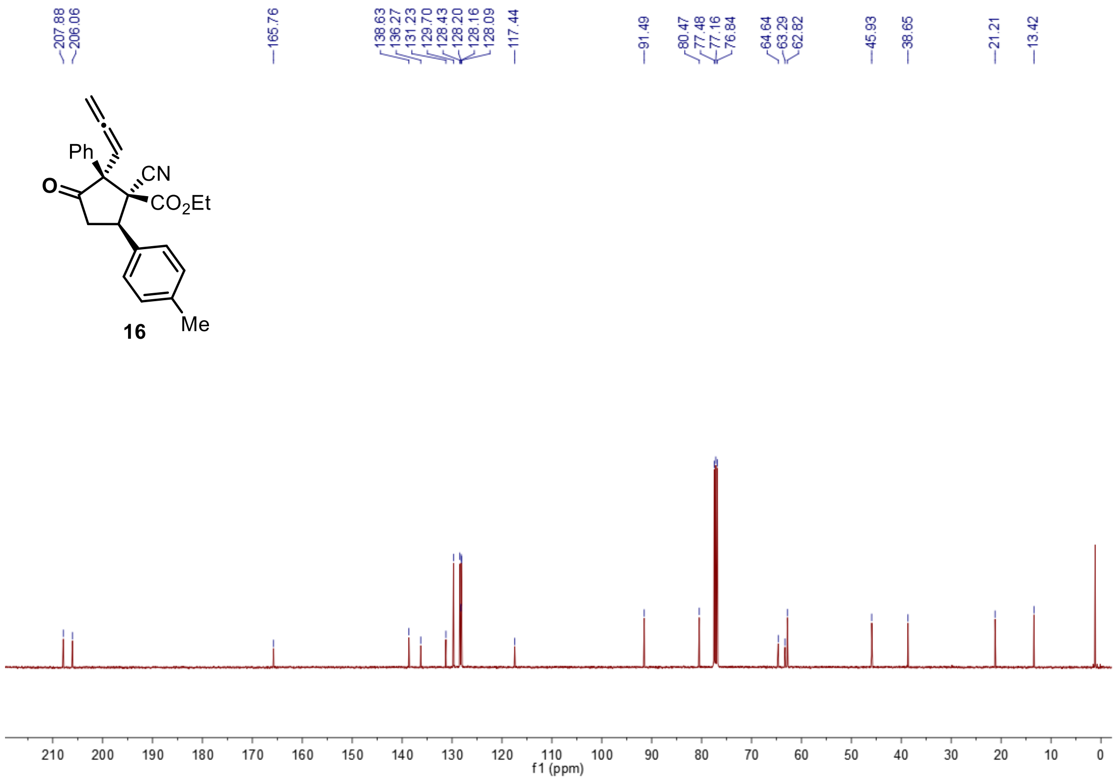

^{1}H NMR (400 MHz, $CDCl_3$)

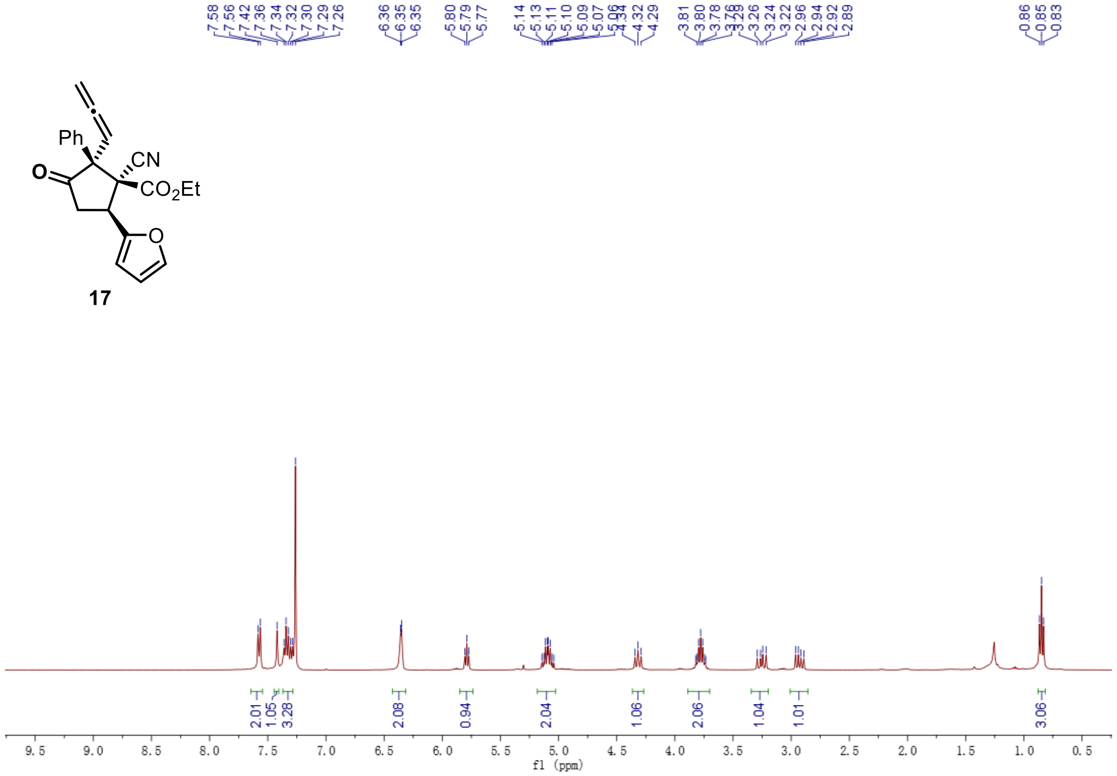

^{13}C NMR (100 MHz, $CDCl_3$)

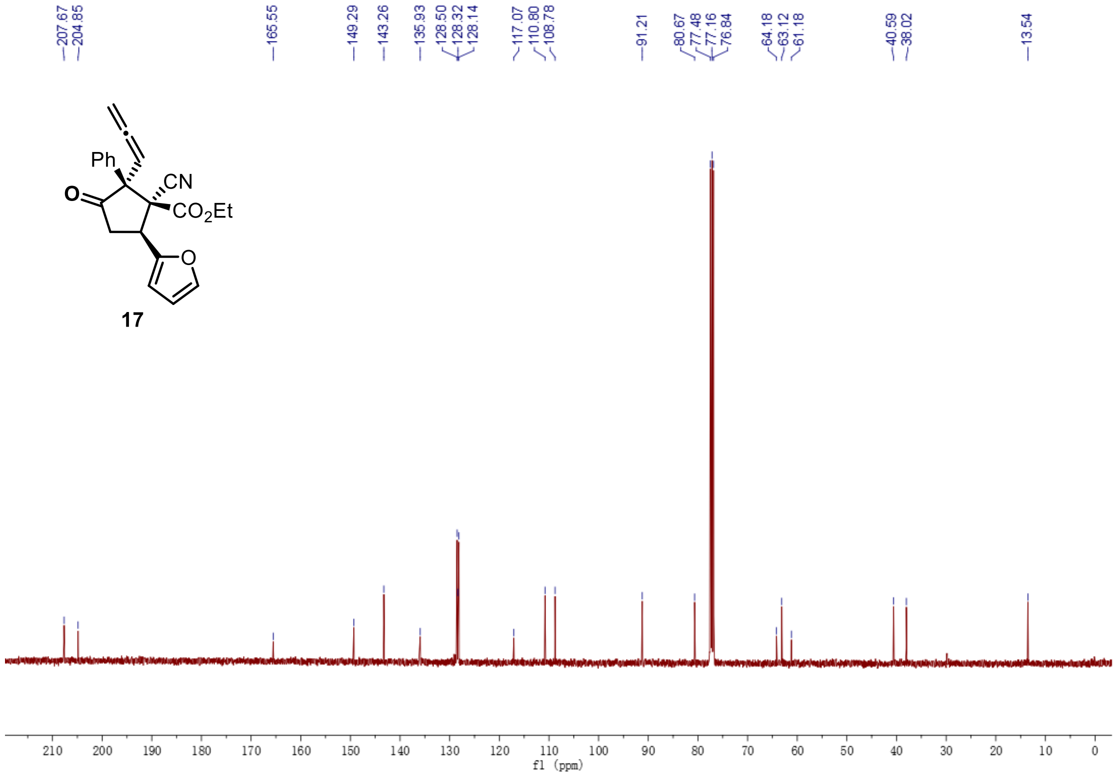

^{1}H NMR (400 MHz, $CDCl_3$)

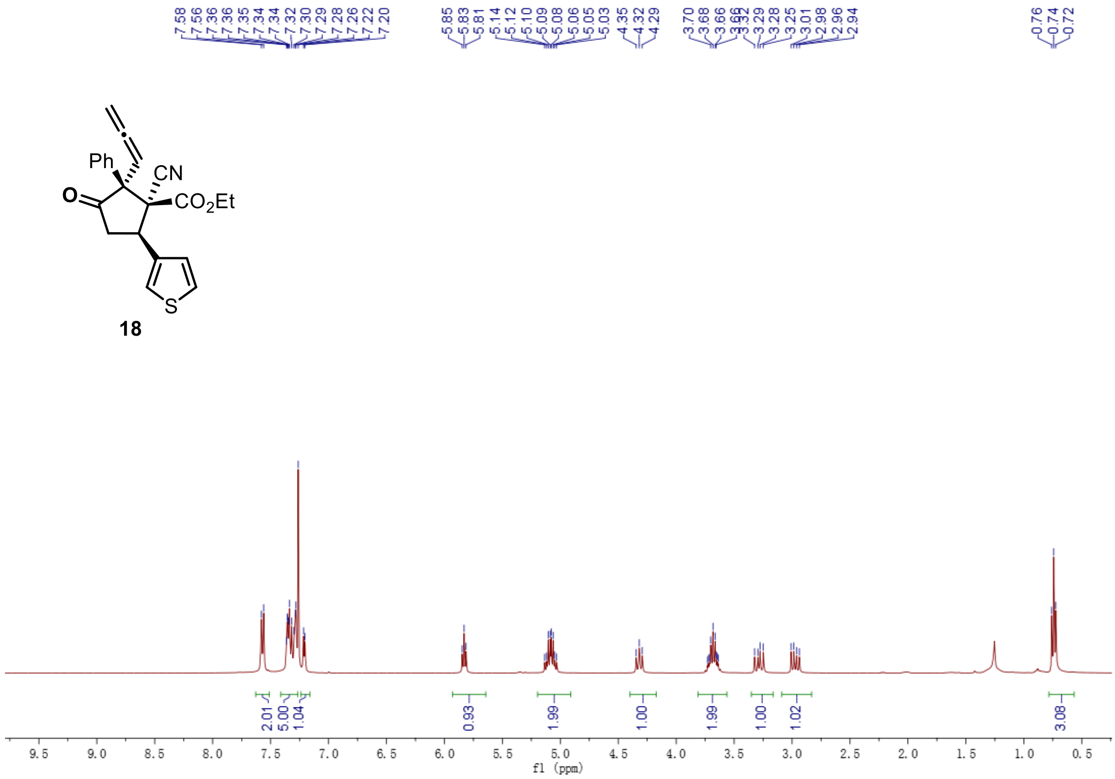

^{13}C NMR (100 MHz, $CDCl_3$)

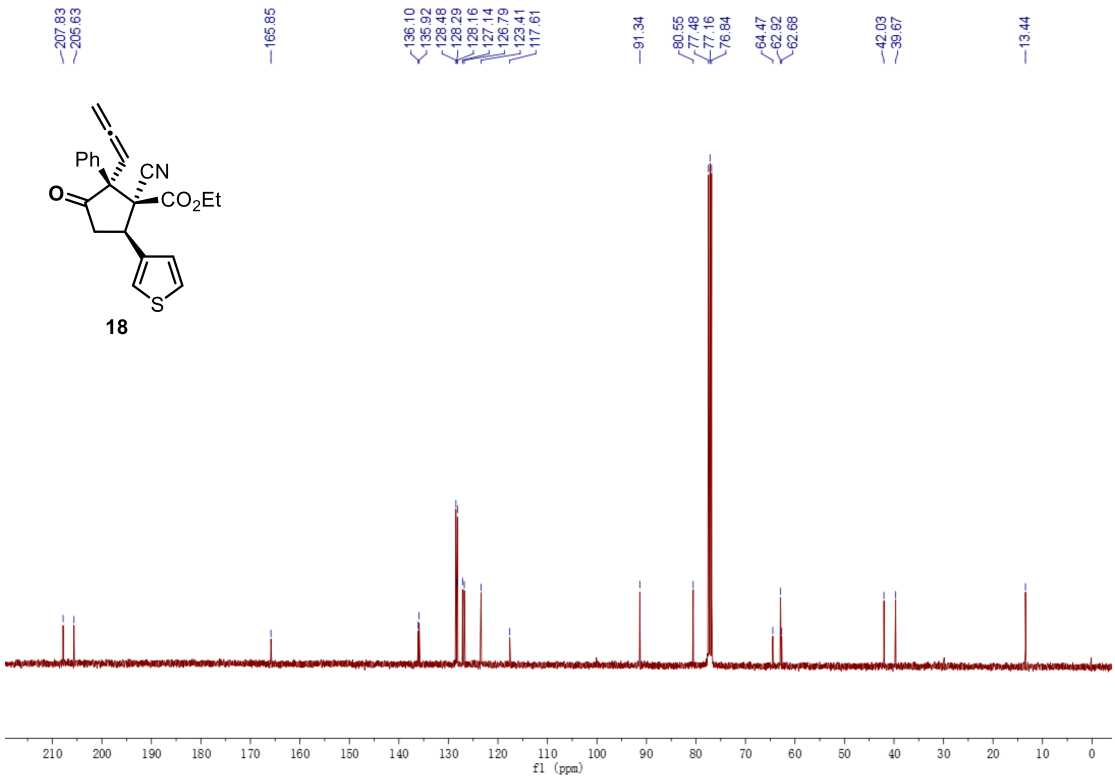

^{1}H NMR (400 MHz, $CDCl_3$)

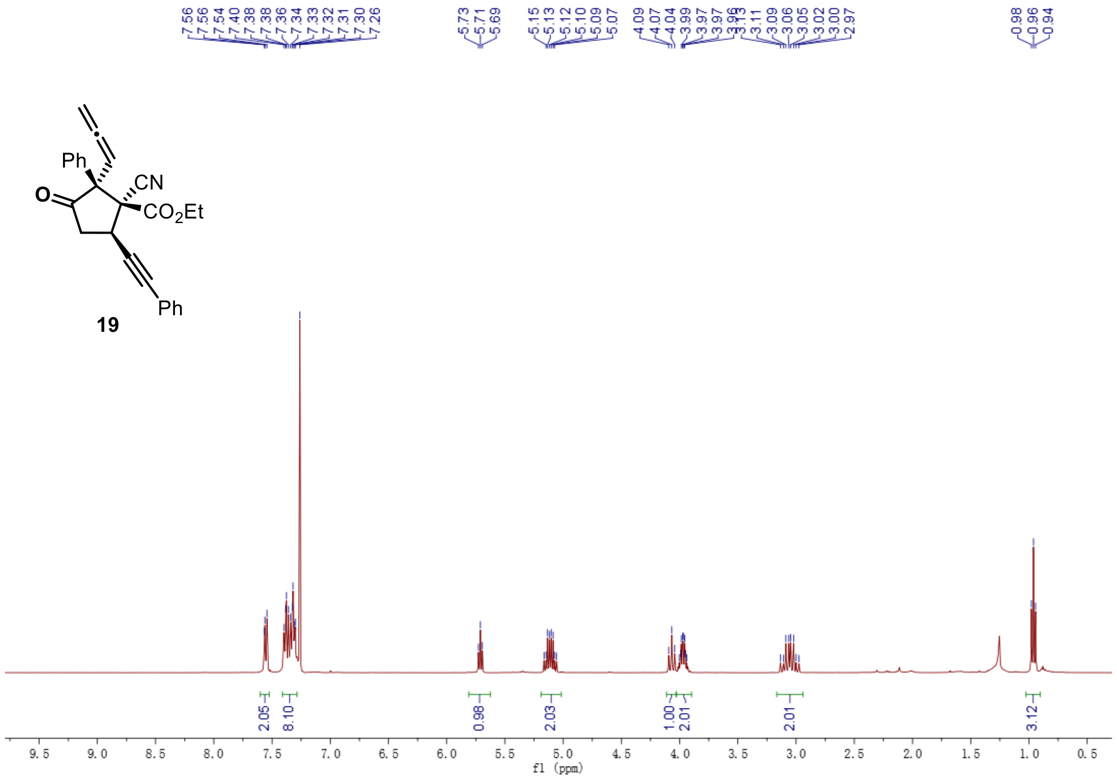

13C NMR (100 MHz, $CDCl_3$)

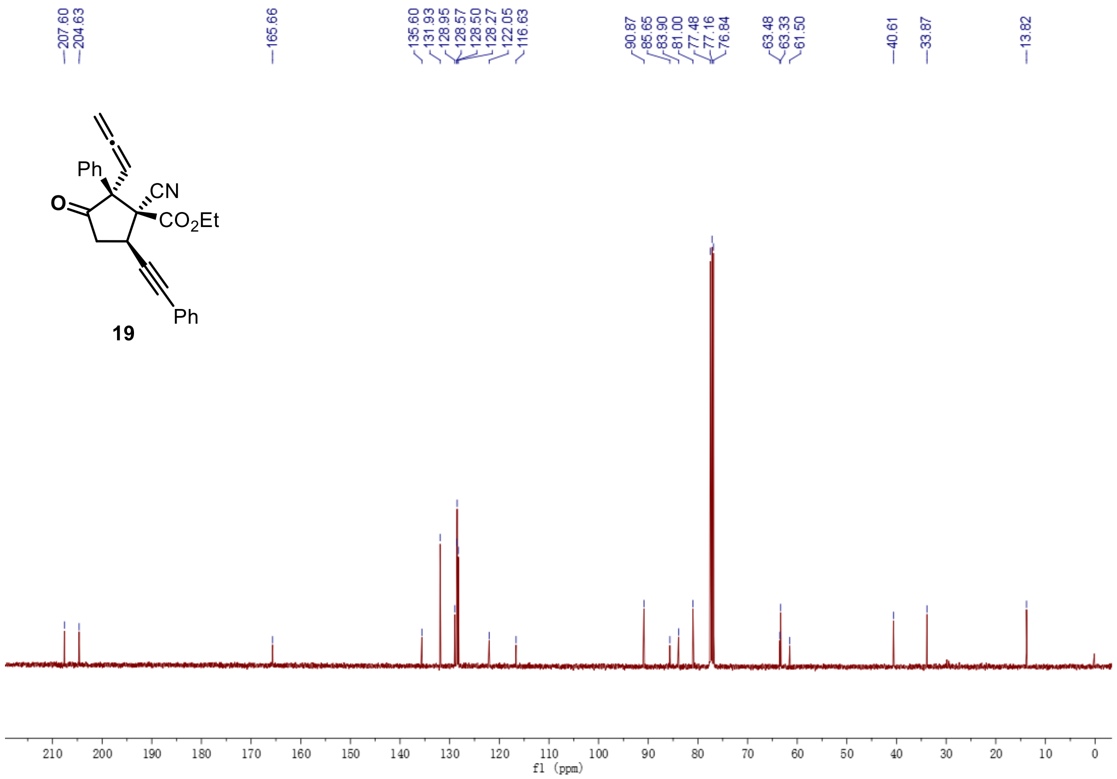

^{1}H NMR (400 MHz, $CDCl_3$)

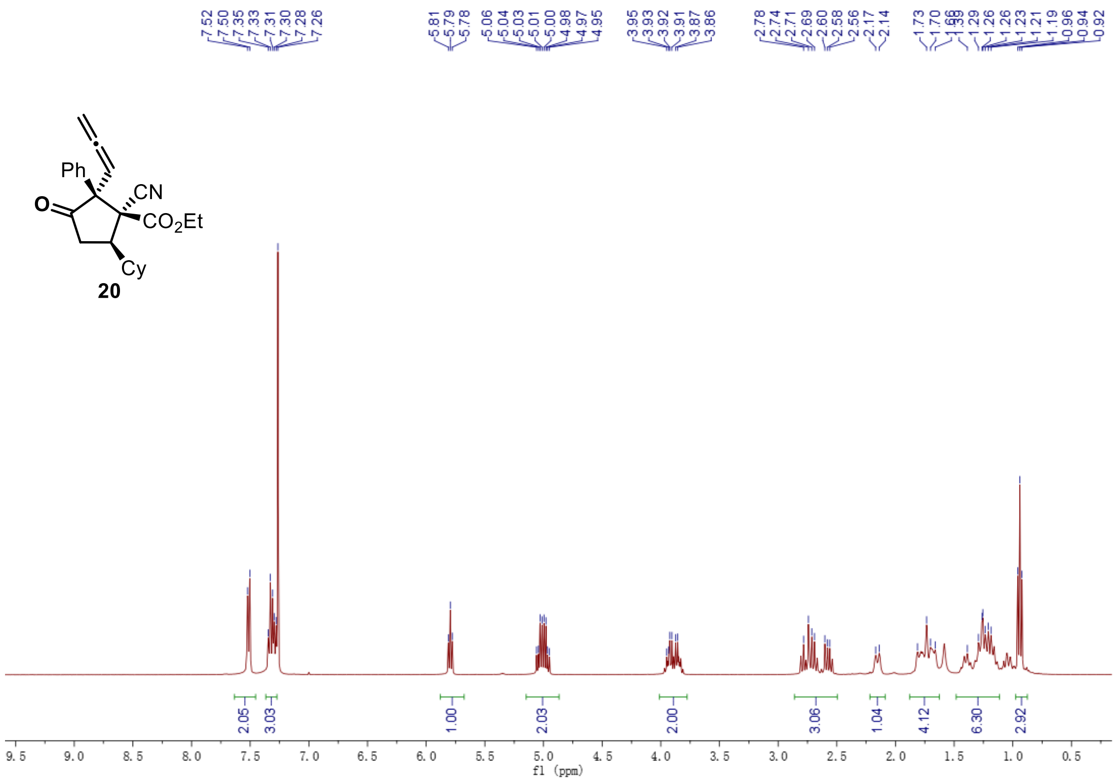

^{13}C NMR (100 MHz, $CDCl_3$)

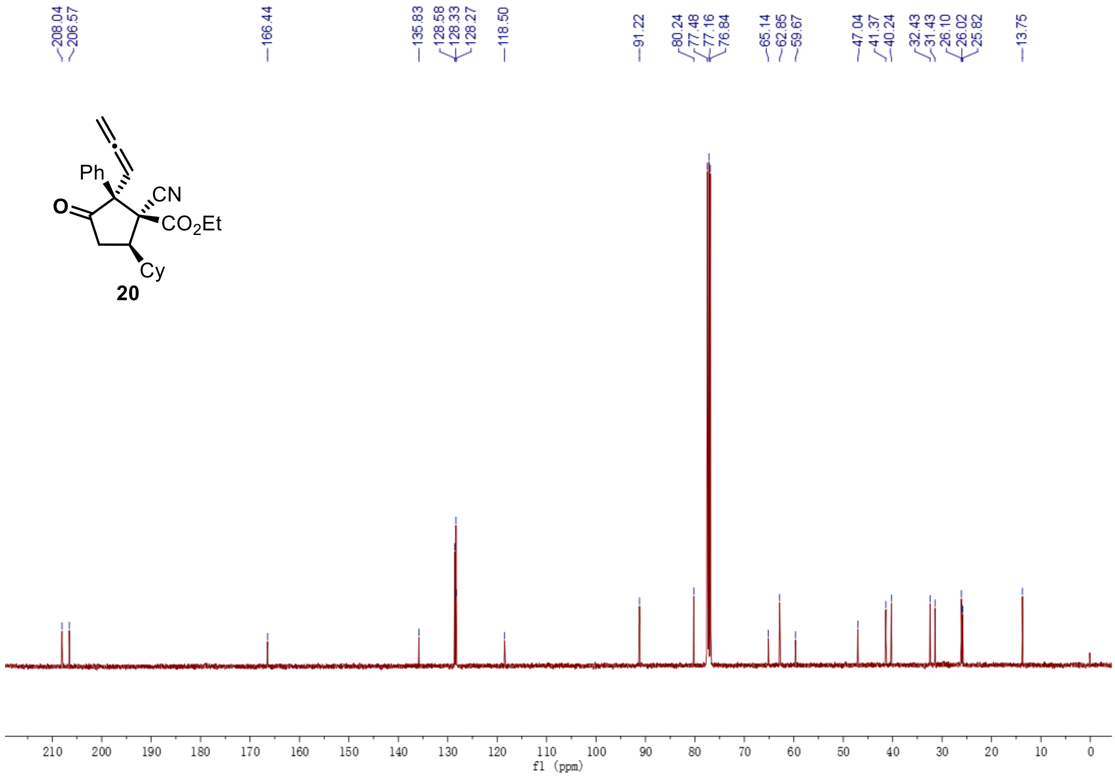

^{1}H NMR (400 MHz, $CDCl_3$)

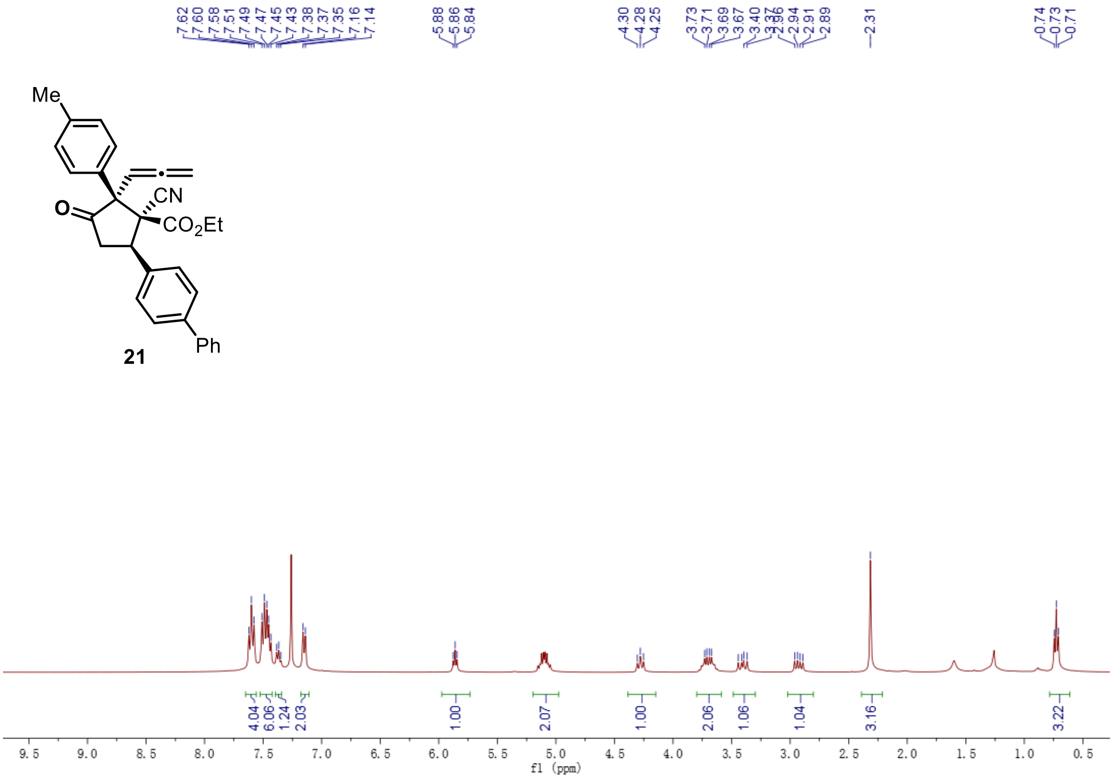

^{13}C NMR (100 MHz, $CDCl_3$)

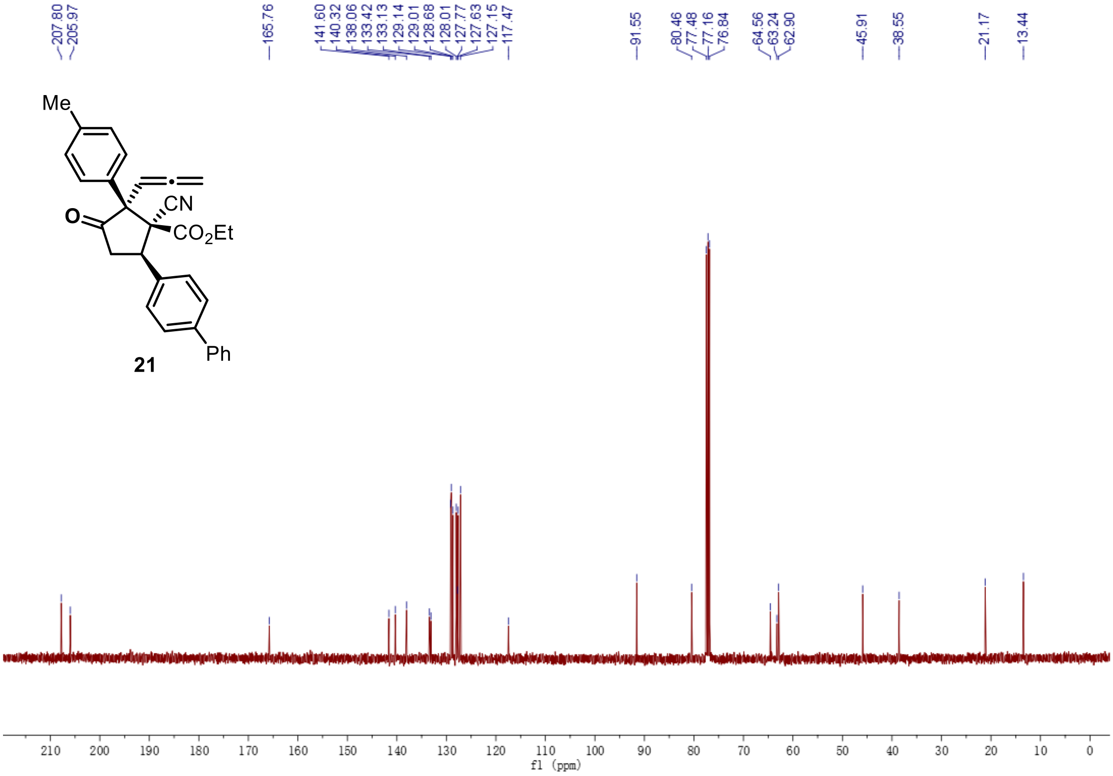

^{1}H NMR (400 MHz, $CDCl_3$)

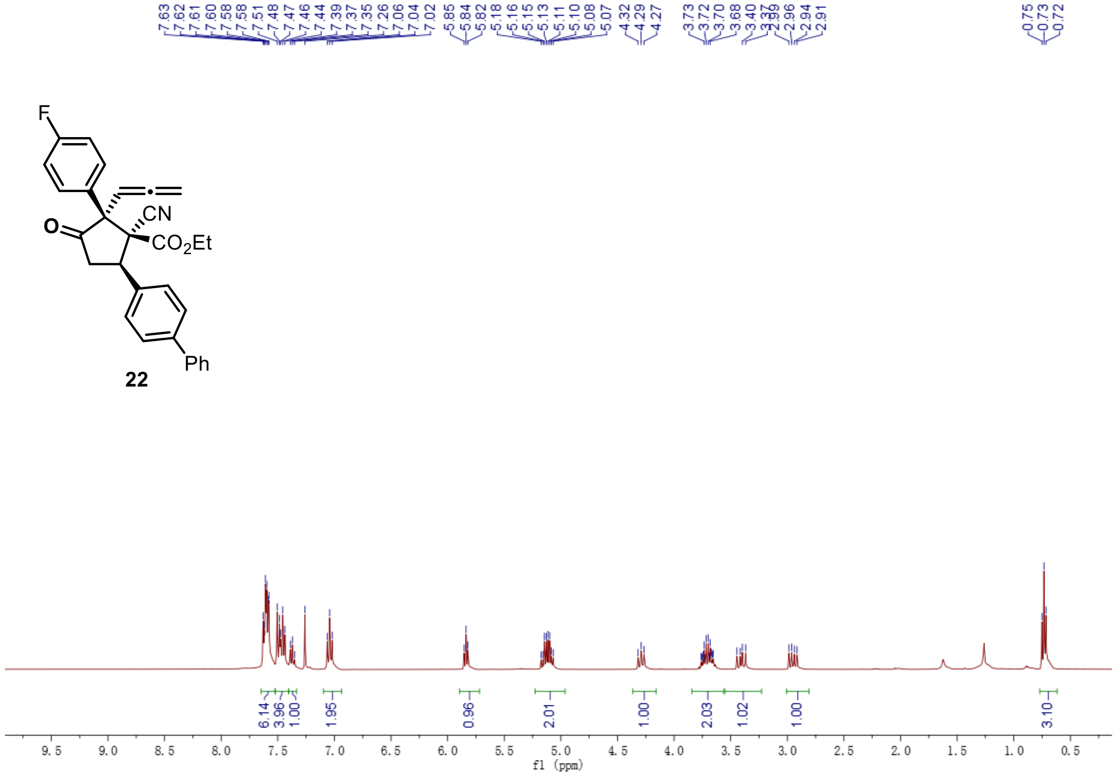

^{13}C NMR (100 MHz, $CDCl_3$)

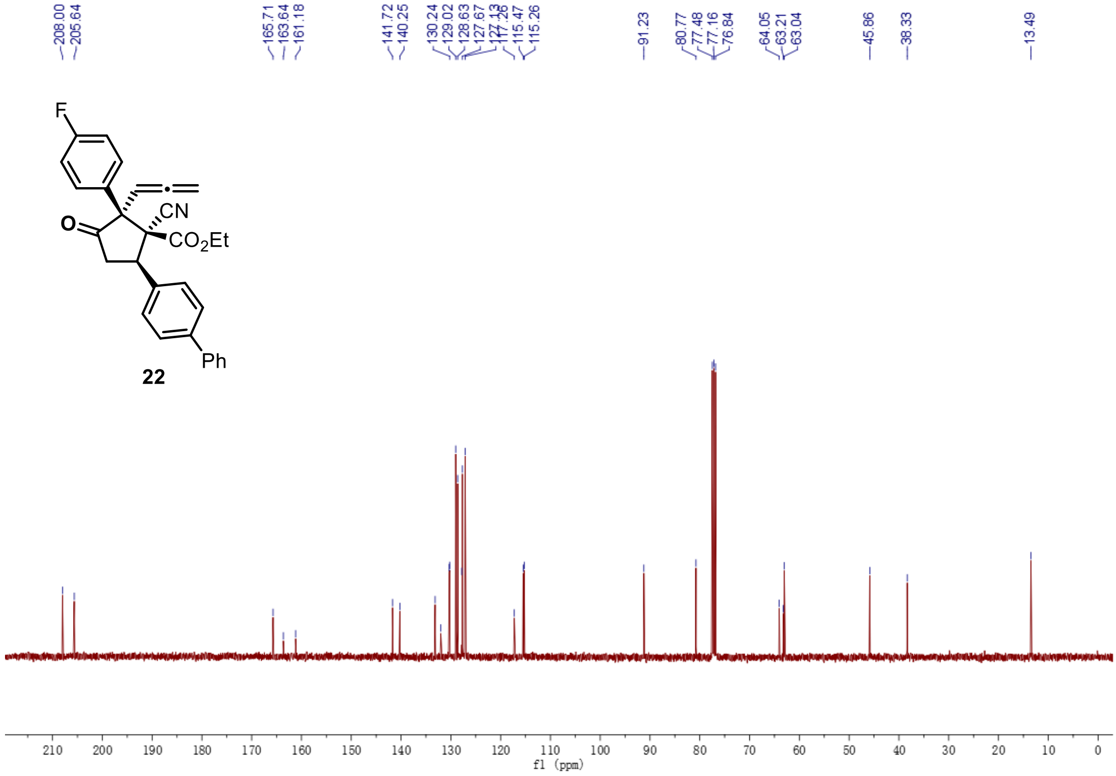

^{19}F NMR (376 MHz, $CDCl_3$)

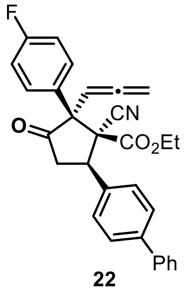

22

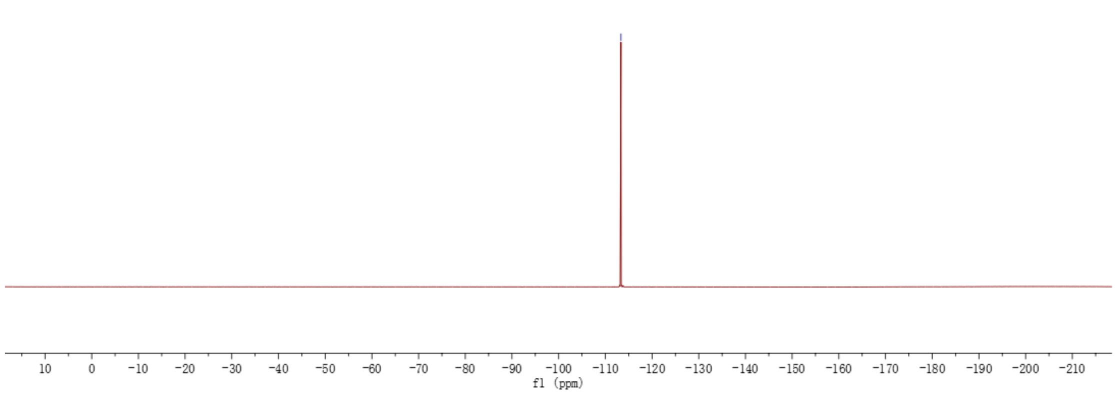

^{1}H NMR (400 MHz, $CDCl_3$)

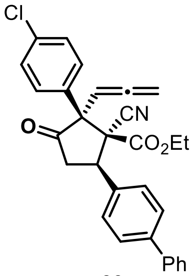

23

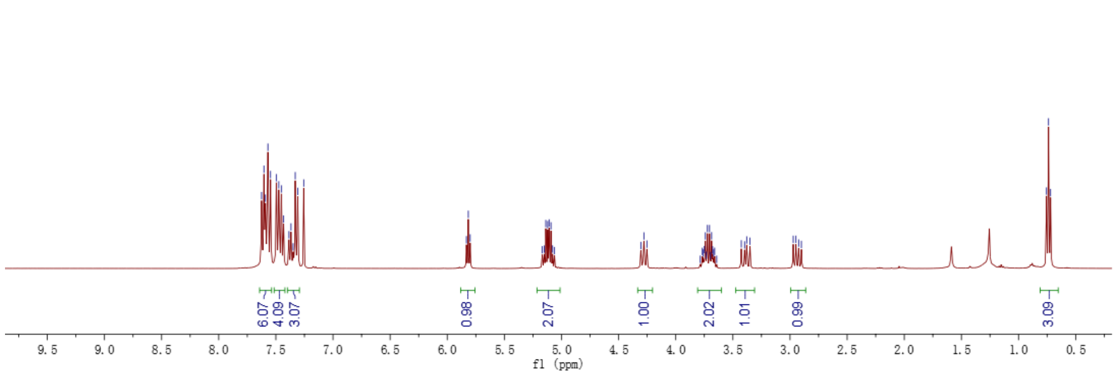

^{13}C NMR (100 MHz, $CDCl_3$)

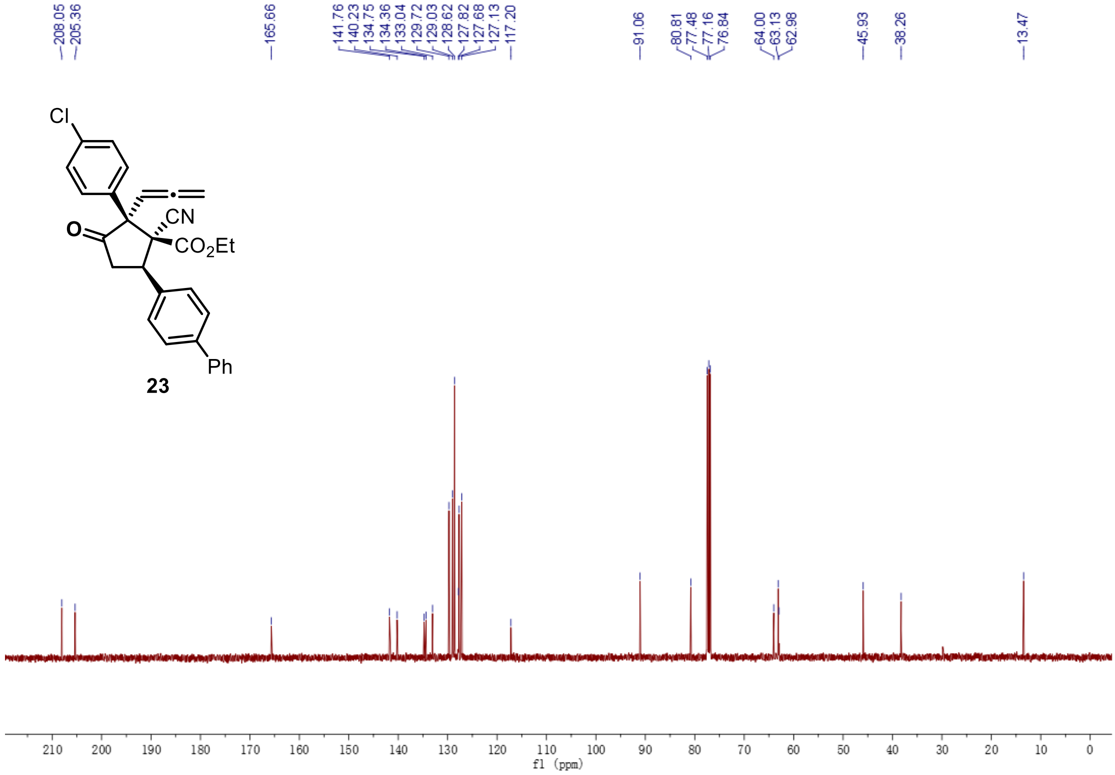

23

^{1}H NMR (400 MHz, $CDCl_3$)

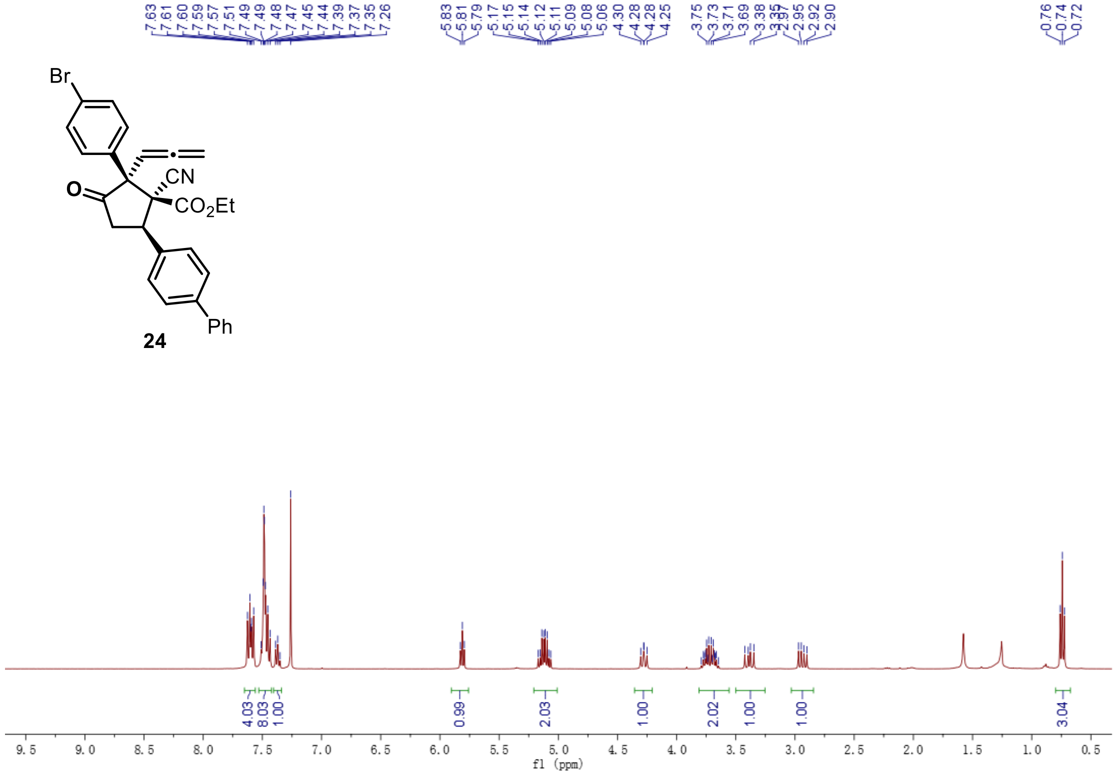

24

^{13}C NMR (100 MHz, $CDCl_3$)

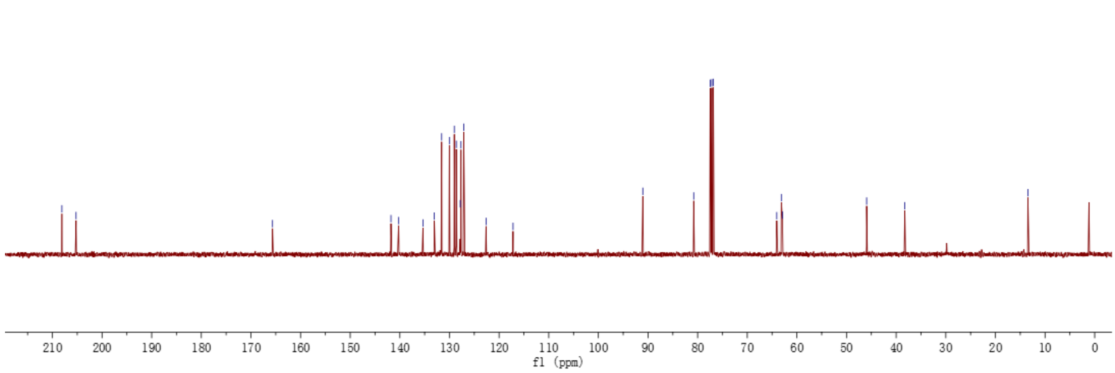

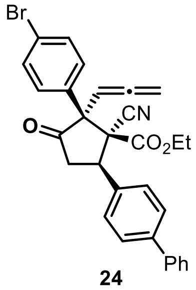

24

^{1}H NMR (400 MHz, $CDCl_3$)

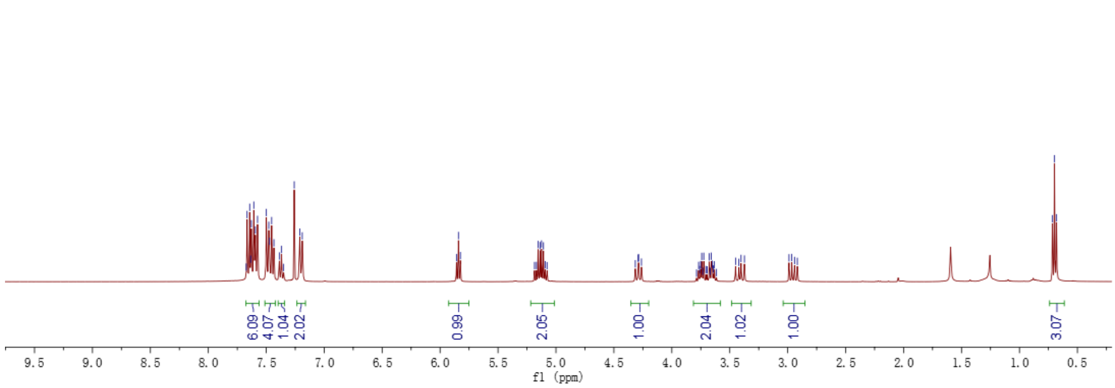

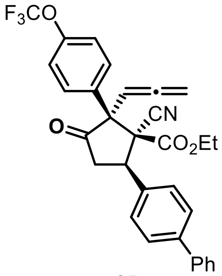

25

^{13}C NMR (100 MHz, $CDCl_3$)

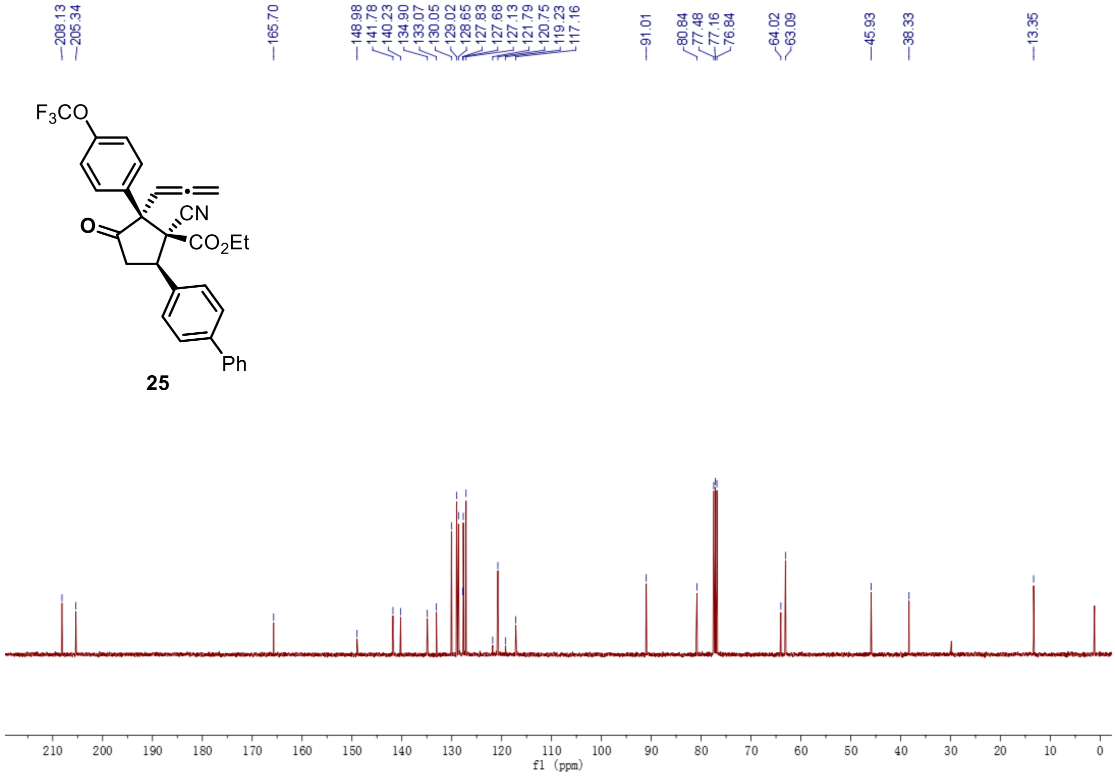

^{19}F NMR (376 MHz, $CDCl_3$)

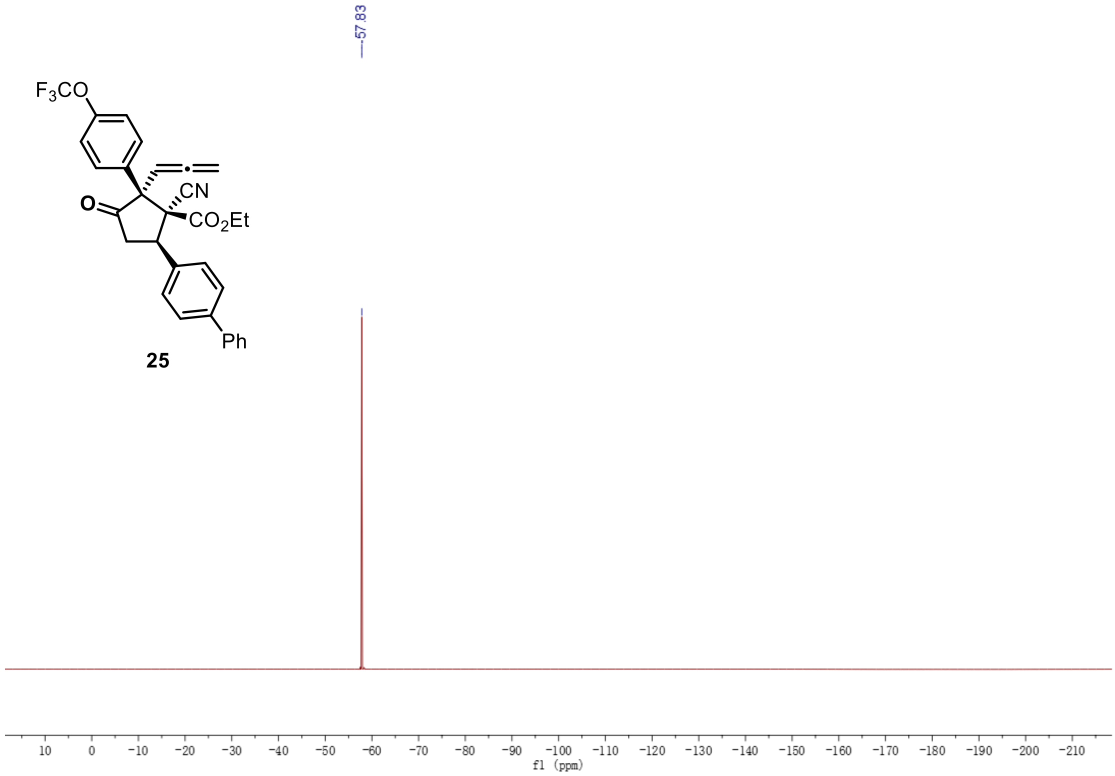

^1H NMR (400 MHz, $CDCl_3$)

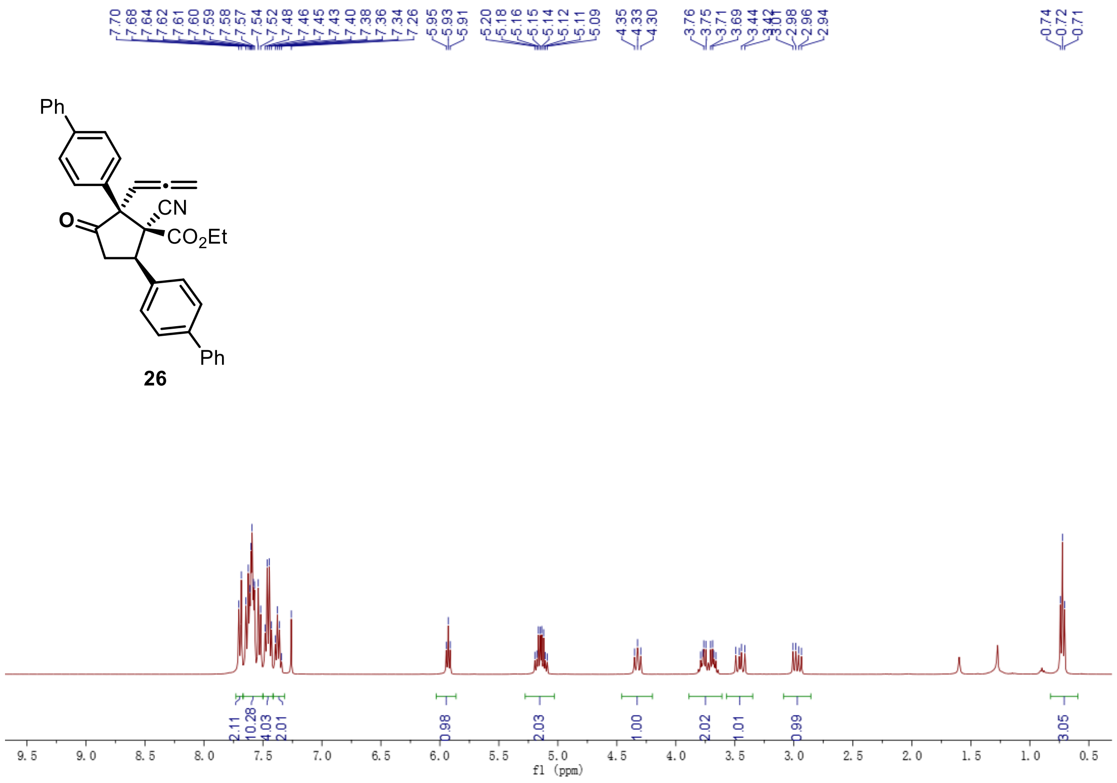

^{13}C NMR (100 MHz, $CDCl_3$)

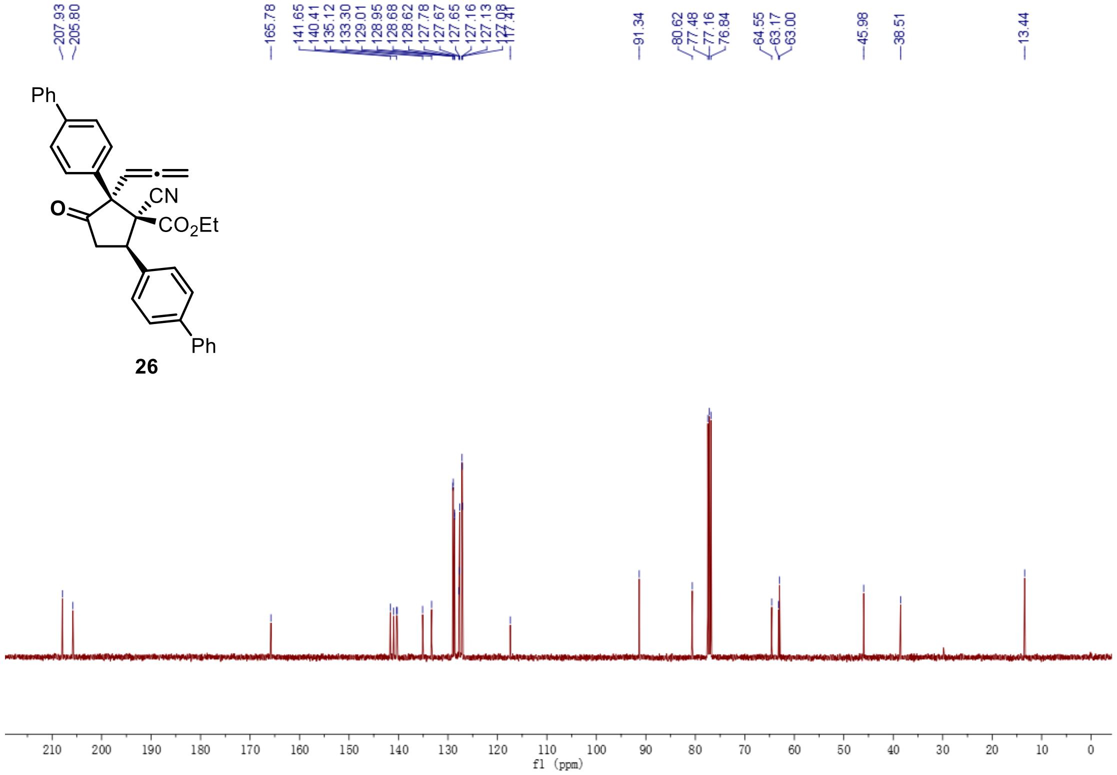

^{1}H NMR (400 MHz, $CDCl_3$)

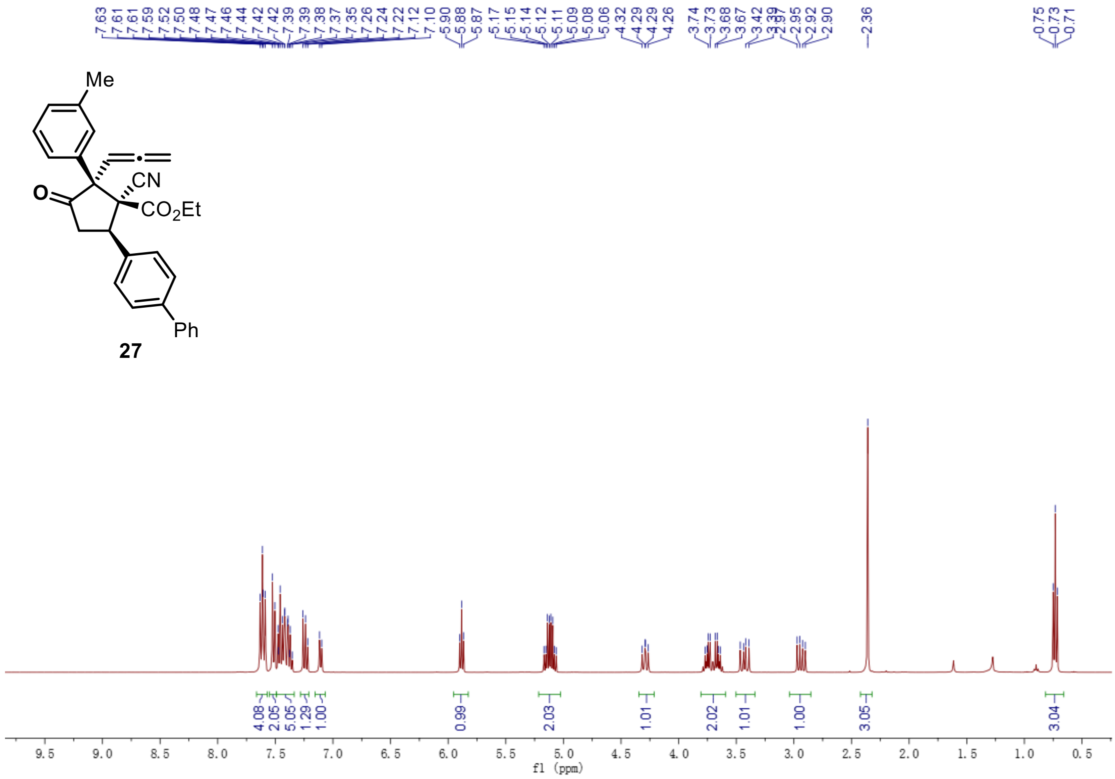

^{13}C NMR (100 MHz, $CDCl_3$)

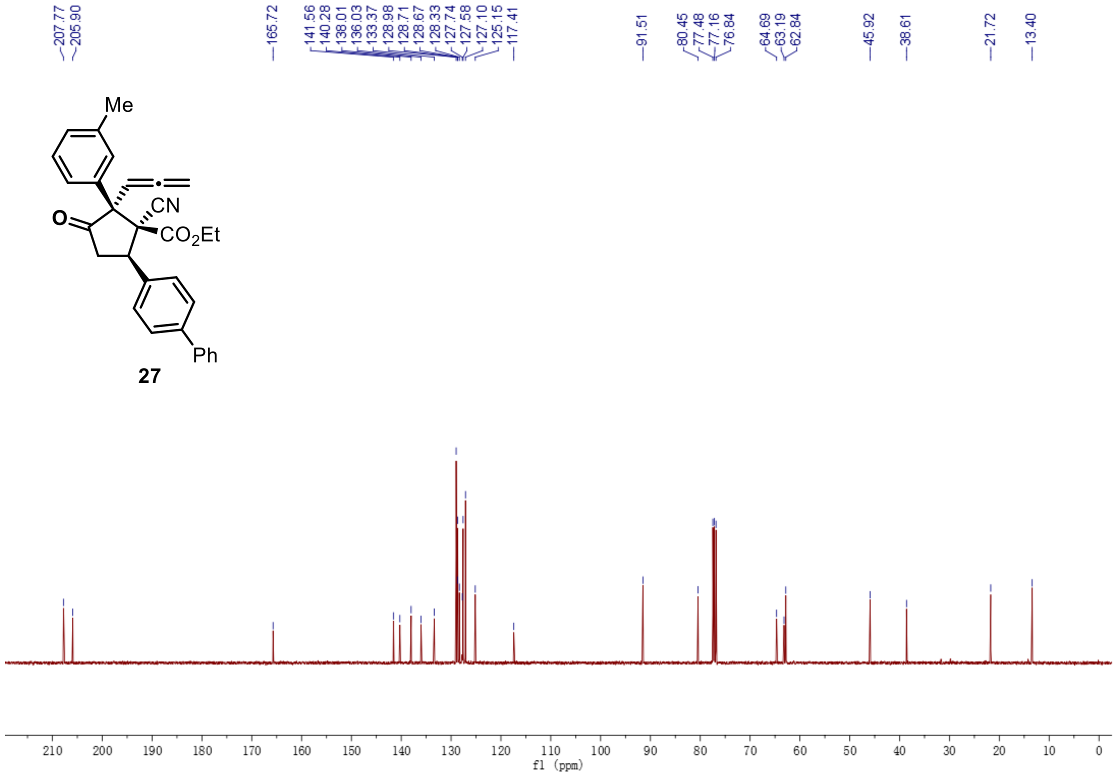

^{1}H NMR (400 MHz, $CDCl_3$)

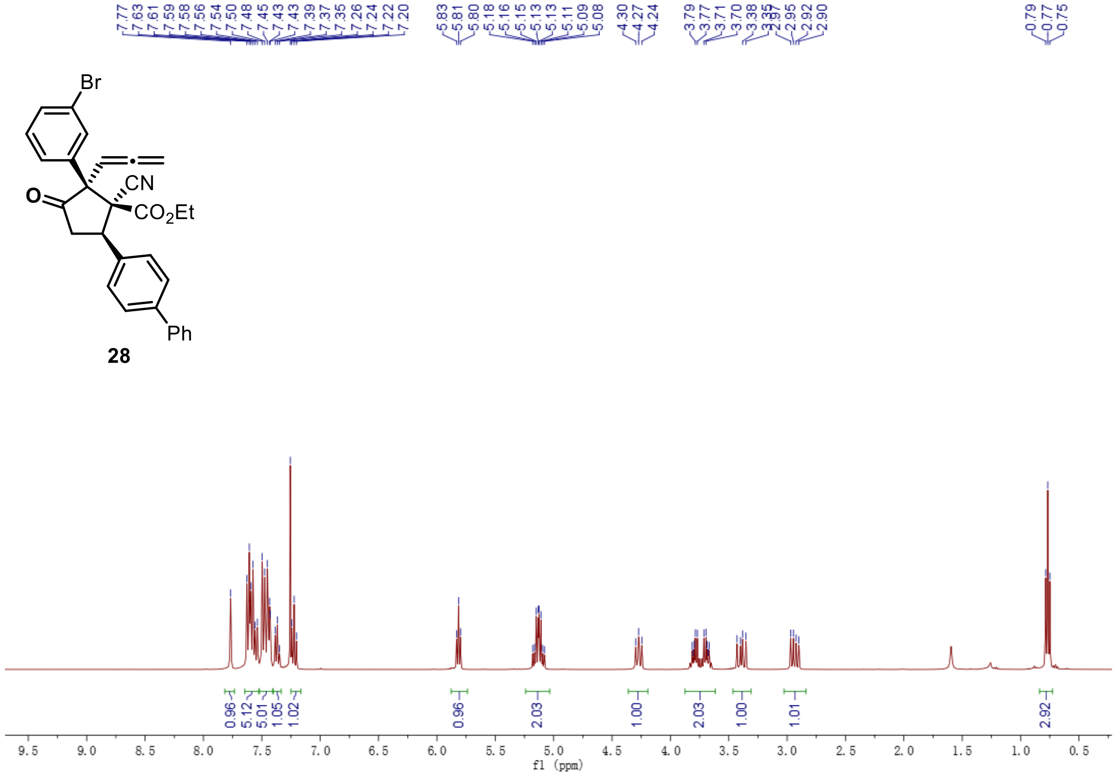

^{13}C NMR (100 MHz, $CDCl_3$)

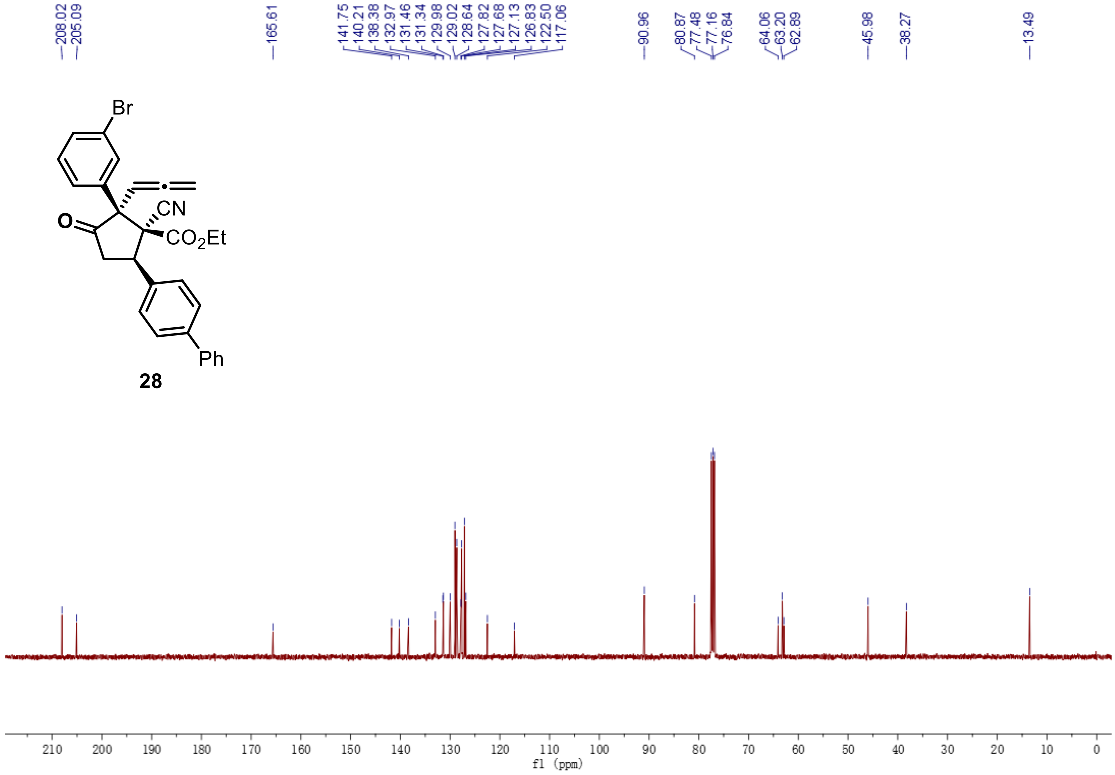

^{1}H NMR (400 MHz, $CDCl_3$)

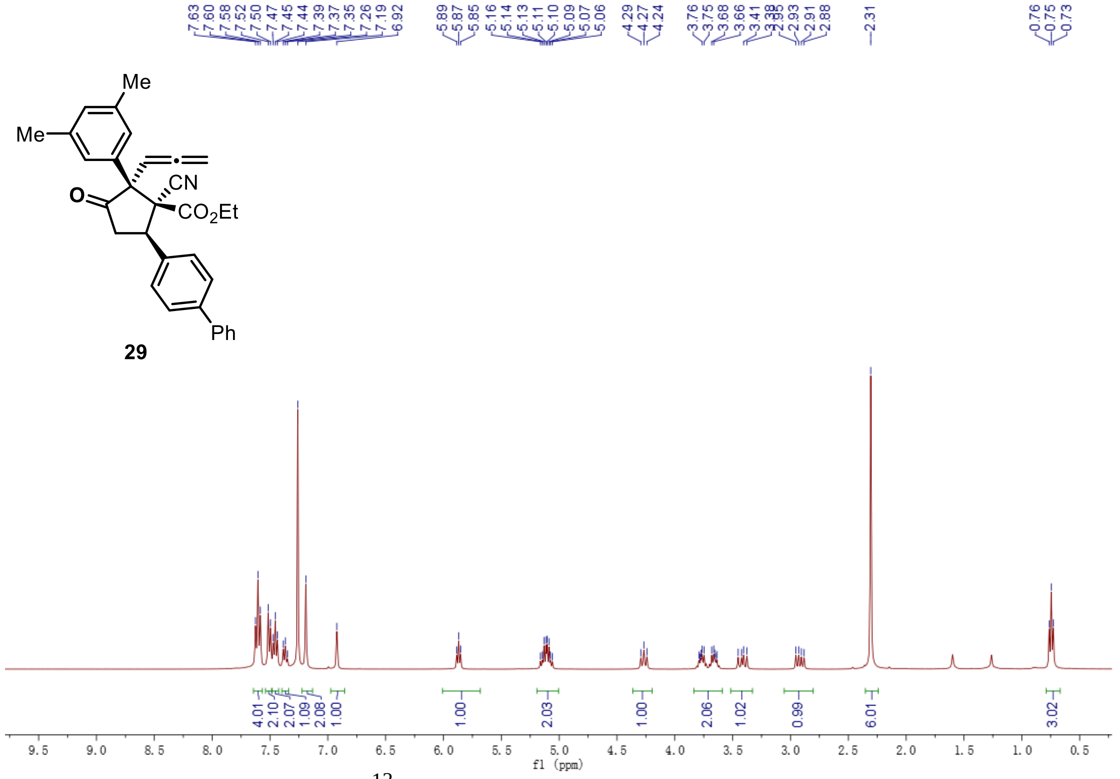

^{13}C NMR (100 MHz, $CDCl_3$)

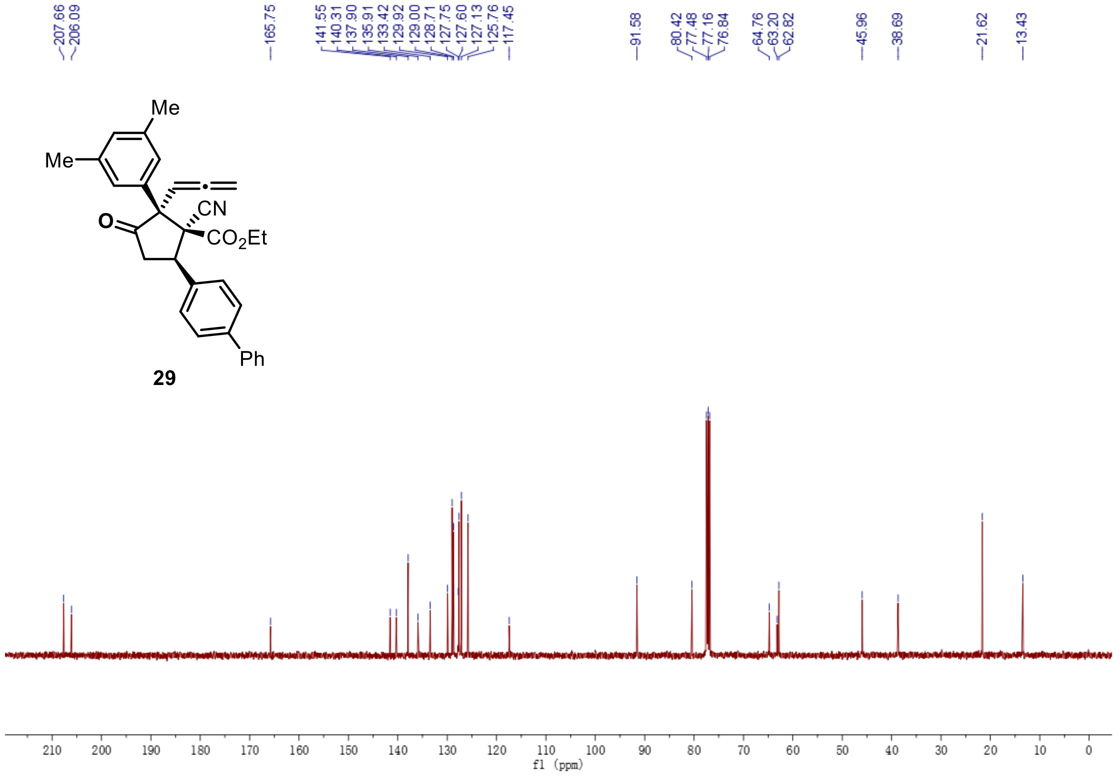

^{1}H NMR (400 MHz, $CDCl_3$)

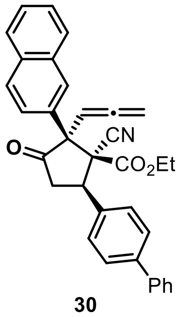

30

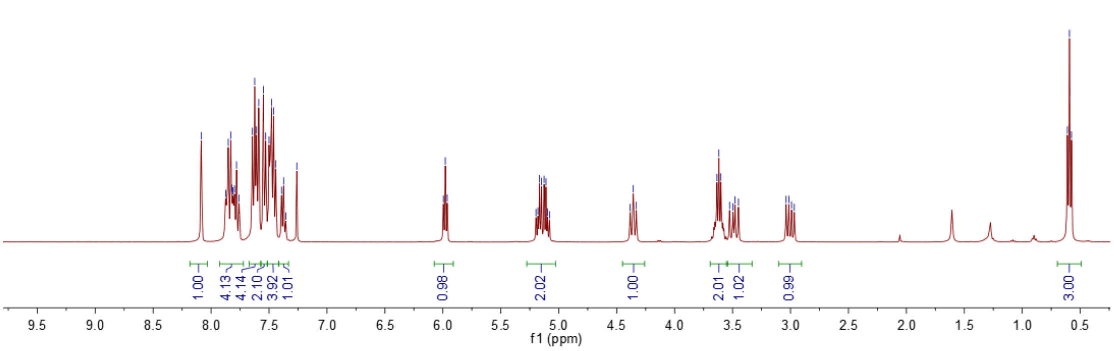

^{13}C NMR (100 MHz, $CDCl_3$)

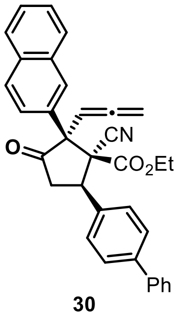

30

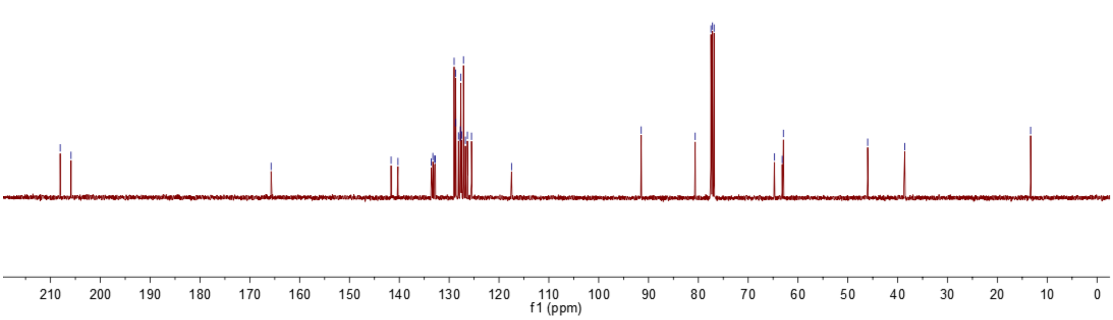

^1H NMR (400 MHz, $CDCl_3$)

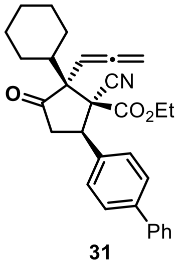

31

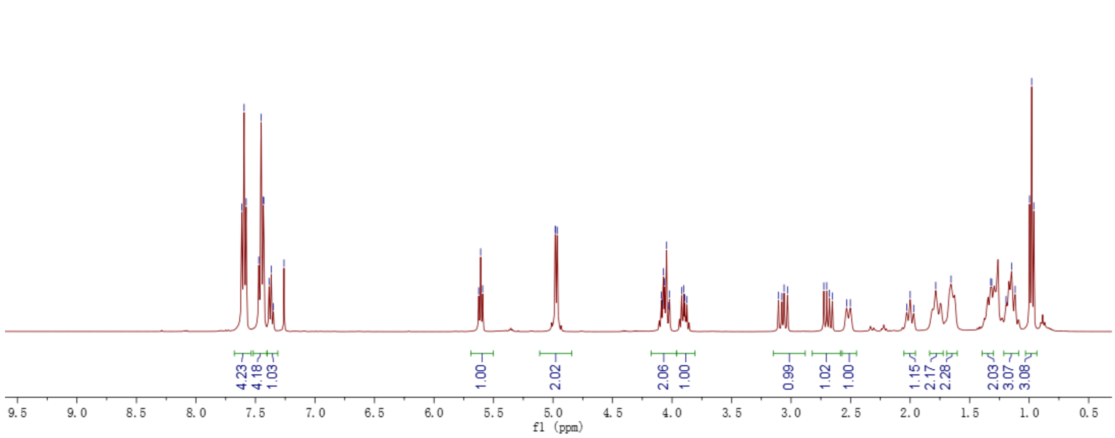

^{13}C NMR (100 MHz, $CDCl_3$)

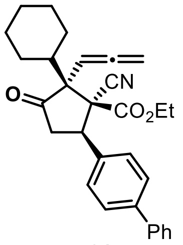

31

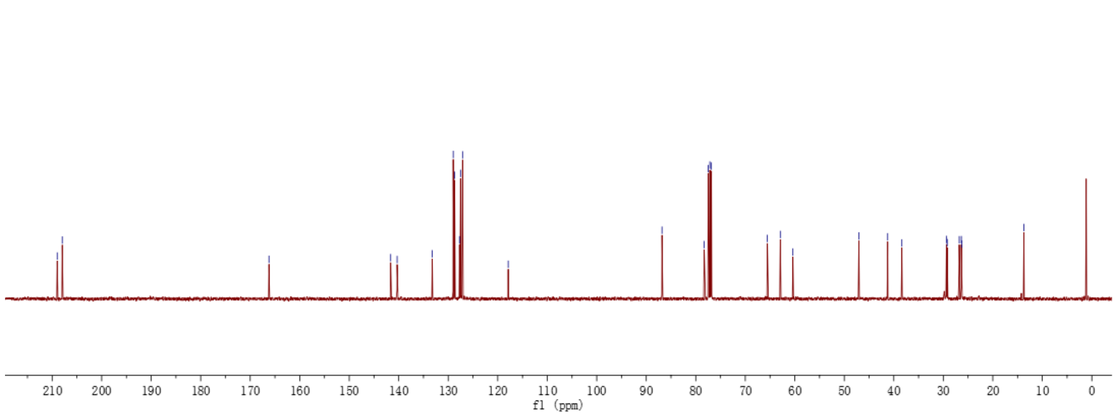

^{1}H NMR (400 MHz, $CDCl_3$)

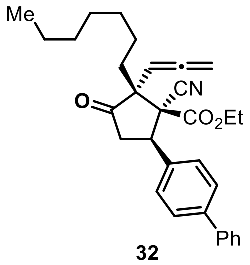

32

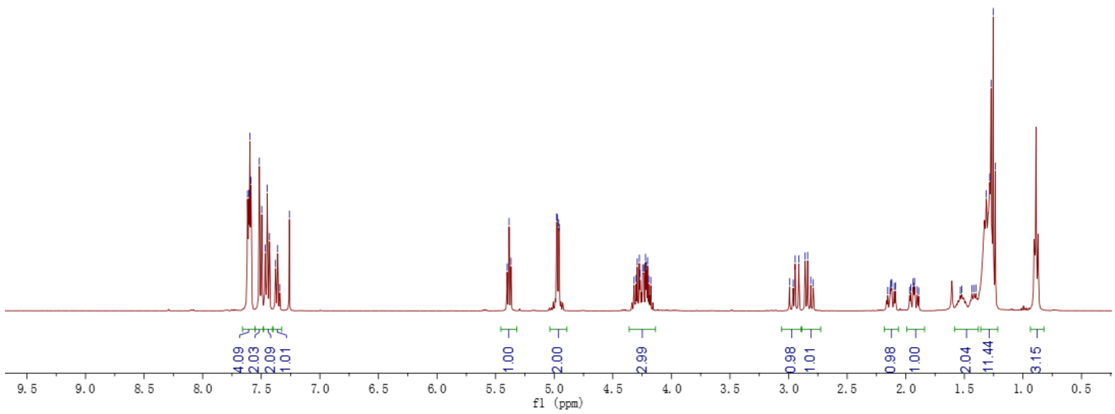

^{13}C NMR (100 MHz, $CDCl_3$)

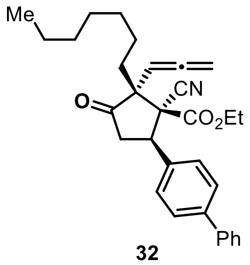

32

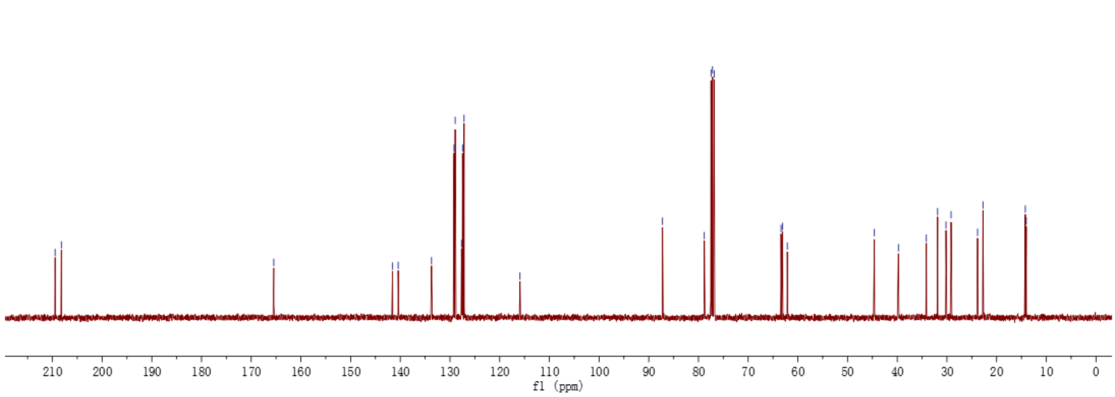

^{1}H NMR (400 MHz, $CDCl_3$)

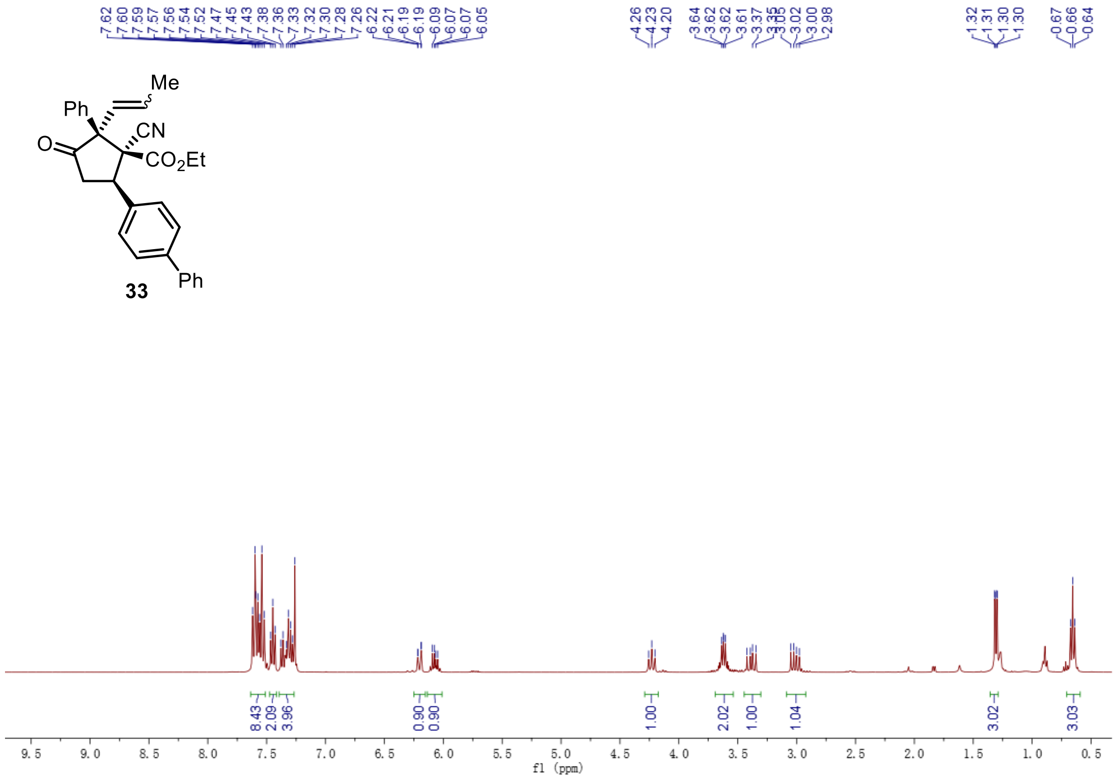

^{13}C NMR (100 MHz, $CDCl_3$)

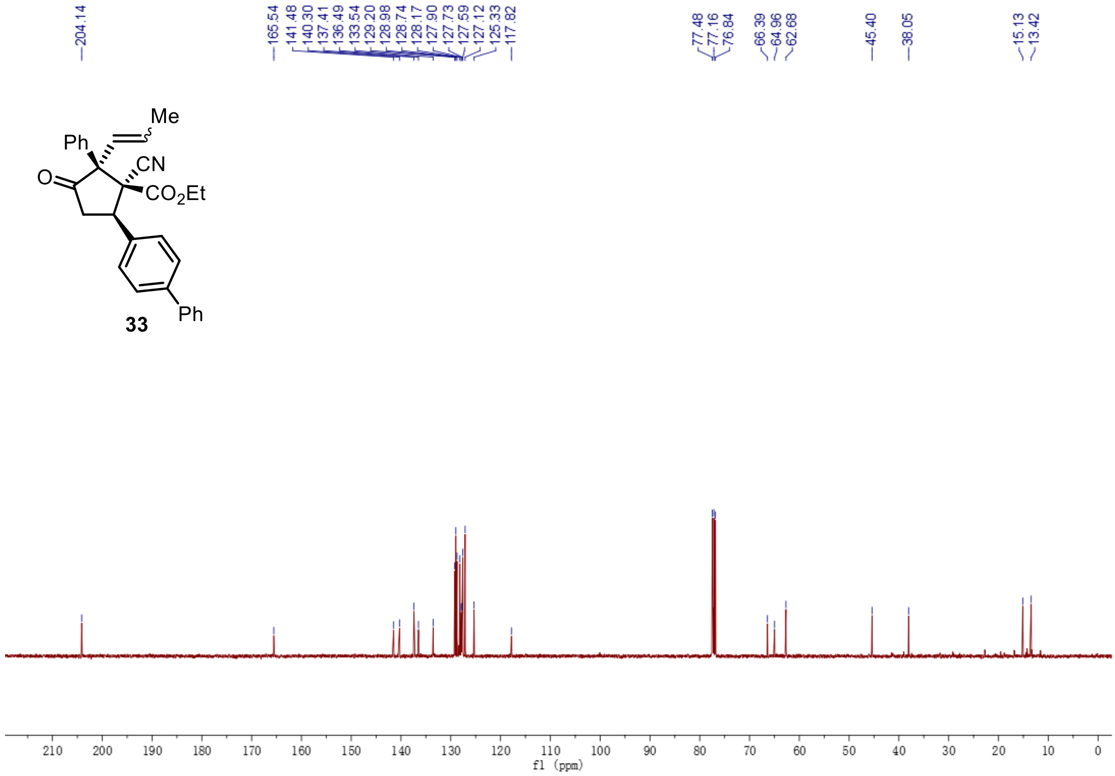

^{1}H NMR (400 MHz, $CDCl_3$)

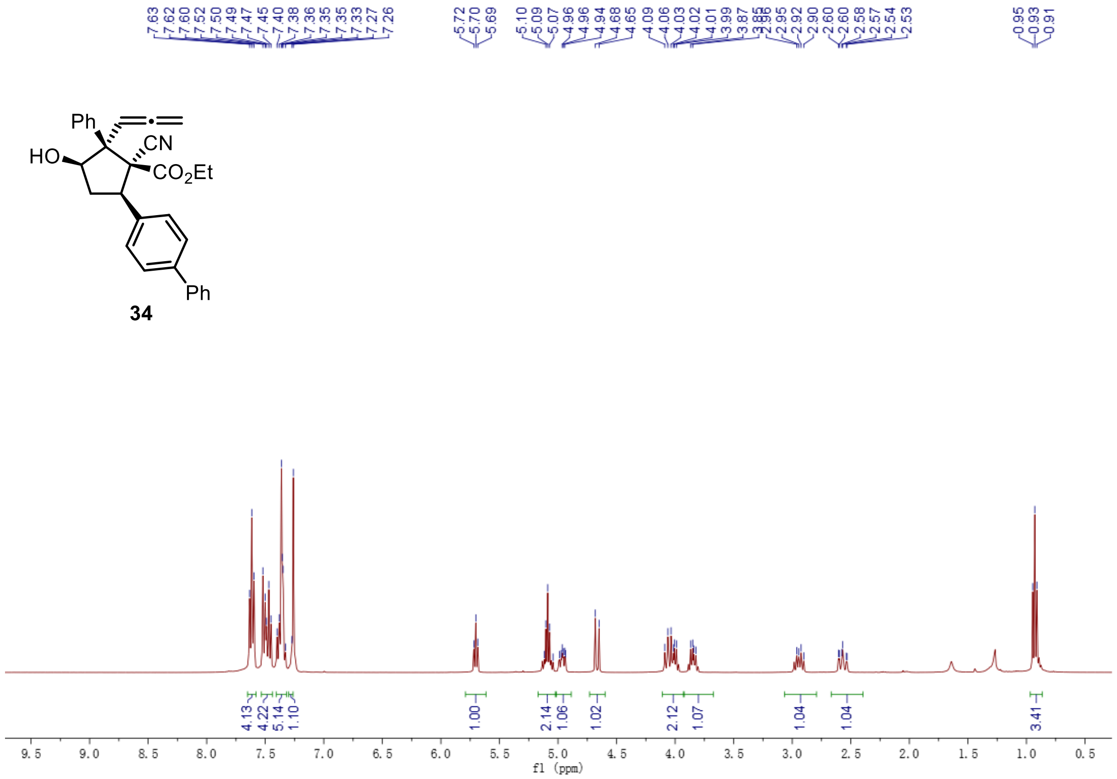

34

^{13}C NMR (100 MHz, $CDCl_3$)

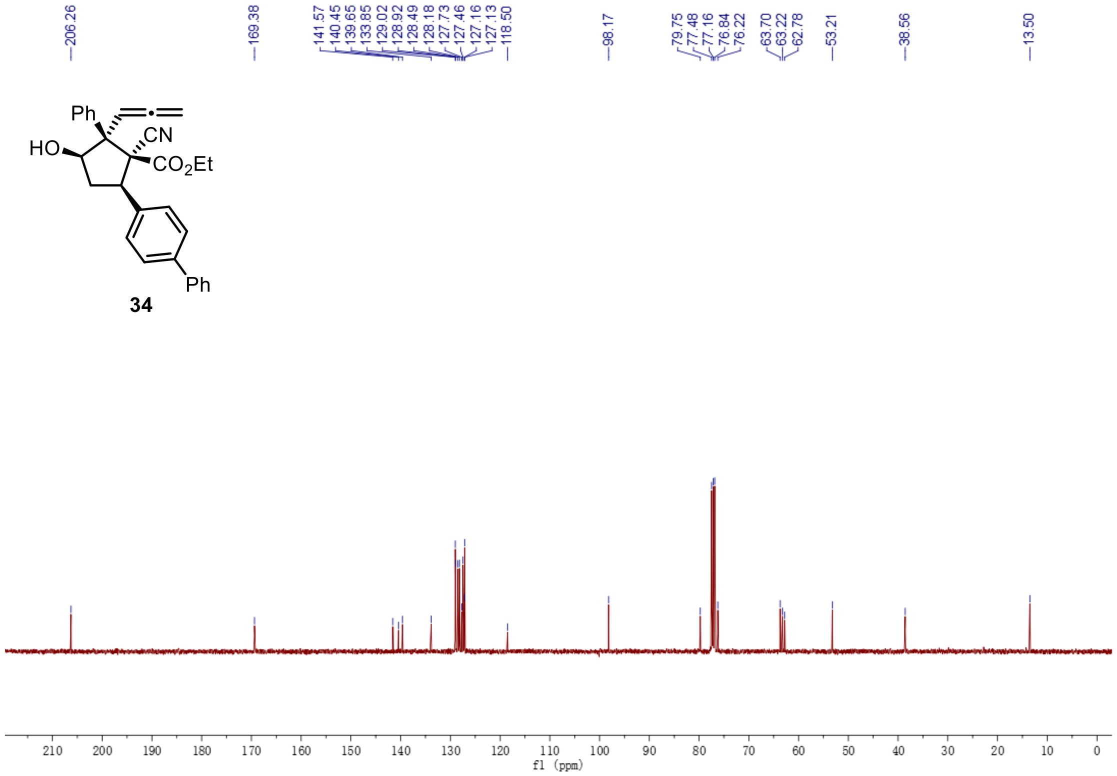

34

^{1}H NMR (400 MHz, $CDCl_3$)

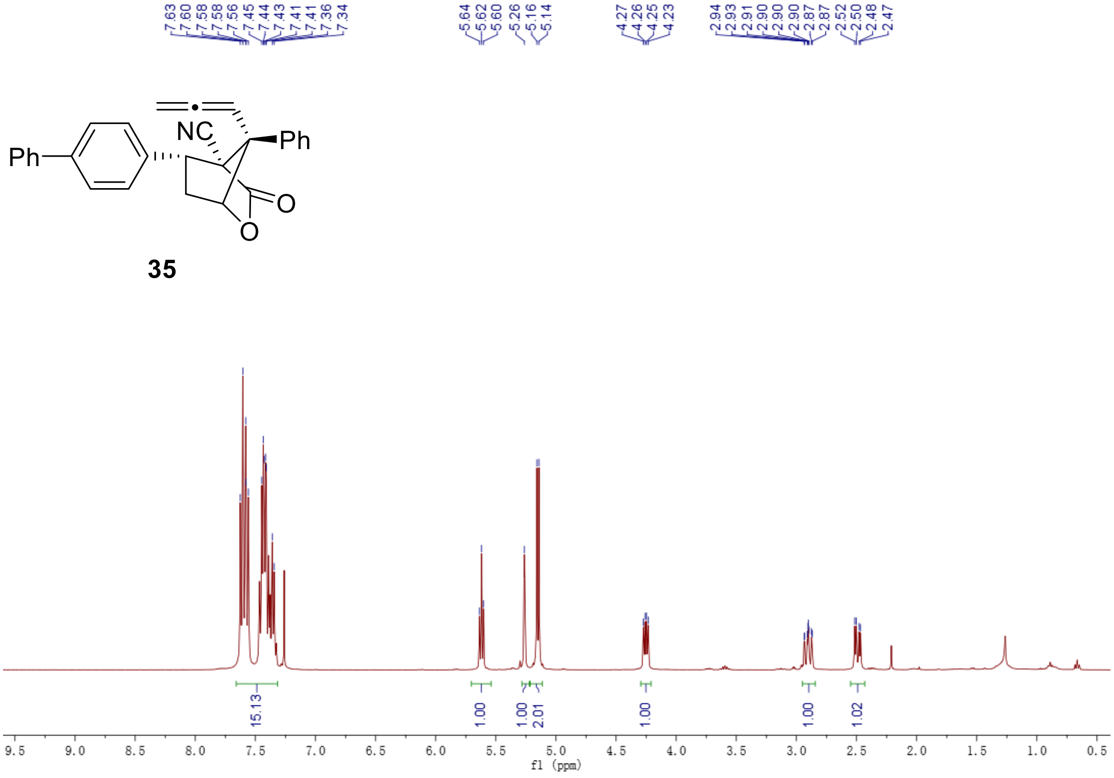

^{13}C NMR (100 MHz, $CDCl_3$)

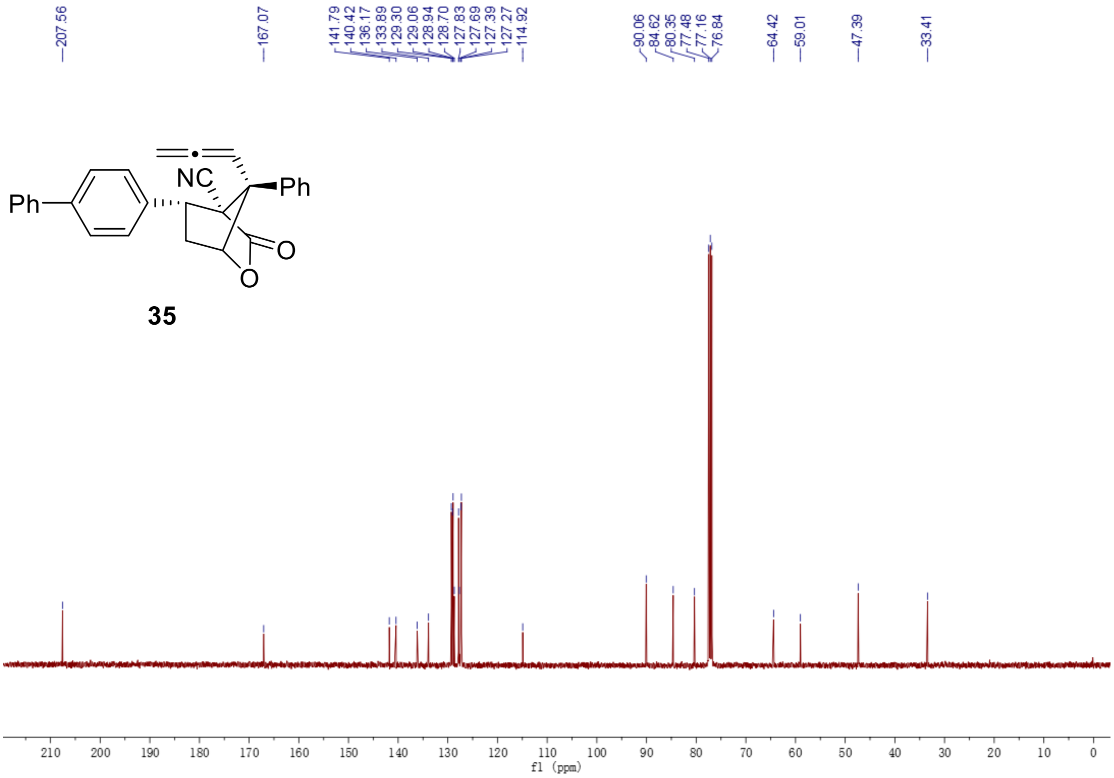

Supplement: SC-016-D4SC06849A-s001 [file SC-016-D4SC06849A-s001.pdf]
